# Discovery of Potent and Selective CB2 Agonists Utilizing a Function-Based Computational Screening Protocol

**DOI:** 10.1021/acschemneuro.3c00580

**Published:** 2023-10-12

**Authors:** Haixia Ge, Beihong Ji, Jiahui Fang, Jiayang Wang, Jing Li, Junmei Wang

**Affiliations:** † School of Life Sciences, Huzhou University, Huzhou 313000, China; ‡ Department of Pharmaceutical Sciences and Computational Chemical Genomics Screening Center, School of Pharmacy, University of Pittsburgh, Pittsburgh, Pennsylvania 15261, United States; § Chinese Academy of Sciences Key Laboratory of Receptor Research, National Center for Drug Screening, Shanghai Institute of Materia Medica, Chinese Academy of Sciences, Shanghai 201203, China

**Keywords:** function-based ligand design, receptor−ligand binding profile, CB1/CB2, structure−activity relationship

## Abstract

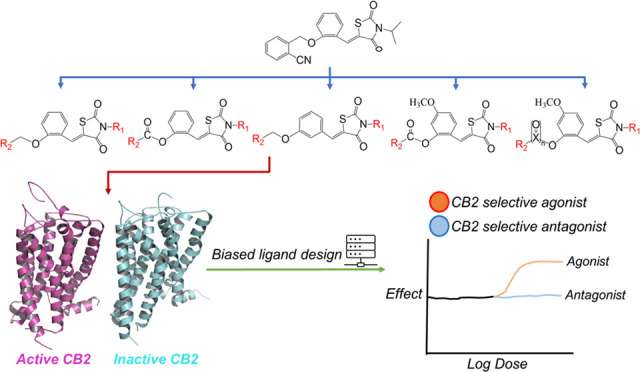

Nowadays, the identification of agonists and antagonists represents a great challenge in computer-aided drug design. In this work, we developed a computational protocol enabling us to design/screen novel chemicals that are likely to serve as selective CB2 agonists. The principle of this protocol is that by calculating the ligand–residue interaction profile (LRIP) of a ligand binding to a specific target, the agonist–antagonist function of a compound is then able to be determined after statistical analysis and free energy calculations. This computational protocol was successfully applied in CB2 agonist development starting from a lead compound, and a success rate of 70% was achieved. The functions of the synthesized derivatives were determined by in vitro functional assays. Moreover, the identified potent CB2 agonists and antagonists strongly interact with the key residues identified using the already known potent CB2 agonists/antagonists. The analysis of the interaction profile of compound **6**, a potent agonist, showed strong interactions with F2.61, I186, and F2.64, while compound **39**, a potent antagonist, showed strong interactions with L17, W6.48, V6.51, and C7.42. Still, some residues including V3.32, T3.33, S7.39, F183, W5.43, and I3.29 are hotspots for both CB2 agonists and antagonists. More significantly, we identified three hotspot residues in the loop, including I186 for agonists, L17 for antagonists, and F183 for both. These hotspot residues are typically not considered in CB1/CB2 rational ligand design. In conclusion, LRIP is a useful concept in rationally designing a compound to possess a certain function.

## Introduction

### CB1 and CB2 Receptors

The endocannabinoid system (ECS) is a complex and homeostatic system mainly located in both the central and peripheral systems and plays a central role in various physiological and pathological processes.^1−8^ ECS contains two major G protein-coupled receptors (GPCRs), which are cannabinoid receptors 1/2 (CB1/CB2), sharing sequence identities of 44% for the full-length amino acid sequence and 68% for the transmembrane domains.^9^ CB1 mainly concentrates in the central nervous system (CNS) with a small amount in areas outside the CNS.^10^ Its ligands can produce serious side effects on the CNS, such as psychotropic effects as agonists or anxiety, depression, and suicidal ideation as antagonists, which limits their clinical development.^11,12^ In contrast, CB2 is primarily expressed in the peripheral cells and tissues derived from the immune system^13^ and also present in low concentrations in the CNS and other cells.^14^ Thus, CB2-selective ligands, especially agonists, have drawn increasing attention to treat a number of disorders, while preventing the severe psychiatric side effects associated with CB1 in recent years. CB2 modulators have potential treatment capability for inflammatory, pain, neurodegenerative disorders, osteoporosis, fibrotic conditions, and cancers.^15−19^ Recently, several synthetic CB2-selective ligands have been reported;^20−28^ however, few have undergone clinical trials, and none have yet gained FDA approval. Thus, discovery of novel agonist ligands which are potent and highly selective among CB2 is in dire need.

### Function-Based Ligand Screen

As an important virtual screening approach, structure-based drug screening has been widely applied in designing and screening drug candidates, especially when high-quality drug–receptor complex structures are available.^29−32^ Recently, several crystal structures of cannabinoid receptors have been identified, including the antagonist-bound CB2^33^ and the agonist-bound CB2^34,35^ as well as the antagonist-bound CB1^36,37^ and the agonist-bound CB1^38^ complexes. These crystal structures paved the road for elucidating the molecular mechanisms of ligand–receptor interaction through molecular simulations and free energy analysis. However, most of the current structure-based design algorithms focus on the *de novo* design or screen potent ligands binding to a receptor. There are only limited reports about the functionality prediction of the ligand as an agonist or antagonist.^39,40^ In this work, we attempted to develop a computational protocol allowing us to develop and screen compounds that are likely acting as selective agonists of CB2. The computational protocol was validated by in vitro functional assays after compound syntheses.

### Lead Compound Identification and Protocol Validation

**Figure 1:**
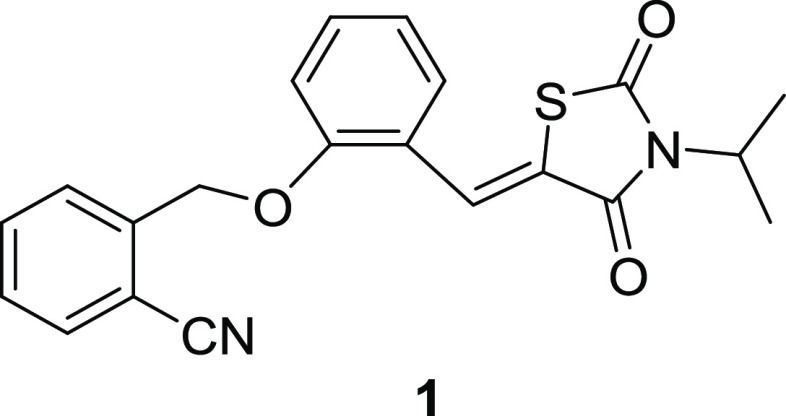
Structures of Compound **1**.

We performed hierarchical virtual screening to search for novel and selective CB2 agonists applying both the pharmacophore-based and docking-based filters against the SPECS database. Previously, we have performed hierarchical virtual screening combining pharmacophore-based and docking-based approaches against the SPECS database to search for novel and selective CB2 agonists. Through multiple virtual screenings and visual inspection, we identified a lead compound displaying >40% response against CHO–CB2 at a concentration of 10 μM by calcium assay. The lead compound (compound **1**, shown in Figure 1) demonstrates a moderate CB2 agonistic activity with an EC_50_ value of 3.12 ± 2.22 μM but no bioactivity against CB1 at a concentration of 10 μM. Thus, compound **1** is a novel selective CB2 agonist with decent drug developability. To validate the function-based ligand screening protocol using the ligand–residue interaction profile (LRIP), **39** compounds were rationally designed and synthesized in four compound series. The CB1/CB2 functional activities of these compounds were assessed by the cell-based calcium mobilization assays.^41,42^ Then, we applied the LRIP function-based ligand screening protocol to predict the functionality of each designed compound and three compounds (AM630, CP55940, compound **1**) with known functions.

## Results

### Chemical Synthesis

**Scheme 1:**
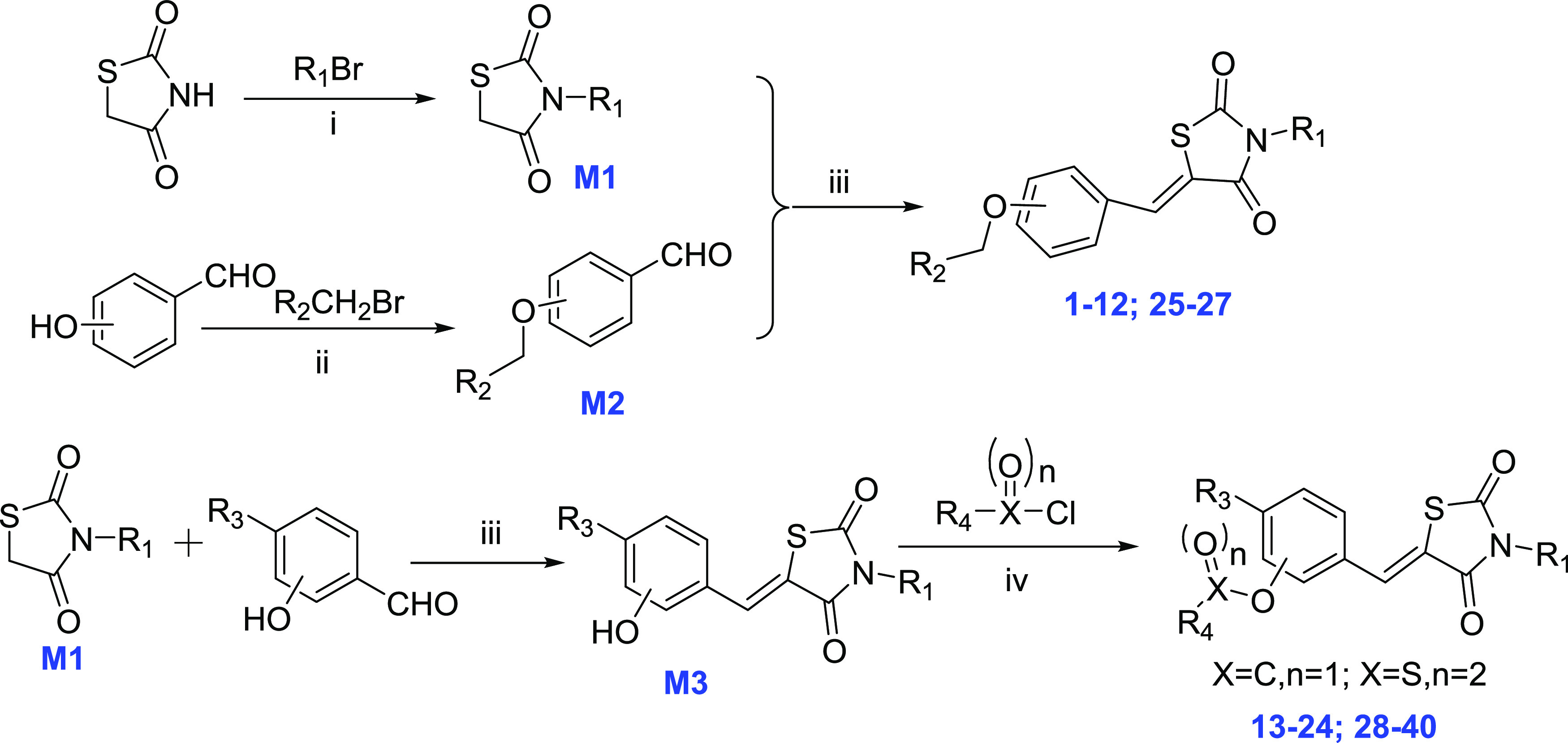
General Synthesis of Compound Series Reagents and conditions: (i) KOH, DMF, reflux, 4 h; (ii) Cs_2_CO3_,_ DMF, 80 °C, 6 h; (iii) 4-methylpiperidine acetate, toluene, reflux, 6 h; and (iv) Et_3_N, CH_2_Cl_2_, rt, 4 h.

The synthetic routes to obtain target compounds are outlined in Scheme 1. The commercially available 2,4-thiazolidinedione was reacted with selected bromides in DMF to give the key intermediate M1. Hydroxybenzaldehyde with diverse substituents was reacted with different bromides to obtain another key intermediate M2. Finally, the coupling reaction between M1 and M2 yielded the corresponding compounds **1**–**12** and **25**–**27**. The intermediate M3 was synthesized by the condensation reaction of M1 and hydroxybenzaldehyde with diverse substituents and then reacted with selected acyl chloride or sulfonyl chloride to obtain the compounds **13**–**24** and **28**–**40**. The structures of target compounds **1**–**40** were characterized and validated by high-resolution mass spectrometry (HR-MS), ^13^C NMR, and ^1^H NMR.

### Function-Based Screening Protocol

Both agonists and antagonists can induce conformational changes in the receptor, but their effects on the downstream signaling pathways differ. Agonists stabilize conformational states of the receptor that promote the activation of downstream signaling pathways such as coupling with G proteins. These conformational changes allow the receptor to interact with intracellular signaling proteins and initiate a cascade of events, leading to cellular responses. On the other hand, antagonists also bind to the receptor, but they stabilize conformational states that prevent or reduce the activation of downstream signaling pathways. They may block the binding site of agonists or inhibit the conformational changes necessary for G protein coupling and subsequent signaling activation.

In this study, our primary focus is on agonist design due to its significant scientific and therapeutic implications. Agonists play a crucial role in activating specific signaling pathways and eliciting the desired cellular responses through dynamic conformational changes in the receptor. These conformational changes facilitate the interaction of the receptor with intracellular signaling proteins, initiating a cascade of events that lead to cellular responses.

**Figure 2:**
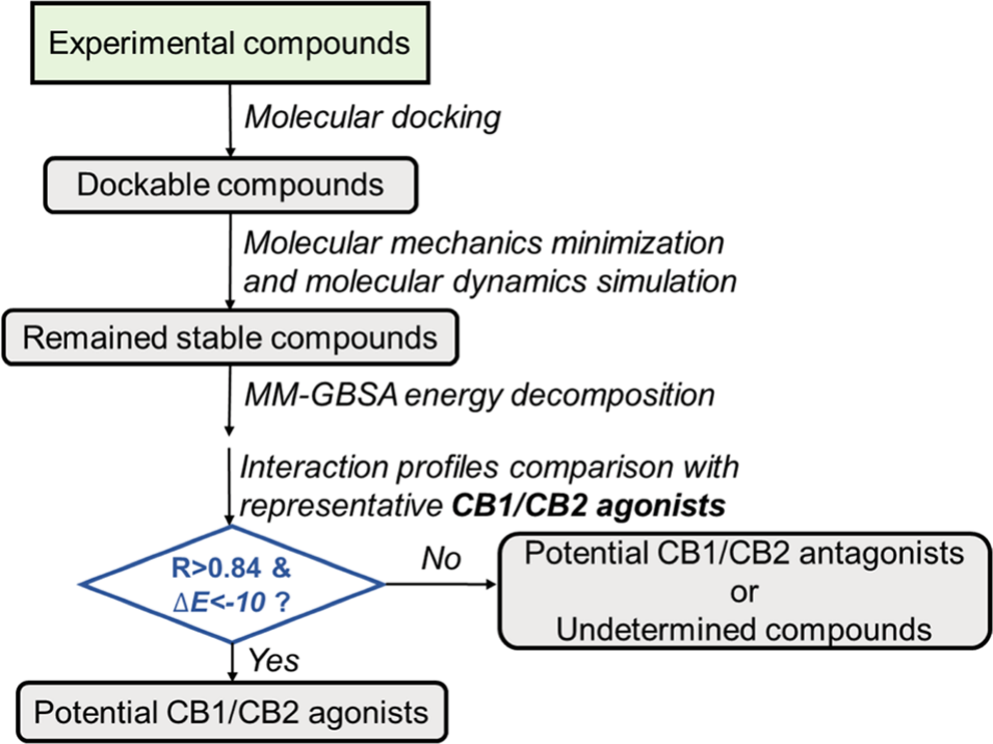
Flowchart for the determination of binding and function selectivity.

As shown in Figure 2, the function of an experimental compound is predicted by utilizing a series of molecular modeling techniques, which include molecular docking, molecular dynamics (MD) simulations, LRIP calculations after molecular mechanics generalized Born surface area (MM-GBSA) binding free energy decomposition analysis, and end-point molecular mechanics Poisson–Boltzmann surface area WSAS (MM-PBSA-WSAS) binding free energy calculations. Note that WSAS is an efficient approach for calculating the entropy contribution TΔ*S* due to the ligand binding.^43^ The protocol consists of the following steps: (i) a designed molecule is docked to the drug target, CB1 or CB2, and the molecule survives if it has a decent docking score; (ii) a molecular simulation system is prepared with the best docking pose, and the ligand–receptor complex is immersed in a water box for running explicit water MD simulations; (iii) if the molecule is stable during the MD simulations, a couple thousands of MD snapshots are collected for MM-GBSA binding free energy decomposition analysis; (iv) LRIP is generated and compared to IPs of representative CB1 or CB2 known modulators (agonists for active receptors and antagonists for inactive receptors). If the correlation coefficient *R* of active CB1/CB2 is larger than a threshold, 0.84 for this work, and at the same time, binding energy Δ*E* is better than −10 kcal/mol, then, the molecule is recognized as a CB2 agonist; and (v) if the molecule is not an agonist, it is recognized as a CB antagonist or an undetermined compound. To be noted, in this study, our primary focus is on agonist design due to its significance and promise in therapeutic implications. Agonists play a crucial role in activating specific signaling pathways and eliciting desired cellular responses through dynamic conformational changes in the receptor. These conformational changes facilitate the interaction of the receptor with intracellular signaling proteins, initiating a cascade of events, leading to cellular responses. It is also noted that the undetermined compounds are those that cannot be explicitly determined as agonists or antagonists of the receptor. In other words, these compounds may have no interaction with the receptor or their activities are too weak to be measured.

It also needs to be pointed out that the thresholds applied in the flowchart may need to be changed for other drug targets. The comparison results of IP between the evaluated compounds (include CP55940 and AM630) and representative agonists/antagonists are shown in Tables S1–S4. According to the calculated *R* results and energies, overall, there are **26** compounds predicted to be CB2 agonists and **16** compounds predicted as antagonists or undetermined compounds. It is worth noting that the correlation between the IPs of two agonists is typically higher than that of two antagonists since agonist binding typically triggers downstream signaling, which requires coherent movement of the surrounding residues. However, due to the limitation of the end-point free energy method, MM-GBSA, exceptions can occasionally occur. For example, CP55940 is a nonselective agonist for both cannabinoid receptors, but its *R* values are slightly higher when compared to the antagonist IPs (Tables S3 and S4) than to the agonist IPs (Tables S1 and S2).

### In Vitro Evaluation of Biological Activity

Calcium mobilization assay with fluorescent dyes is a highly sensitive and easy-to-handle method that has been widely applied to the study of GPCR ligands. In the previous work, we have developed a robust high-throughput screening assay based on Gα15/16-mediated calcium mobilization to identify novel modulators of GPCR.^44^ All the synthesized compounds were evaluated in vitro for their functional activities by using these developed cell-based calcium mobilization assays. The synthetic cannabinoid CP55940 is a well-documented, high-affinity, and nonselective cannabinoid receptor agonist and served as a positive control for the comparison of agonist activities of CB1 and CB2. On the other hand, rimonabant and AM630 served as positive controls for the comparison of antagonist activities of CB1 and CB2, respectively, while DMSO served as a negative control. Dose–response studies were conducted to obtain the EC_50_ or IC_50_ values of all compounds by evaluating their functional effects for the CB1/CB2 receptors. Positive control compounds were used in every independent experiment, and the results of positive control compounds were similar every time. The structures of the synthesized compounds as well as the bioactive results are displayed in Tables 1–5. Encouragingly, 14 compounds were identified as novel CB2 agonists with better activities than the lead compound **1**. Moreover, we also discovered three CB2-selective antagonists, among which compounds **38** and **39** have IC_50_ values of 0.40 ± 0.16 μM and 0.33 ± 0.09 μM, respectively, exhibiting a similar effect as AM630 (IC_50_ = 0.34 ± 0.10 μM), a known CB2-selective antagonist.

**Table 1:**
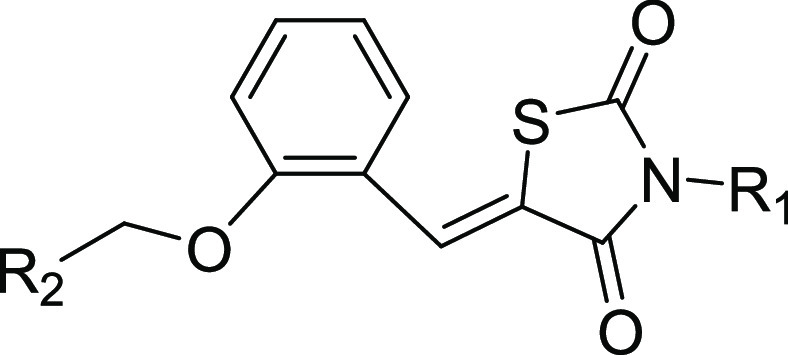

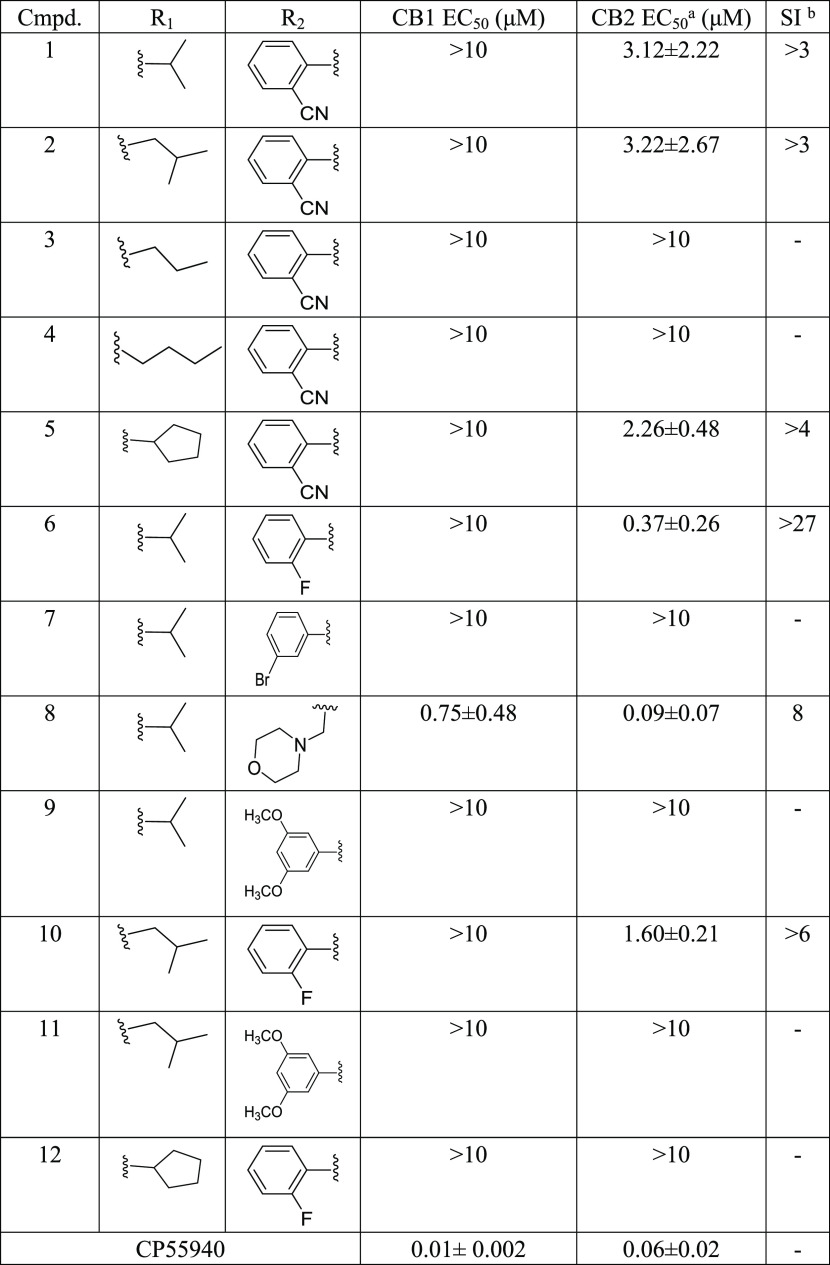
Agonist Activities of CP55940 and Compounds **1**–**12** in CHO Expressing the CB1 or CB2 Receptor Determined by Calcium Mobilization Assays

a EC_50_ values were obtained from three independent calcium mobilization assays with three repetitions and eight gradients for each time.

b SI: selectivity index for the CB2, SI = EC_50_(CB1)/EC_50_ (CB2).

### Evaluation of the Computational Protocol

**Figure 3:**
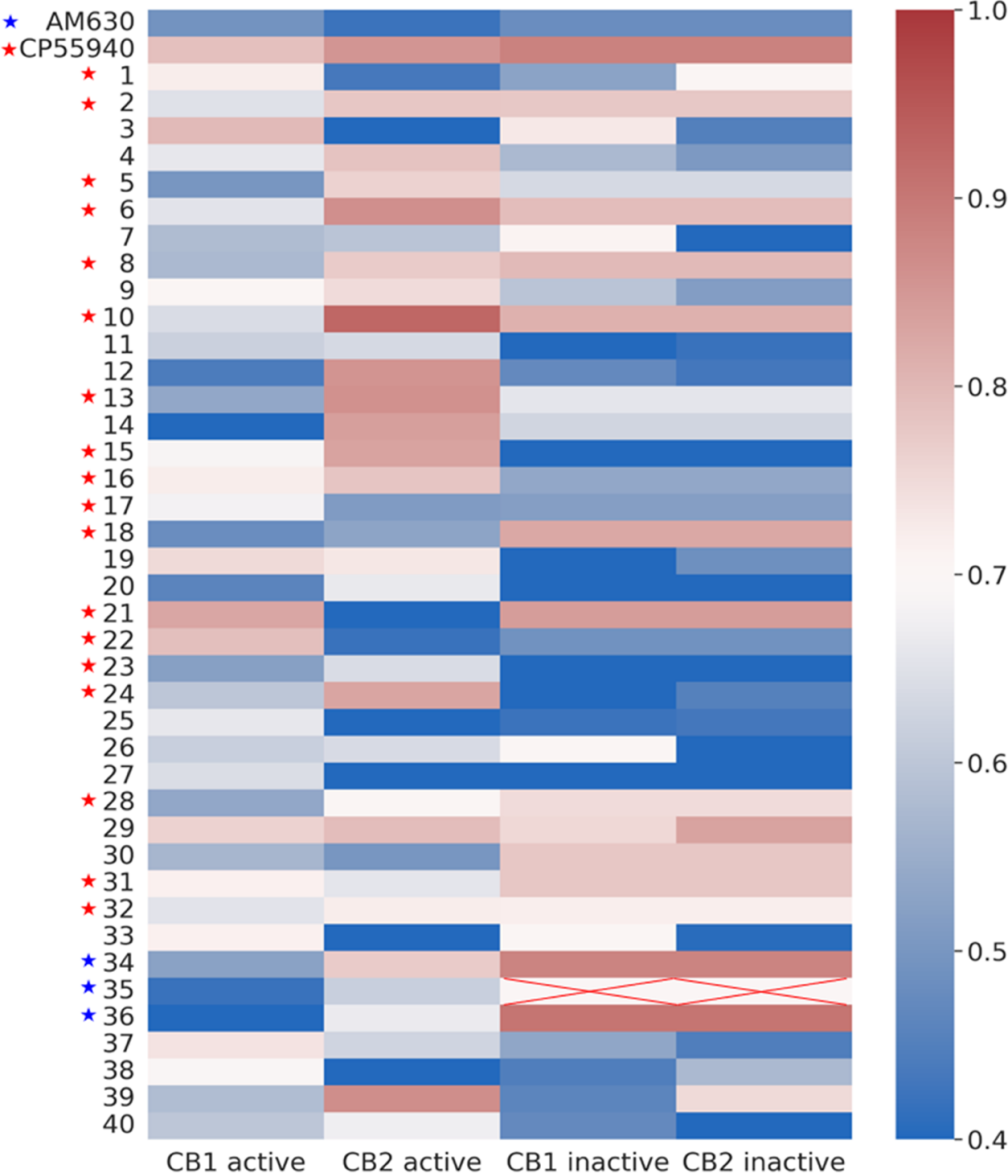
Calculated correlation coefficients for control and experimental compounds. Blue stars represent compounds that are evaluated as antagonists in functional assays, and red stars represent compounds that are evaluated as agonists in functional assays. The cell with cross means that the value is unavailable.

**Figure 4:**
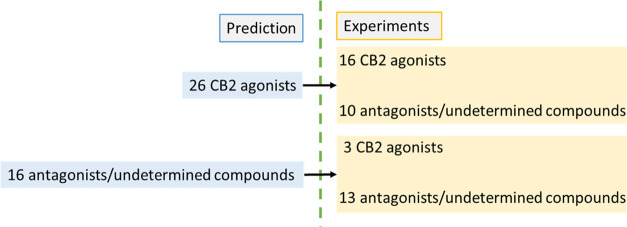
Comparison diagram of predicted and experimental activities.

To evaluate the protocol, the measured activities from the functional assay and prediction results were compared. The correctly predicted compounds are highlighted in Tables S1–S4, and the description of the calculated *R* values is shown in Figure 3. It is noted that 26 compounds were predicted to be CB2-selective agonists, and among them, 16 were correctly predicted. On the contrary, **16** compounds were predicted to be CB1/CB2 antagonists or their functions were undermined. Among them, 3 were correctly predicted as CB2 antagonists and 10 were correctly predicted as inactive compounds. In summary, the functions of 29 out of 42 compounds were correctly predicted, and the overall prediction accuracy is 69%. As for CB2-selective agonist prediction, the success rate is 62%, indicating that this computational protocol can guide CB2-selective agonist design. The overall prediction results are summarized in Figure 4.

## Discussion

### Structure–Activity Relationship (SAR) Analysis

On the basis of the lead compound **1**, we first synthesized a series of benzylidenethiazolidinedione derivatives with different substituents on R1 and R2 for investigating the influence of the variable on activities. As shown in Table 1, compounds **2**–**5** with different R1 substituents and the same R2 group of *o*-nitrile phenyl showed significant differences in activity toward CB2. The R1 groups of compounds **2** and **5** were, respectively, substituted with isobutyl and cyclopentyl groups, and their CB2 agonistic activities are marginally improved in comparison with compound **1**. When R1 is substituted by other smaller groups, such as *n*-propyl and *n*-butyl, or a bigger group such as cyclohexyl, the compounds showed no CB1 and CB2 activities at the receptor concentration of 10 μM. Therefore, R1 with an adequate size is critical and only isopropyl, isobutyl, and cyclopentyl are considered in the subsequent structural modifications. Compounds **6**, **10**, and **12** have the same R2 group of *o*-fluorophenyl, while R1 was substituted by the aforementioned three substituents. Compound **6** exhibits better CB2 agonistic activity than that of compound **10**, with their EC_50_ values of 0.37 ± 0.26 μM and 1.60 ± 0.21 μM, respectively, while compound **12** has no bioactivity under the conditions of our assays. Therefore, we speculated that the two branched aliphatic R1 groups, isopropyl and isobutyl, are more desirable to improve the bioactivities. Next, we explored different R2 groups, with R1 being isopropyl. Compound **8** with the R2 group of 4-methylmorpholine illustrated the best CB2 agonist activity so far with an EC_50_ of 0.09 ± 0.07 μM. Interestingly, this compound also has a strong CB1 agonist effect with an EC_50_ of 0.75 ± 0.48 μM. In contrast, compounds **7** and **9** do not show biological activities. Overall, when R2 is a phenyl group with an electron-withdrawing group at the ortho position, the compound is likely to have CB2 but no CB1 agonist activity.

Considering that the activities of both CB1 and CB2 were enhanced when the R2 group is a nonaromatic substituent as in compound **8**, we further explored R2 substitution with R1 being either isopropyl or isobutyl. A variety of structurally diverse R2 substituents were designed, and some were synthesized. Unlike compounds in Table 1 having R2 substitution on anisole, compounds in Table 2 have R2 substitution on phenyl formate. As expected, compounds **15** and **17** exhibited good CB2 agonistic activities; compound **23**, a more hydrophobic compound with a larger size than that of compounds **15** and **17**, also achieves a strong CB2 agonist activity and selectivity against CB1. Overall, for this compound series, a set of structurally diverse R2 substituents may achieve decent CB2 agonist activities and selectivity. However, it is hard to reach a conclusion on the requirement of R2′s size and overall electronic characteristics.

**Table 2:**
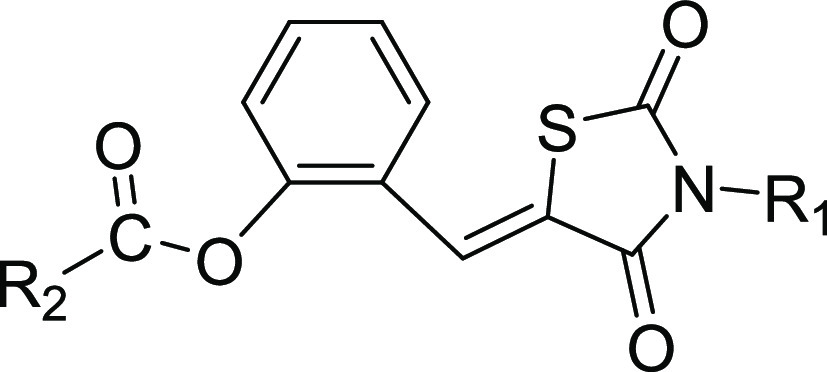

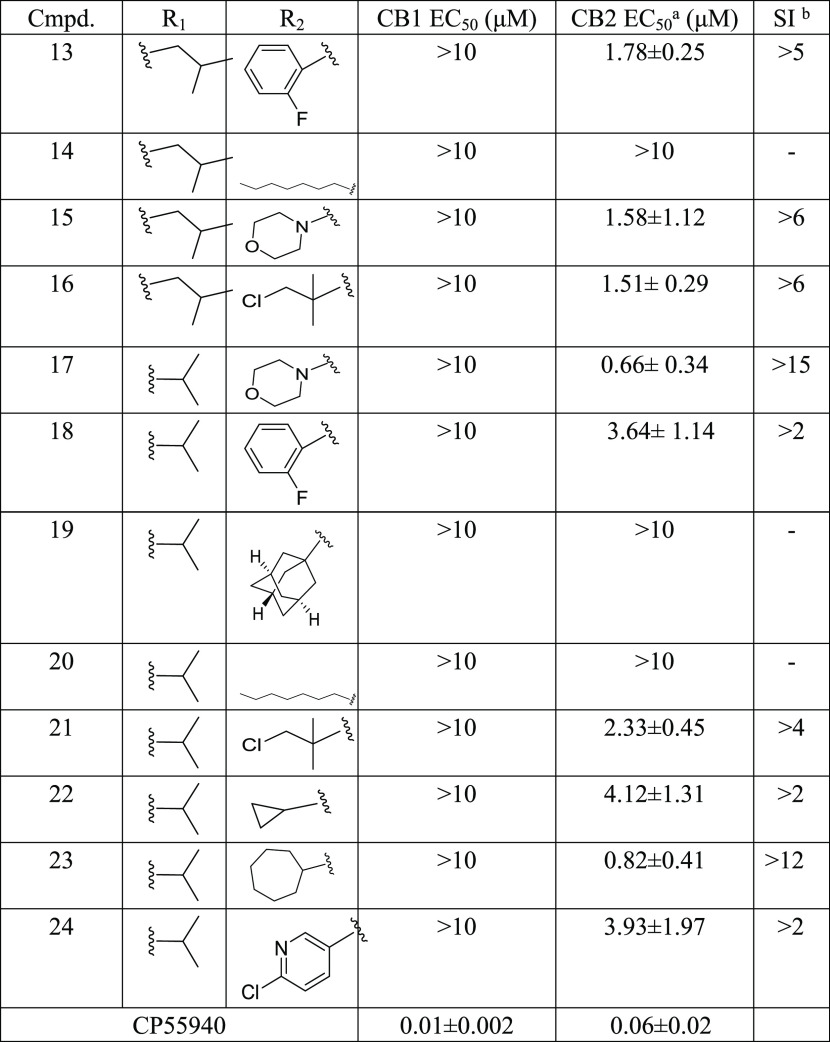
Agonist Activities of Compounds **13**–**24** in CHO Expressing CB1 or CB2 Receptor Determined by Calcium Mobilization Assays

a EC_50_ values were obtained from three independent calcium mobilization assays with three repetitions and eight gradients for each time.

b SI: selectivity index for the CB2, SI = EC_50_(CB1)/EC_50_ (CB2).

Next, we evaluated a new scaffold for which the R2-substituted ether group and the thiazolidinedione group take the meta positions of the central benzene ring. As shown in Table 3, none of the synthesized compounds (**25**–**27**) show either CB1 or CB2 activities. It seems that the two groups taking the ortho positions of the central benzene ring are required for a compound to be an CB2 or CB1 agonist.

**Table 3:**
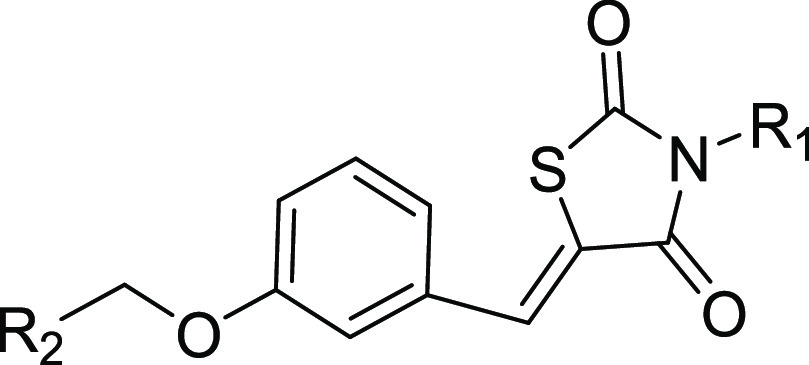

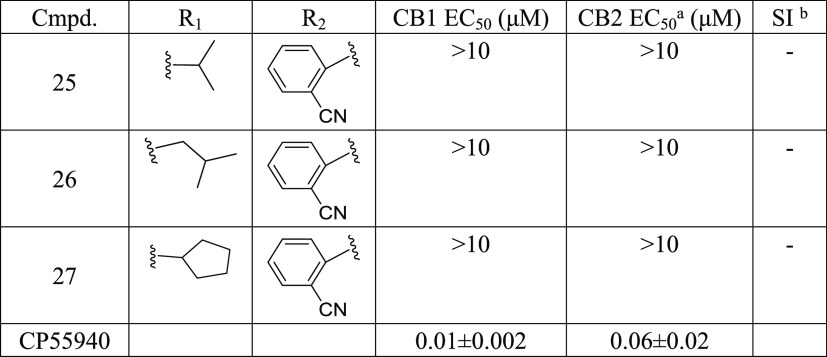
Agonist Activities of CP55940 and Compounds **25**–**27** in CHO Expressing CB1 or CB2 Receptors Determined by Calcium Mobilization Assays

Then, we explored a new compound series by replacing the anisole with phenyl formate at the R2 substitute site and introduced a methoxy group to the central phenyl ring at the para position of the thiazolidinedione group. As shown in Table 4, compounds **34**–**37** with R1 being isobutyl proved to be inactive analogues on CB2, while when R1 was substituted by isopropyl, compounds **28**, **31**, and **32** show certain CB2 agonistic activity with EC_50_ values between 0.83 and 3.38 μM. For this compound series, all compounds have no CB1 agonist activities.

**Table 4:**
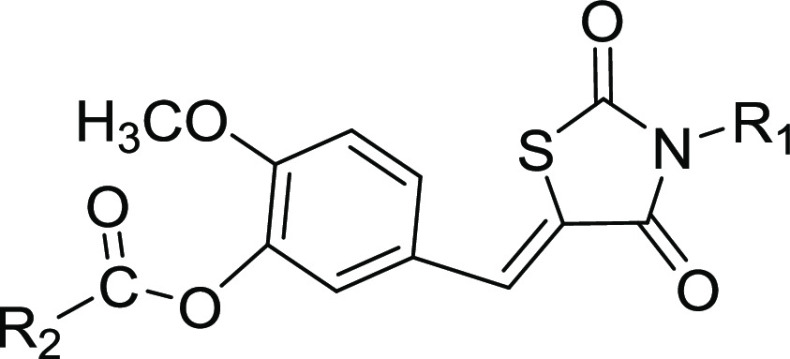

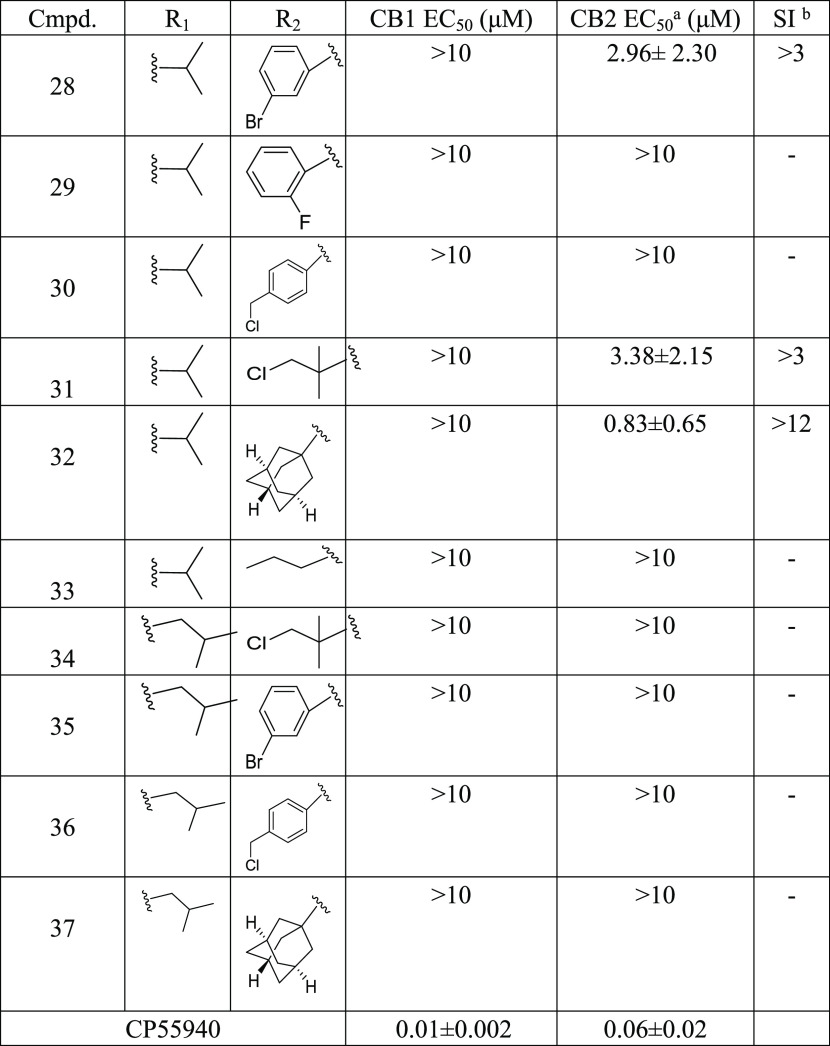
Agonist Activities of CP55940 and Compounds **28**–**37** in CHO Expressing CB1 or CB2 Receptors Determined by Calcium Mobilization Assays

a EC_50_ values were obtained from three independent calcium mobilization assays with three repetitions and eight gradients for each time.

b SI: selectivity index for the CB2, SI = EC_50_(CB1)/EC_50_ (CB2).

Last, we explored a new scaffold by replacing the ester group with carbonyl and sulfone groups. The activities of these compounds are summarized in Table 5. Very interestingly, we found that analogues **38–40** were CB2 antagonists instead of agonists; especially, compounds **38** and **39** exhibit excellent antagonist activities on CB2 with IC_50_ values of 0.40 ± 0.16 μM and 0.33 ± 0.09 μM, respectively, which are comparable to that of AM630 (IC_50_ = 0.34 ± 0.10 μM), a known CB2 antagonist. Moreover, compounds **38** and **39** have decent selectivity against CB1 with SI values of 25 and 23, respectively. Compound **39** has a much higher potency than that of **40**, indicating that the potent CB2 antagonists prefer a smaller R1 substituent group.

**Table 5:**
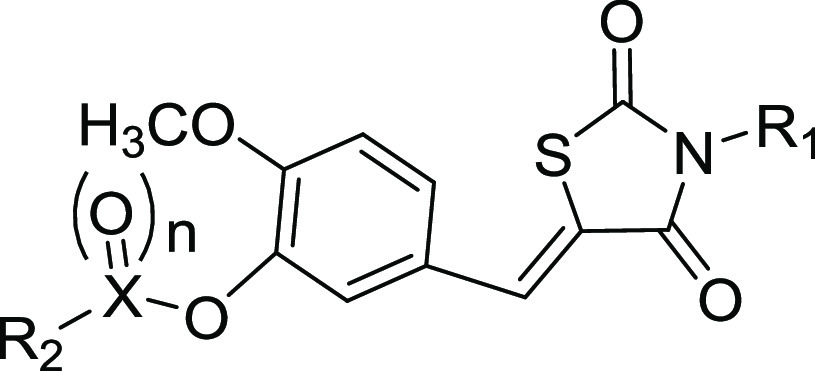

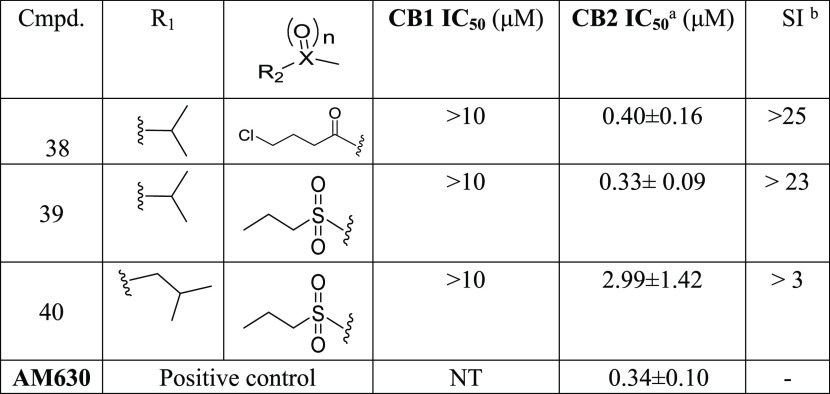
Antagonist Activities of AM630 and Compounds **38**–**40** in CHO Expressing the CB1 or CB2 Receptor Determined by Calcium Mobilization Assays

a IC_50_ values were obtained from three independent calcium mobilization assays with three repetitions and eight gradients for each time.

b SI: selectivity index for the CB2, SI = IC_50_(CB1)/IC_50_ (CB2).

### Evaluation of Function-Based Ligand Screening Protocol

It is a challenging task to develop small molecules with certain biological function using current computational approaches, particularly for those binding to targets with a cellular signaling response. Recently, we proposed the LRIP concept and successfully applied it in constructing drug target-specific scoring functions—IPSFs.^45^ The IPSF has achieved a significantly better performance in drug lead screening, measured by scoring power and ranking power. LRIP has also been successfully applied by us to interpret the immunological response of the antigenic peptides of H2-M3.^46^ We found that the antigenic peptides have similar immunological responses as long as their LRIPs are similar. In 2022, Wang et al. also found that residue–ligand interaction pattern could reflect not only the binding affinity but also the function of a ligand.^47^ Inspired by those successful studies, in this paper, we proposed a novel computational approach that allows us to rationally screen and design agonists that target specific receptors. Considering that CB2 is a promising drug target, which has many indications, and the crystal or cryo-EM structures of active/inactive CB1/CB2 are available, CB1/CB2 is an ideal model system to validate our computational protocol.

The leading hypothesis of our computational protocol is that the agonist function of a ligand is encoded in its LRIP, which can be revealed by using the common interaction patterns of known CB1/CB2 agonists. Note that only the residues for which the LRIP among the CB1 or CB2 agonists has small deviations will be collected to generate the signature of the CB1 or CB2 ligands. In practice, we averaged those ligand–residue interaction energies for the signature residues. We then followed the sample computational protocol to generate IP for a designed molecule and then measured its similarity with the signature using different metrics, including correlation coefficient *R*^2^ and average unsigned error, average signed error, and root-mean-square error. As summarized in Tables S1–S4, 26 designed molecules were predicted to be agonists of CB2 using a criterion of *R*^2^ > 0.7 and Δ*G*_bind_ < −10 kcal/mol. Very encouragingly, 16 of them were confirmed to be CB2 agonists by functional assays. Specifically, for the AM630, **38**, **39**, and **40**, which were confirmed as CB2 antagonists, only compound **38** was incorrectly predicted as a CB2 agonist with a *R*^2^ of 0.77, indicating that our computational protocol is also able to make the right prediction for antagonists.

### Binding Mechanisms of Representative CB2 Agonists and Antagonists

**Figure 5:**
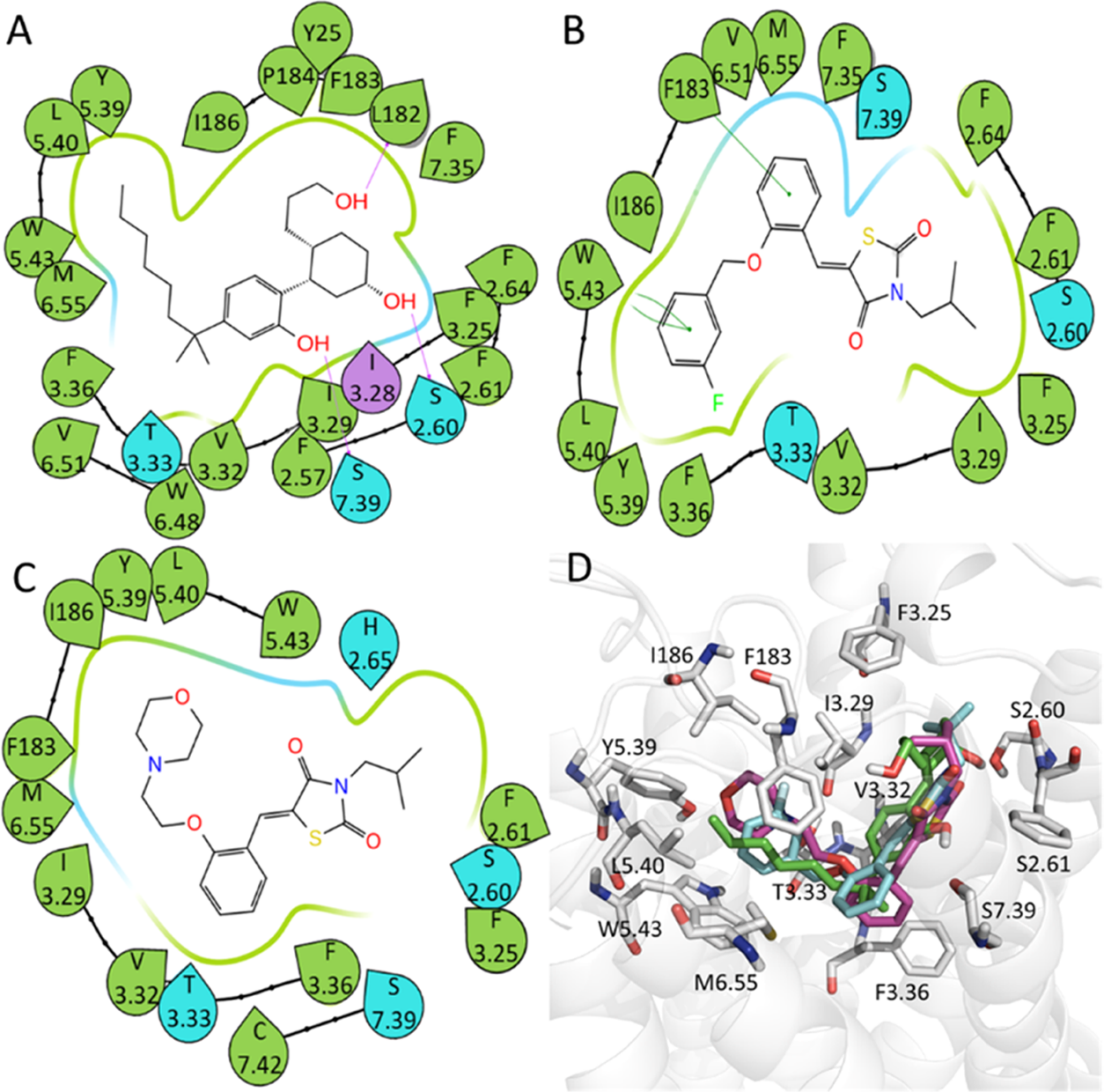
2D ligand–residue interaction diagrams for CB2 agonists generated using the representative MD conformations. (A) CP55940, (B) compound **6**, (C) compound **8**, (D) superposition of CP55940 (green), compound **6** (cyan), and Compound **8** (magenta) and their interacting residues.

According to Tables S1–S4, based on the prediction results and functional assay activities, we selected two potential agonists, **6** and **8**, to look into their binding profiles. As shown in Figure 5D, the CB2 binding orientation of both agonists is similar to the known positive control ligand CP55940. As expected, most of the key residues around these CB2 agonists are consistent with the findings in previous publications.^48−50^ The surrounding residues of compound **6** are depicted in the two-dimensional (2D) ligand–residue interaction diagram generated using the Schrödinger software package (Version 11.2) (Figure 5B). Interestingly, as shown in Figure 7A, the conformations of the key residues in the docking pose only slightly changed after the MD simulation. It is shown that F183 and W5.43 (LRIPs are −2.16 and −1.35 kcal/mol, respectively, Table S5) can both form π–π stacking with the benzene rings in compound **6** to enhance the ligand–receptor interaction. Correspondingly, the importance of these two residues to CB2 binding has also been mentioned in our previous publication.^48^

**Figure 6:**
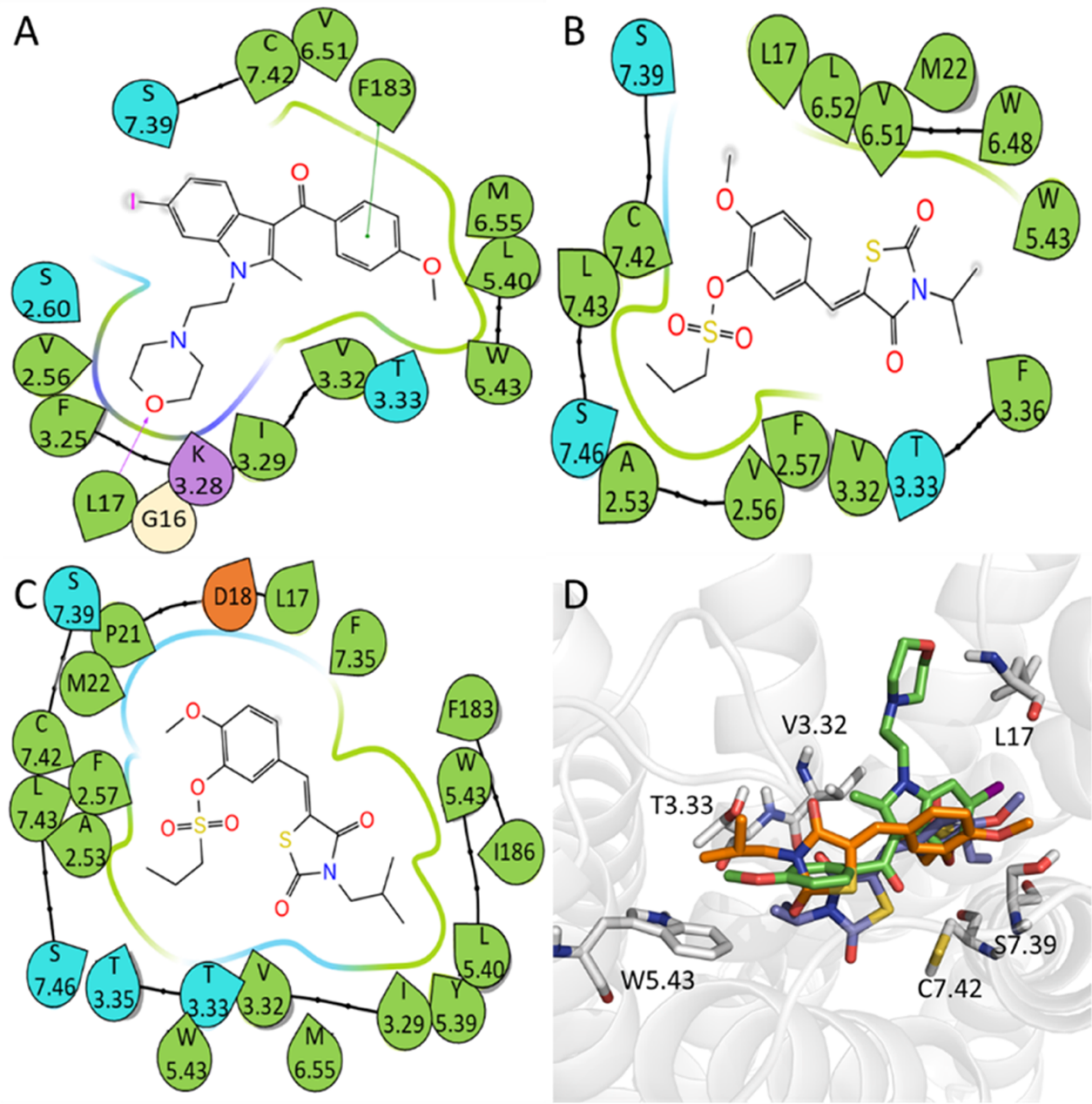
2D ligand–residue interaction diagrams for CB2 antagonists using the representative MD conformations. (A) AM630, (B) compound **39**, (C) compound **40**, and (D) superposition of AM630 (green), compound **39** (purple), and compound **40** (orange) and their interacting residues.

**Figure 7:**
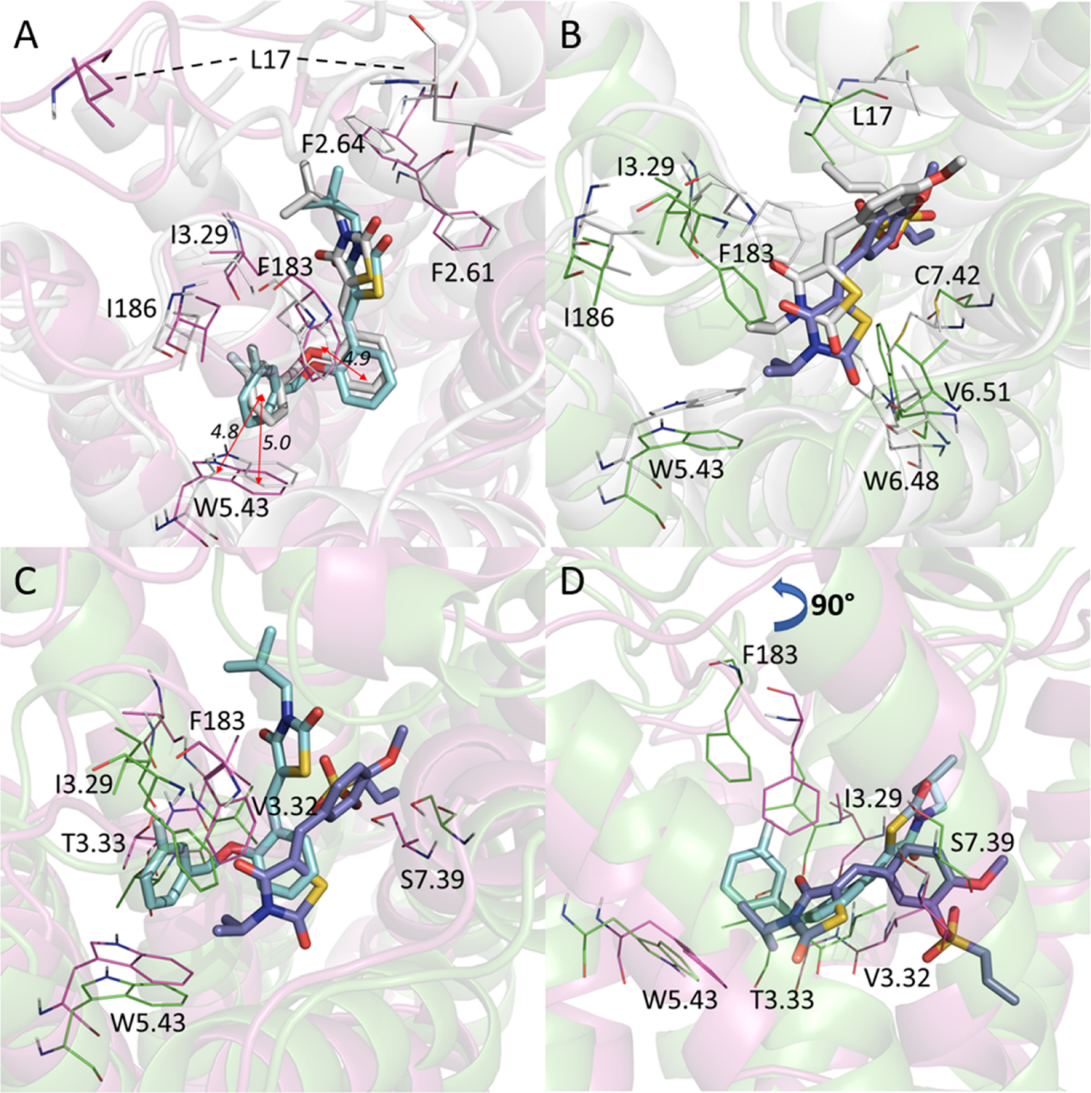
Superposition of the initial docking and representative MD binding poses of the representative CB2 agonist and antagonist. (A) Docking pose (white) vs MD poses for compound **6**. (B) Docking pose (white) vs MD pose for Compound **39**. (C) MD pose of compound **6** (magenta and cyan) vs MD pose of compound **39** (green and purple). (D) Side view of the MD pose of compound **6** (magenta and cyan) vs MD pose of compound **39** (green and purple).

Unlike agonists that share a similar binding pattern (Figure 5D), the antagonists adopt more diverse binding patterns (Figure 6D). However, all the three antagonists, AM630 and compounds **39** and **40**, tend to form strong hydrophobic interactions with residues L17 and C7.42. After MD simulations, the antagonists adopted different binding conformations from the initial ones, such as compound **39** shown in Figure 7B. It is shown that the key residues for compound **39** binding, including L17, W6.48,^51^ V6.51, and C7.42 (LRIPs are −1.24, −1.31, −0.78, −1.52 kcal/mol, respectively, Table S6), adopted different conformations from the initial one. When comparing the binding mode of the representative agonist and antagonist, it is demonstrated that the CB2 antagonist can penetrate deeper in the binding cavity by forming favorable interaction with W6.48 (Figure 7C,7D).

Interestingly, we found that some residues on the intracellular loops of CB2 are likely to play a significant role in CB2 ligand binding. It is shown in Figure 7 that I186 (−0.50 kcal/mol in Table S5) and F183 (−2.16 kcal/mol in Table S5) are key residues for CB2 agonist binding, while L17 (−1.24 kcal/mol in Table S6) and F183 (−1.12 kcal/mol in Table S6) are key residues for CB2 antagonist binding. This information may be useful to develop a modulator without LRIP calculations. Of course, the success rate may not be high.

### Limitations and Future Work

However, this developed protocol is still in development and has some limitations. For example, the numbers of selected representative CB1/CB2 ligands are limited (only AM-4030, AM-11542, THC, WIN-55,212–2, UR-144, SR-147778, AM-251, MK-0364, AM-10257, and AM-630) and maybe not enough to represent the true interaction profiles of agonists/antagonists. To solve this problem, more CB1/CB2 ligands with known activities in functional assays from external databases, such as GPCRdb library (https://gpcrdb.org/), can be used. Another limitation is that not only the established interaction profiles but also the criteria in Figure 2 are system-dependent.

We will continue to improve the prediction accuracy for both the CB1/CB2 targets. Currently, the interaction profiles were established only using several ligands (Table S7). We plan to collect more representative ligands to construct more accurate interaction profiles and establish better criteria to improve the success rate of the function prediction. In the long run, we will construct interaction profiles and selection protocols for other drug targets and disseminate them in a public database. Another method that we will consider is generative chemistry with artificial intelligence.^52,53^ By serving LRIP of some ligands with known function as inputs for training, we can predict the function of more ligands in which we are interested in. If we incorporate a machine learning method into our computational protocol, we can further improve the success rate of functional prediction.

## Conclusions

In this study, we proposed a computational protocol for function-based ligand design and we validated this protocol by taking the CB1/CB2 protein system as an example. Among the 42 tested compounds, 26 were predicted as selective CB2 agonists and 16 were predicted to be CB1/CB2 antagonists or compounds with their function undetermined. Encouragingly, among these 26 agonists, 16 of them were validated as selective CB2 agonists according to their experimental activities. For those predicted as antagonists or functions undetermined, 3 compounds were determined as CB2 antagonists and 10 do not have any CB1/CB2 agonist/antagonist activities in the experiment. In brief, the functions of 29 compounds (16 CB2 selective agonists + 3 CB2 antagonists and 10 undetermined) were successfully predicted, and the success rate is 70%. Thus, the reliability of our function-based agonist design/screen protocol was validated. The analysis of interaction profiles for compound **6** and compound **39** showed that F2.61, I186, and F2.64 are important for CB2 agonists, while L17, W6.48, V6.51, and C7.42 are important for CB2 antagonists. V3.32, T3.33, S7.39, F183, W5.43, and I3.29 are significant for both CB2 agonists and antagonists. Of note, L17, I186, and F183 are loop residues that have not been extensively considered in CB2 ligand design. In conclusion, we have proposed and validated a promising computational protocol for functional design and screen. This approach has a potential to overcome a challenge in current drug design, i.e., rational design of ligands to fulfill certain biological functions.

## Methods and Materials

### Chemicals

#### Materials and Instrumentation

All reagents were procured from commercial suppliers and utilized with no further purification. Thin-layer chromatography (TLC) layers were ordered from Qingdao Marine Chemical Group Co., PR China. NMR spectra (^13^C NMR, ^1^H NMR) were recorded using a Bruker Avance 400 spectrometer within CDCl_3_ solution, where TMS is the internal standard. High-resolution electrospray ionization mass spectrometry (HR-ESI-MS) experiments were processed utilizing a Waters Xevo G2-XS quadrupole time-of-flight (QTOF) mass spectrometer. All compounds are >95% pure as observed by high-performance liquid chromatography (HPLC) analysis.

### General Procedure for the Synthesis of Compounds **1**–**12** and **25**–**27**

#### 2-((2-((3-Isopropyl-2,4-dioxothiazolidin-5-ylidene)methyl)phenoxy)methyl) Benzonitrile (**1**)

##### Preparation of 3-Isopropylthiazolidine-2,4-dione (**1a**)

Thiazolidine-2,4-dione (5.03 g, 43 mmol) was dissolved in ethanol (25 mL), and then, the solution was added to the hot solution of potassium hydroxide (2.54 g, 45 mmol) in ethanol (25 mL). The mixture was stirred and refluxed for 30 min and then cooled to room temperature. The reaction solution was filtered and washed with cold ethanol to obtain potassium 2,4-dioxothiazolidin-3-ide. Then, isopropyl bromide (0.68 g, 5.5 mmol) was added to a solution of 2,4-dioxothiazolidin-3-ide (0.86 g, 5.5 mmol) in DMF (15 mL). The reaction was stirred at reflux for 4 h, and the mixture was poured into water and extracted with dichloromethane (DCM). The organic layer was washed with saturated NaCl aqueous solution and then dried over anhydrous Na_2_SO_4_. The mixture was filtered, and the solvent was evaporated in vacuum. The residue was purified by flash chromatography on silica to obtain 3-isopropylthiazolidine-2,4-dione as a colorless liquid (0.72 g, yield: 82%).

##### Preparation of 2-((2-Formylphenoxy)methyl)benzonitrile (**1b**)

Salicylaldehyde (1.22 g, 10 mmol) was dissolved in *N*,*N*-dimethylformamide (DMF, 25 mL), and then, cesium carbonate (6.52 g, 20 mmol) and α-bromo-*o*-tolunitrile (2.34 g, 12 mmol) were added sequentially. The reaction was stirred at 80 °C for 4 h. After cooling to room temperature, the mixture was poured into water and extracted with dichloromethane (DCM). The organic layer was washed with a saturated NaCl aqueous solution and dried over anhydrous Na_2_SO_4_. The mixture was filtered, and the solvent was evaporated in vacuum. The product was obtained as a white solid (0.72 g, yield: 82%) without further purification.

##### Preparation of Compound **1** (**1**)

A mixture of intermediate **1a** (0.095 g, 0.6 mmol), **1b** (0.12 g, 0.5 mmol), and catalytic amounts of 4-methylpiperidine acetic acid in toluene (10 mL) was refluxed for 4 h. Toluene was removed, and water was added. The mixture was extracted with DCM, and the organic layer was washed with saturated NaCl aqueous solution and dried over anhydrous Na_2_SO_4_ and concentrated under reduced pressure. The residues were purified by flash silica gel chromatography to give compound **1** as a light-yellow solid (0.064 g, yield: 34%). ^1^H NMR (400 MHz, Chloroform-*d*) δ 8.31 (s, 1H), 7.73 (d, *J* = 7.7 Hz, 1H), 7.71–7.64 (m, 2H), 7.52–7.36 (m, 3H), 7.10 (t, *J* = 7.6 Hz, 1H), 7.02 (d, *J* = 8.3 Hz, 1H), 5.36 (s, 2H), 4.67 (p, *J* = 6.9 Hz, 1H), 1.49 (s, 3H), 1.47 (s, 3H). ^13^C NMR (101 MHz, CDCl_3_) δ 19.30, 19.30, 47.19, 68.01, 111.03, 112.66, 116.93, 121.88, 122.12, 123.21, 128.10, 128.28, 128.68, 129.19, 132.02, 132.99, 133.39, 139.81, 156.93, 166.32, 167.96. HRMS (ESI, *m*/*z*) calculated for [C_21_H_18_N_2_O_3_S + H]^+^: 379.1116; found: 379.1109.

#### 2-((2-((3-Isobutyl-2,4-dioxothiazolidin-5-ylidene)methyl)phenoxy)methyl) Benzonitrile h(**2**)

White solid, yield: 25%. ^1^H NMR (400 MHz, CDCl_3_) δ 8.35 (s, 1H), 7.74–7.71 (m, 1H), 7.70–7.62 (m, 2H), 7.53–7.38 (m, 3H), 7.16–7.07 (m, 1H), 7.03 (dd, *J* = 8.4, 1.0 Hz, 1H), 5.37 (s, 2H), 3.57 (d, *J* = 7.5 Hz, 2H), 2.20–2.10 (m, 1H), 0.95 (s, 3H), 0.93 (s, 3H). ^13^C NMR (101 MHz, CDCl_3_) δ 20.00, 20.00, 27.24, 49.03, 68.05, 111.04, 112.71, 116.91, 121.90, 122.00, 123.11, 128.27, 128.50, 128.68, 129.21, 132.15, 132.99, 133.37, 139.79, 157.00, 166.60, 168.37. HRMS (ESI, *m*/*z*) calculated for [C_22_H_21_N_2_O_3_S + H]^+^: 393.1267; found: 393.1259.

#### 2-((2-((2,4-Dioxo-3-propylthiazolidin-5-ylidene)methyl)phenoxy)methyl) Benzo Nitrile (**3**)

Light-yellow solid, yield: 42%. ^1^H NMR (400 MHz, Chloroform-*d*) δ 8.35 (s, 1H), 7.73 (d, *J* = 7.2 Hz, 1H), 7.71–7.64 (m, 2H), 7.51–7.38 (m, 3H), 7.11 (t, *J* = 7.6 Hz, 1H), 7.03 (d, *J* = 8.3 Hz, 1H), 5.37 (s, 2H), 3.76–3.68 (m, 2H), 1.75–1.66 (m, 2H), 0.95 (t, *J* = 7.4 Hz, 3H). ^13^C NMR (101 MHz, CDCl_3_) δ 11.19, 21.16, 43.50, 68.06, 111.05, 112.72, 116.90, 121.90, 122.13, 123.12, 128.27, 128.49, 128.68, 129.19,132.15, 132.99, 133.36, 139.79, 157.00, 166.40, 168.21. HRMS (ESI, *m*/*z*) calculated for [C_21_H_18_N_2_O_3_S + H]^+^: 379.1111; found: 379.1109.

#### 2-((2-((3-Butyl-2,4-dioxothiazolidin-5-ylidene)methyl)phenoxy)methyl)benzo Nitrile (**4**)

Light-yellow solid, yield: 51%. ^1^H NMR (400 MHz, Chloroform-*d*) δ 8.40 (s, 1H), 7.78 (d, *J* = 7.6 Hz, 1H), 7.73–7.69 (m, 2H), 7.52–7.29 (m, 2H), 7.56–744 (m, 3H), 7.18–7.14 (m, 1H), 7.08–7.06 (m, 1H), 5.42 (s, 2H), 3.82–3.78 (m, 2H), 1.74–1.66 (m, 2H), 1.47–1.37 (m, 2H), 1.02–0.98 (m, 3H). ^13^C NMR (101 MHz, CDCl_3_) δ: 13.68, 20.06, 29.89, 41.84, 68.07, 111.07, 112.72, 116.97, 121.93, 122.16, 123.13, 128.32, 128.52, 128.74, 129.23, 132.21, 133.04, 133.43, 139.82, 157.03, 166.44, 168.26. HRMS (ESI, *m*/*z*) calculated for [C_22_H_20_N_2_O_3_S + H]^+^: 393.1267; found: 393.1259.

#### 2-((2-((3-Cyclopentyl-2,4-dioxothiazolidin-5-ylidene)methyl)phenoxy)methyl) Benzonitrile (**5**)

Light-yellow solid, yield: 43%. ^1^H NMR (400 MHz, Chloroform-*d*) δ 8.31 (s, 1H), 7.75–7.64 (m, 3H), 7.53–7.35 (m, 3H), 7.10 (t, *J* = 7.6 Hz, 1H), 7.02 (d, *J* = 8.3 Hz, 1H), 5.36 (s, 2H), 4.81–4.72 (m, 1H), 2.16–2.03 (m, 2H), 2.00–1.86 (m, 4H), 1.68–1.58 (m, 2H). ^13^C NMR (101 MHz, CDCl_3_) δ: 25.26, 25.26, 28.69, 28.69, 54.67, 68.02, 111.04, 112.68, 116.91, 121.88, 122.07, 123.25, 128.14, 128.28, 128.67, 129.20, 132.02, 132.98, 133.38, 139.82, 156.94, 166.43, 168.07. HRMS (ESI, *m*/*z*) calculated for [C_23_H_20_N_2_O_3_S + H]^+^: 405.1267; found: 405.1271.

#### 5-(2-((2-Fluorobenzyl)oxy)benzylidene)-3-isopropylthiazolidine-2,4-dione (**6**)

White solid, yield: 38.5%. ^1^H NMR (400 MHz, Chloroform-*d*) δ 8.30 (s, 1H), 7.51–7.45 (m, 2H), 7.40–7.29 (m, 2H), 7.18 (td, *J* = 7.6, 1.2 Hz, 1H), 7.13–7.00 (m, 3H), 5.24 (s, 2H), 4.71–4.64 (m, 1H), 1.49 (s, 3H), 1.47 (s, 3H). ^13^C NMR (101 MHz, CDCl_3_) δ 19.29, 47.12, 64.28, 64.32, 112.66, 115.34, 115.55, 121.39, 121.77, 123.17, 123.35, 123.49, 124.47, 124.51, 128.54, 129.13, 129.45, 129.49, 129.89, 129.97, 131.95, 157.38, 159.13, 161.58, 166.35, 168.10. HRMS (ESI, *m*/*z*) calculated for [C_20_H_18_FNO_3_S + H]^+^: 372.1064; found: 372.1075.

#### 5-(2-((3-Bromobenzyl)oxy)benzylidene)-3-isopropylthiazolidine-2,4-dione(**7**)

White solid, yield: 32%. ^1^H NMR (400 MHz, Chloroform-*d*) δ 8.29 (s, 1H), 7.55 (s, 1H), 7.46 (d, *J* = 7.8 Hz, 2H), 7.35 (td, *J* = 7.8, 1.8 Hz, 2H), 7.27–7.24 (m, 1H), 7.06 (t, *J* = 7.6 Hz, 1H), 6.92 (d, *J* = 8.3 Hz, 1H), 5.15 (s, 2H), 4.71–4.64 (m, 1H), 1.49 (s, 3H), 1.47 (s, 3H). ^13^C NMR (101 MHz, CDCl_3_) δ 19.30, 29.71, 47.17, 69.69, 112.75, 121.47, 121.95, 122.79, 123.19, 125.65, 128.41, 129.20, 130.11, 130.40, 131.27, 131.90, 138.63, 157.27, 166.33, 168.06. HRMS (ESI, *m*/*z*) calculated for [C_20_H_18_BrNO_3_S + H]^+^: 432.0264; found: 432.0245.

#### 3-Isopropyl-5-(2-(2-morpholinoethoxy)benzylidene) Thiazolidine-2,4-dione (**8**)

White solid, yield: 41%. ^1^H NMR (400 MHz, Chloroform-*d*) δ 8.18 (s, 1H), 7.37 (dd, *J* = 7.7, 1.6 Hz, 1H), 7.33–7.28 (m, 1H), 6.98 (td, *J* = 7.6, 1.1 Hz, 1H), 6.88 (dd, *J* = 8.4, 1.1 Hz, 1H), 4.64–4.57 (m, 1H), 4.12 (t, *J* = 5.9 Hz, 2H), 3.69–3.64 (m, 4H), 2.81 (t, *J* = 5.8 Hz, 2H), 2.57–2.51 (m, 4H), 1.42 (s, 3H), 1.40 (s, 3H). ^13^C NMR (101 MHz, CDCl_3_) δ 19.29, 19.29, 47.14, 54.11, 54.11, 57.40, 66.79, 66.96, 66.96, 112.36, 121.16, 121.52, 123.00, 128.53, 128.98, 131.92, 157.75, 166.36, 168.06. HRMS (ESI, *m*/*z*) calculated for [C_19_H_24_N_2_O_4_S + H]^+^: 377.1530; found: 377.1536.

#### 5-(2-((3,5-Dimethoxybenzyl)oxy)benzylidene)-3-isopropylthiazolidine-2,4-dione (**9**)

White solid, yield: 45%. ^1^H NMR (400 MHz, CDCl_3_) δ 8.35 (s, 1H), 7.45 (d, *J* = 7.7 Hz, 1H), 7.36–7.32 (m, 1H), 7.06–7.02 (m, 1H), 6.95 (d, *J* = 7.7 Hz, 1H), 6.58 (s, 2H), 6.41 (s, 1H), 5.13 (s, 2H), 4.71–4.64 (m, 1H), 3.81 (s, 6H), 1.49 (s, 3H), 1.47 (s, 3H). ^13^C NMR (101 MHz, CDCl_3_) δ 19.29, 19.29, 47.11, 55.42, 55.42, 69.94, 99.68, 100.03, 104.68, 104.68, 112.93, 121.24, 121.85, 123.57, 125.12, 128.65, 129.07, 131.91, 140.10, 156.51, 161.11, 167.73. HRMS (ESI, *m*/*z*) calculated for [C_22_H_23_NO_5_S + H]^+^: 414.1370; found: 414.1381.

#### 5-(2-((2-Fluorobenzyl)oxy)benzylidene)-3-isobutylthiazolidine-2,4-dione (**10**)

White solid, yield: 31%. ^1^H NMR (400 MHz, Chloroform-*d*) δ 8.27 (s, 1H), 7.45–7.39 (m, 2H), 7.33–7.22 (m, 2H), 7.11 (td, *J* = 7.5, 1.2 Hz, 1H), 7.06–6.94 (m, 3H), 5.18 (s, 2H), 3.50 (d, *J* = 7.4 Hz, 2H), 2.13–2.02 (m, 1H), 0.88 (s, 3H), 0.86 (s, 3H). ^13^C NMR (101 MHz, CDCl_3_) δ 20.00, 27.24, 49.00, 64.33, 64.37, 112.73, 115.34, 115.55, 121.43, 121.68, 123.09, 123.34, 123.48, 124.47, 124.51, 128.94, 129.16, 129.44, 129.47, 129.90, 129.98, 132.09, 157.46, 159.13, 161.59, 166.63, 168.51. HRMS (ESI, *m*/*z*) calculated for [C_22_H_22_FNO_4_S + H]^+^:386.1221; found: 386.1230.

#### 5-(2-((3,5-Dimethoxybenzyl)oxy)benzylidene)-3-isobutylthiazolidine-2,4-dione (**11**)

White solid, yield: 39%. ^1^H NMR (400 MHz, CDCl_3_) δ 8.39 (s, 1H), 7.47 (dd, *J* = 7.8, 1.6 Hz, 1H), 7.35 (td, *J* = 7.8, 1.6 Hz, 1H), 7.05 (td, *J* = 7.5, 1.0 Hz, 1H), 6.96 (dd, *J* = 8.4, 1.1 Hz, 1H), 6.58 (d, *J* = 2.3 Hz, 2H), 6.41 (t, *J* = 2.3 Hz, 1H), 5.13 (s, 2H), 3.80 (s, 6H), 3.57 (d, *J* = 7.5 Hz, 2H), 2.14 (m, 1H), 0.94 (s, 3H), 0.93 (s, 3H). ^13^C NMR (101 MHz, CDCl_3_) δ 19.99, 19.99, 27.24, 48.97, 55.41, 55.41, 70.44, 100.05, 104.67, 104.67, 112.95, 121.25, 121.61, 123.04, 129.04, 129.10, 132.04, 138.77, 157.64, 161.12, 161.12, 166.60, 168.54. HRMS (ESI, *m*/*z*) calculated for [C_23_H_25_NO_5_S + H]^+^: 428.1526; found: 428.1537.

#### 3-Cyclopentyl-5-(2-((2-fluorobenzyl)oxy)benzylidene)thiazolidine-2,4-dione (**12**)

White solid, yield: 43%. ^1^H NMR (400 MHz, Chloroform-*d*) δ 8.23 (s, 1H), 7.44–7.37 (m, 2H), 7.32–7.23 (m, 2H), 7.11 (td, *J* = 7.5, 1.2 Hz, 1H), 7.06–6.92 (m, 3H), 5.17 (s, 2H), 4.74–4.65 (m, 1H), 2.08–1.98 (m, 2H), 1.91–1.78 (m, 4H), 1.60–1.52 (m, 2H). ^13^C NMR (101 MHz, CDCl_3_) δ 25.28, 28.69, 54.61, 64.27, 64.32, 112.66, 115.35, 115.56, 121.39, 121.71, 123.20, 123.35, 123.49, 124.48, 124.52, 128.60, 129.14, 129.47, 129.51, 129.90, 129.98, 131.96, 157.39, 159.14, 161.59, 166.47, 168.24. HRMS (ESI, *m*/*z*) calculated for [C_22_H_20_FNO_3_S + H]^+^: 398.1221; found: 398.1213.

#### 2-((3-((3-Isopropyl-2,4-dioxothiazolidin-5-ylidene)methyl)phenoxy)methyl) Benzonitrile (**25**)

White solid, yield: 38%. ^1^H NMR (400 MHz, CDCl_3_) δ 7.81 (s, 1H), 7.74 (d, *J* = 7.8 Hz, 1H), 7.69–7.63 (m, 2H), 7.51–7.37 (m, 2H), 7.15 (d, *J* = 7.7 Hz, 1H), 7.10–7.01 (m, 2H), 5.30 (s, 2H), 4.71–4,63 (m, 1H), 1.49 (s, 3H), 1.47 (s, 3H). ^13^C NMR (101 MHz, CDCl_3_) δ 19.28, 19.28, 47.35, 67.86, 111.33, 116.35, 116.93, 122.37, 123.47, 128.53, 128.69, 130.44, 132.84, 133.07, 133.17, 133.17, 134.97, 140.00, 158.59, 166.24, 167.19. HRMS (ESI, *m*/*z*) calculated for [C_21_H_18_N_2_O_3_S + H]^+^: 379.1111; found: 379.1109.

#### 2-((3-((3-Isobutyl-2,4-dioxothiazolidin-5-ylidene)methyl)phenoxy)methyl) Benzonitrile (**26**)

White solid, yield: 37%. ^1^H NMR (400 MHz, CDCl_3_) δ 7.89 (s, 1H), 7.78 (d, *J* = 7.7 Hz, 1H), 7.71 (dd, *J* = 6.5, 1.5 Hz, 2H), 7.56–7.42 (m, 2H), 7.24–7.09 (m, 3H), 5.34 (s, 2H), 3.62 (d, *J* = 7.4 Hz, 2H), 2.21–2.13 (m, 1H), 0.99 (s, 3H), 0.97 (s, 3H). HRMS (ESI, *m*/*z*) calculated for [C_22_H_21_N_2_O_3_S + H]^+^: 393.1267; found: 393.1259.

#### 2-((3-((3-Cyclopentyl-2,4-dioxothiazolidin-5-ylidene)methyl)phenoxy)methyl) Benzonitrile (**27**)

Light-yellow solid, yield: 44%. ^1^H NMR (400 MHz, Chloroform-*d*) δ 7.81 (s, 1H), 7.77–7.70 (m, 1H), 7.69–7.61 (m, 2H), 7.52–7.37 (m, 2H), 7.15 (dd, *J* = 7.3, 1.6 Hz, 1H), 7.12–7.01 (m, 2H), 5.30 (s, 2H), 4.81–4.73 (m, 1H), 2.16–2.02 (m, 2H), 1.99–1.88 (m, 4H), 1.68–1.58 (m, 2H), 0.90 (d, *J* = 6.8 Hz, 1H). ^13^C NMR (101 MHz, CDCl_3_) δ 25.26, 25.26, 28.71, 28.71, 54.81, 67.87, 111.34, 116.36, 116.94, 116.96, 122.31, 123.47, 128.52, 128.68, 130.43, 132.85, 133.06, 133.16, 134.99, 140.01, 158.60, 166.35, 167.63. HRMS (ESI, *m*/*z*) calculated for [C_23_H_20_N_2_O_3_S + H]^+^: 405.1267; found: 405.1271.

### General Procedure for the Synthesis of Compounds **13**–**24**, **28**–**37**

#### 2-((3-Isobutyl-2,4-dioxothiazolidin-5-ylidene)methyl)phenyl 2-Fluorobenzoate (**13**)

##### Preparation of 3-Isopropylthiazolidine-2,4-dione (**13a**)

A mixture of 3-isobutylthiazolidine-2,4-dione (0.83 g, 4.8 mmol), salicylaldehyde (0.49 g, 4.0 mmol), and catalytic amounts of 4-methylpiperidium acetate in toluene (15 mL) was refluxed for 4 h. The reaction solution was concentrated under reduced pressure, and the residue was dissolved in DCM and further washed with water. The organic layer was dried over anhydrous Na_2_SO_4_. The mixture was filtered, and the solvent was evaporated in vacuum. The residue was purified by recrystallization to give 3-isopropylthiazolidine-2,4-dione (**17a**) as a white solid (0.57 g, yield: 51%).

##### Preparation of Compound **13**

The mixture of 3-isopropylthiazolidine-2,4-dione (0.28 g, 1.0 mmol), trimethylamine (1.2 mmol), and catalytic amounts of 4-dimethylaminopyridine (DMAP) in DCM (15 mL) was treated dropwise under stirring with 2-fluorobenzoyl chloride (0.095 g, 0.6 mmol) in DCM. The reaction was stirred at room temperature overnight, and then, the mixture was washed with water and brine. The organic layer was dried over anhydrous Na_2_SO_4_. The mixture was filtered, and the solvent was evaporated in vacuum. The residue was purified by recrystallization to give compound **13** as a white solid (0.084 g, yield: 42%).

^1^H NMR (400 MHz, Chloroform-*d*) δ 8.16–8.06 (m, 2H), 7.67–7.61 (m, 2H), 7.51 (td, *J* = 7.8, 1.6 Hz, 1H), 7.44–7.28 (m, 3H), 7.24 (dd, *J* = 8.4, 1.2 Hz, 1H), 3.55 (d, *J* = 7.5 Hz, 2H), 2.17–2.07 (m, 1H), 0.93 (s, 3H), 0.91 (s, 3H). ^13^C NMR (101 MHz, CDCl_3_) δ 19.99, 27.21, 49.16, 117.31, 117.35, 117.41, 117.57, 123.41, 123.98,124.40, 124.44, 126.48, 126.70, 126.92, 128.65, 131.49, 132.63, 135.70, 135.79, 149.99, 161.12, 162.45, 162.50, 163.72, 166.23, 167.92. HRMS (ESI, *m*/*z*) calculated for [C_21_H_18_FNO_4_S + H]^+^:400.1013; found: 400.0994.

#### 2-((3-Isobutyl-2,4-dioxothiazolidin-5-ylidene) methyl) Phenyl Octanoate (**14**)

White solid, yield: 48%. ^1^H NMR (400 MHz, Chloroform-*d*) δ 7.94 (s, 1H), 7.56 (dd, *J* = 7.8, 1.6 Hz, 1H), 7.46 (td, *J* = 7.8, 1.6 Hz, 1H), 7.35 (td, *J* = 7.6, 1.3 Hz, 1H), 7.18 (dd, *J* = 8.1, 1.3 Hz, 1H), 3.57 (d, *J* = 7.5 Hz, 2H), 2.65 (t, *J* = 7.5 Hz, 2H), 2.19–2.09 (m, 1H), 1.83–1.75 (m, 2H), 1.47–1.30 (m, 8H), 0.95 (s, 3H), 0.94 (s, 3H), 0.90 (t, *J* = 6.7 Hz, 3H). ^13^C NMR (101 MHz, CDCl_3_) δ 14.08, 20.00, 20.00, 22.62, 24.95, 27.25, 28.92, 29.09, 31.63, 34.30, 49.16, 123.35, 123.74, 126.38, 126.41, 126.98, 128.60, 131.46, 150.13, 166.26, 167.97, 171.97. HRMS (ESI, *m*/*z*) calculated for [C_22_H_29_NO_4_S + H]^+^: 404.1890; found: 404.1886.

#### 2-((3-Isobutyl-2,4-dioxothiazolidin-5-ylidene)methyl)phenyl Morpholine-4-carboxylate (**15**)

White solid, yield: 38%. ^1^H NMR (400 MHz, Chloroform-*d*) δ 7.98 (s, 1H), 7.55 (dd, *J* = 7.8, 1.6 Hz, 1H), 7.46 (td, *J* = 7.8, 1.6 Hz, 1H), 7.34 (td, *J* = 7.7, 1.3 Hz, 1H), 7.24 (dd, *J* = 8.1, 1.2 Hz, 1H), 3.81–3.76 (m, 6H), 3.58 (d, *J* = 7.4 Hz, 4H), 2.19–2.09 (m, 1H), 0.96 (s, 3H), 0.94 (s, 3H). ^13^C NMR (101 MHz, CDCl_3_) δ 19.99, 19.99, 27.24, 27.24, 44.41, 45.16, 49.16, 66.57, 123.50, 123.55, 126.21, 126.51, 126.84, 128.43, 131.52, 150.62, 152.92, 166.37, 167.94. HRMS (ESI, *m*/*z*) calculated for [C_19_H_22_N_2_O_5_S + H]^+^: 391.1322; found: 391.1323.

#### 2-((3-Isobutyl-2,4-dioxothiazolidin-5-ylidene) methyl) Phenyl 3-Chloro-2,2-dimethylpropanoate (**16**)

White solid, yield: 45%. ^1^H NMR (400 MHz, Chloroform-*d*) δ 7.97 (s, 1H), 7.57 (dd, *J* = 7.8, 1.6 Hz, 1H), 7.47 (td, *J* = 7.8, 1.7 Hz, 1H), 7.37 (td, *J* = 7.7, 1.3 Hz, 1H), 7.19 (dd, *J* = 8.1, 1.3 Hz, 1H), 3.78 (s, 2H), 3.57 (d, *J* = 7.5 Hz, 2H), 2.17–2.08 (m, 1H), 1.52 (s, 6H), 0.94 (s, 3H), 0.93 (s, 3H). ^13^C NMR (101 MHz, CDCl_3_) δ 19.97, 19.97, 23.41, 23.41, 27.22, 45.52, 49.16, 51.65, 123.24, 123.90, 126.53, 126.58, 126.70, 128.44, 131.53, 150.13, 166.21, 167.89, 173.31. HRMS (ESI, *m*/*z*) calculated for [C_19_H_22_ClNO_4_S + H]^+^: 396.1031; found: 396.1033.

#### 2-((3-Isopropyl-2,4-dioxothiazolidin-5-ylidene)methyl)phenyl Morpholine-4-carboxylate (**17**)

White solid, yield: 34%. ^1^H NMR (400 MHz, Chloroform-*d*) δ 7.93 (s, 1H), 7.53 (dd, *J* = 7.8, 1.6 Hz, 1H), 7.45 (td, *J* = 7.7, 1.6 Hz, 1H), 7.33 (td, *J* = 7.7, 1.3 Hz, 1H), 7.23 (dd, *J* = 8.1, 1.2 Hz, 1H), 4.72–4.63 (m, 1H), 3.84–3.74 (m, 6H), 3.62–3.55 (m, 2H), 1.49 (s, 3H), 1.47 (s, 3H). ^13^C NMR (101 MHz, CDCl_3_) δ 19.28, 19.28, 44.41, 45.17, 47.38, 66.55, 66.59, 123.51, 123.63, 126.20, 126.43, 126.65, 128.45, 131.39, 150.57, 152.96, 166.07, 167.50. HRMS (ESI, *m*/*z*) calculated for [C_18_H_20_N_2_O_5_S + H]^+^: 377.1166; found: 377.1182.

#### 2-((3-Isopropyl-2,4-dioxothiazolidin-5-ylidene) methyl) Phenyl 2-Fluoro Benzoate (**18**)

Light-yellow solid, yield: 57%. ^1^H NMR (400 MHz, Chloroform-*d*) δ 8.12 (t, *J* = 7.5 Hz, 1H), 8.05 (s, 1H), 7.66–7.60 (m, 2H), 7.50 (t, *J* = 7.8 Hz, 1H), 7.41–7.26 (m, 4H), 4.67–4.59 (m, 1H), 1.46 (s, 3H), 1.45 (s, 3H). ^13^C NMR (101 MHz, CDCl_3_) δ 19.25, 47.37, 117.33, 117.44, 117.55, 123.35, 124.16, 124.38, 124.42, 126.48, 126.64, 126.67, 128.66, 131.34, 132.63, 135.66, 135.75, 149.95, 161.14, 162.44, 162.48, 163.74, 165.91, 167.45. HRMS (ESI, *m*/*z*) calculated for [C_20_H_16_FNO_4_S + H]^+^: 386.0857; found: 386.0853.

#### 2-((3-Isopropyl-2,4-dioxothiazolidin-5-ylidene) Methyl) Phenyl (3r,5r,7r)-Adamantane-1-carboxylate (**19**)

White solid, yield: 76%. ^1^H NMR (400 MHz, Chloroform-*d*) δ 7.88 (s, 1H), 7.54 (dd, *J* = 7.8, 1.6 Hz, 1H), 7.43 (td, *J* = 7.8, 1.6 Hz, 1H), 7.33 (td, *J* = 7.7, 1.2 Hz, 1H), 7.11 (dd, *J* = 8.0, 1.2 Hz, 1H), 4.70–4.63 (m, 1H), 2.13 (s, 9H), 1.80 (s, 6H), 1.49 (s, 3H), 1.47 (s, 3H). ^13^C NMR (101 MHz, CDCl_3_) δ 19.28, 19.28, 27.89, 27.89, 27.89, 36.39, 36.39, 36.39, 38.77, 38.77, 38.77, 41.40, 47.37, 123.27, 123.59, 126.29, 126.36, 126.64, 128.43, 131.32, 150.58, 165.95, 167.51, 175.84. HRMS (ESI, *m*/*z*) calculated for [C_24_H_27_NO_4_S + H]^+^: 426.1734; found: 426.1749.

#### 2-((3-Isopropyl-2,4-dioxothiazolidin-5-ylidene) Methyl) Phenyl Octanoate (**20**)

White solid, yield: 38%. ^1^H NMR (400 MHz, Chloroform-*d*) δ 7.89 (s, 1H), 7.54 (dd, *J* = 7.8, 1.6 Hz, 1H), 7.45 (td, *J* = 7.8, 1.7 Hz, 1H), 7.34 (td, *J* = 7.6, 1.2 Hz, 1H), 7.17 (dd, *J* = 8.2, 1.2 Hz, 1H), 4.70–4.63 (m, 1H), 2.65 (t, *J* = 7.5 Hz, 2H), 1.83–1.76 (m, 2H), 1.49 (s, 3H), 1.47 (s, 3H), 1.40–1.29 (m, 6H), 0.90 (t, *J* = 6.6 Hz, 3H). ^13^C NMR (101 MHz, CDCl_3_) δ 14.08, 19.27, 19.27, 22.62, 24.96, 28.93, 29.10, 31.63, 34.30, 47.36, 123.30, 123.86, 126.39, 126.51, 126.56, 128.60, 131.33, 150.08, 165.96, 167.53, 171.99. HRMS (ESI, *m*/*z*) calculated for [C_21_H_27_NO_4_S + H]^+^: 390.1734; found: 390.1741.

#### 2-((3-Isopropyl-2,4-dioxothiazolidin-5-ylidene)methyl)phenyl 3-Chloro-2,2-dimethylpropanoate (**21**)

White solid, yield: 49%. ^1^H NMR (400 MHz, Chloroform-*d*) δ 7.92 (s, 1H), 7.55 (dd, *J* = 7.8, 1.6 Hz, 1H), 7.46 (td, *J* = 7.7, 1.7 Hz, 1H), 7.36 (td, *J* = 7.6, 1.3 Hz, 1H), 7.18 (dd, *J* = 8.1, 1.3 Hz, 1H), 4.69–4.62 (m, 1H), 3.79 (s, 2H), 1.52 (s, 6H), 1.48 (s, 3H), 1.46 (s, 3H). ^13^C NMR (101 MHz, CDCl_3_) δ 19.26, 19.26, 23.41, 23.41, 45.51, 47.40, 51.67, 123.19, 124.02, 126.12, 126.63, 126.68, 128.47, 131.40, 150.09, 165.89, 167.44, 173.33. HRMS (ESI, *m*/*z*) calculated for [C_18_H_20_ClNO_4_S + H]^+^: 382.0874; found: 382.0860.

#### 2-((3-Isopropyl-2,4-dioxothiazolidin-5-ylidene)methyl)phenyl Cyclopropane Carboxylate (**22**)

White solid, yield: 39%. ^1^H NMR (400 MHz, Chloroform-*d*) δ 7.85 (s, 1H), 7.46 (dd, *J* = 7.8, 1.6 Hz, 1H), 7.36 (td, *J* = 7.7, 1.6 Hz, 1H), 7.26 (td, *J* = 7.6, 1.3 Hz, 1H), 7.12 (dd, *J* = 8.1, 1.3 Hz, 1H), 4.65–4.55 (m, 1H), 1.90–1.83 (m, 1H), 1.42 (s, 3H), 1.40 (s, 3H), 1.18–1.13 (m, 2H), 1.06–1.00 (m, 2H). ^13^C NMR (101 MHz, CDCl_3_) δ 9.66, 9.66, 13.01, 19.28, 19.28, 47.37, 123.29, 123.76, 126.33, 126.46, 126.63, 128.58, 131.32, 150.10, 166.04, 167.55, 173.11. HRMS (ESI, *m*/*z*) calculated for [C_17_H_17_NO_4_S + H]^+^: 332.0951; found: 332.0944.

#### 2-((3-Isopropyl-2,4-dioxothiazolidin-5-ylidene)methyl)phenyl Cycloheptane Carboxylate (**23**)

Light-yellow solid, yield: 54%. ^1^H NMR (400 MHz, Chloroform-*d*) δ 7.90 (s, 1H), 7.54 (dd, *J* = 7.8, 1.6 Hz, 1H), 7.44 (td, *J* = 7.7, 1.6 Hz, 1H), 7.33 (td, *J* = 7.6, 1.2 Hz, 1H), 7.14 (dd, *J* = 8.1, 1.3 Hz, 1H), 4.72–4.61 (m, 1H), 2.88–2.81 (m, 1H), 2.19–2.11 (m, 2H), 1.92–1.79 (m, 4H), 1.66–1.57 (m, 6H), 1.49 (s, 3H), 1.47 (s, 3H). ^13^C NMR (101 MHz, CDCl_3_) δ 19.27, 19.27, 26.26, 26.26, 28.33, 28.33, 30.79, 30.79, 45.02, 47.36, 123.23, 123.76, 126.31, 126.55, 126.60, 128.52, 131.31, 150.29, 165.96, 167.53, 175.10. HRMS (ESI, *m*/*z*) calculated for [C_21_H_25_NO_4_S + H]^+^: 388.1577; found: 388.1566.

#### 2-((3-Isopropyl-2,4-dioxothiazolidin-5-ylidene)methyl)phenyl 6-Chloronico Tinate (**24**)

White solid, yield: 62%. ^1^H NMR (400 MHz, Chloroform-*d*) δ 9.20 (d, *J* = 2.4 Hz, 1H), 8.42 (dd, *J* = 8.3, 2.5 Hz, 1H), 7.89 (s, 1H), 7.62 (dd, *J* = 7.7, 1.7 Hz, 1H), 7.54–7.49 (m, 2H), 7.43 (td, *J* = 7.6, 1.3 Hz, 1H), 7.31 (dd, *J* = 8.1, 1.3 Hz, 1H), 4.68–4.59 (m, 1H), 1.46 (s, 3H), 1.44 (s, 3H). ^13^C NMR (101 MHz, CDCl_3_) δ 19.24, 19.24, 47.47, 123.17, 123.79, 124.68, 124.70, 125.74, 126.66, 127.10, 128.81, 131.49, 140.09, 149.48, 151.81, 156.92, 162.77, 165.79, 167.23. HRMS (ESI, *m*/*z*) calculated for [C_19_H_15_ClN_2_O_4_S + H]^+^: 403.0514; found: 403.0526.

#### 5-((3-Isopropyl-2,4-dioxothiazolidin-5-ylidene)methyl)-2-methoxyphenyl 3-Bromobenzoate (**28**)

White solid, yield: 45%. ^1^H NMR (400 MHz, Chloroform-*d*) δ 8.36 (s, 1H), 8.15 (d, *J* = 7.8 Hz, 1H), 7.82–7.73 (m, 2H), 7.46–7.35 (m, 2H), 7.31 (d, *J* = 2.3 Hz, 1H), 7.10 (d, *J* = 8.6 Hz, 1H), 4.71- 4.64 (m, 1H), 3.88 (s, 3H), 1.49 (s, 3H), 1.47 (s, 3H). ^13^C NMR (101 MHz, CDCl_3_) δ 19.28, 19.28, 47.30, 56.16, 112.81, 120.06, 122.70, 124.56, 126.53, 128.96, 129.90, 130.19, 130.91, 132.08, 133.32, 136.71, 140.12, 152.95, 163.13, 166.37, 167.49. HRMS (ESI, *m*/*z*) calculated for [C_21_H_18_BrNO_5_S + H] ^+^: 476.0162; found: 476.0180.

#### 5-((3-Isopropyl-2,4-dioxothiazolidin-5-ylidene)methyl)-2-methoxyphenyl 2-Fluorobenzoate (**29**)

White solid, yield: 64%. ^1^H NMR (400 MHz, Chloroform-*d*) δ 8.12 (dd, *J* = 7.8, 1.6 Hz, 1H), 7.79 (s, 1H), 7.58–7.47 (m, 2H), 7.47–7.37 (m, 2H), 7.35 (d, *J* = 2.3 Hz, 1H), 7.10 (d, *J* = 8.6 Hz, 1H), 4.71–4.64 (m, 1H), 3.91 (s, 3H), 1.49 (s, 3H), 1.47 (s, 3H). ^13^C NMR (101 MHz, CDCl_3_) δ 19.29, 19.29, 47.29, 56.20, 112.81, 119.99, 124.68, 126.51, 126.75, 128.63, 129.84, 131.42, 132.14, 132.22, 133.40, 134.72, 140.08, 152.99, 163.04, 166.37, 167.56.

#### 5-((3-Isopropyl-2,4-dioxothiazolidin-5-ylidene) Methyl)-2-methoxyphenyl 4-(Chloromethyl) Benzoate (**30**)

White solid, yield: 28%. ^1^H NMR (400 MHz, Chloroform-*d*) δ 8.25–8.18 (m, 2H), 7.79 (s, 1H), 7.55 (d, *J* = 8.2 Hz, 2H), 7.43 (dd, *J* = 8.7, 2.3 Hz, 1H), 7.32 (d, *J* = 2.2 Hz, 1H), 7.09 (d, *J* = 8.6 Hz, 1H), 4.71–4.64 (m, 3H), 3.88 (s, 3H), 1.49 (s, 3H), 1.47 (s, 3H). HRMS (ESI, *m*/*z*) calculated for [C_22_H_20_ClNO_5_S + H]^+^: 446.0823; found: 446.0811.

#### 5-((3-Isopropyl-2,4-dioxothiazolidin-5-ylidene) Methyl)-2-methoxyphenyl 3-Chloro-2,2-dimethylpropanoate (**31**)

White solid, yield: 33%. ^1^H NMR (400 MHz, Chloroform-*d*) δ 7.76 (s, 1H), 7.38 (dd, *J* = 8.6, 2.3 Hz, 1H), 7.20 (d, *J* = 2.2 Hz, 1H), 7.03 (d, *J* = 8.6 Hz, 1H), 4.70–4.63 (m, 1H), 3.87 (s, 3H), 3.77 (s, 2H), 1.48 (s, 3H), 1.47 (s, 9H). ^13^C NMR (101 MHz, CDCl_3_) δ 19.27, 19.27, 23.28, 23.28, 45.04, 47.27, 51.77, 56.08, 112.67, 119.96, 124.62, 126.47, 129.58, 132.12, 140.19, 152.92, 166.35, 167.56, 172.91. HRMS (ESI, *m*/*z*) calculated for [C_19_H_22_ClNO_5_S + H]^+^: 412.0980; found: 412.0962.

#### 5-((3-Isopropyl-2,4-dioxothiazolidin-5-ylidene) Methyl)-2-methoxyphenyl (3r, 5r, 7r)-Adamantane-1-carboxylate (**32**)

White solid, yield: 40%. ^1^H NMR (400 MHz, Chloroform-*d*) δ 7.75 (s, 1H), 7.36 (dd, *J* = 8.6, 2.3 Hz, 1H), 7.15 (d, *J* = 2.2 Hz, 1H), 7.02 (d, *J* = 8.6 Hz, 1H), 4.70–4.63 (m, 1H), 3.86 (s, 3H), 2.09 (s, 9H), 1.82–1.75 (m, 6H), 1.48 (s, 3H), 1.47 (s, 3H). ^13^C NMR (101 MHz, CDCl_3_) δ 19.28, 19.28, 27.92, 27.92, 27.92, 36.47, 36.47, 36.47, 38.80, 38.80, 38.80, 41.14, 47.24, 56.11, 112.59, 119.62, 124.68, 126.37, 129.36, 132.37, 140.63, 153.05, 166.40, 167.65, 175.47. HRMS (ESI, *m*/*z*) calculated for [C_25_H_29_NO_5_S + H]^+^:456.1839; found: 456.1844.

#### 5-((3-Isopropyl-2,4-dioxothiazolidin-5-ylidene)methyl)-2-methoxyphenylbuty Rate (**33**)

White solid, yield: 48%. ^1^H NMR (400 MHz, Chloroform-*d*) δ 7.76 (s, 1H), 7.37 (dd, *J* = 8.6, 2.3 Hz, 1H), 7.19 (d, *J* = 2.3 Hz, 1H), 7.04 (d, *J* = 8.6 Hz, 1H), 4.70–4.63 (m, 1H), 3.88 (s, 3H), 2.59 (t, *J* = 7.4 Hz, 2H), 1.86–1.77 (m, 2H), 1.49 (s, 3H), 1.47 (s, 3H), 1.07 (t, *J* = 7.4 Hz, 3H). ^13^C NMR (101 MHz, CDCl_3_) δ: 13.57, 18.52, 19.28, 19.28, 35.83, 47.27, 56.07, 112.64, 119.79, 124.62, 126.41, 129.59, 132.25, 140.24, 152.97, 166.39, 167.60, 171.39. HRMS (ESI, *m*/*z*) calculated for [C_18_H_21_NO_5_S + H]^+^: 364.1213; found: 364.1221.

#### 5-((3-Isobutyl-2,4-dioxothiazolidin-5-ylidene)methyl)-2-methoxyphenyl 3-Chloro-2,2-dimethylpropanoate(**34**)

White solid, yield: 82%. ^1^H NMR (400 MHz, Chloroform-*d*) δ 7.80 (s, 1H), 7.40 (dd, *J* = 8.6, 2.3 Hz, 1H), 7.22 (d, *J* = 2.3 Hz, 1H), 7.04 (d, *J* = 8.6 Hz, 1H), 3.88 (s, 3H), 3.77 (s, 2H), 3.57 (d, *J* = 7.5 Hz, 2H), 2.17–2.10 (m, 1H), 1.47 (s, 6H), 0.94 (s, 3H), 0.93 (s, 3H). ^13^C NMR (101 MHz, CDCl_3_) δ 19.96, 23.12, 23.27, 27.22, 44.43, 45.06, 49.08, 51.61, 51.76, 56.09, 112.69, 119.79, 124.64, 126.32, 129.68, 132.59, 140.19, 153.03, 166.65, 168.04, 172.94, 180.31. HRMS (ESI, *m*/*z*) calculated for [C_20_H_24_ClNO_5_S + H]^+^: 426.1136; found: 426.1151.

#### 5-((3-Isobutyl-2,4-dioxothiazolidin-5-ylidene)methyl)-2-methoxyphenyl 3-Bromobenzoate (**35**)

White solid, yield: 43%. ^1^H NMR (400 MHz, Chloroform-*d*) δ 8.36 (t, *J* = 1.8 Hz, 1H), 8.15 (dt, *J* = 7.8, 1.4 Hz, 1H), 7.83 (s, 1H), 7.80–7.77 (m, 1H), 7.47–7.39 (m, 2H), 7.33 (d, *J* = 2.3 Hz, 1H), 7.10 (d, *J* = 8.6 Hz, 1H), 3.89 (s, 3H), 3.58 (d, *J* = 7.5 Hz, 2H), 2.19–2.09 (m, 1H), 0.95 (s, 3H), 0.93 (s, 3H). ^13^C NMR (101 MHz, CDCl_3_) δ 19.97, 19.97, 27.23, 49.11, 56.17, 112.84, 119.97, 122.70, 124.59, 126.43, 128.95, 129.93, 130.19, 130.92, 132.47, 133.32, 136.71, 140.17, 153.06, 163.12, 166.63, 167.91. HRMS (ESI, *m*/*z*) calculated for [C_22_H_20_BrNO_5_S + H] ^+^: 490.0318; found: 490.0291.

#### 5-((3-Isobutyl-2,4-dioxothiazolidin-5-ylidene)methyl)-2-methoxyphenyl 4-(Chloromethyl)benzoate (**36**)

White solid, yield: 36%. ^1^H NMR (400 MHz, Chloroform-*d*) δ 8.21 (d, *J* = 8.3 Hz, 2H), 7.83 (s, 1H), 7.55 (d, *J* = 8.3 Hz, 2H), 7.45 (dd, *J* = 8.6, 2.3 Hz, 1H), 7.34 (d, *J* = 2.3 Hz, 1H), 7.10 (d, *J* = 8.7 Hz, 1H), 4.66 (s, 2H), 3.88 (s, 3H), 3.57 (d, *J* = 7.5 Hz, 2H), 2.18–2.11 (m, 1H), 0.95 (s, 3H), 0.93 (s, 3H). ^13^C NMR (101 MHz, CDCl_3_) δ 19.97, 19.97, 27.23, 30.20, 31.44, 45.29, 49.09, 56.16, 112.80, 119.85, 124.71, 126.38, 128.73, 128.92, 129.84, 130.85, 132.58, 140.32, 143.19, 153.18, 163.92, 166.66, 167.97. HRMS (ESI, *m*/*z*) calculated for [C_23_H_22_ClNO_5_S + H]^+^: 460.0980; found: 460.0991.

#### 5-((3-Isobutyl-2,4-dioxothiazolidin-5-ylidene) methyl)-2-methoxyphenyl (3r, 5r,7r)-adamantane-1-carboxylate (**37**)

White solid, yield: 48%. ^1^H NMR (400 MHz, Chloroform-*d*) δ 7.80 (s, 1H), 7.37 (dd, *J* = 8.6, 2.3 Hz, 1H), 7.17 (d, *J* = 2.3 Hz, 1H), 7.03 (d, *J* = 8.6 Hz, 1H), 3.87 (s, 3H), 3.57 (d, *J* = 7.5 Hz, 2H), 2.18–2.12 (m, 1H), 2.09 (s, 8H), 2.06–2.02 (m, 4H), 1.78 (s, 3H), 0.94 (s, 3H), 0.93 (s, 3H). ^13^C NMR (101 MHz, CDCl_3_) δ 19.96, 27.23, 27.68, 27.84, 27.93, 29.70, 36.32, 36.42, 36.47, 38.28, 38.64, 38.80, 40.37, 41.14, 42.23, 49.06, 56.12, 112.62, 119.53, 124.71, 126.27, 129.42, 132.79, 140.67, 153.18, 166.68, 168.10, 175.48, 181.98. HRMS (ESI, *m*/*z*) calculated for [C_26_H_31_NO_5_S + H]^+^: 470.1996; found: 470.1996.

#### 5-((3-Isopropyl-2,4-dioxothiazolidin-5-ylidene) methyl)-2-methoxyphenyl 4-Chlorobutanoate (**38**)

White solid, yield: 43%. ^1^H NMR (400 MHz, Chloroform-*d*) δ 7.80 (s, 1H), 7.43 (d, *J* = 8.6 Hz, 1H), 7.24 (s, 1H), 7.09 (d, *J* = 8.6 Hz, 1H), 4.74–4.67 (m, 1H), 3.93 (s, 3H), 3.74 (t, *J* = 6.3 Hz, 2H), 2.86 (t, *J* = 7.1 Hz, 2H), 2.32–2.25 (m, 2H), 1.53 (s, 3H), 1.51 (s, 3H). HRMS (ESI, *m*/*z*) calculated for [C_18_H_20_ClNO_5_S + H] ^+^: 398.0823; found: 398.0805.

#### 5-((3-Isopropyl-2,4-dioxothiazolidin-5-ylidene)methyl)-2-methoxyphenyl Propane-1-sulfonate (**39**)

White solid, yield: 71%. ^1^H NMR (400 MHz, Chloroform-*d*) δ 7.75 (s, 1H), 7.47–7.40 (m, 2H), 7.08 (d, *J* = 8.6 Hz, 1H), 4.70–4.63 (m, 1H), 3.95 (s, 3H), 3.35–3.27 (m, 2H), 2.13–1.99 (m, 2H), 1.48 (s, 3H), 1.47 (s, 3H), 1.14 (t, *J* = 7.5 Hz, 3H). ^13^C NMR (101 MHz, CDCl_3_) δ 12.95, 17.38, 19.27, 19.27, 47.36, 53.41, 56.30, 113.21, 120.89, 126.03, 126.76, 130.16, 131.49, 138.45, 153.14, 166.21, 167.32. HRMS (ESI, *m*/*z*) calculated for [C_17_H_21_NO_6_S_2_ + H]^+^:400.0883; found: 400.0871.

#### 5-((3-Isobutyl-2,4-dioxothiazolidin-5-ylidene)methyl)-2-methoxyphenyl Propane-1-sulfonate (**40**)

White solid, yield: 62%. ^1^H NMR (400 MHz, Chloroform-*d*) δ 7.79 (s, 1H), 7.47 (d, *J* = 2.3 Hz, 1H), 7.43 (dd, *J* = 8.6, 2.3 Hz, 1H), 7.09 (d, *J* = 8.6 Hz, 1H), 3.95 (s, 3H), 3.58 (d, *J* = 7.5 Hz, 2H), 3.35–3.27 (m, 2H), 2.21–2.06 (m, 2H), 2.06–1.99 (m, 1H), 1.14 (t, *J* = 7.5 Hz, 3H), 0.95 (s, 3H), 0.93 (s, 3H). ^13^C NMR (101 MHz, CDCl_3_) δ 12.93, 17.38, 19.95, 19.95, 27.22, 49.13, 53.43, 56.30, 113.24, 120.80, 126.03, 126.66, 130.19, 131.88, 138.49, 153.26, 166.48, 167.74. HRMS (ESI, *m*/*z*) calculated for [C_18_H_23_NO_6_S_2_ + H]^+^: 414.1040; found: 414.1051.

### Bioactivity Assays

#### Cell Culture and Transfection

Chinese hamster ovarian (CHO) cells collected from the American Type Culture Collection were maintained in Ham’s F-12K (Kaighn’s) supplemented with 100 mg/L streptomycin, 100 mg/L penicillin, and 10% fetal bovine serum (FBS) in a humidified atmosphere of 5% CO_2_ at 37 °C. CHO cells were co-transfected with plasmids encoding CB1 or CB2 and Gα16 by electroporation. To establish stable cell lines, transfected cells were seeded on 10 cm dishes with 40 μg/mL blasticidin and 1 mg/mL G418 in culture medium after 24 h. The selection medium was substituted once every 3 days until colonies formed. A solitary colony was obtained, propagated, and assessed using a calcium mobilization assay to verify the function and expression of the transfected genes.

#### Calcium Mobilization Assay

3 × 10^4^ cells/well were employed to seed cells onto 96-well plates, which were subsequently incubated throughout the night. Cells were then treated with 2 μM Fluo-4 AM in HBSS (0.3 mM Na_2_HPO_4_, 5.4 mM KCl, 4.2 mM NaHCO_3_, 0.4 mM KH_2_PO_4_, 0.5 mM MgCl_2_, 1.3 mM CaCl_2_, 137 mM NaCl, 0.6 mM MgSO_4_, 250 μM sulfinpyrazone, and 5.6 mM d-glucose, pH 7.4) for 45 min at 37 °C. In agonist mode, the extra dye was washed away and 50 μL of HBSS was included, followed by dispensation of 25 μL of HBSS with the test compound, DMSO (negative control), or CP55940 (positive control), into the wells through a FlexStation microplate reader. Meanwhile, the change of intracellular calcium was measured at an excitation wavelength of 525 and 485 nm. For antagonist mode, the extra dye was washed out and 50 μL of HBSS with test compounds, DMSO (negative control), or AM630 (positive control) was introduced. Following a 10 min incubation under room temperature, 25 μL of CP55940 was placed in the wells utilizing a FlexStation microplate reader. The change of intracellular calcium was captured at an excitation wavelength of 525 and 485 nm. To be noticed, each experiment has been independently replicated three times, with three repetitions and eight gradients each time.

#### Data Analysis

The collected data were processed with GraphPad Prism software (GraphPad). Nonlinear regression analysis was applied to produce dose–response curves and compute concentrations for half maximal effective concentration (EC_50_) values and 50% inhibition (IC_50_) values. Means ± SEM were computed utilizing this software. The analyses were evaluated by the Student *t* test. When the p value is smaller than 0.05, the result is statistically significant.

#### Computational Methods

We selected representative CB1 and CB2 ligands as a reference to generate the signature of CB1 or CB2 agonist. The residues of those ligands are supposed to have small deviations of the ligand–residue interaction energies among the CB1 or CB2 agonists. The experimental K_i_ values of reference ligands are shown in Table S7. The details of preparation procedures of those ligands, including ligand collection, homology modeling, MD simulations, and energy calculations, can be further found from previous publication.^48^ The docking procedure of derivatives is the same as that of reference ligands. For the selected representative ligands and synthesized compound series, a set of hierarchical computational methods, including homology modeling, molecular docking, molecular dynamics (MD) simulation, and molecular mechanics Poisson–Boltzmann/generalized Born surface area (MM-PB/GBSA) energy calculation, were applied. Then, interaction profiles (IPs) calculated by MM-GBSA were compared between derivatives and representative known CB ligands.

#### Molecular Dynamics Simulation

To study the dynamics of the CB receptor–ligand complexes, molecular dynamics simulations were conducted. Subsequent to this, binding free energy calculations were performed utilizing the molecular mechanics Poisson–Boltzmann surface area-WSAS (MM-PBSA-WSAS).^54,55^

To set up a MD system with the protein being inserted in a bilayer membrane, we employed CHARMM-GUI^56^ to incorporate TIP3P water molecules,^57^ 0.15 M Na^+^/Cl^–^ ions, and 1-palmitoyl-2-oleoyl-*sn*-glycero-3-phosphocholine bilayer lipids (POPC) into a rectangle box with approximate dimensions of 95 × 95 × 95 Å^3^. A typical system consisted of the cannabinoid receptor, a ligand staying in the binding site, approximately 17,000 TIP3P water molecules, 58 Cl^–^, 48 Na^+^, and 240 POPC lipid molecules.

During molecular mechanics (MM) minimizations and MD simulations, the force field parameters for POPC lipids, ligands, and proteins were obtained from the Lipid14 force field,^58^ FF14SB,^59^ and the general Amber force field (GAFF),^60^ respectively. The HF/6-31G* electrostatic potentials were produced by utilizing the Gaussian 16 software package.^61^ To fit them, the atomic partial charges were obtained using the RESP program.^62^ All of the residue topologies of ligands were produced by utilizing the Antechamber module.^63^ We use the PMEMD.cuda and PMEMD.mpi programs built in the AMBER 18 package to perform the MD simulations.^64−66^ For each MD system, the protocols were applied to conduct the minimizations and subsequent MD simulations. First, to eliminate potential steric crashes within the systems, five 10,000-step energy minimizations were initially implemented, with position restraint being applied to the main-chain atoms using a force constant from 20, 10, 5, 1, and eventually to 0 kcal/mol/Å^2^, subsequentially. The MD simulation stage can be separated into three phases. They are system relaxation, equilibrium, and sampling. During the relation phase, the system temperature gradually rose from 0 to 300 K in steps of 50 K, with a 0.1 ns MD simulation performed at each step. At this phase, the equations of motion were numerically integrated at a time step of 1 fs (fs). During the equilibrium and sampling phases, the time step was 2 fs and the temperature was 298.15 K. For the pressure regulation, an anisotropic scaling algorithm was employed with a relaxation constant of 2 ps. The reference pressure was 1 atm. The temperature was controlled using Langevin dynamics,^67^ and the collision frequency was 5 ps^–1^. The SHAKE algorithm was applied to restrain the hydrogen atoms. In total, 115 ns MD was performed for each protein–ligand system. 5000 MD snapshots were evenly collected from the last 100 ns in the sampling phase for postanalysis.

#### MM-PBSA Binding Free Energy Calculations

MM-PB/GBSA is an end-point approach broadly used for free energy calculations.^68−73^ Taking the dynamics into consideration, MD simulation is a common approach utilized to obtain representative conformations. WSAS is a solvent-accessible surface area-based method. It was developed to quickly compute how the conformational entropy changes during the binding between protein and ligand, for further improving the computational effectiveness in computing conformational entropy via normal mode analysis.^55^ In addition, the MM-PB/GBSA binding free energy is able to be partitioned into individual interaction terms, from which every energy part is calculated from several conformational snapshots obtained during simulations.^74^

The following formula describes how the free energy (Δ*G*_MM-PBSA_) of ligand–receptor binding in a complex is computed in MM-PBSA.
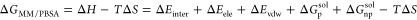
1where Δ*E*_inter_, Δ*E*_ele_, and Δ*E*_vdw_ represent changes in internal-bonded MM energy, MM electrostatic energy, and MM van der Waals energy, respectively. Meanwhile, variables Δ*G*_p_^sol^ and Δ*G*_np_^sol^ represent the polar and nonpolar solvation free energies, respectively. T is the absolute temperature, and Δ*S* represents the change of entropy. However, in a realistic situation, usually, only the complex state is simulated, leading to the removal of Δ*E*_inter_; thus, the equation is converted to
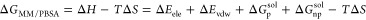
2The binding free energy for individual ligands was computed for every MD snapshot. The free energy decompositions were carried out for the collected 5000 snapshots. We applied a GBSA solvation model, which was developed by Hawkins et al.,^75^ to compute the LRIP to identify the interaction patterns. As for the binding free energy computations, we evenly selected 200 from the 5000 snapshots to conduct the MM-PBSA-WSAS analysis for each system. The Delphi program (http://compbio.clemson.edu/delphi) was applied to solve the Poisson–Boltzmann equation for the Δ*G*_p_^sol^ calculation.^69^

#### Ligand–Residue Interaction Profile (LRIP)

To systematically compare the LRIPs, we summarized the ligand–residue interaction energies for the CB1/CB2 active/inactive systems. To achieve this, we first selected known active compounds for each receptor according to the MM-PBSA binding free energies. In detail, AM-4030, AM-11542, THC, and WIN-55,212–2 were selected for active CB1; THC, AM-4030, WIN-55,212–2, and UR-144 were selected for active CB2; SR-147778, AM-251, MK-0364, and THC were selected for inactive CB1; AM-10257, AM-630, and THC were selected for inactive CB2. Then, we selected the key residues for each protein–ligand system if the ligand–residue interaction energies are stronger than −0.1 kcal/mol. Next, we calculated the average of these ligand–residue interaction energies for key residues. The mean IPs can be considered a signature of a receptor in an active or inactive form. At last, for a given compound in question, its IP similarity to the receptor IP signature was evaluated using four metrics, average standard error (ASE), root-mean-square deviation (RMSE), average unsigned error (AUE), and squared correlation coefficient (*R*^2^).

In this work, we are more interested in designing agonists. Thus, we first evaluated whether a query compound is an agonist in priority. If the R between a compound and selected known agonists is higher than 0.84 (*R*^2^ > 0.7) and meanwhile, the binding energy (Δ*E*) is better than −10 kcal/mol, then, it is considered to be a potential agonist. Otherwise, we determine that the compound is probably an antagonist or an undetermined compound (Figure 2).

## Supporting Information Available

Comparison results of interaction profiles between the evaluated compounds (including CP55940 and AM630) and representative agonists/antagonists; ligand–residue interaction energies (< −0.1 kcal/mol) of compound **6** in complex with CB2-active receptor and compound **39** in complex with CB2-inactive receptor; their ligand–residue interaction energies in the heatmap; experimental *K_i_* values of representative ligands; copies of ^1^H NMR, ^13^C NMR, and HR-MS spectra of all target compounds; templates of the MD input file and trajectory analysis (PDF)
Root-mean-square deviation (RMSD) plots of CB1/CB2 receptors and compounds (XLSX)

## Abbreviations Used

CB1/CB2: cannabinoid receptor 1/2
ECS: endocannabinoid system
GPCRS: G protein-coupled receptors
CNS: central nervous system
HR-MS: high-resolution mass spectrometry
IP: interaction profile
LRIP: ligand–residue interaction profile
MM-GBSA: molecular mechanics generalized Born surface area
MM-PBSA: molecular mechanics Poisson–Boltzmann surface area
MD: molecular dynamics
*R*: correlation coefficient
*R*^2^: squared correlation coefficient
SAR: structure–activity relationship
SI: selectivity index
TLC: thin-layer chromatography
POPC: 1-palmitoyl-2-oleoyl-*sn*-glycero-3-phosphocholine bilayer lipids
MM: molecular mechanics
ASE: average standard error
AUE: average unsigned error
RMSE: root-mean-square deviation
Δ*E*: binding energy
CHO: Chinese hamster ovarian
SEM: standard error of the mean
RMSD: root-mean-square deviation
