# Supplementary material for: Discovery of Potent and Selective CB2 Agonists Utilizing a Function-Based Computational Screening Protocol
Source: ACS Chem Neurosci. 2023 Oct 12;14(21):3941–58. doi: 10.1021/acschemneuro.3c00580 (PMC10623575; doi:10.1021/acschemneuro.3c00580)
Supplement: Supplementary file 1 — cn3c00580_si_001.pdf [file cn3c00580_si_001.pdf]

Discovery of Potent and Selective CB2 Agonists Utilizing a Function-Based Computational Screening Protocol

Supporting Information

Haixia Ge[1,⊥,*], Beihong Ji[2,⊥], Jiahui Fang[3,⊥], Jiayang Wang[1], Jing Li[3,*], Junmei Wang[2,*]

1. School of Life Sciences, Huzhou University, Huzhou 313000, China
2. Department of Pharmaceutical Sciences and Computational Chemical Genomics Screening Center, School of Pharmacy, University of Pittsburgh, Pittsburgh, PA 15261, USA.
3. Chinese Academy of Sciences Key Laboratory of Receptor Research, National Center for Drug Screening, Shanghai Institute of Materia Medica, Chinese Academy of Sciences, Shanghai 201203, China

Table S1 MM-PBSA calculation energy and metrics (ASE, AUE, RMSE, R^2, R) of compound series in CB1 active system. The unit of MM-PBSA energy is kcal/mol. The correctly predicted compounds are highlighted

Compound ID	ASE	AUE	RMSE	R^2	R	MM-PBSA
am630	-1.0511	1.1001	1.3864	0.4919	0.7013	-8.4571
cp55940	-0.9898	0.9898	1.2365	0.7880	0.8877	-9.8892
1	-1.0951	1.1041	1.3597	0.7227	0.8501	-13.1852
2	-1.0801	1.0801	1.3845	0.6498	0.8061	-8.4816
3	-1.0355	1.0851	1.2575	0.7971	0.8928	-11.3131
4	-1.0912	1.0912	1.3780	0.6608	0.8129	-11.4505
5	-1.0077	1.0863	1.3534	0.4997	0.7069	-12.2962
6	-1.0728	1.0736	1.3727	0.6543	0.8089	-10.3891
7	-1.1190	1.1190	1.4235	0.5797	0.7614	-8.1210
8	-1.0403	1.0811	1.3562	0.5735	0.7573	-10.9803
9	-1.1324	1.1324	1.4374	0.7025	0.8382	-10.0202
10	-1.0818	1.0818	1.3738	0.6395	0.7997	-10.0798
11	-0.9631	1.0189	1.2694	0.6181	0.7862	-13.1005
12	-1.0635	1.0792	1.4239	0.4417	0.6646	-11.7528
13	-1.1277	1.1820	1.4328	0.5375	0.7332	-11.5827
14	-1.0755	1.2221	1.4939	0.3247	0.5698	-15.4422
15	-1.0689	1.0689	1.3516	0.6877	0.8293	-11.2091
16	-1.1310	1.1310	1.3998	0.7220	0.8497	-10.1332
17	-1.0630	1.0630	1.3450	0.6803	0.8248	-9.1987
18	-1.1213	1.1213	1.4658	0.4803	0.6930	-5.6184
19	-0.9979	1.0241	1.2716	0.7505	0.8663	-15.0719
20	-1.0467	1.0467	1.4102	0.4593	0.6777	-10.5635
21	-1.0612	1.0777	1.2956	0.8283	0.9101	-12.2104
22	-1.1939	1.1939	1.4590	0.7892	0.8884	-8.1997
23	-1.0466	1.0773	1.3842	0.5218	0.7223	-8.6249
24	-1.0661	1.0794	1.3805	0.6006	0.7750	-3.1256
25	-1.1507	1.1507	1.4426	0.6621	0.8137	-10.1620
26	-1.0127	1.0265	1.3251	0.6168	0.7854	-14.9018
27	-1.0807	1.0898	1.3646	0.6417	0.8011	-12.9049
28	-1.0970	1.1302	1.4021	0.5369	0.7328	-10.6032
29	-1.0512	1.0512	1.3094	0.7625	0.8732	-12.5649
30	-0.9907	1.0366	1.3366	0.5671	0.7530	-10.3362
31	-1.1114	1.1114	1.3760	0.7157	0.8460	-10.6473
32	-0.9706	0.9762	1.2753	0.6533	0.8083	-17.6331
33	-1.1141	1.1178	1.3933	0.7147	0.8454	-9.6424
34	-1.0313	1.0440	1.3051	0.7358	0.8578	-11.9313
35	-1.0461	1.0461	1.3329	0.6926	0.8322	-12.9494
36	-0.9931	1.0446	1.3236	0.5814	0.7625	-10.8378
37	-0.9600	0.9668	1.2753	0.5995	0.7743	-18.8555
38	-1.0544	1.0829	1.4176	0.5246	0.7243	-7.8325
39	-1.2262	1.2807	1.5850	0.4190	0.6473	-6.5070

40	-1.0761	1.1731	1.5078	0.3088	0.5557	-8.7820

Table S2 MM-PBSA calculation energy and metrics (ASE, AUE, RMSE, R^2, R) of compound series in CB2 active system. The unit of MM-PBSA energy is kcal/mol. The correctly predicted compounds are highlighted

Compound ID	ASE	AUE	RMSE	R^2	R	MM-PBSA
am630	-1.0904	1.1476	1.4057	0.4215	0.6492	-7.1296
cp55940	-0.8448	0.8457	1.0307	0.8544	0.9243	-14.4288
1	-1.0989	1.0989	1.3962	0.4335	0.6584	-11.3935
2	-1.0322	1.0322	1.2318	0.7795	0.8829	-10.6895
3	-1.2059	1.2157	1.5297	0.2926	0.5409	-8.1386
4	-1.1091	1.1091	1.2915	0.7820	0.8843	-9.6576
5	-1.0305	1.0305	1.2523	0.7624	0.8732	-10.8432
6	-1.1227	1.1227	1.3190	0.8634	0.9292	-8.4332
7	-1.2167	1.2199	1.4541	0.5950	0.7714	-10.6652
8	-1.0035	1.0035	1.2152	0.7715	0.8783	-11.5223
9	-1.1555	1.1555	1.3416	0.7487	0.8653	-11.6509
10	-1.0766	1.0766	1.2467	0.9271	0.9629	-6.7205
11	-1.0334	1.0448	1.2418	0.6354	0.7971	-13.7845
12	-1.0971	1.0971	1.2708	0.8564	0.9254	-11.6718
13	-1.0746	1.0746	1.2433	0.8597	0.9272	-7.4448
14	-1.0851	1.0851	1.2634	0.8405	0.9168	-15.5671
15	-1.0630	1.0630	1.2517	0.8331	0.9127	-10.9315
16	-1.1777	1.1777	1.3463	0.7813	0.8839	-8.7475
17	-1.0917	1.1103	1.3554	0.7813	0.8839	-7.1778
18	-1.1408	1.1536	1.3933	0.5296	0.7278	-5.2586
19	-1.0263	1.0263	1.2225	0.7313	0.8552	-16.0291
20	-1.1393	1.1393	1.3321	0.6642	0.8150	-11.2474
21	-1.1683	1.1849	1.4681	0.3887	0.6235	-4.9737
22	-1.2390	1.2415	1.5105	0.4210	0.6488	-8.9095
23	-1.0790	1.0790	1.2976	0.6397	0.7998	-10.4622
24	-1.0372	1.0372	1.2417	0.8308	0.9115	-10.7325
25	-1.2536	1.3060	1.5329	0.3759	0.6131	-5.2656
26	-1.1738	1.2034	1.3668	0.6387	0.7992	-9.8524
27	-1.2785	1.3849	1.6343	0.2108	0.4592	-11.6593
28	-1.1087	1.1087	1.3344	0.7015	0.8375	-11.2789
29	-1.1373	1.1373	1.3299	0.7934	0.8908	-7.9895
30	-1.0476	1.0570	1.3323	0.4988	0.7062	-11.1263
31	-1.0500	1.0500	1.2764	0.6563	0.8101	-10.3886
32	-0.8778	0.8778	1.1187	0.7222	0.8498	-13.2928
33	-1.2184	1.2184	1.5239	0.3281	0.5728	-9.0418
34	-1.1114	1.1293	1.3152	0.6267	0.7916	-13.6977
35	-1.1772	1.2164	1.5306	0.2419	0.4919	-12.0463
36	-1.1138	1.1138	1.2661	0.8650	0.9301	-5.6637

37	-1.0056	1.0056	1.2295	0.6734	0.8206	-14.6095
38	-1.0418	1.0418	1.2379	0.7708	0.8780	-10.8647
39	-1.0100	1.0214	1.2675	0.6176	0.7859	-10.5626
40	-0.9662	0.9889	1.2119	0.6652	0.8156	-10.6084

Table S3 MM-PBSA calculation energy and metrics (ASE, AUE, RMSE, R^2, R) of compound series in CB1 inactive system. The unit of MM-PBSA energy is kcal/mol. The correctly predicted compounds are highlighted

Compound ID	ASE	AUE	RMSE	R^2	R	MM-PBSA
am630	-0.8896	0.9405	1.2507	0.4809	0.6934	-13.5886
cp55940	-0.8019	0.8167	1.0042	0.8833	0.9399	-14.7773
1	-0.9519	1.0309	1.3113	0.5270	0.7259	-10.9943
2	-0.8802	0.8847	1.1115	0.7770	0.8815	-11.8355
3	-0.9839	1.0169	1.2144	0.7288	0.8537	-9.8156
4	-0.9541	1.0042	1.2596	0.5742	0.7577	-13.3965
5	-1.0135	1.0238	1.3027	0.6356	0.7972	-9.3401
6	-0.9727	0.9727	1.1903	0.7933	0.8907	-11.0920
7	-1.0472	1.1013	1.2559	0.7102	0.8427	-8.0915
8	-0.9053	0.9097	1.1592	0.7963	0.8923	-12.6789
9	-0.9226	1.0123	1.2586	0.5957	0.7718	-10.3733
10	-0.9229	0.9229	1.1417	0.8110	0.9005	-12.6416
11	-0.9687	0.9707	1.3599	0.3883	0.6232	-14.1573
12	-1.0717	1.1142	1.3961	0.4704	0.6859	-10.0275
13	-0.9286	0.9436	1.2296	0.6564	0.8102	-9.7846
14	-0.8482	0.9035	1.1786	0.6289	0.7930	-14.6483
15	-0.8745	0.9983	1.3131	0.3570	0.5975	-14.5826
16	-0.9380	0.9636	1.2817	0.5375	0.7332	-9.1612
17	-0.9084	0.9222	1.2665	0.5180	0.7197	-7.2307
18	-0.9590	0.9590	1.1864	0.8258	0.9087	-9.4764
19	-0.8636	1.0938	1.4296	0.1732	0.4162	-14.9801
20	-0.8914	1.0405	1.3412	0.3283	0.5730	-11.2255
21	-0.9769	0.9769	1.2313	0.8415	0.9173	-7.6093
22	-1.0109	1.0302	1.3489	0.4892	0.6994	-8.7937
23	-0.9901	1.0551	1.4111	0.3208	0.5664	-10.1628
24	-1.0049	1.1129	1.4694	0.2276	0.4770	-8.6576
25	-0.9385	0.9883	1.3304	0.4220	0.6496	-11.1915
26	-0.9880	1.0119	1.2114	0.7046	0.8394	-11.9168
27	-0.9976	1.1291	1.4027	0.3464	0.5885	-15.5286
28	-0.8735	0.9279	1.1385	0.7486	0.8652	-11.0234
29	-0.8999	0.9072	1.1512	0.7517	0.8670	-10.2171
30	-1.0073	1.0116	1.2087	0.7791	0.8827	-8.3179
31	-0.9215	0.9585	1.1311	0.7780	0.8820	-10.3606
32	-0.9405	0.9657	1.1658	0.7204	0.8487	-14.2177
33	-1.0154	1.0154	1.2351	0.7011	0.8373	-6.4075

34	-0.8783	0.9110	1.2419	0.5337	0.7305	-10.5774
35	-0.8840	0.9477	1.2782	0.4446	0.6668	-15.0046
36	-0.8450	0.8955	1.2348	0.4616	0.6794	-16.9042
37	-1.0504	1.0891	1.3937	0.4717	0.6868	-11.8722
38	-0.9403	0.9403	1.0972	0.8806	0.9384	-6.8324
39	-	-	-	-	-	-
40	-0.9844	0.9844	1.1381	0.9045	0.9510	-4.8200

Table S4 MM-PBSA calculation energy and metrics (ASE, AUE, RMSE, R^2, R) of compound series in CB2 inactive system. The unit of MM-PBSA energy is kcal/mol. The correctly predicted compounds are highlighted

Compound ID	ASE	AUE	RMSE	R^2	R	MM-PBSA
am630	-0.8896	0.9405	1.2507	0.4809	0.6934	-8.5794
cp55940	-0.8019	0.8167	1.0042	0.8833	0.9399	-10.2640
1	-1.2664	1.2664	1.4139	0.7015	0.8376	-8.1972
2	-0.8802	0.8847	1.1115	0.7770	0.8815	-9.3252
3	-1.1972	1.2151	1.4194	0.4511	0.6716	-5.3401
4	-1.1694	1.2020	1.3732	0.5075	0.7124	-9.0001
5	-1.0135	1.0238	1.3027	0.6356	0.7972	-14.7847
6	-0.9727	0.9727	1.1903	0.7933	0.8907	-7.7182
7	-1.3449	1.3559	1.5896	0.2927	0.5410	-9.7320
8	-0.9053	0.9097	1.1592	0.7963	0.8923	-15.0961
9	-1.0228	1.0700	1.2427	0.5157	0.7181	-11.4581
10	-0.9229	0.9229	1.1417	0.8110	0.9005	-7.8208
11	-1.2276	1.2496	1.4516	0.4206	0.6486	-6.4863
12	-1.1906	1.2017	1.4188	0.4321	0.6573	-13.7278
13	-0.9286	0.9436	1.2296	0.6564	0.8102	-8.4462
14	-0.8482	0.9035	1.1786	0.6289	0.7930	-10.4175
15	-0.8745	0.9983	1.3131	0.3570	0.5975	-10.8869
16	-0.9380	0.9636	1.2817	0.5375	0.7332	-7.9868
17	-0.9084	0.9222	1.2665	0.5180	0.7197	-8.2353
18	-0.9590	0.9590	1.1864	0.8258	0.9087	-7.2842
19	-1.2024	1.2292	1.4290	0.4848	0.6963	-12.0951
20	-1.0529	1.1240	1.3325	0.3418	0.5846	-10.8079
21	-0.9769	0.9769	1.2313	0.8415	0.9173	-7.3369
22	-1.0109	1.0302	1.3489	0.4892	0.6994	-4.4685
23	-0.9901	1.0551	1.4111	0.3208	0.5664	-6.1661
24	-1.0876	1.0876	1.3210	0.4532	0.6732	-9.8957
25	-1.0326	1.0836	1.2811	0.4321	0.6573	-7.8259
26	-1.0420	1.1049	1.3625	0.2550	0.5049	-10.9465
27	-1.0349	1.0990	1.3450	0.2706	0.5202	-12.5278
28	-0.8735	0.9279	1.1385	0.7486	0.8652	-14.6285
29	-1.0662	1.0662	1.2245	0.8316	0.9119	-5.8786
30	-1.0073	1.0116	1.2087	0.7791	0.8827	-

31	-0.9215	0.9585	1.1311	0.7780	0.8820	-10.1744
32	-0.9405	0.9657	1.1658	0.7204	0.8487	-
33	-1.1306	1.1826	1.3720	0.4063	0.6374	-4.8571
34	-1.3125	1.3125	1.5192	0.4463	0.6681	-8.1228
35	-1.3888	1.3888	1.5584	0.5754	0.7586	-13.2612
36	-1.2004	1.2004	1.3341	0.7492	0.8656	-12.1814
37	-1.5245	1.5245	1.7316	0.3637	0.6031	-13.8741
38	-0.9403	0.9403	1.0972	0.8806	0.9384	-5.6485
39	-	-	-	-	-	-6.0369
40	-0.9844	0.9844	1.1381	0.9045	0.9510	-5.1387

Table S5 Ligand-residue interaction energies (<-0.1 kcal/mol) of Compound 6 in complex with CB2 active receptor. Residue ID1 represents general residue numbering scheme, while Residue ID2 represents Ballesteros-Weinstein residue numbering scheme. The unit of energy is kcal/mol.

Residue ID1	Residue ID2	Energy
F87	F2.57	-0.7480
S90	S2.60	-0.9363
F91	F2.61	-1.4414
F94	F2.64	-1.1839
H95	H2.65	-0.4539
F106	F3.25	-0.7094
K109	K3.28	-0.3685
I110	I3.29	-2.0124
V113	V3.32	-1.2518
T114	T3.33	-1.1754
F117	F3.36	-1.0961
L182	L182	-0.1199
F183	F183	-2.1599
P184	P184	-0.2093
I186	I186	-0.4955
Y190	Y5.39	-0.4756
L191	L5.40	-0.5809
W194	W5.43	-1.3543
W258	W6.48	-0.3291
V261	V6.51	-0.2456
M265	M6.55	-0.6902
F281	F7.35	-0.6062
S285	S7.39	-1.0532
C288	C7.42	-0.4172
L289	L7.43	-0.1856

Table S6 Ligand-residue interaction energies (<-0.1 kcal/mol) of Compound 39 in complex with CB2 inactive receptor. Residue ID1 represents general residue numbering scheme, while Residue ID2 represents Ballesteros-Weinstein residue numbering scheme. The unit of energy is kcal/mol.

Residue ID1	Residue ID2	Energy
L17	L17	-1.2402
P21	P21	-0.6623
S47	S1.46	-0.1672
D80	D2.50	-0.1259
A83	A2.53	-0.7718
S84	S2.54	-0.4760
V86	V2.56	-0.2598
F87	F2.57	-1.4311
I110	I3.29	-0.5939
V113	V3.32	-1.9140
T114	T3.33	-1.0954
T116	T3.35	-0.1394
F117	F3.36	-0.2222
F183	F183	-1.1246
L191	L5.40	-0.3011
W194	W5.43	-0.7830
W258	W6.48	-1.3057
V261	V6.51	-0.7837
L262	L6.52	-0.4414
M265	M6.55	-0.5486
F281	F7.35	-0.2258
S285	S7.39	-1.2644
M286	M7.40	-0.4207
L287	L7.41	-0.1006
C288	C7.42	-1.5244
L289	L7.43	-1.3663
S292	S7.46	-0.3909

Table S7 Experimental K_i values of representative ligands for active/inactive CB1/CB2 receptors.

Active CB1		Active CB2		Inactive CB1		Inactive CB2	
AM-11542	1.3 [1], 0.11[2]	UR-144	1.8[3]	AM-251	7.5[4]	AM-10257	0.08[5]
AM-4030	0.7[6, 7]	AM-4030	8.6[6, 7]	MK-0364	0.13[8]	AM-630	31.2[9]
THC	2.9[10]	THC	41[10]	THC	NA	THC	NA
WIN-55,212-2	9.2[11]	WIN-55,212-2	2.1[11]	SR-147778	3.5[12]		

1. Kawakami, J. K.; Martinez, Y.; Sasaki, B.; Harris, M.; Kurata, W. E.; Lau, A. F., Investigation of a novel molecular descriptor for the lead optimization of 4-aminoquinazolines as vascular endothelial growth factor receptor-2 inhibitors: application for quantitative structure-activity relationship analysis in lead optimization. *Bioorg. Med. Chem. Lett.* **2011,** *21* (5), 1371-1375.
2. Hua, T.; Vemuri, K.; Nikas, S. P.; Laprairie, R. B.; Wu, Y.; Qu, L.; Pu, M.; Korde, A.; Jiang, S.; Ho, J.-H.; Han, G. W.; Ding, K.; Li, X.; Liu, H.; Hanson, M. A.; Zhao, S.; Bohn, L. M.; Makriyannis, A.; Stevens, R. C.; Liu, Z.-J., Crystal structures of agonist-bound human cannabinoid receptor CB1. *Nature* **2017,** *547* (7664), 468-471.
3. Frost, J. M.; Dart, M. J.; Tietje, K. R.; Garrison, T. R.; Grayson, G. K.; Daza, A. V.; El-Kouhen, O. F.; Yao, B. B.; Hsieh, G. C.; Pai, M.; Zhu, C. Z.; Chandran, P.; Meyer, M. D., Indol-3-ylcycloalkyl Ketones: Effects of N1 Substituted Indole Side Chain Variations on CB2 Cannabinoid Receptor Activity. *J. Med. Chem.* **2010,** *53* (1), 295-315.
4. Lan, R.; Liu, Q.; Fan, P.; Lin, S.; Fernando, S. R.; McCallion, D.; Pertwee, R.; Makriyannis, A., Structure−Activity Relationships of Pyrazole Derivatives as Cannabinoid Receptor Antagonists. *J. Med. Chem.* **1999,** *42* (4), 769-776.
5. Schrodinger, LLC, The PyMOL Molecular Graphics System, Version 1.8. 2015.
6. Drake, D. J.; Jensen, R. S.; Busch-Petersen, J.; Kawakami, J. K.; Concepcion Fernandez-Garcia, M.; Fan, P.; Makriyannis, A.; Tius, M. A., Classical/Nonclassical Hybrid Cannabinoids: Southern Aliphatic Chain-Functionalized C-6β Methyl, Ethyl, and Propyl Analogues. *J. Med. Chem.* **1998,** *41* (19), 3596-3608.
7. Tius, M. A.; Hill, W. A. G.; Zou, X. L.; Busch-Petersen, J.; Kawakami, J. K.; Fernandez-Garcia, M. C.; Drake, D. J.; Abadji, V.; Makriyannis, A., Classical/non-classical cannabinoid hybrids; Stereochemical requirements for the southern hydroxyalkyl chain. *Life Sci.* **1995,** *56* (23), 2007-2012.
8. Shao, Z.; Yin, J.; Chapman, K.; Grzemska, M.; Clark, L.; Wang, J.; Rosenbaum, D. M., High-resolution crystal structure of the human CB1 cannabinoid receptor. *Nature* **2016,** *540*, 602.
9. Lavey, B. J.; Kozlowski, J. A.; Hipkin, R. W.; Gonsiorek, W.; Lundell, D. J.; Piwinski, J. J.; Narula, S.; Lunn, C. A., Triaryl bis-sulfones as a new class of cannabinoid CB2 receptor inhibitors: identification of a lead and initial SAR studies. *Bioorg. Med. Chem. Lett.* **2005,** *15* (3), 783-786.
10. Pagé, D.; Balaux, E.; Boisvert, L.; Liu, Z.; Milburn, C.; Tremblay, M.; Wei, Z.; Woo, S.; Luo, X.; Cheng, Y.-X.; Yang, H.; Srivastava, S.; Zhou, F.; Brown, W.; Tomaszewski, M.; Walpole, C.; Hodzic, L.; St-Onge, S.; Godbout, C.; Salois, D.; Payza, K., Novel benzimidazole derivatives as selective CB2 agonists. *Bioorg. Med. Chem. Lett.* **2008,** *18*

(13), 3695-3700.

11. Tonelli, M.; Cichero, E.; Mahmoud, A. M.; Rabbito, A.; Tasso, B.; Fossa, P.; Ligresti, A., Exploring the effectiveness of novel benzimidazoles as CB2 ligands: synthesis, biological evaluation, molecular docking studies and ADMET prediction. *MedChemComm.* **2018,** *9* (12), 2045-2054.

12. Rinaldi-Carmona, M.; Barth, F.; Congy, C.; Martinez, S.; Oustric, D.; Perio, A.; Poncelet, M.; Maruani, J.; Arnone, M.; Finance, O.; Soubrie, P.; Le Fur, G., SR147778, a new potent and selective antagonist of the CB1 cannabinoid receptor. Biochemical and pharmacological characterization. *J. Pharmacol. Exp. Ther.* **2004,** *310*, 905-914.

Copies of ^{1}H NMR, ^{13}C NMR and HR-MS Spectra of all target compounds

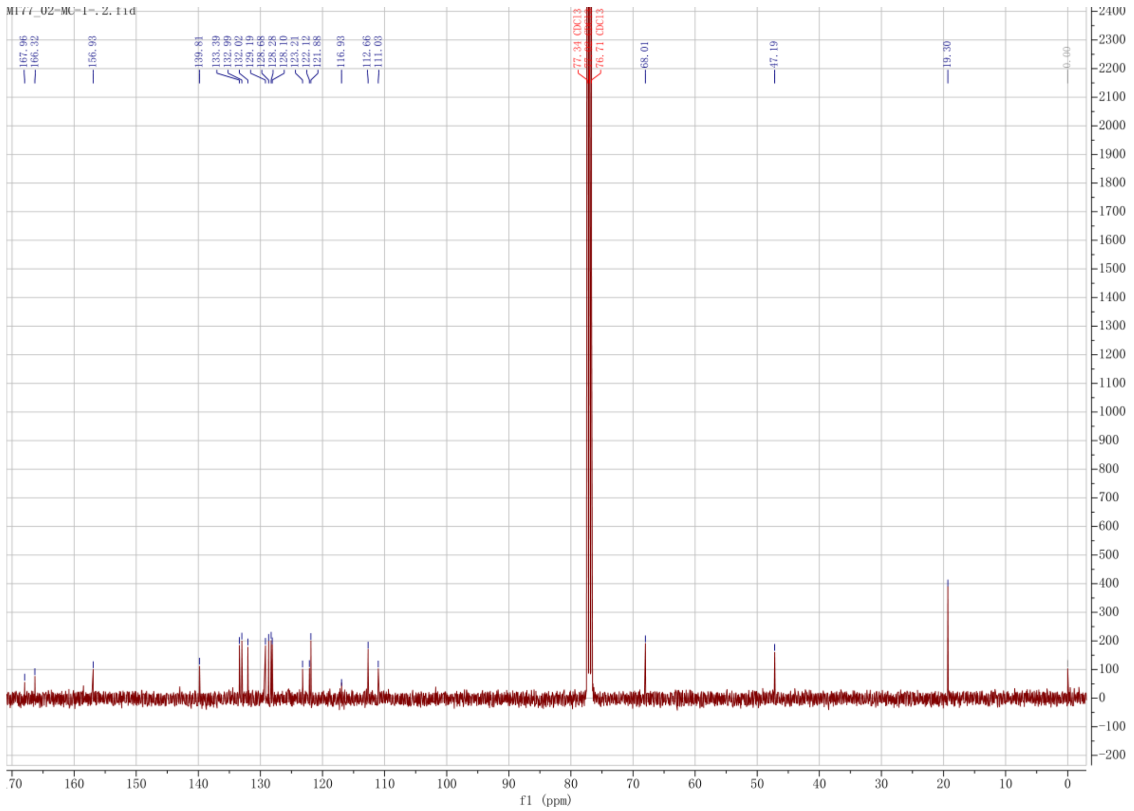

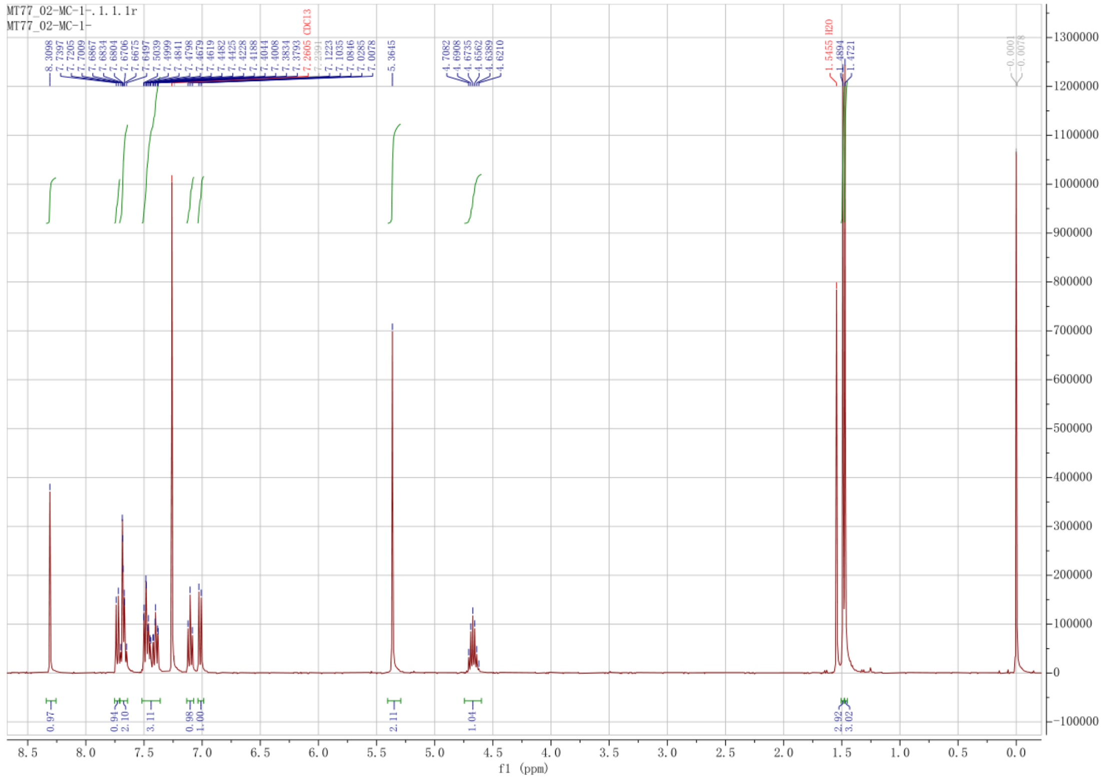

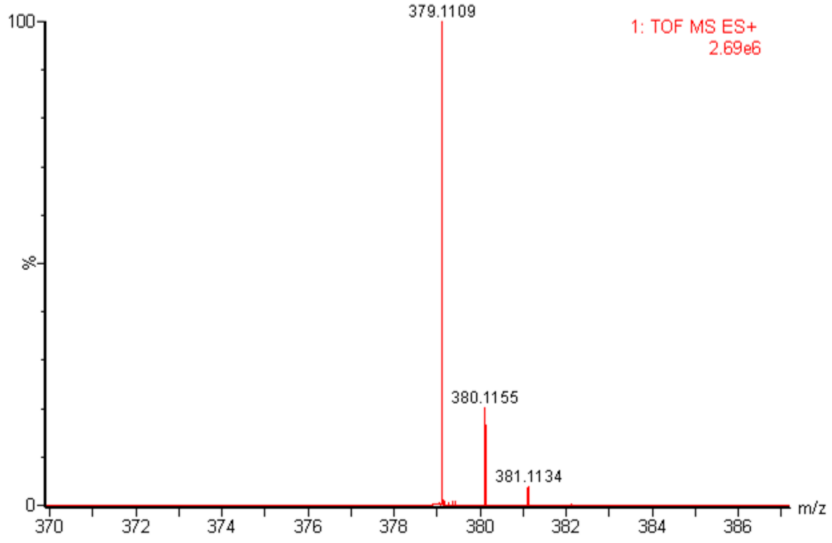

^{1}H NMR, ^{13}C NMR and HR-MS Spectra of compound 1

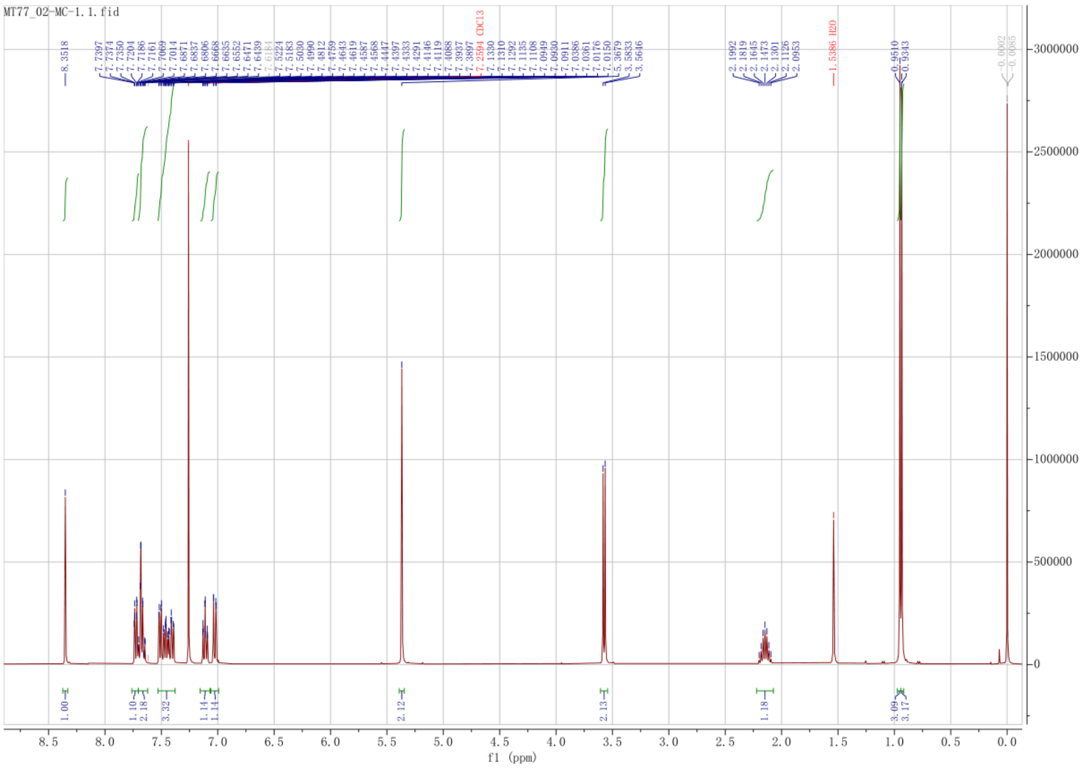
MT77_02-MC-1.1.fid
f1 (ppm)

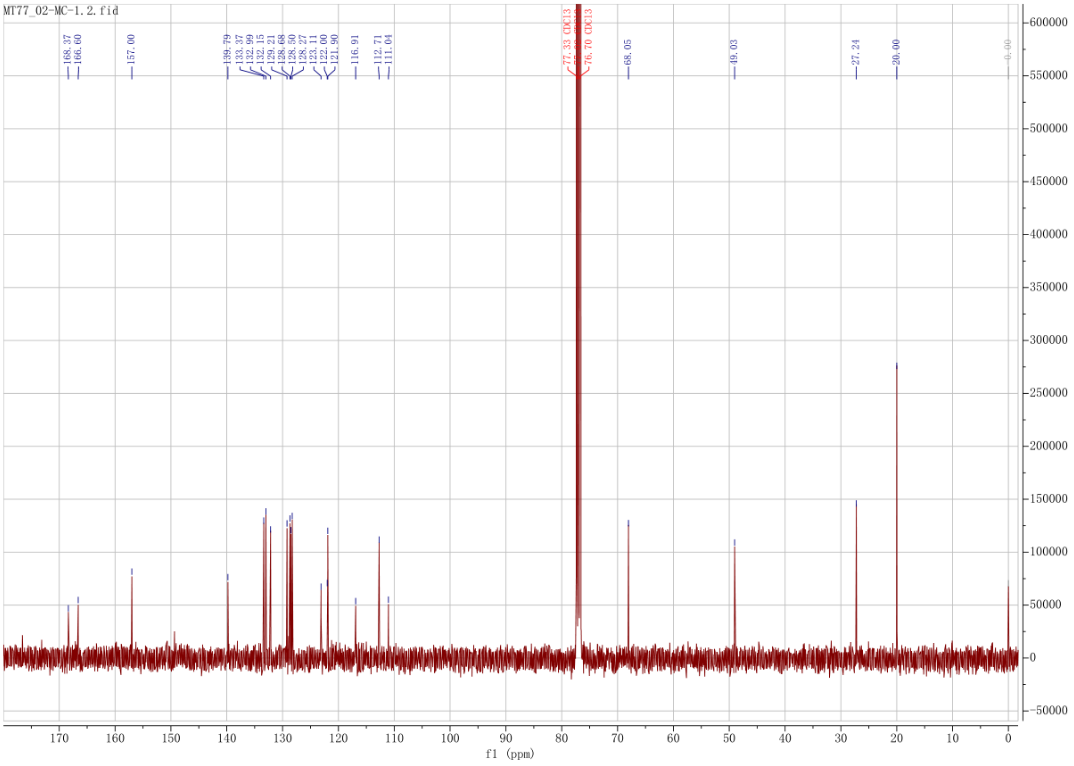
MT77_02-MC-1.2.fid
f1 (ppm)

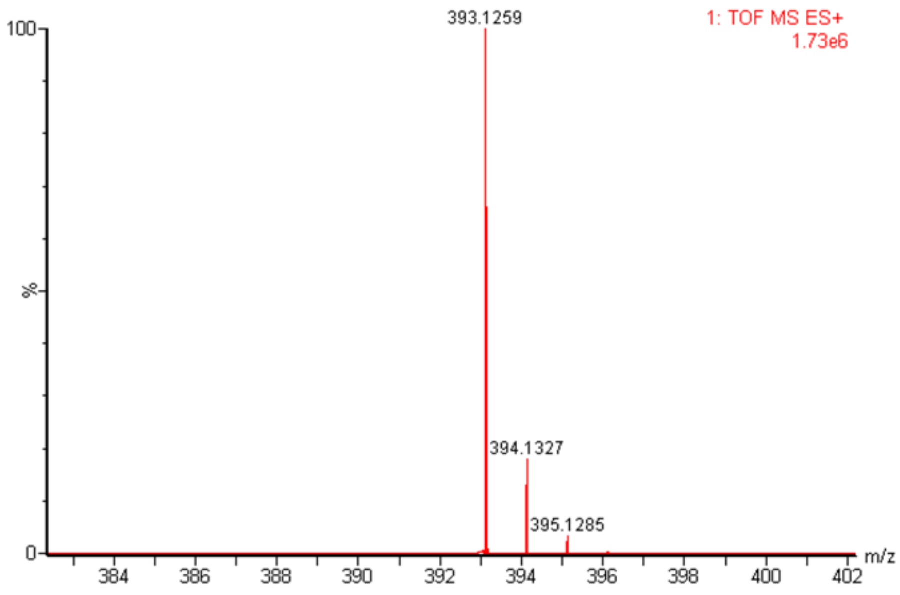

^{1}H NMR, ^{13}C NMR and HR-MS Spectra of compound 2

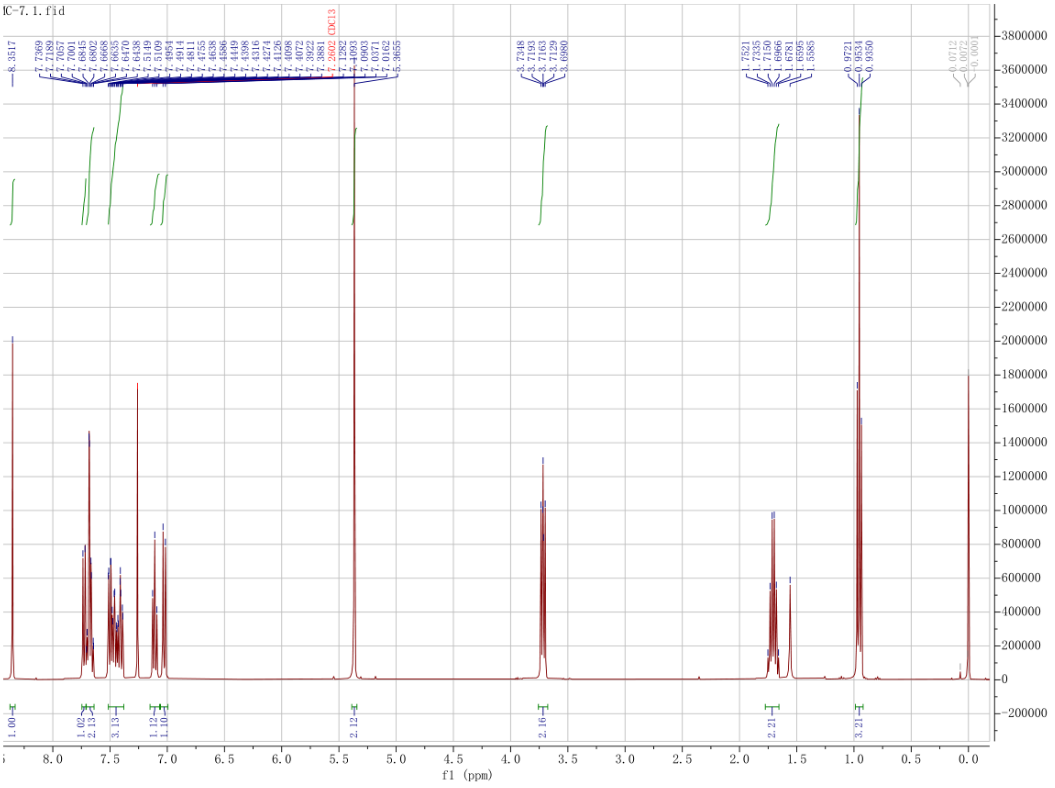

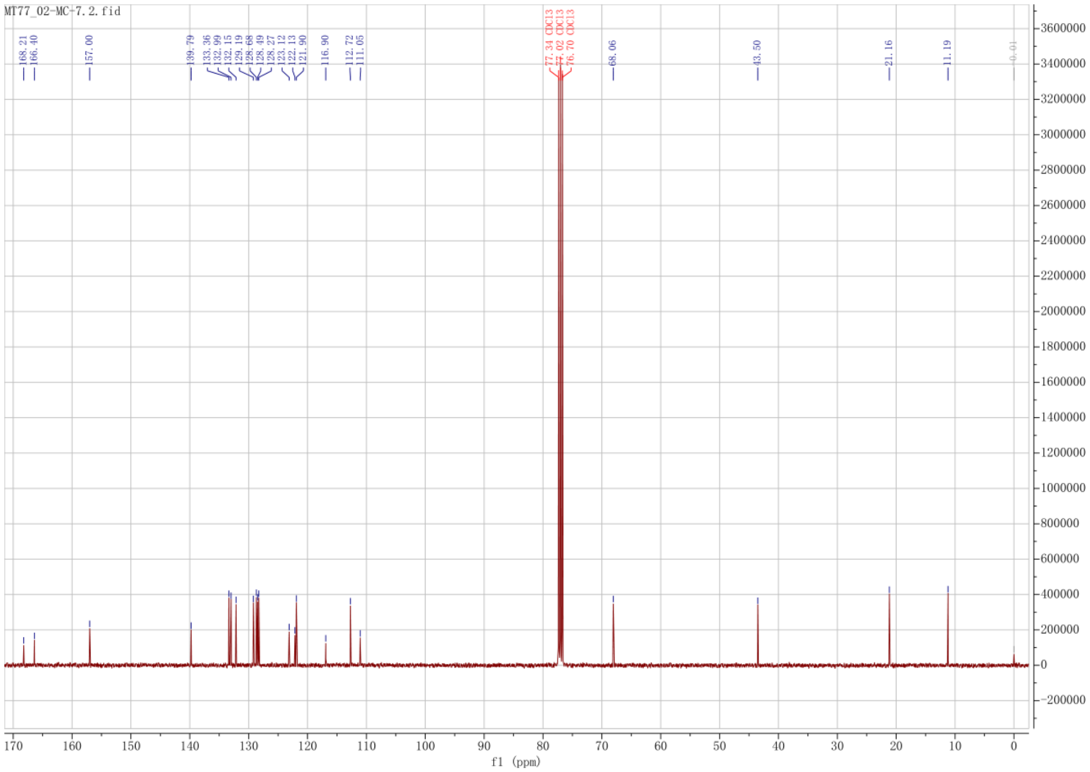

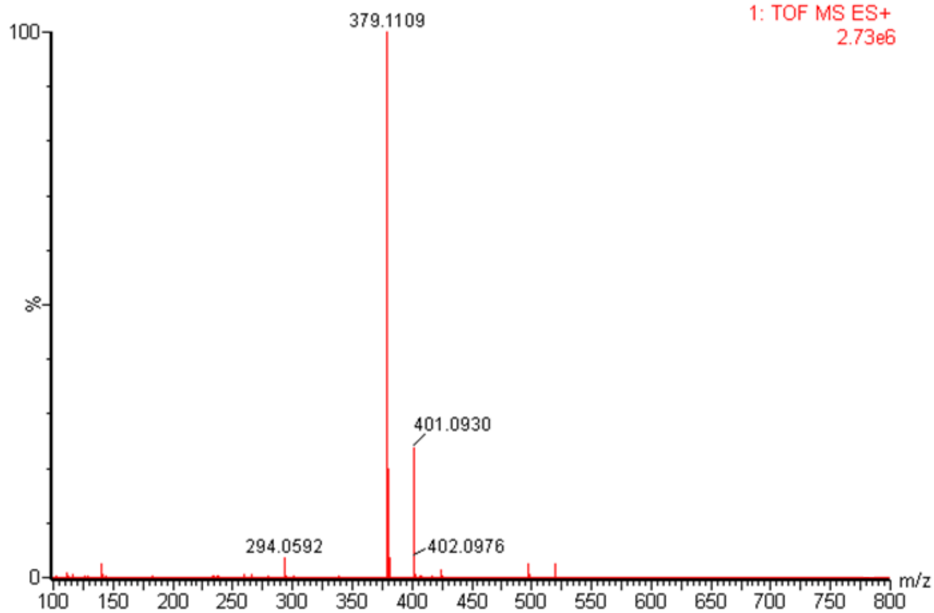

^{1}H NMR, ^{13}C NMR and HR-MS Spectra of compound 3

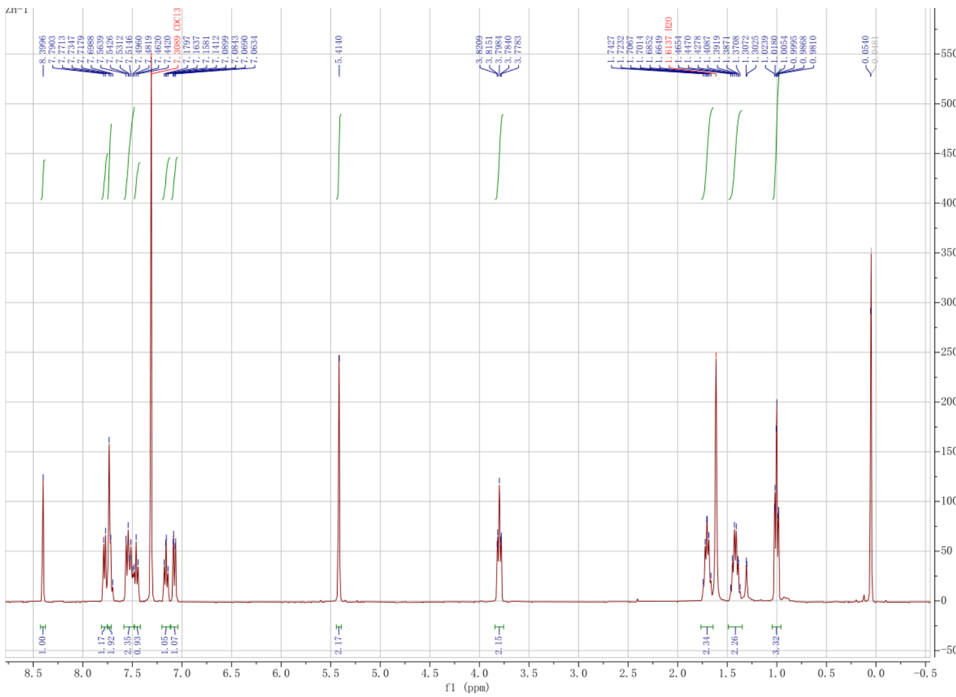
ZH-1
8.3996
7.7903
7.7713
7.7347
7.7179
7.6988
7.5639
7.5426
7.5312
7.5116
7.4960
7.4819
7.4620
7.4420
7.3089 CDCl3
7.1797
7.1637
7.1581
7.1412
7.0899
7.0843
7.0690
7.0634
5.4140
3.8209
3.8151
3.7984
3.7840
3.7783
1.7427
1.7232
1.7067
1.7014
1.6852
1.6649
1.6137 H2O
1.4654
1.4470
1.4278
1.4087
1.3919
1.3871
1.3708
1.3072
1.3025
1.0239
1.0180
1.0054
0.9995
0.9868
0.9810
0.0540
1.00
1.17
1.92
2.35
0.93
1.05
1.07
2.17
2.15
2.34
2.26
3.32
f1 (ppm)

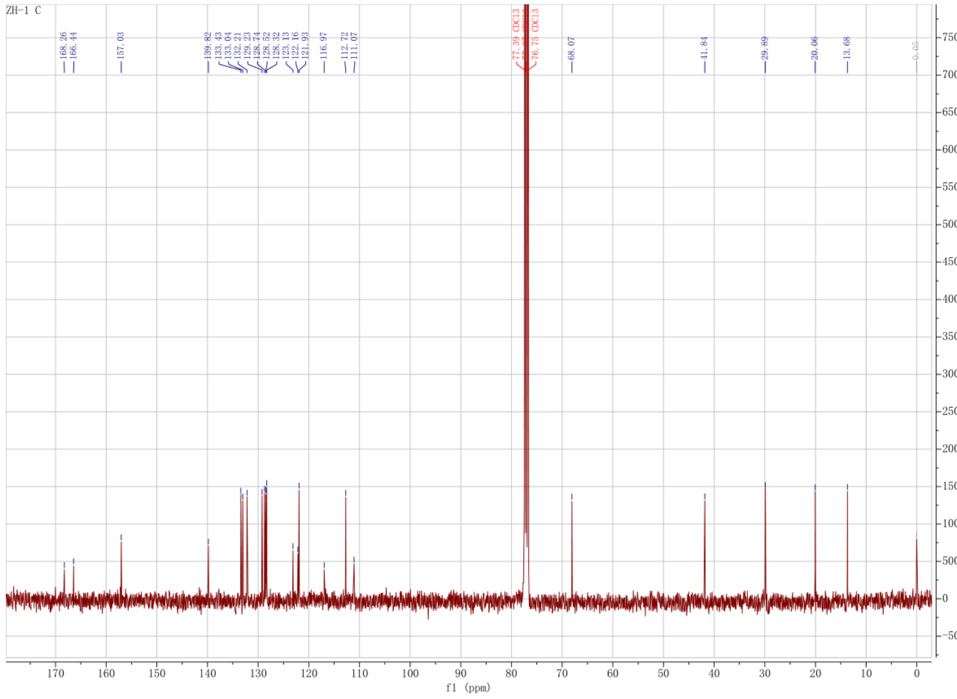
ZH-1 C
168.26
166.44
157.03
139.82
133.43
133.04
132.21
129.23
128.74
128.52
128.32
123.13
122.16
121.93
116.97
112.72
111.07
77.39 CDCl3
76.75 CDCl3
68.07
41.84
29.89
20.06
13.68
0.05
f1 (ppm)

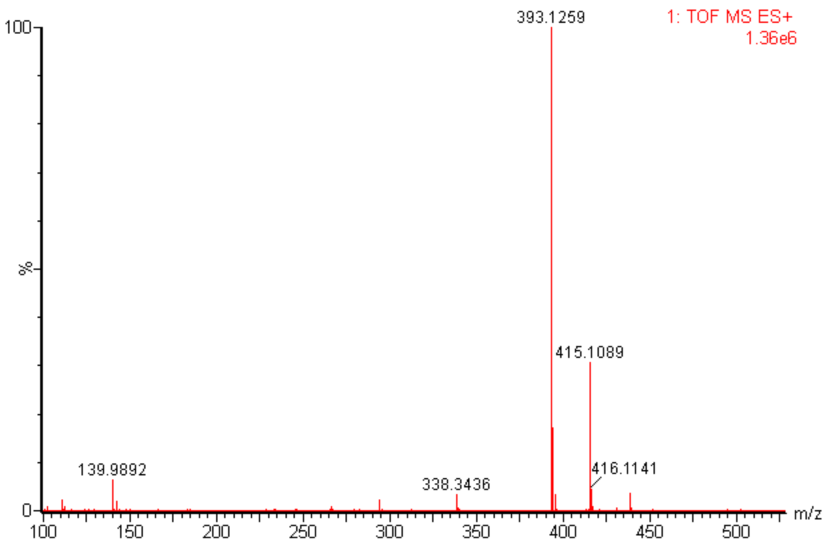

^{1}H NMR, ^{13}C NMR and HR-MS Spectra of compound 4

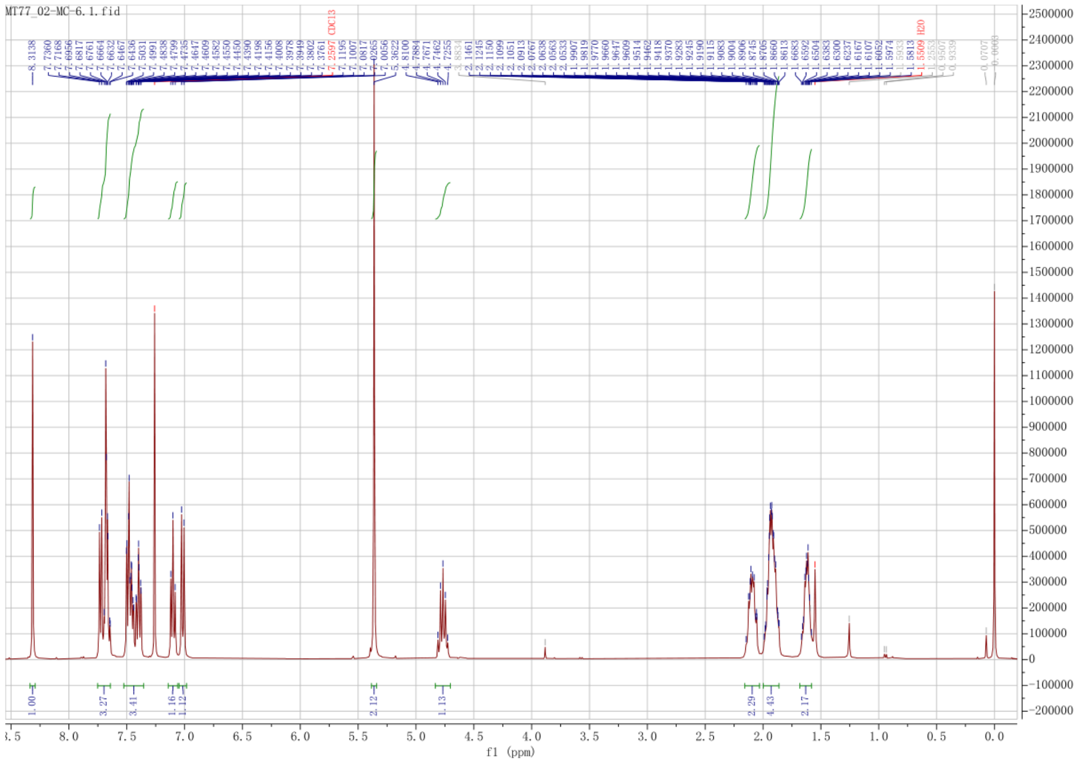

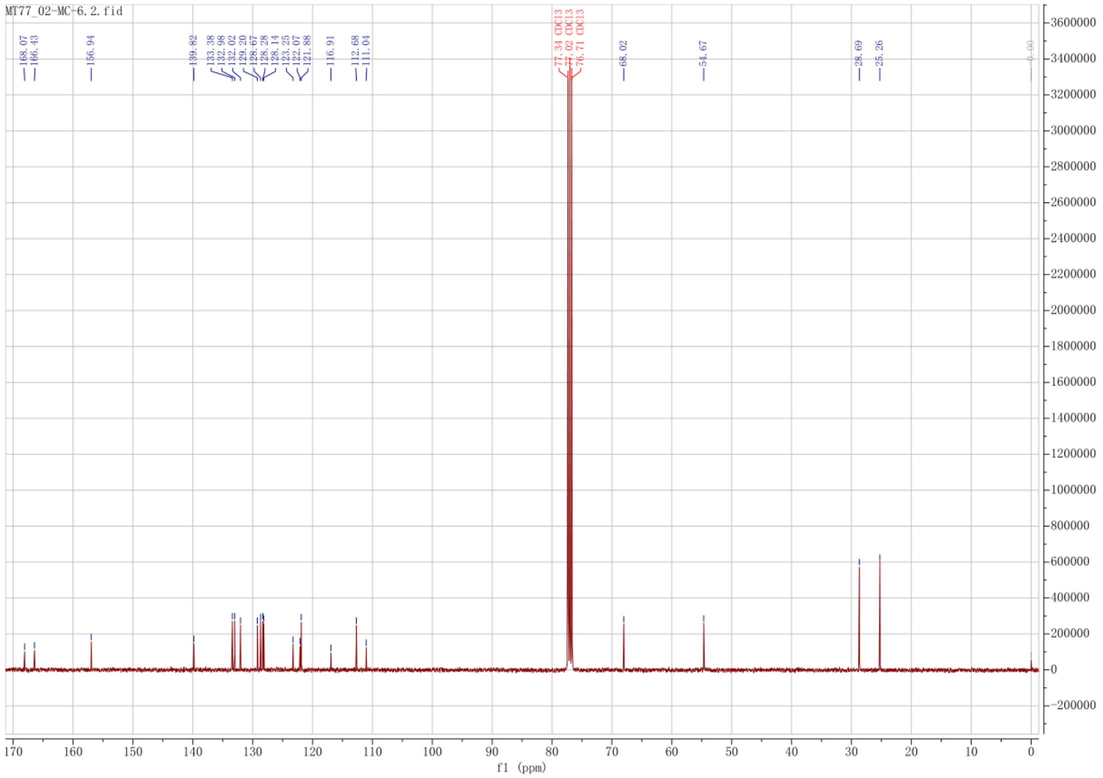

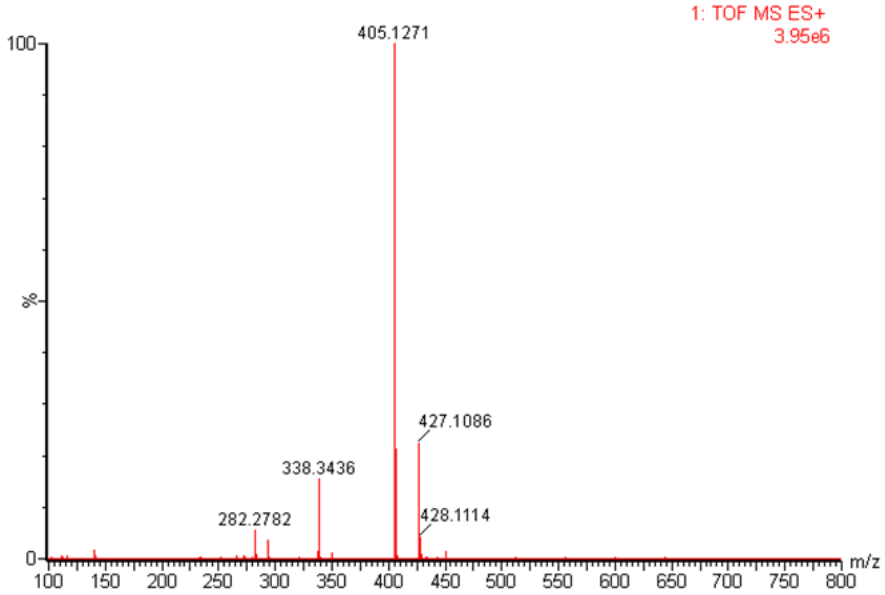

^{1}H NMR, ^{13}C NMR and HR-MS Spectra of compound 5

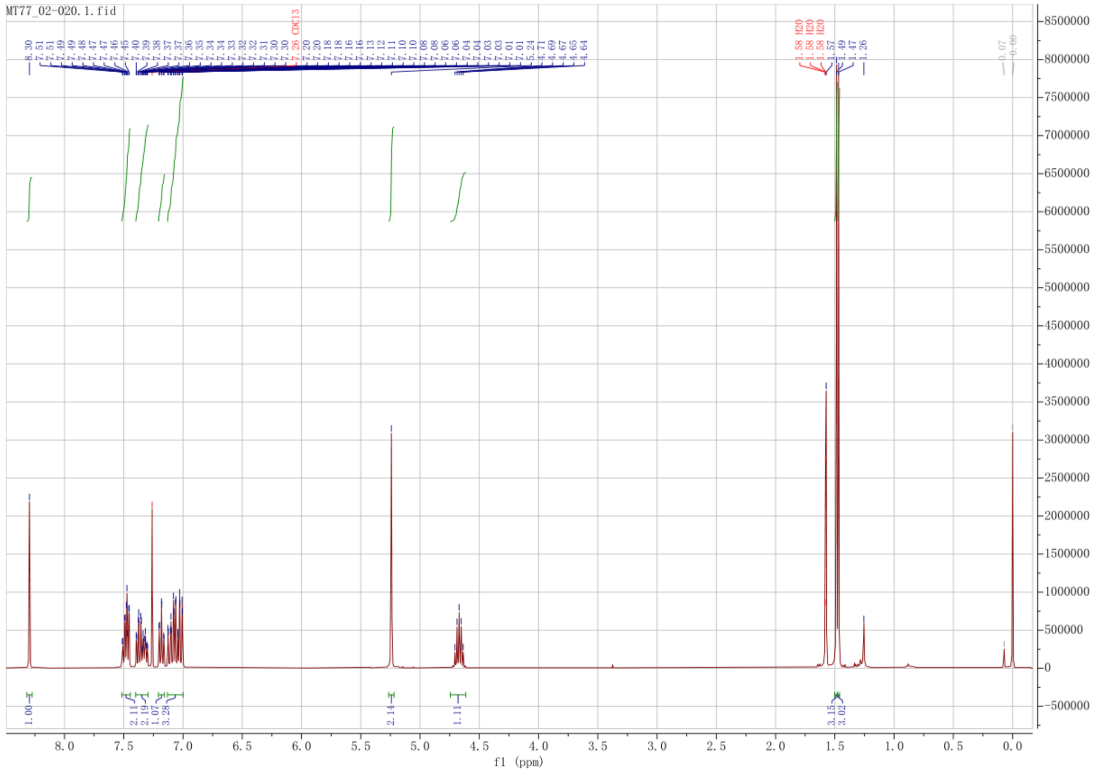
MT77_02-020.1.fid
f1 (ppm)

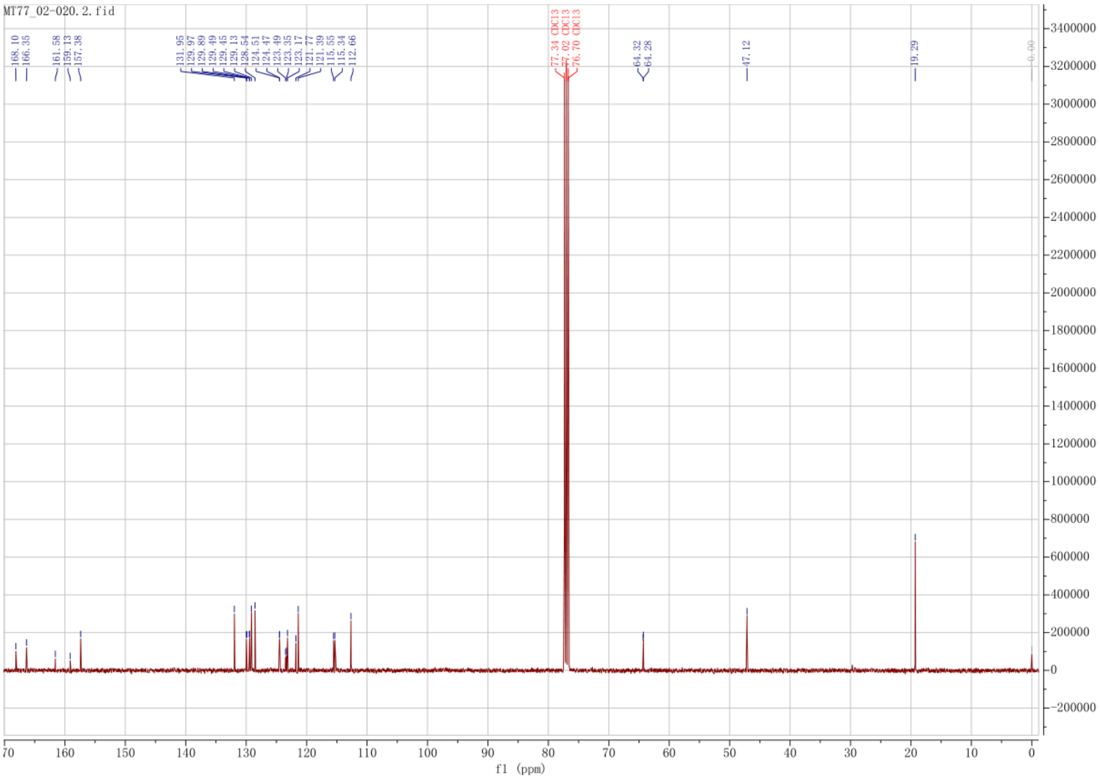
MT77_02-020.2.fid
f1 (ppm)

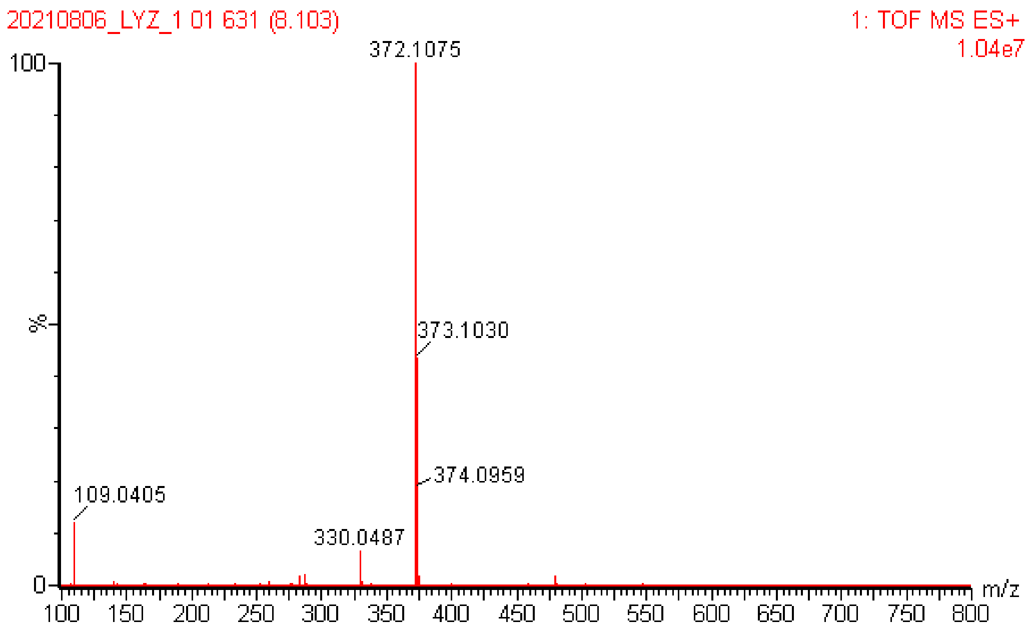

^{1}H NMR, ^{13}C NMR and HR-MS Spectra of compound 6

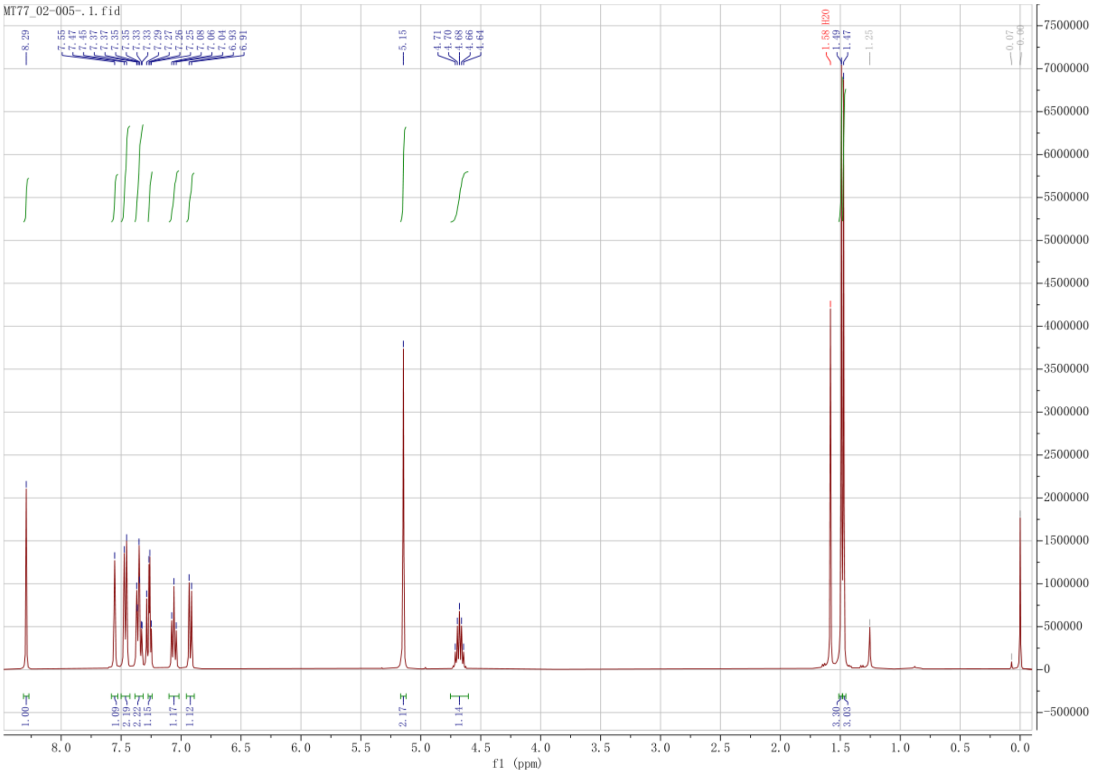

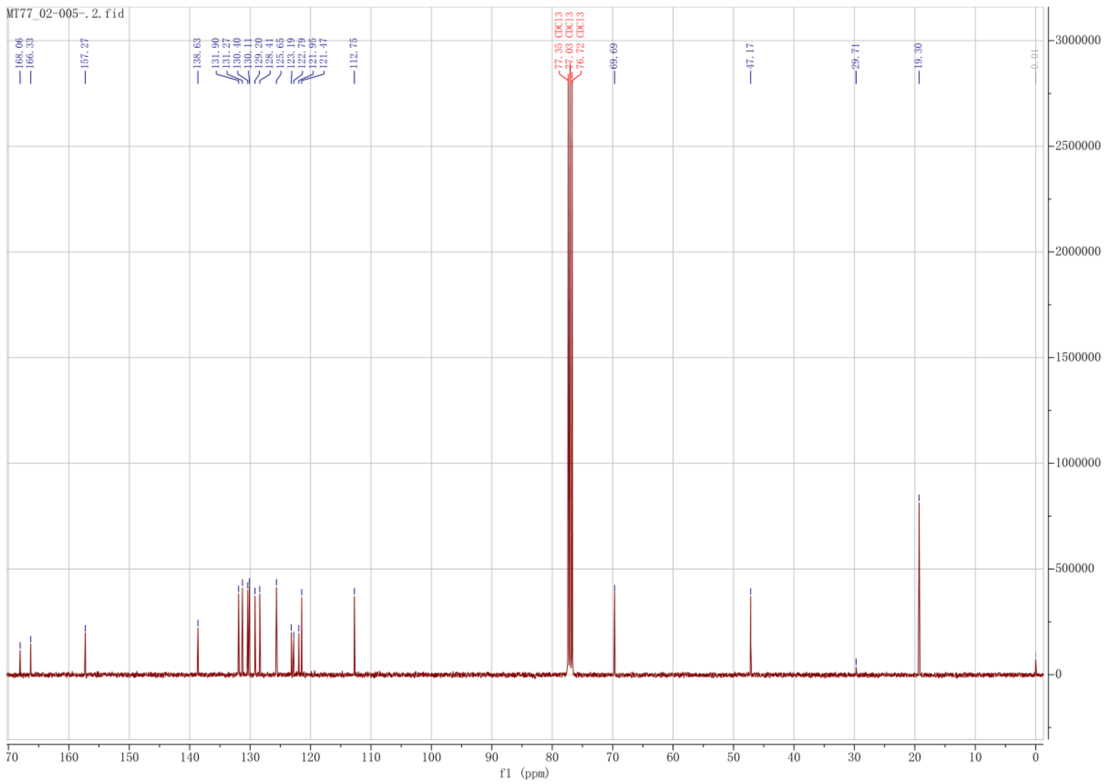

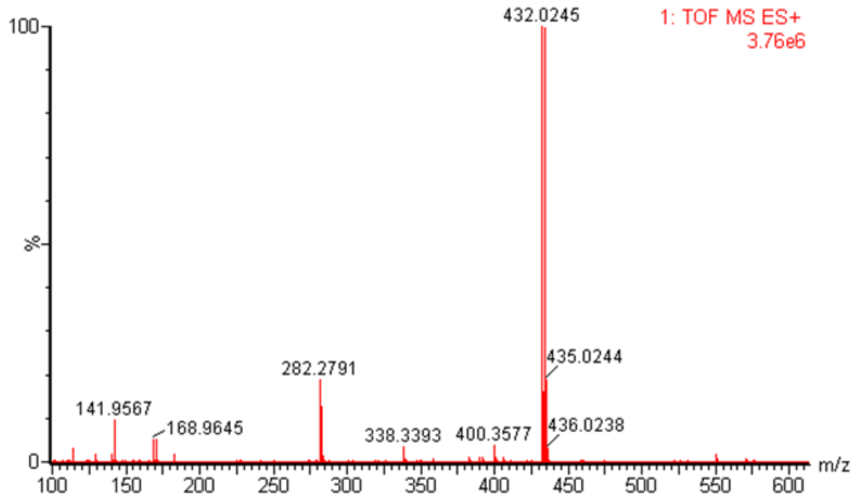

^{1}H NMR, ^{13}C NMR and HR-MS Spectra of compound 7

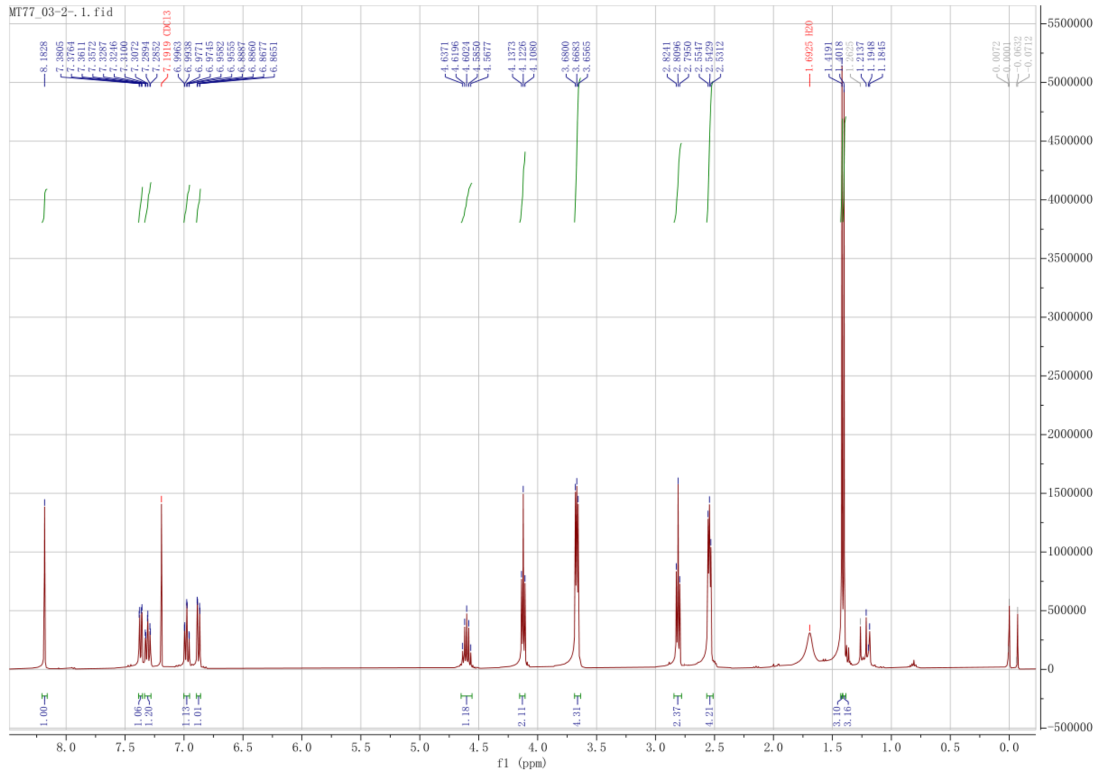
MT77_03-2-.1.fid
f1 (ppm)

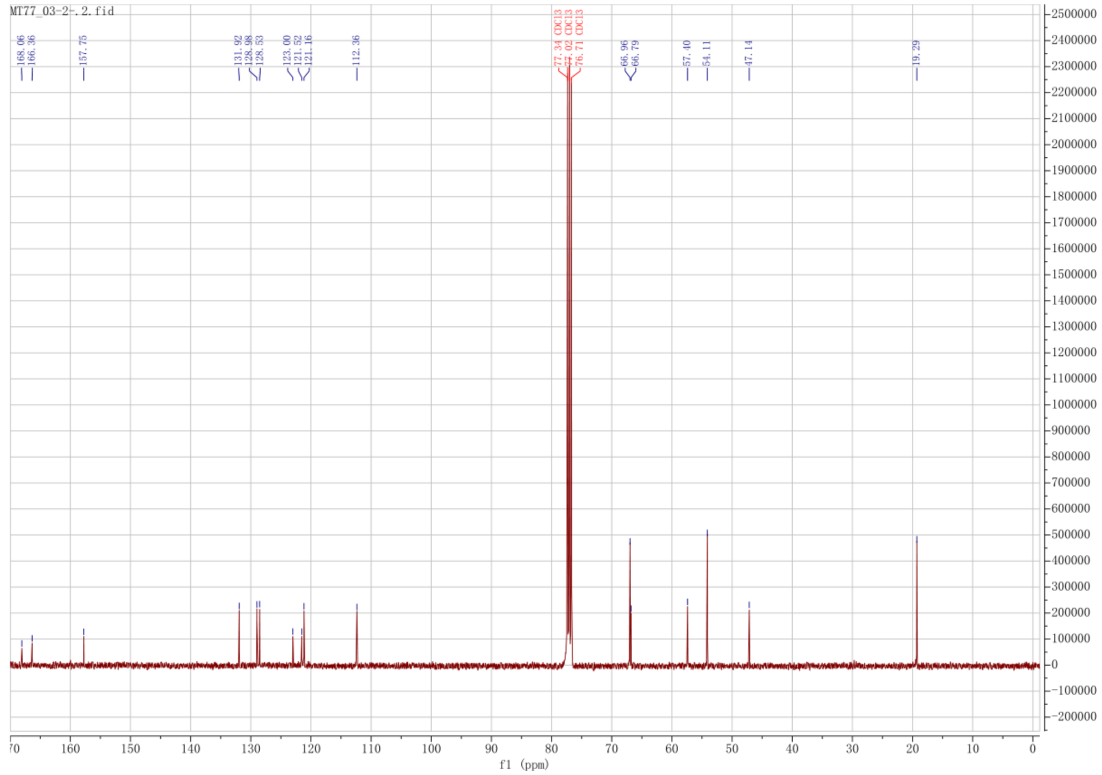
MT77_03-2-.2.fid
f1 (ppm)

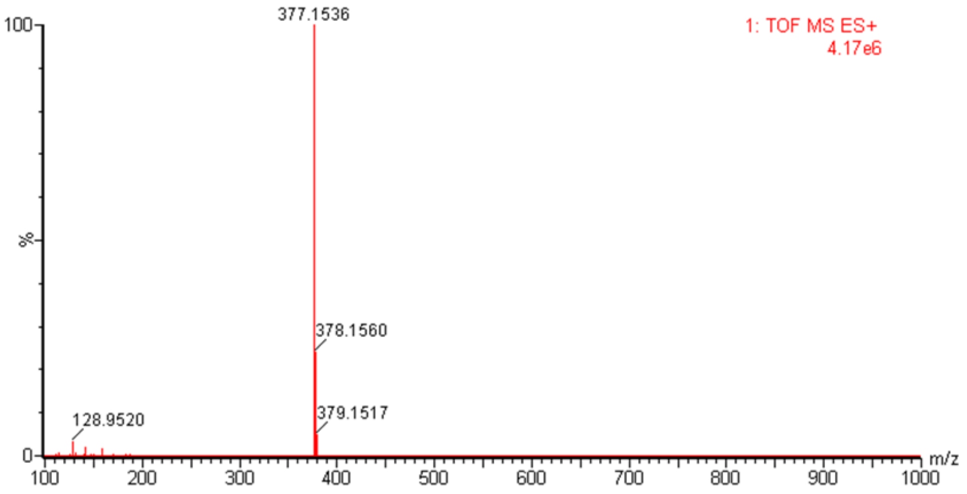

^{1}H NMR, ^{13}C NMR and HR-MS Spectra of compound 8

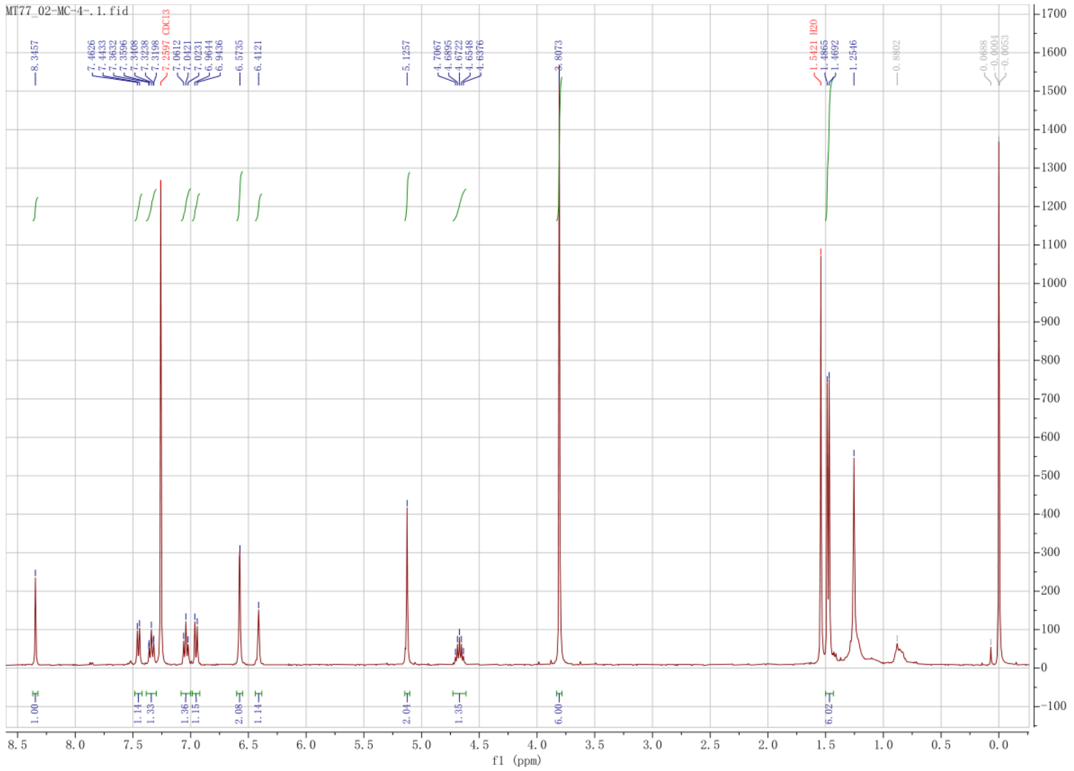

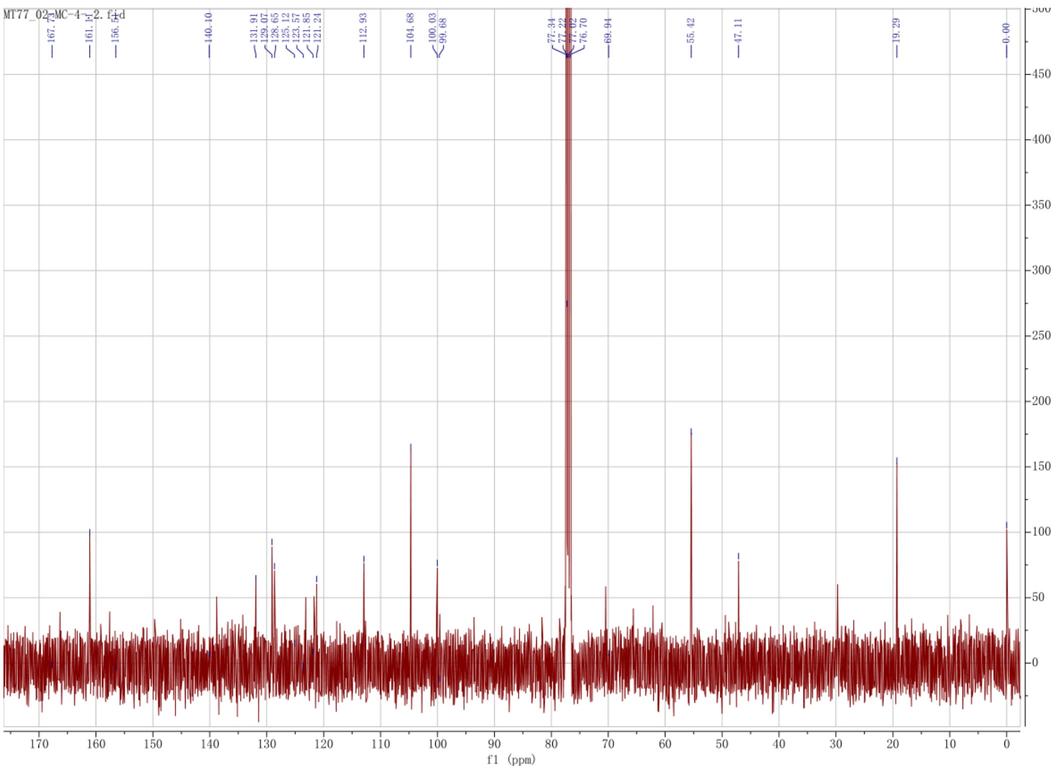

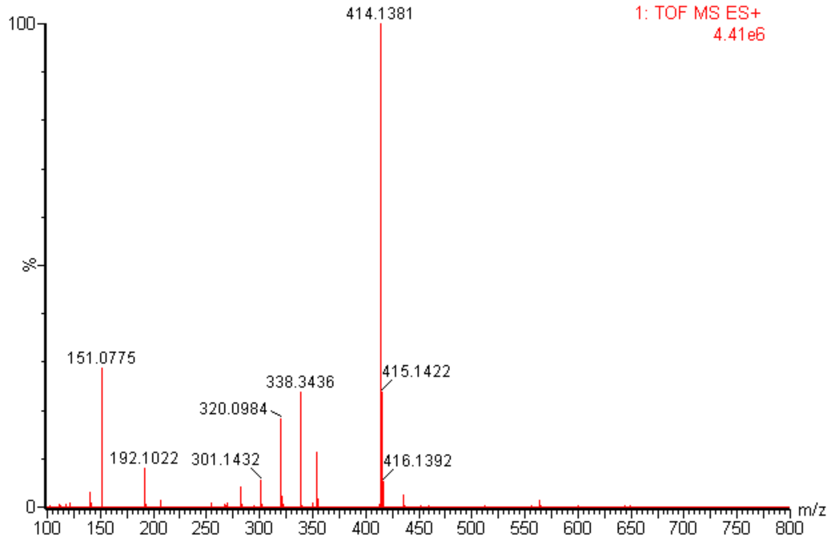

^{1}H NMR, ^{13}C NMR and HR-MS Spectra of compound 9

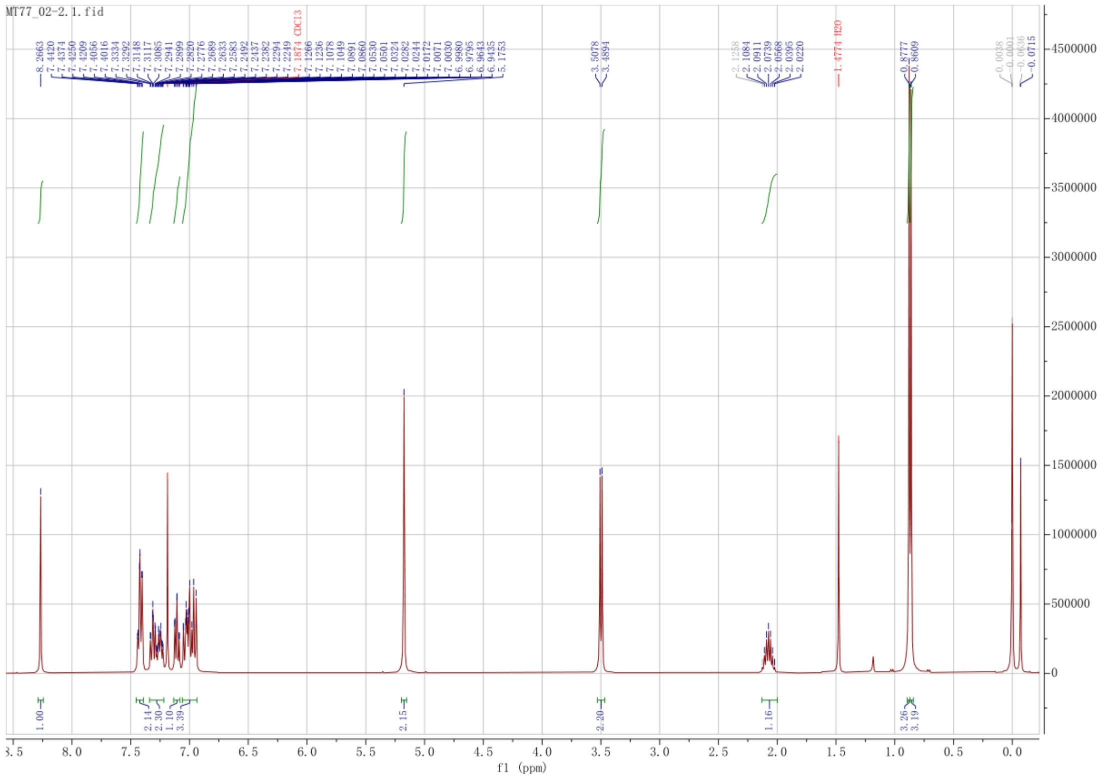
MT77_02-2.1.fid
f1 (ppm)

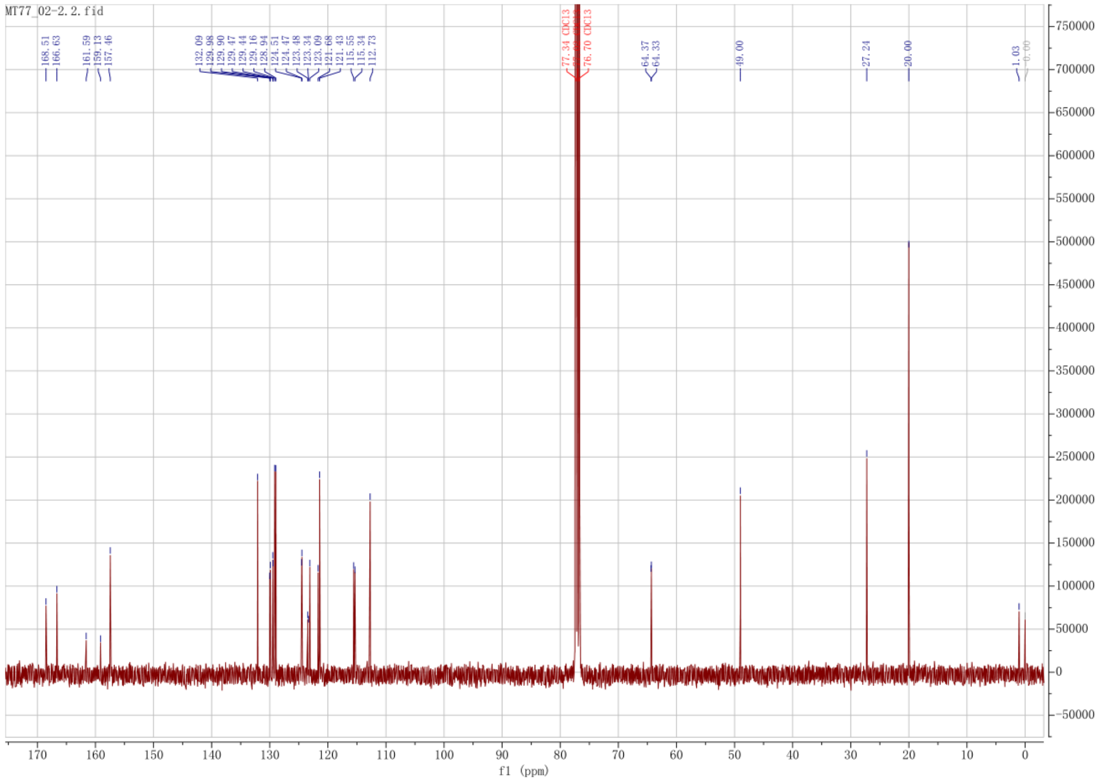
MT77_02-2.2.fid
f1 (ppm)

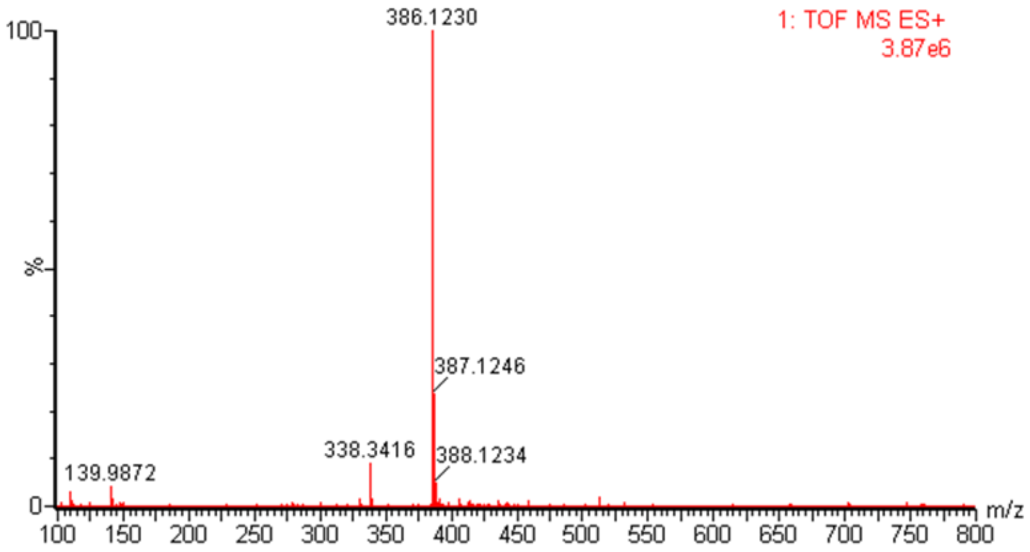

^{1}H NMR, ^{13}C NMR and HR-MS Spectra of compound 10

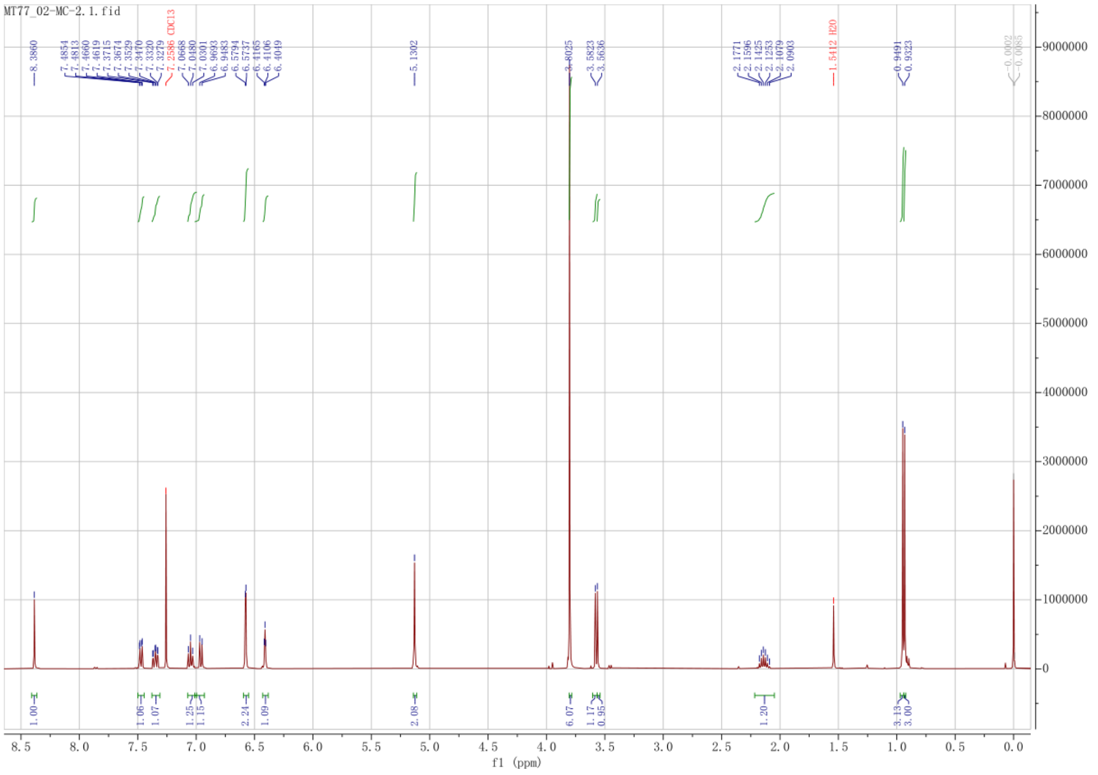

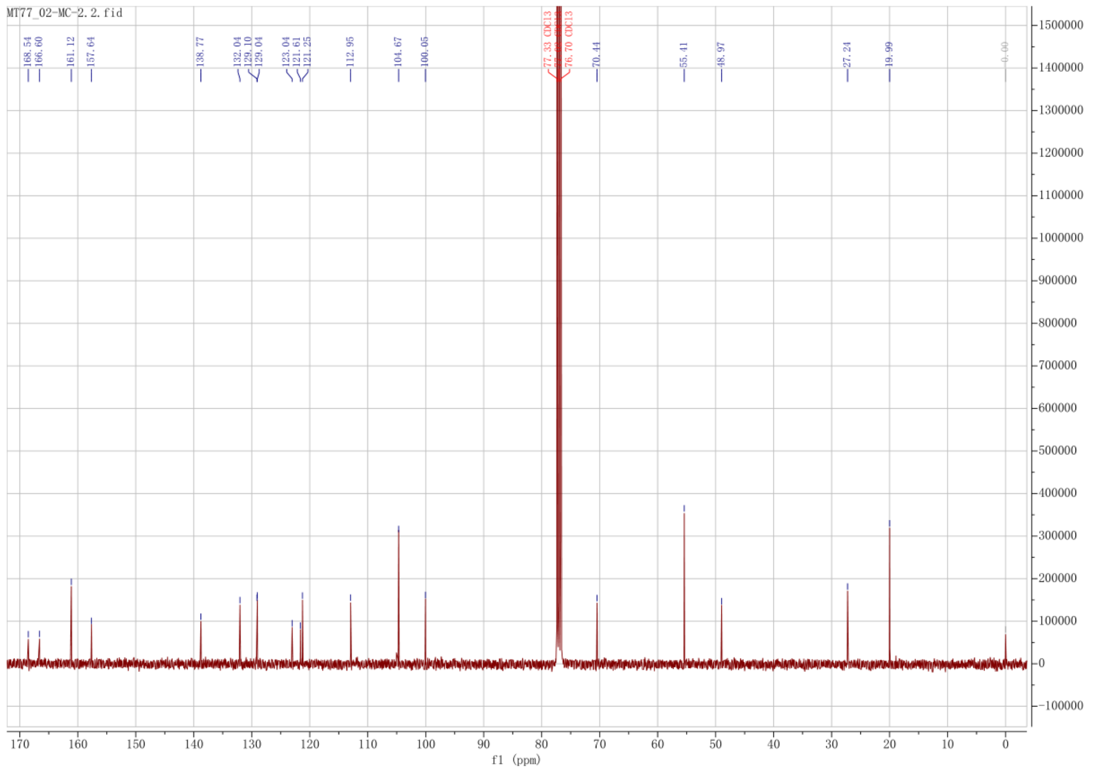

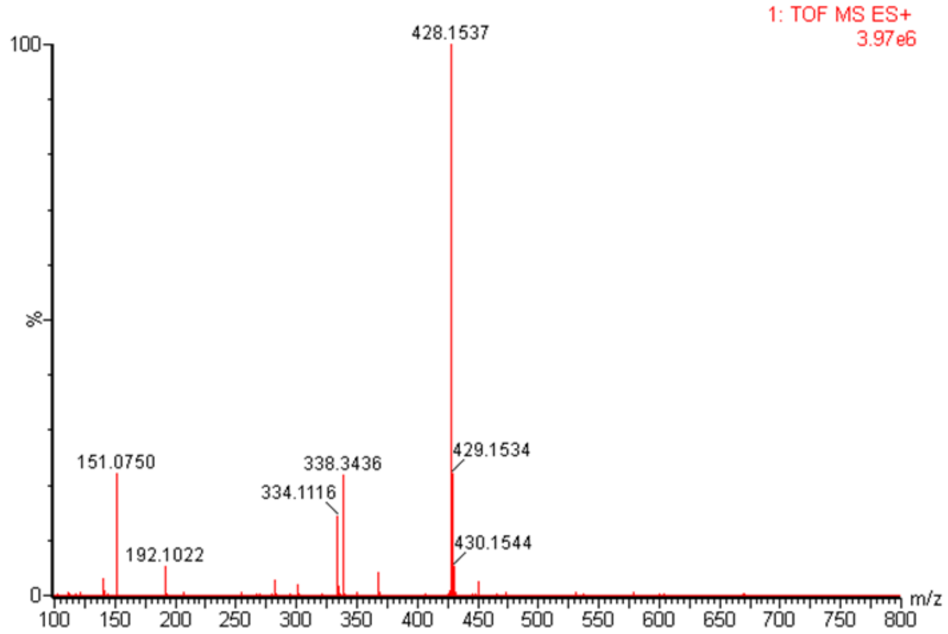

^{1}H NMR, ^{13}C NMR and HR-MS Spectra of compound 11

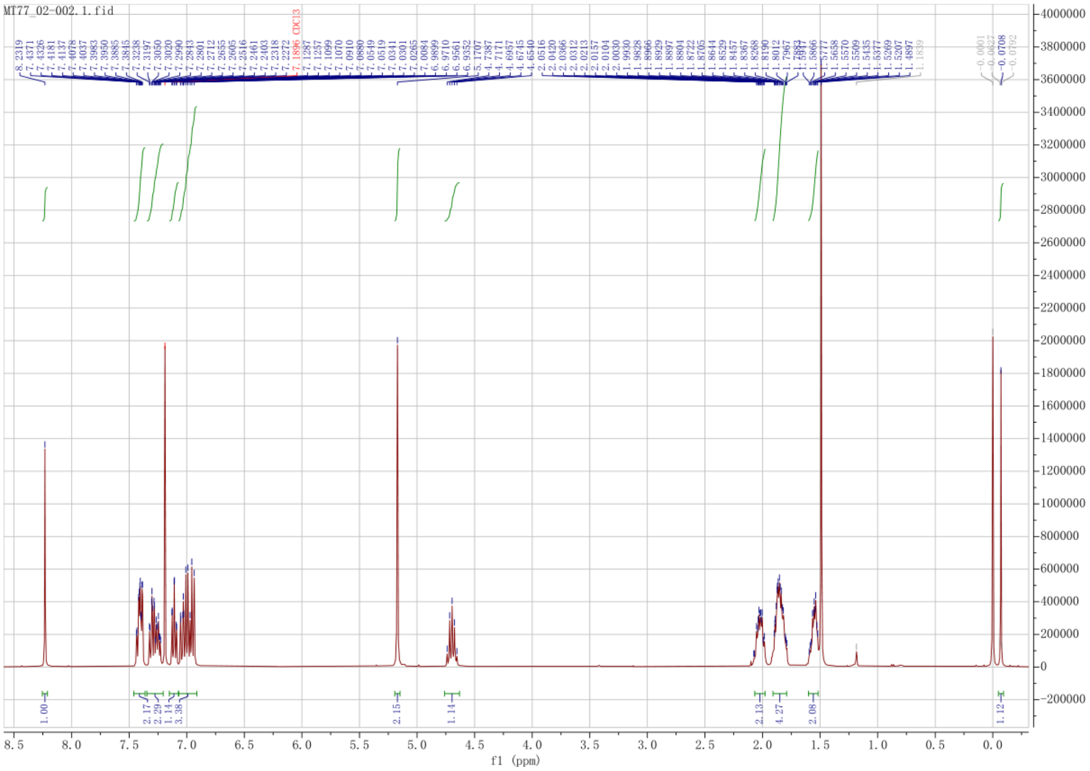

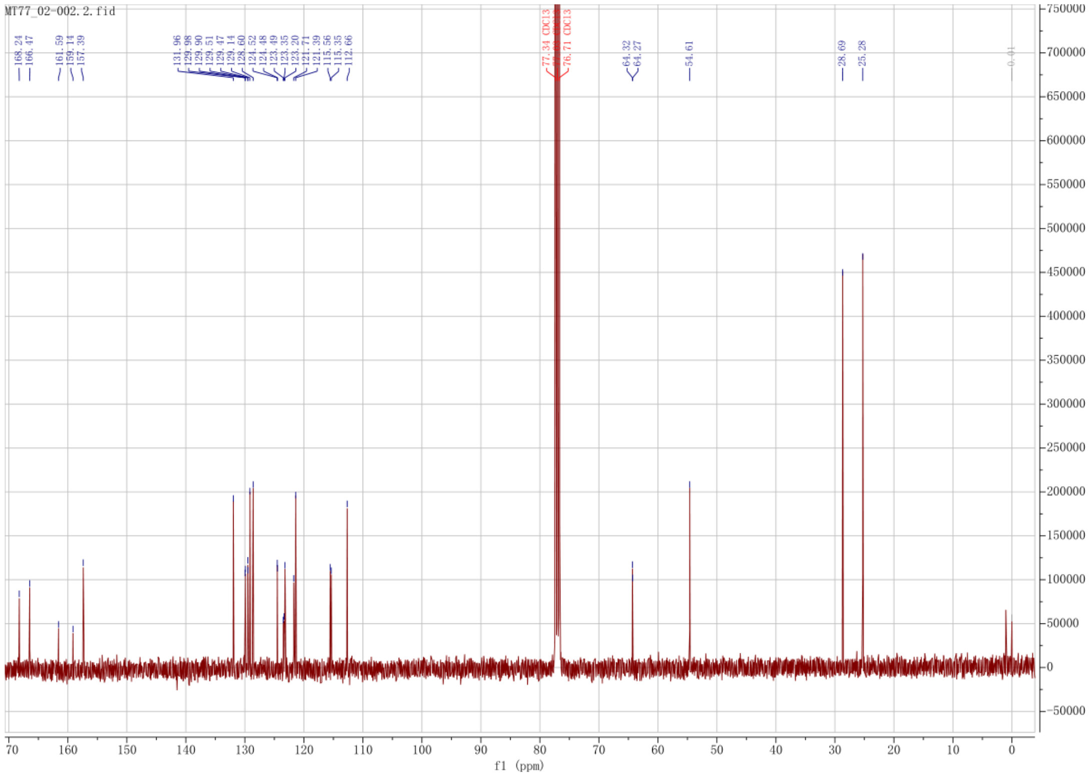

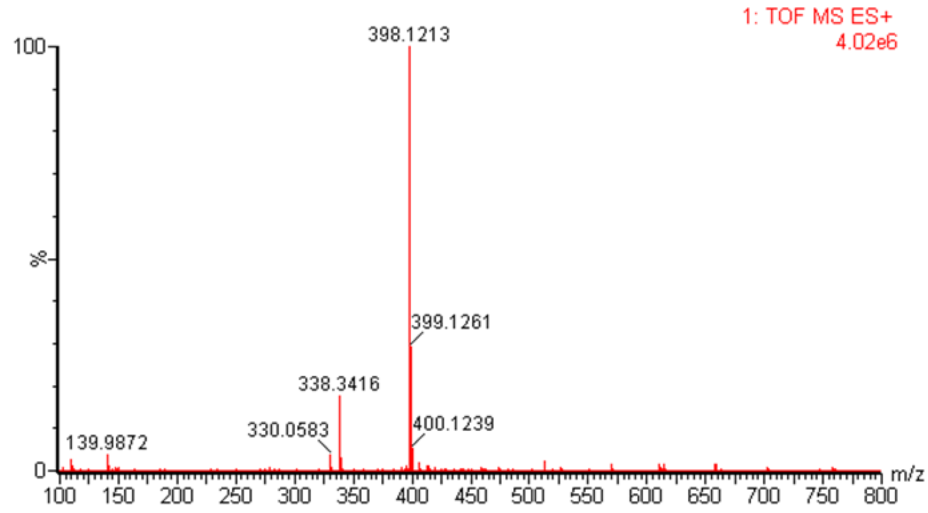

^{1}H NMR, ^{13}C NMR and HR-MS Spectra of compound 12

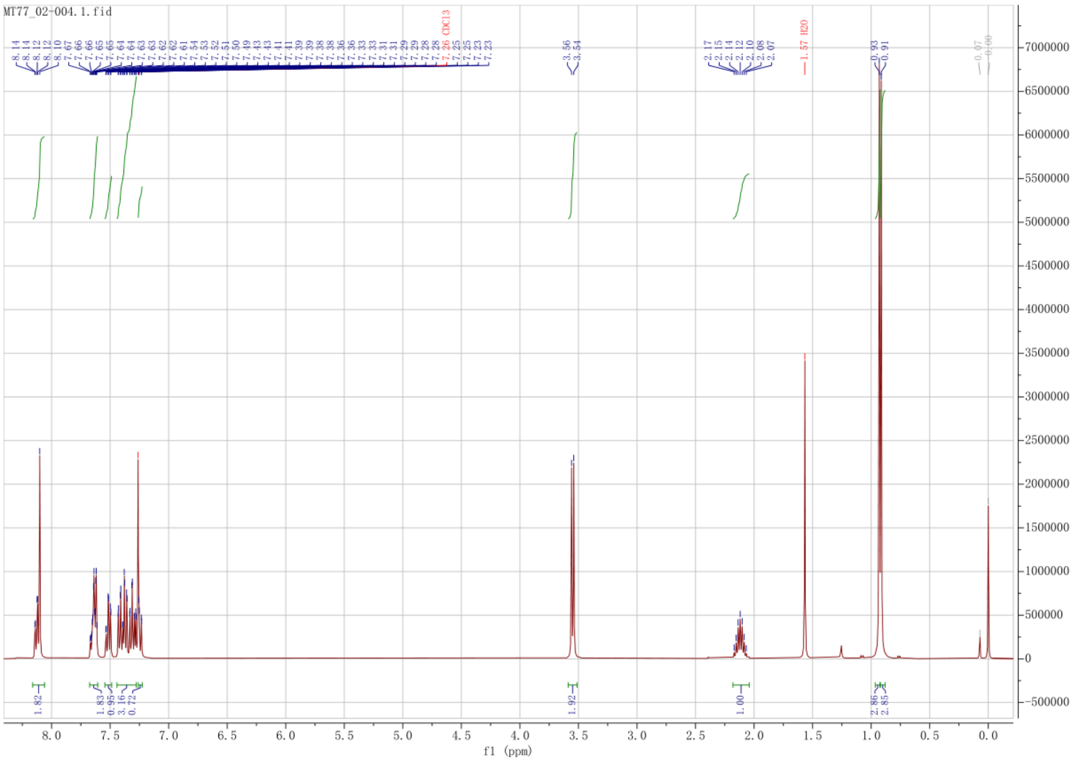

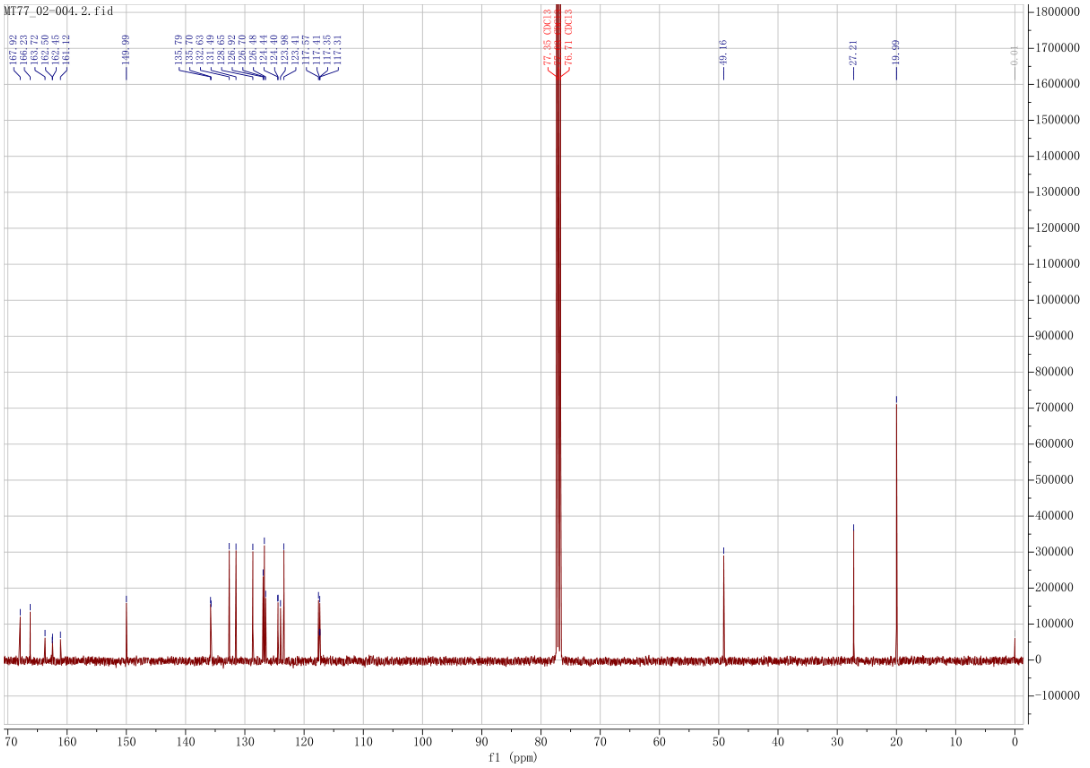

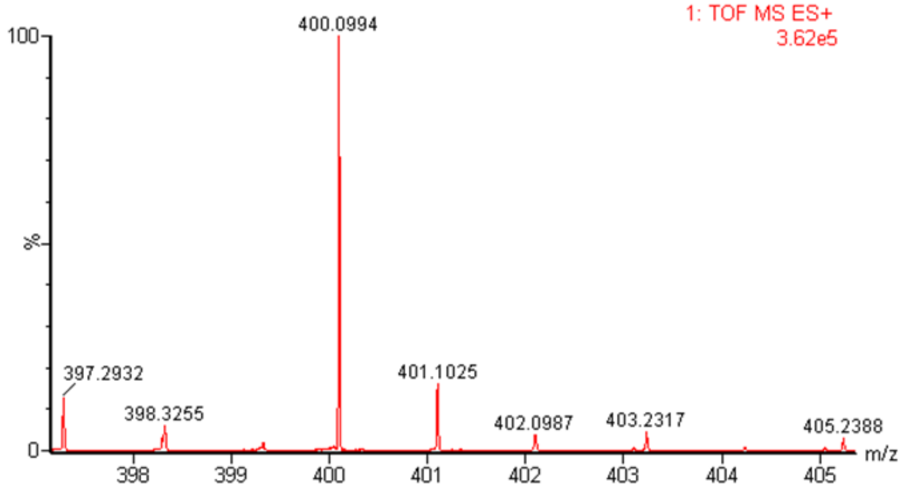

^{1}H NMR, ^{13}C NMR and HR-MS Spectra of compound 13

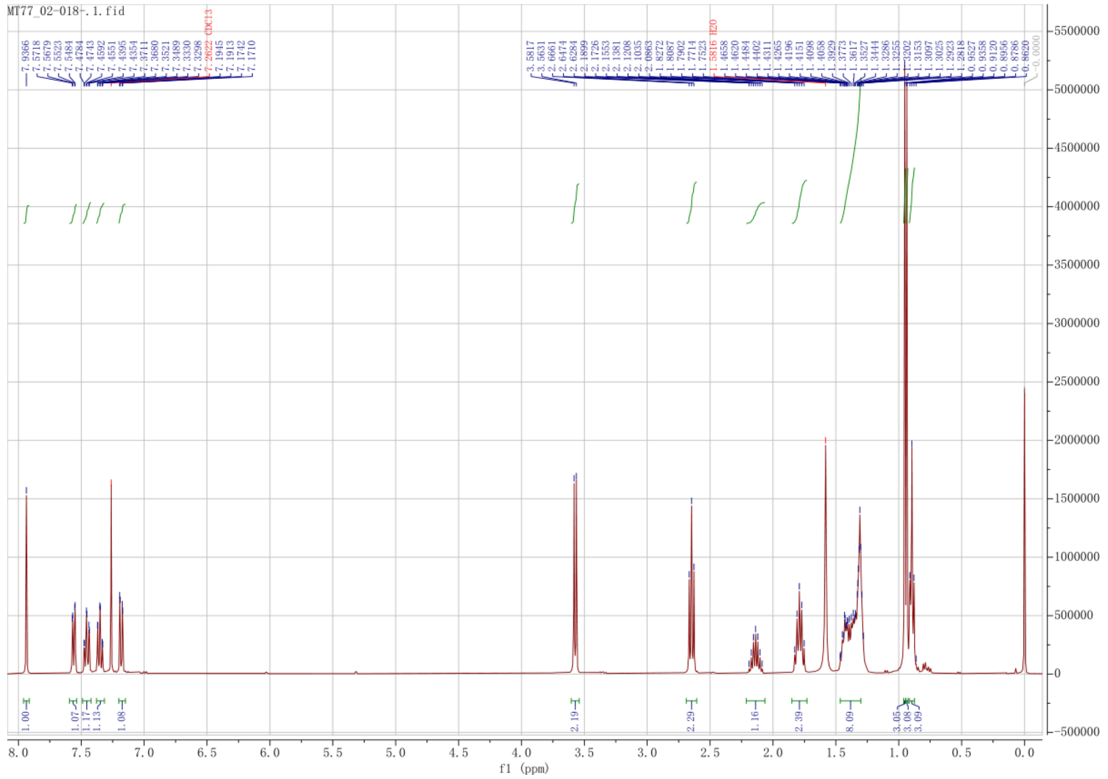
MT77_02-018-.1.fid
f1 (ppm)

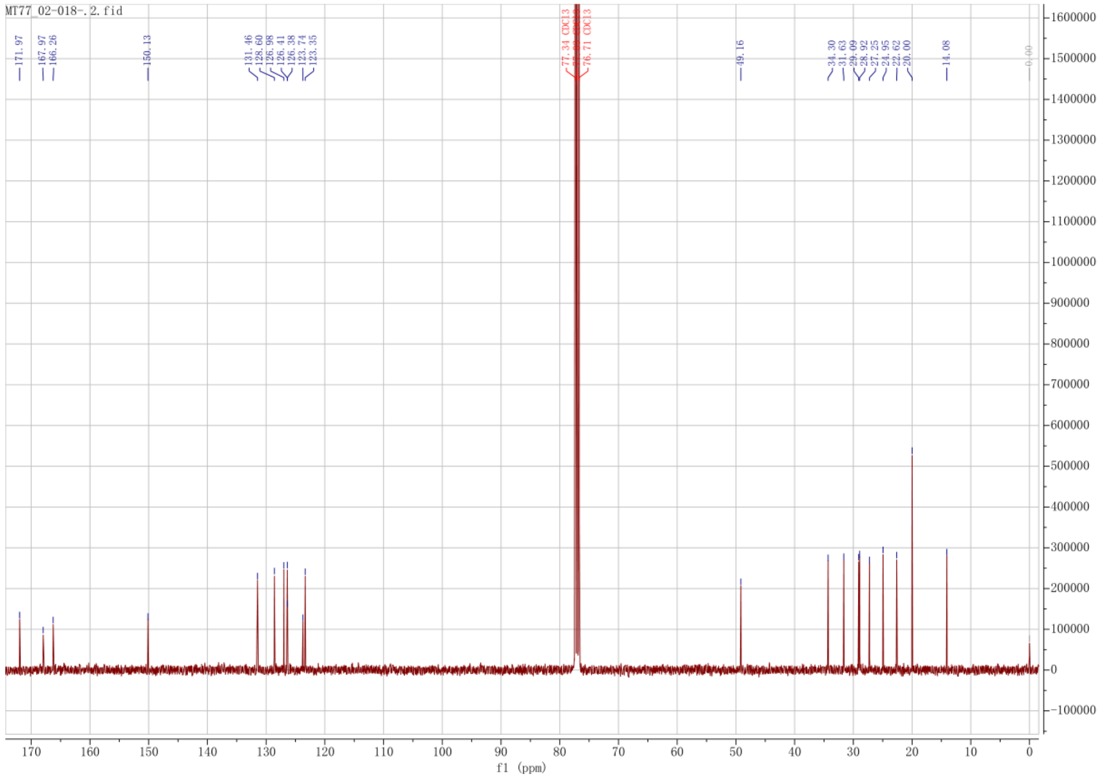
MT77_02-018-.2.fid
171.97
167.97
166.26
150.13
131.46
128.60
126.98
126.41
126.38
123.74
123.35
77.34 CDCl3
76.71 CDCl3
49.16
34.30
31.63
29.09
28.92
27.25
24.95
22.62
20.00
14.08
f1 (ppm)

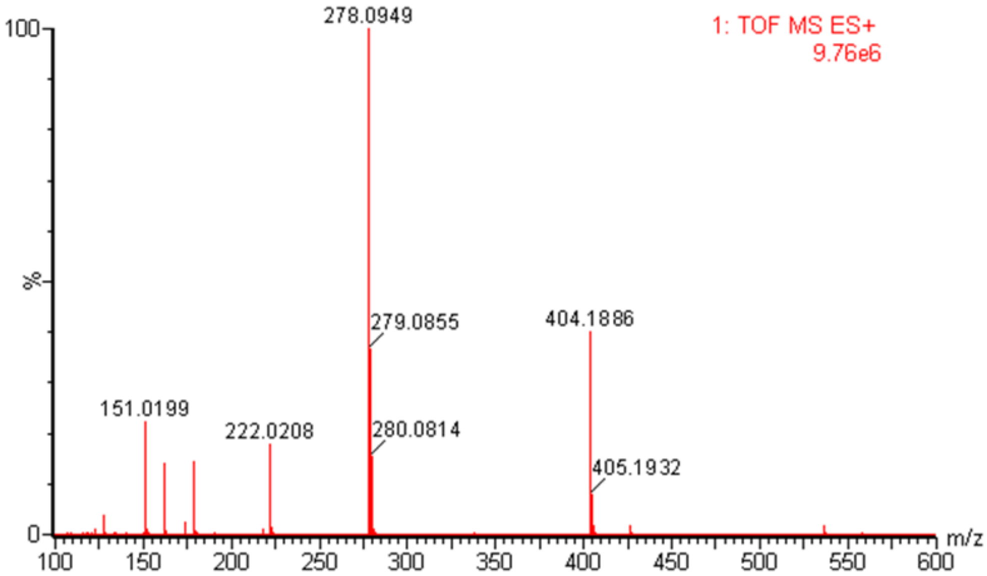

^{1}H NMR, ^{13}C NMR and HR-MS Spectra of compound 14

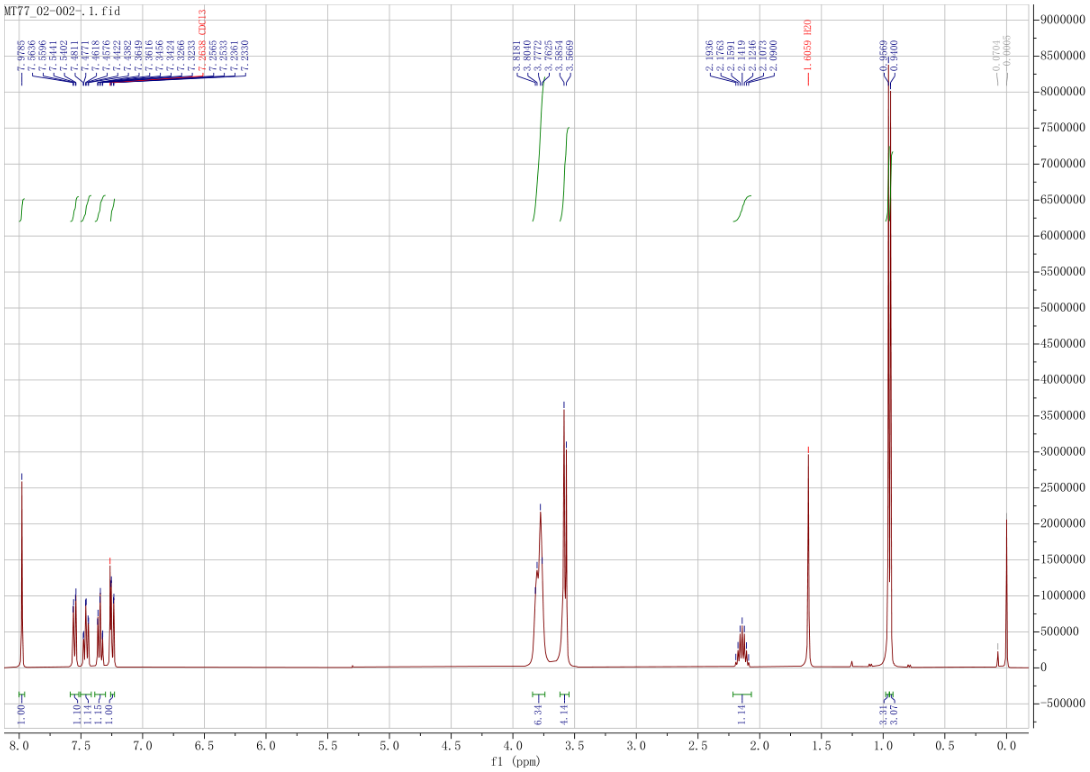

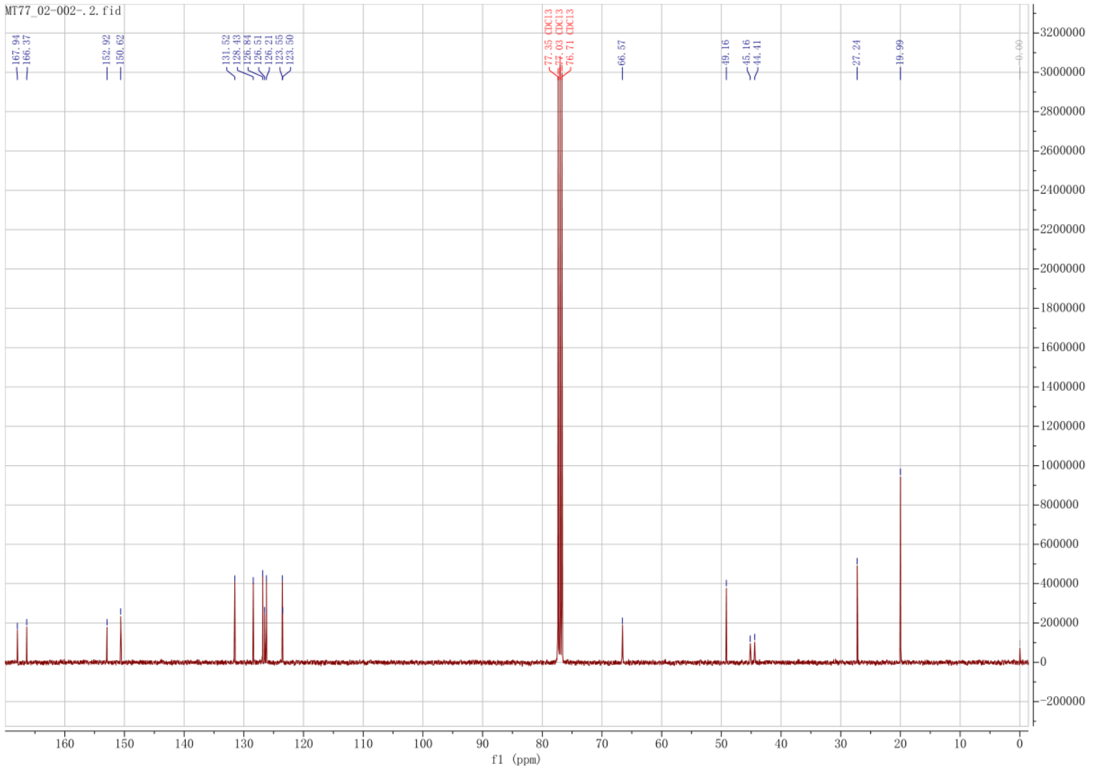

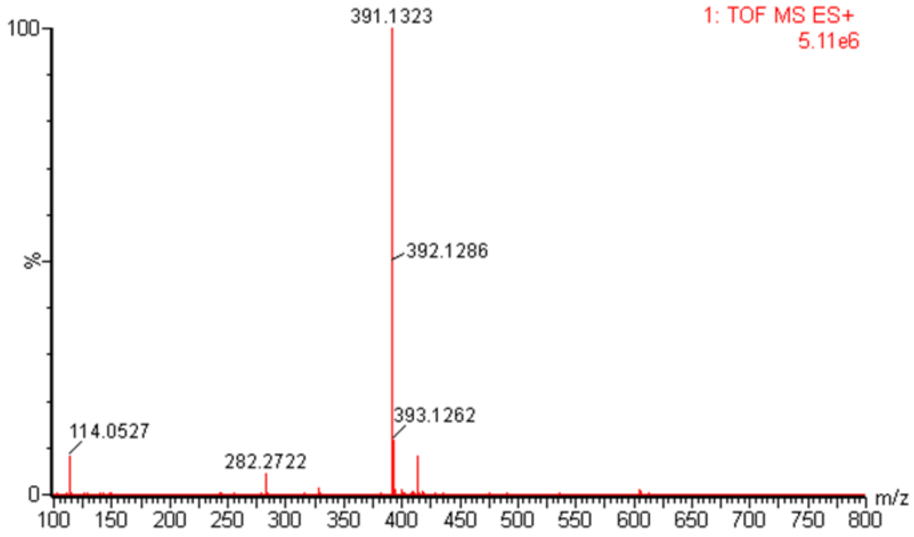

^{1}H NMR, ^{13}C NMR and HR-MS Spectra of compound 15

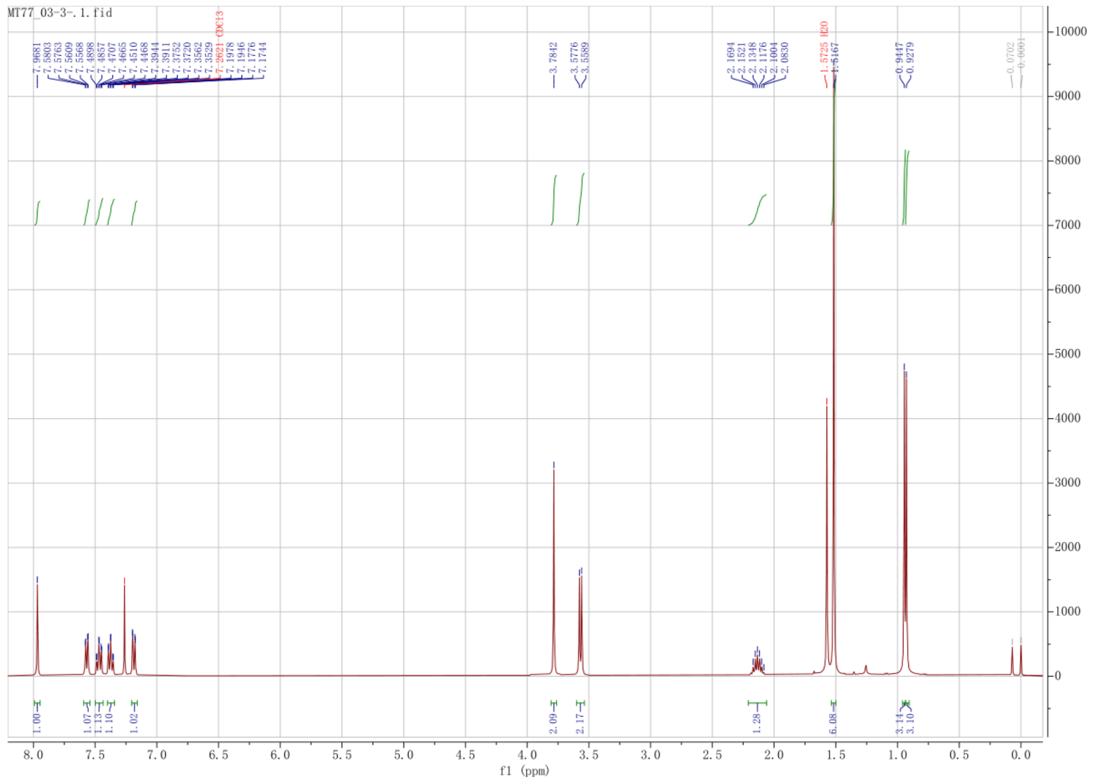
MT77_03-3-.1.fid
f1 (ppm)

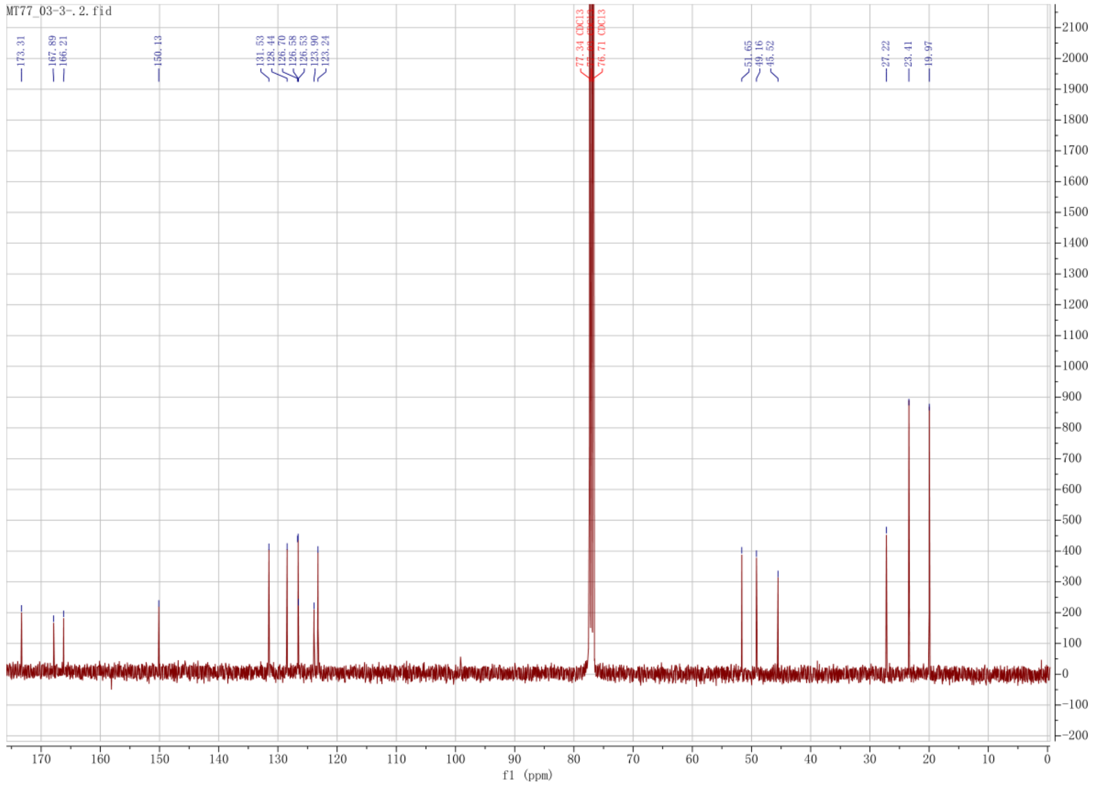
MT77_03-3-.2.fid
173.31
167.89
166.21
150.13
131.53
128.44
126.70
126.58
126.53
123.90
123.24
77.34 CDCl3
76.71 CDCl3
51.65
45.52
27.22
23.41
19.97
f1 (ppm)

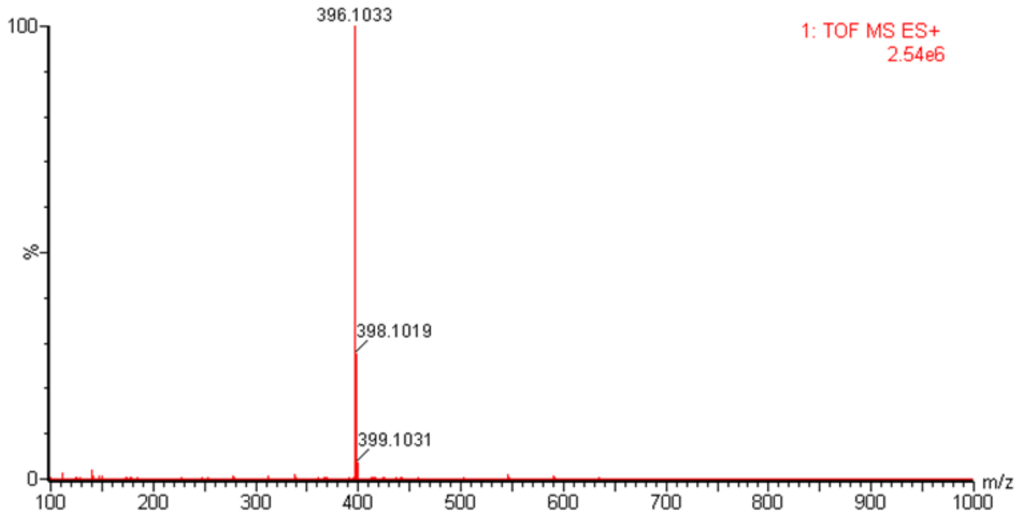

^{1}H NMR, ^{13}C NMR and HR-MS Spectra of compound 16

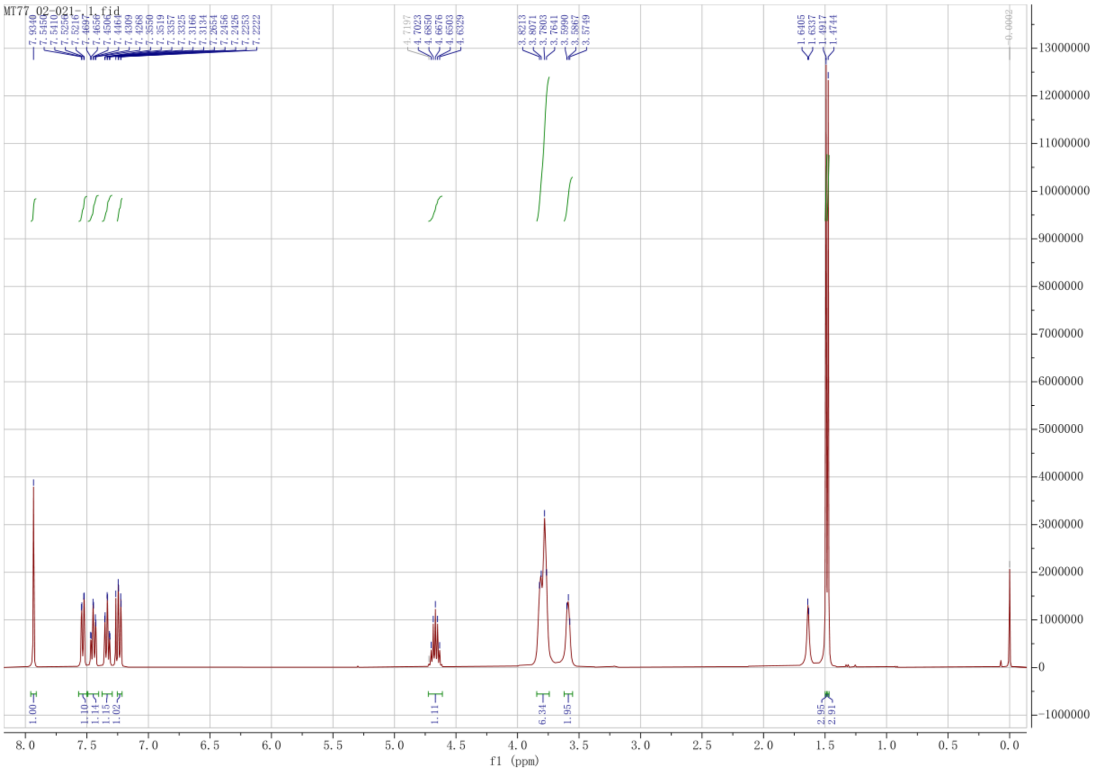

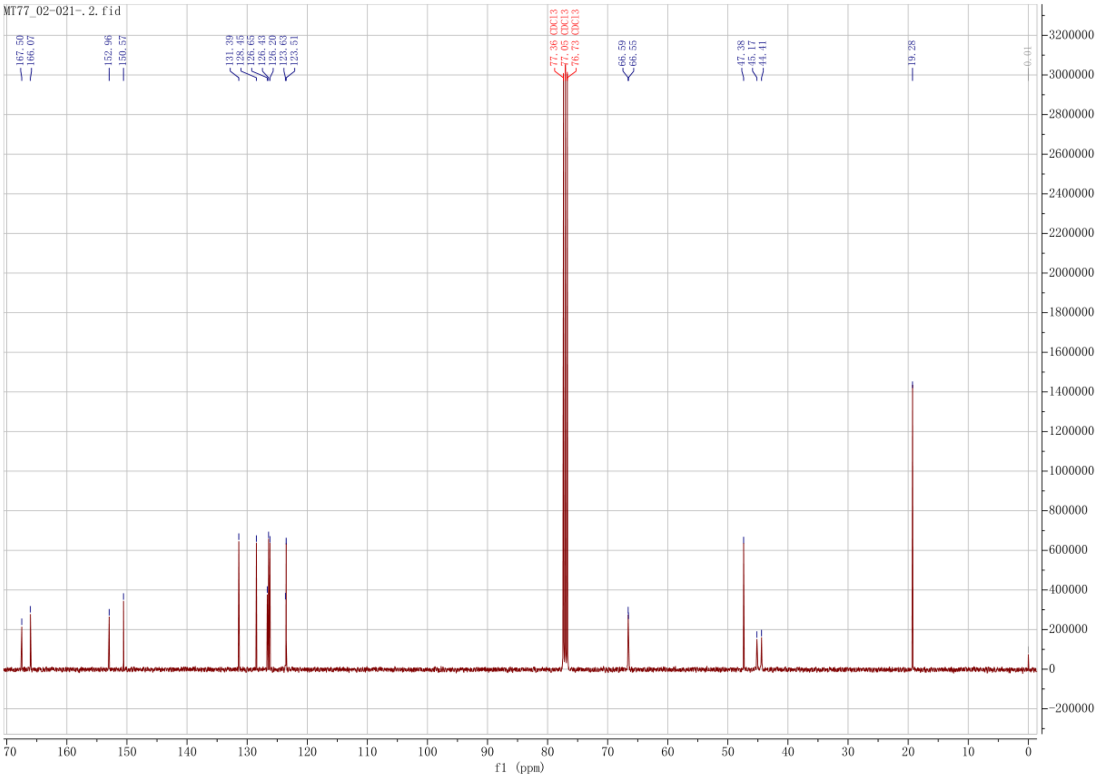

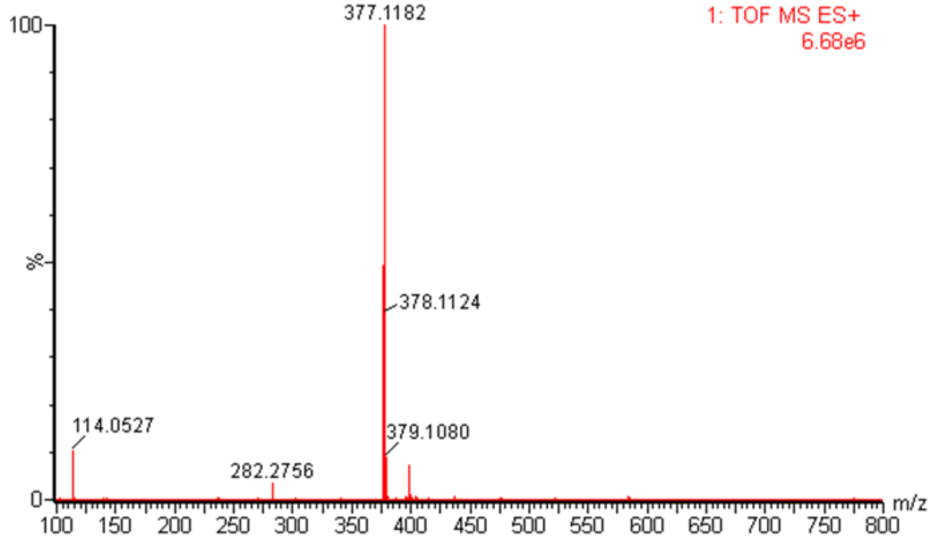

^{1}H NMR, ^{13}C NMR and HR-MS Spectra of compound 17

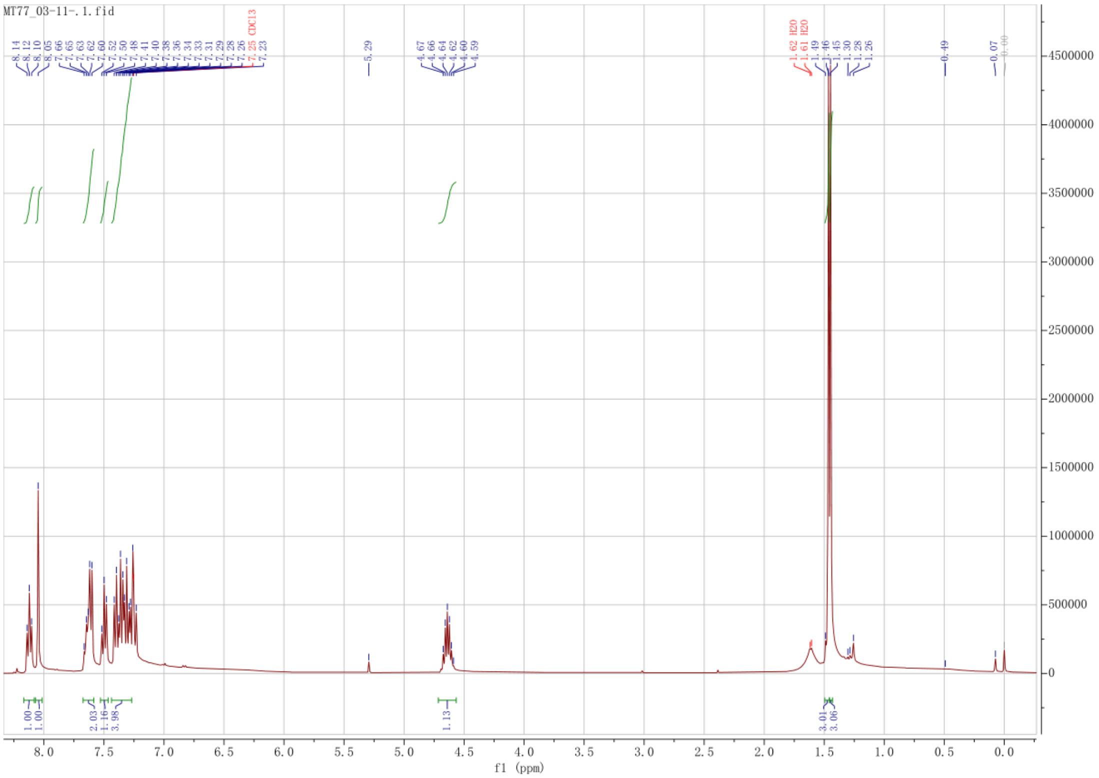
MT77_03-11-.1.fid
f1 (ppm)

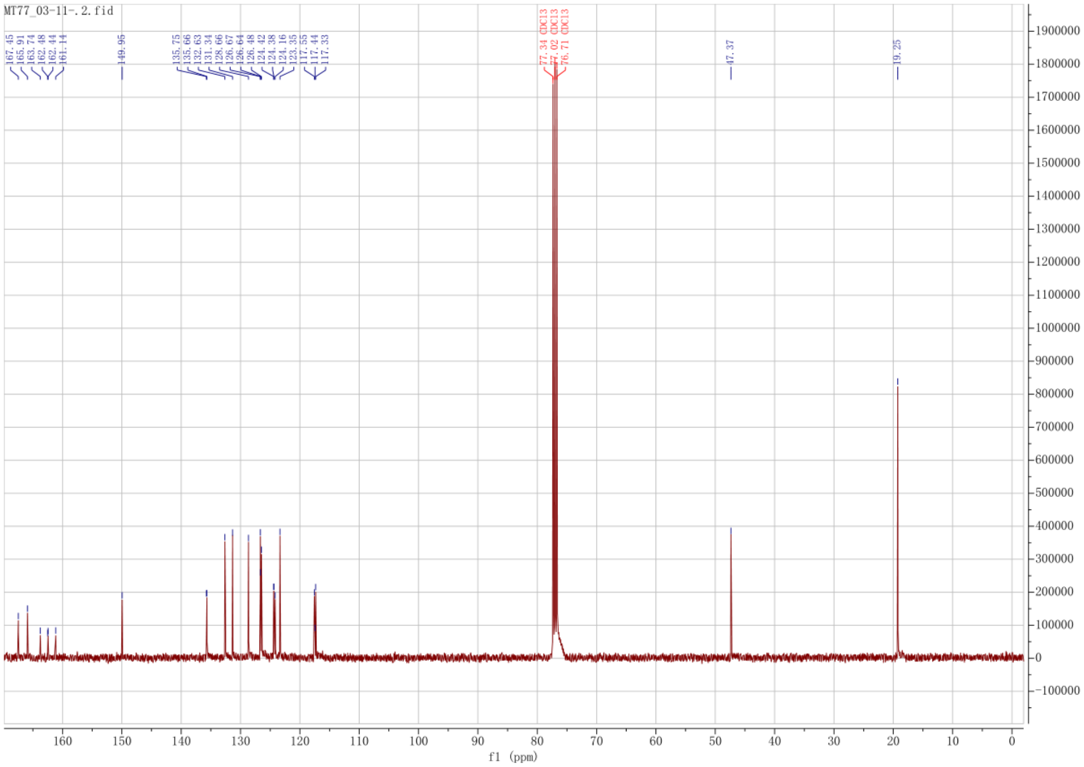
MT77_03-11-.2.fid
f1 (ppm)

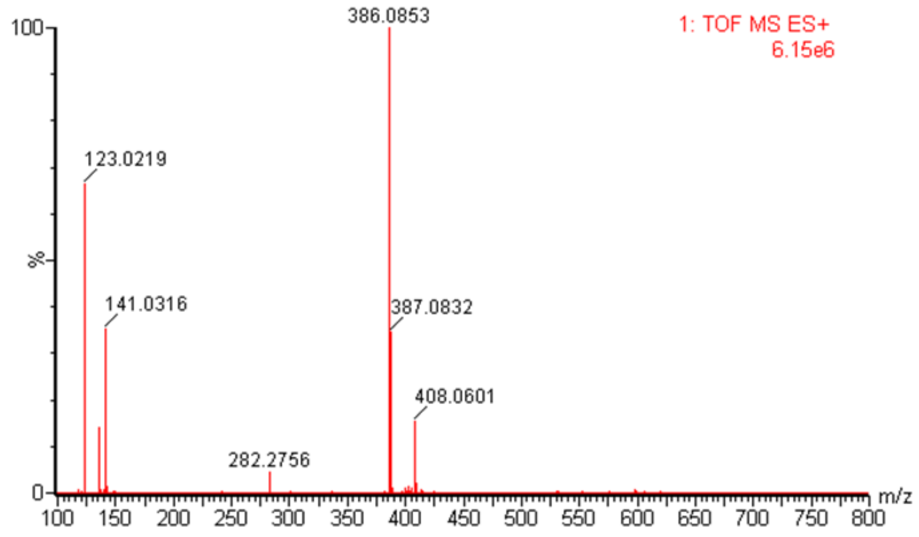

^{1}H NMR, ^{13}C NMR and HR-MS Spectra of compound 18

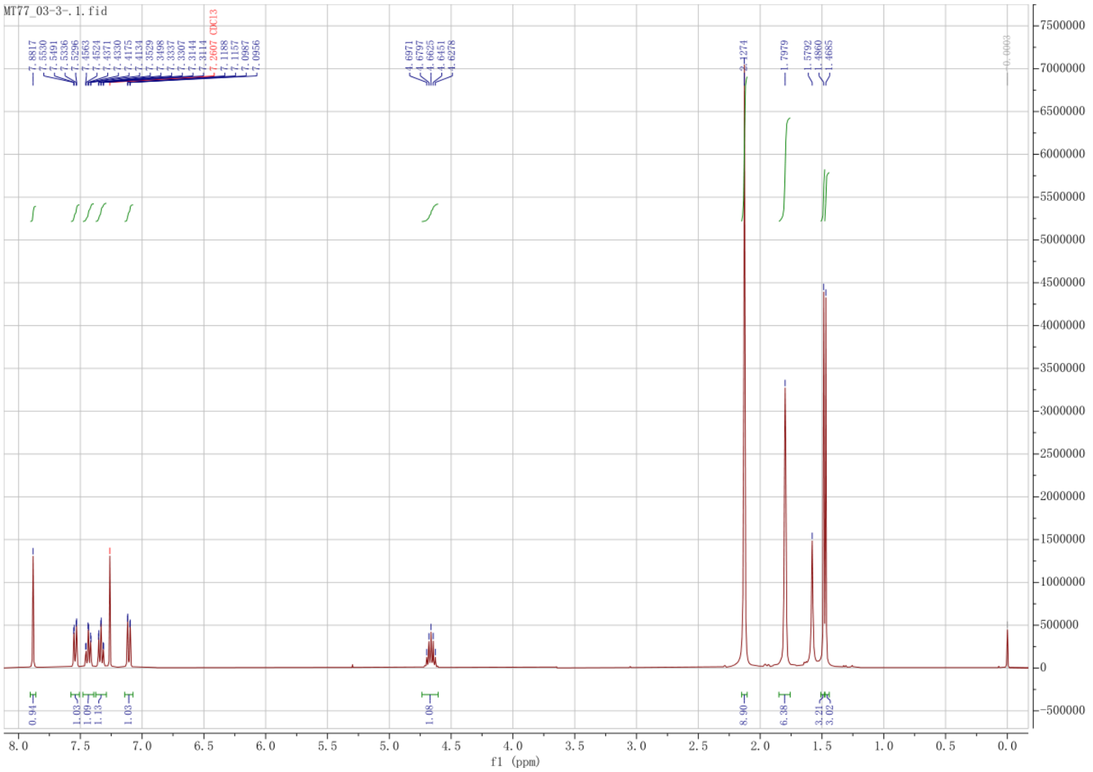

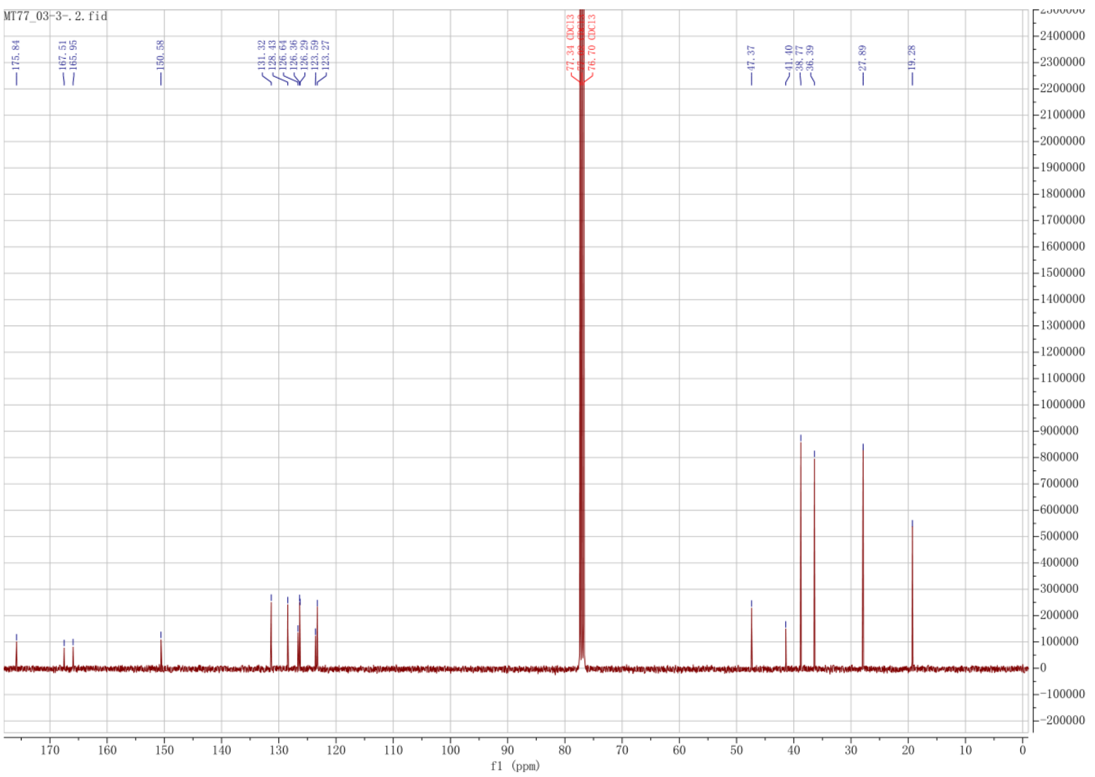

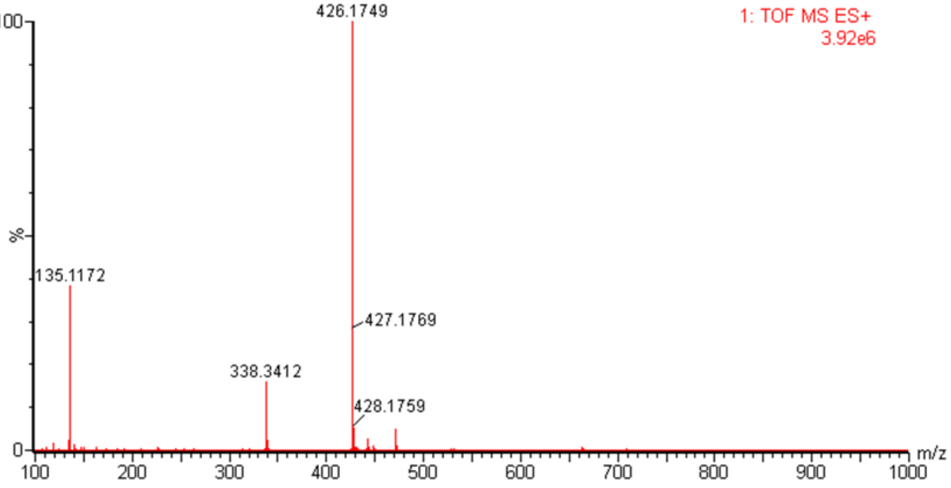

^{1}H NMR, ^{13}C NMR and HR-MS Spectra of compound 19

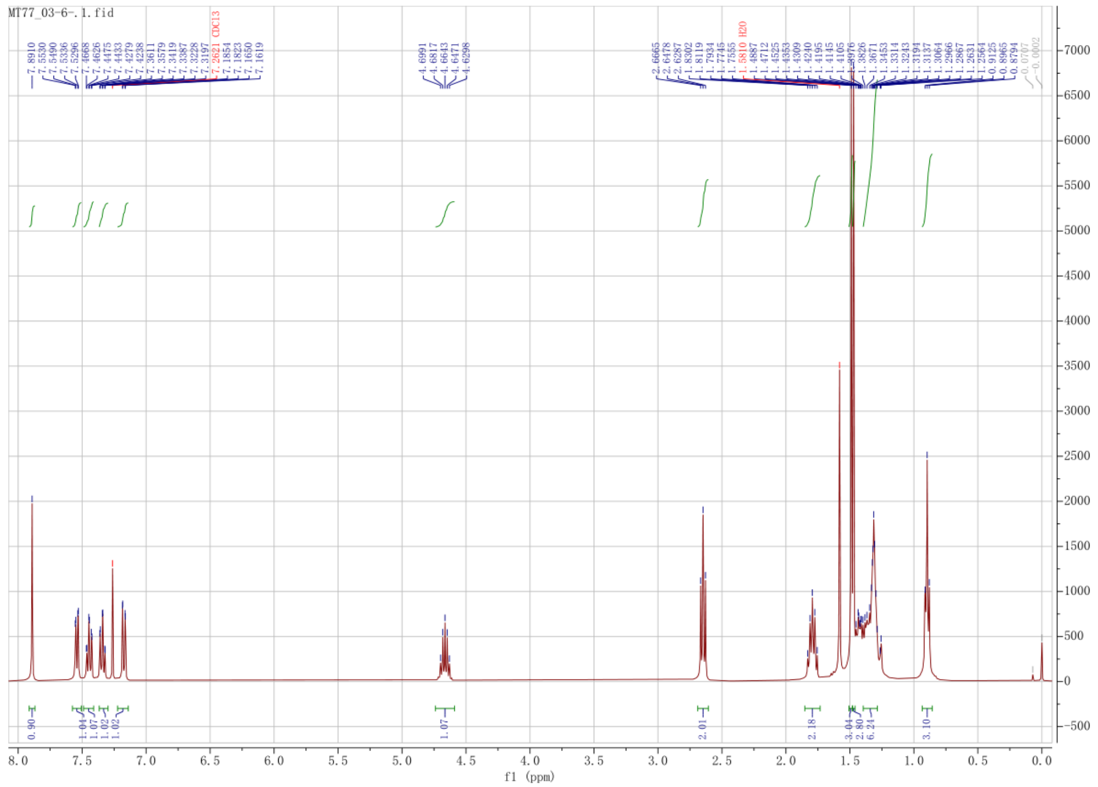

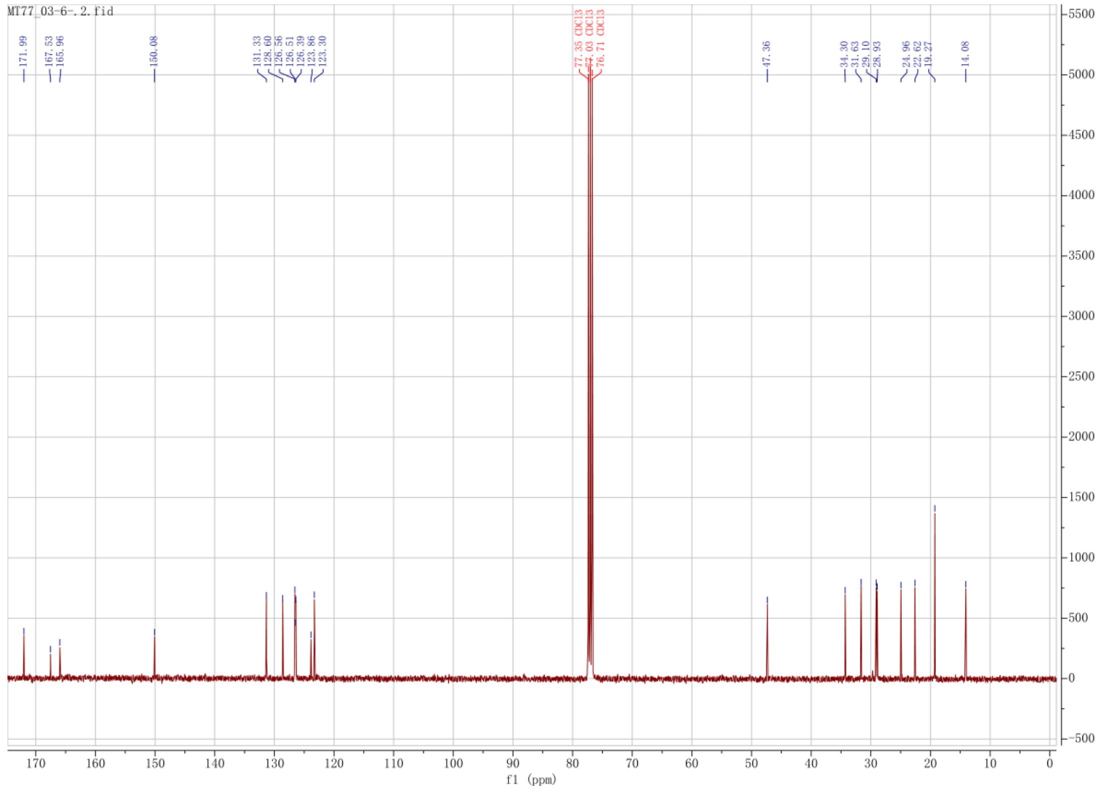

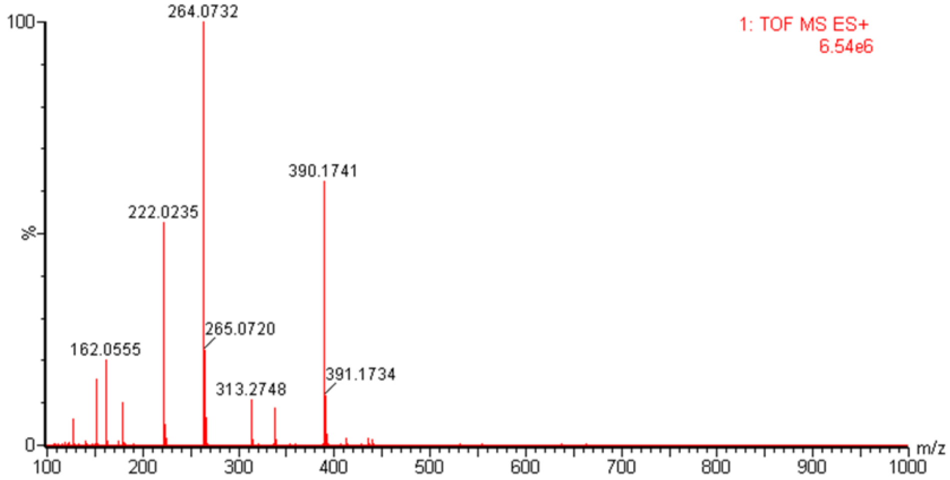

^{1}H NMR, ^{13}C NMR and HR-MS Spectra of compound 20

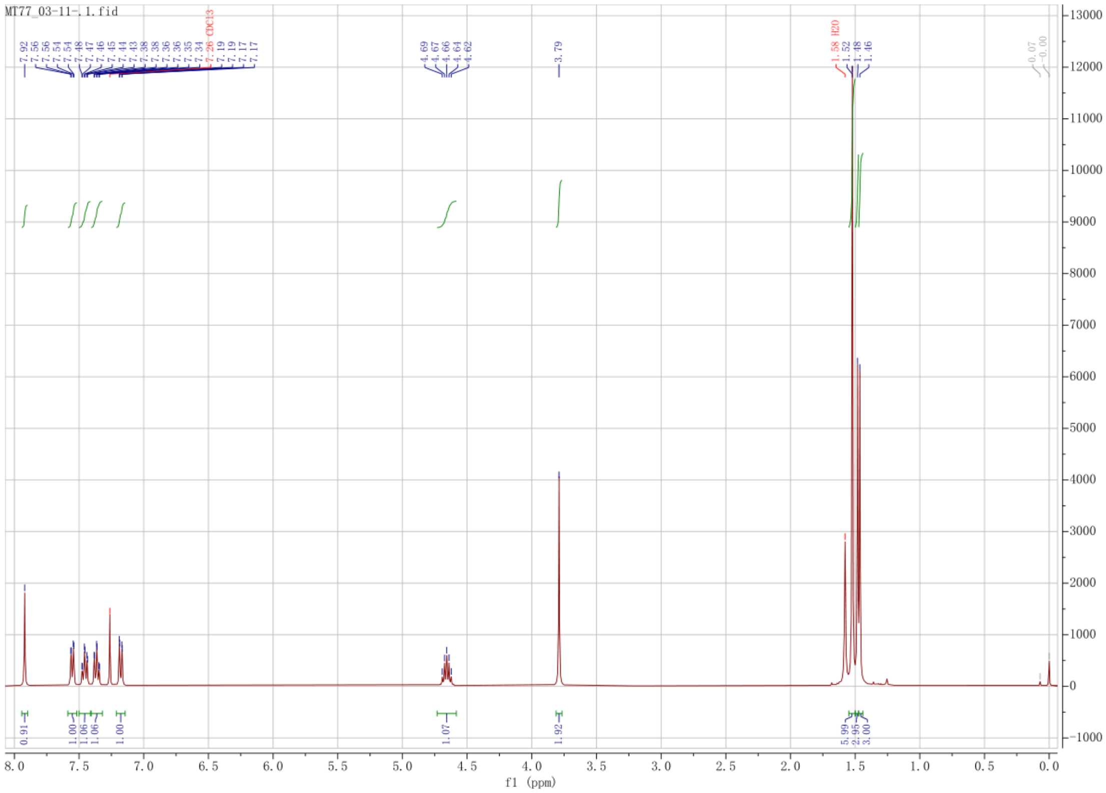

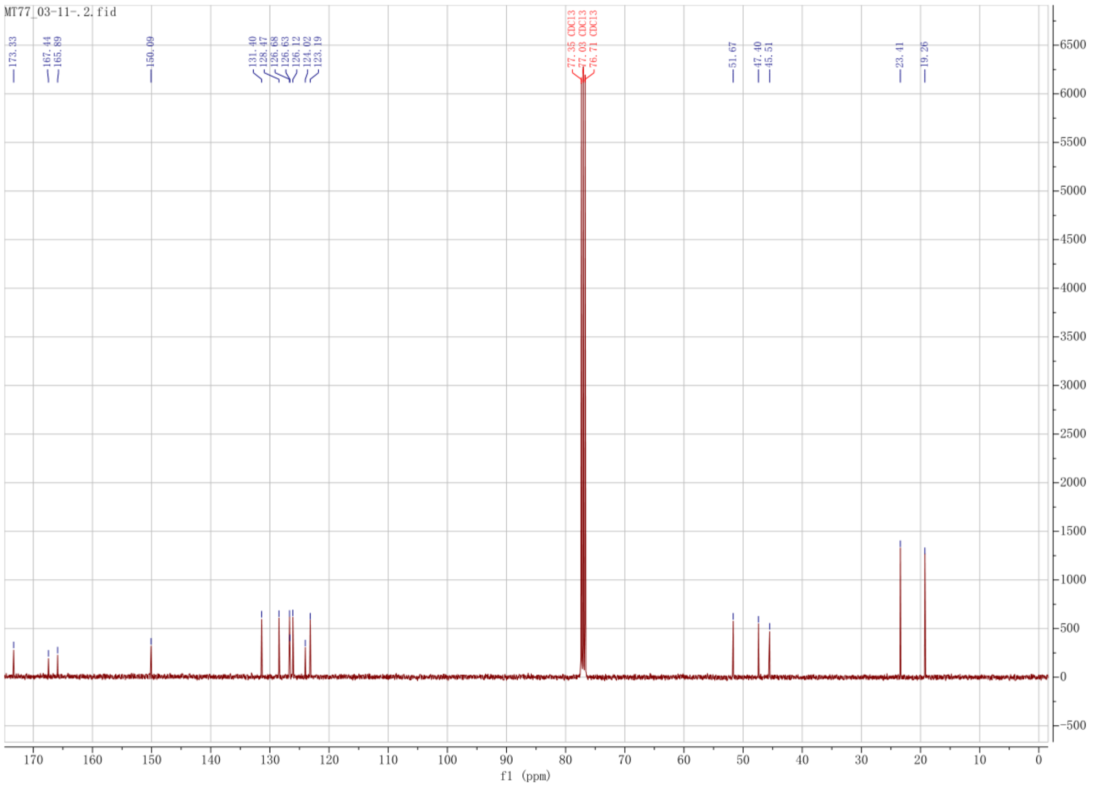

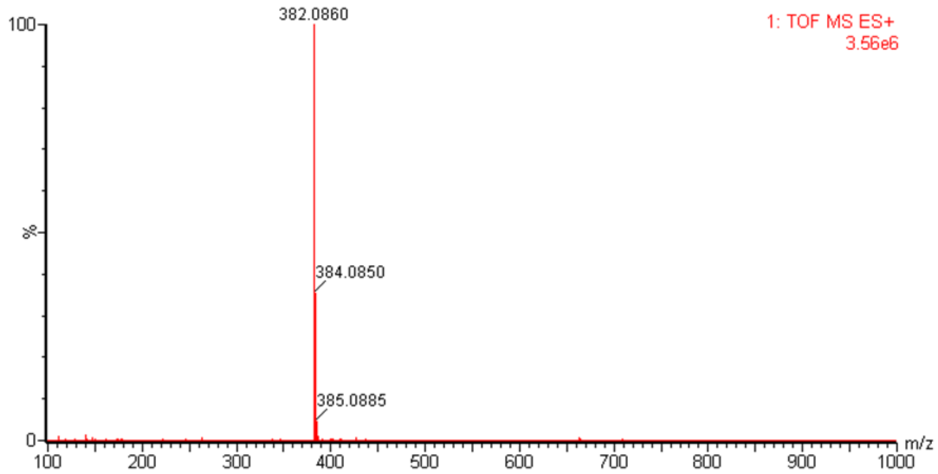

^{1}H NMR, ^{13}C NMR and HR-MS Spectra of compound 21

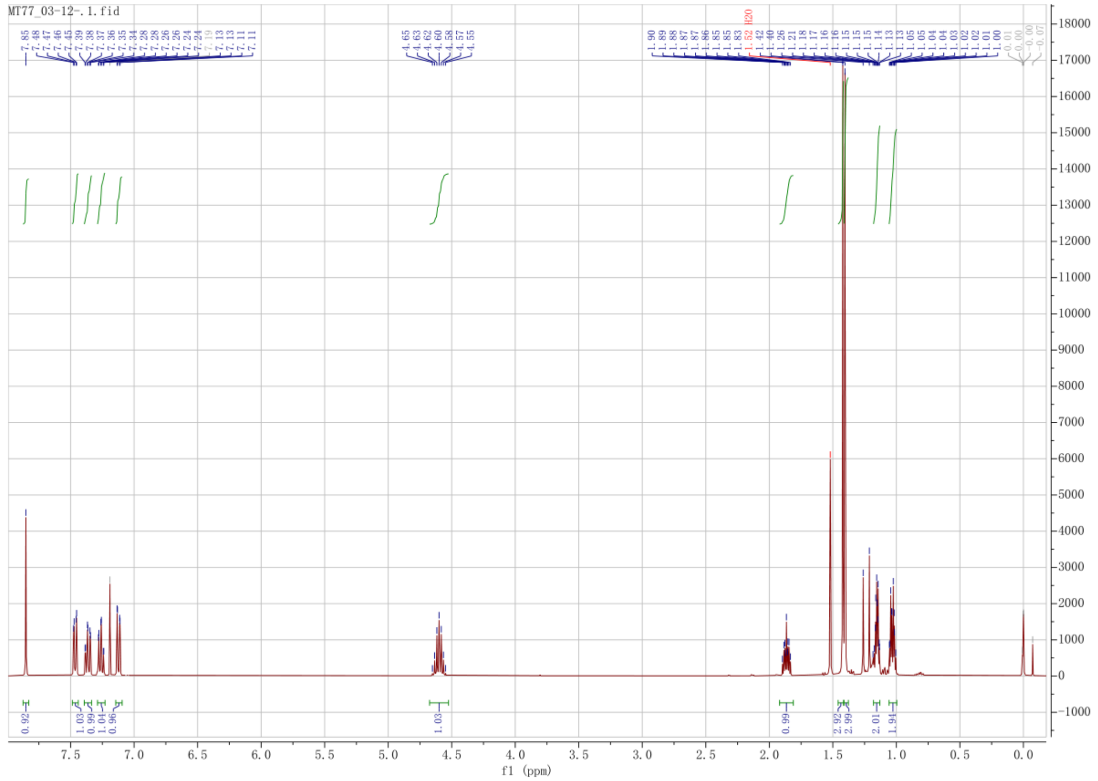

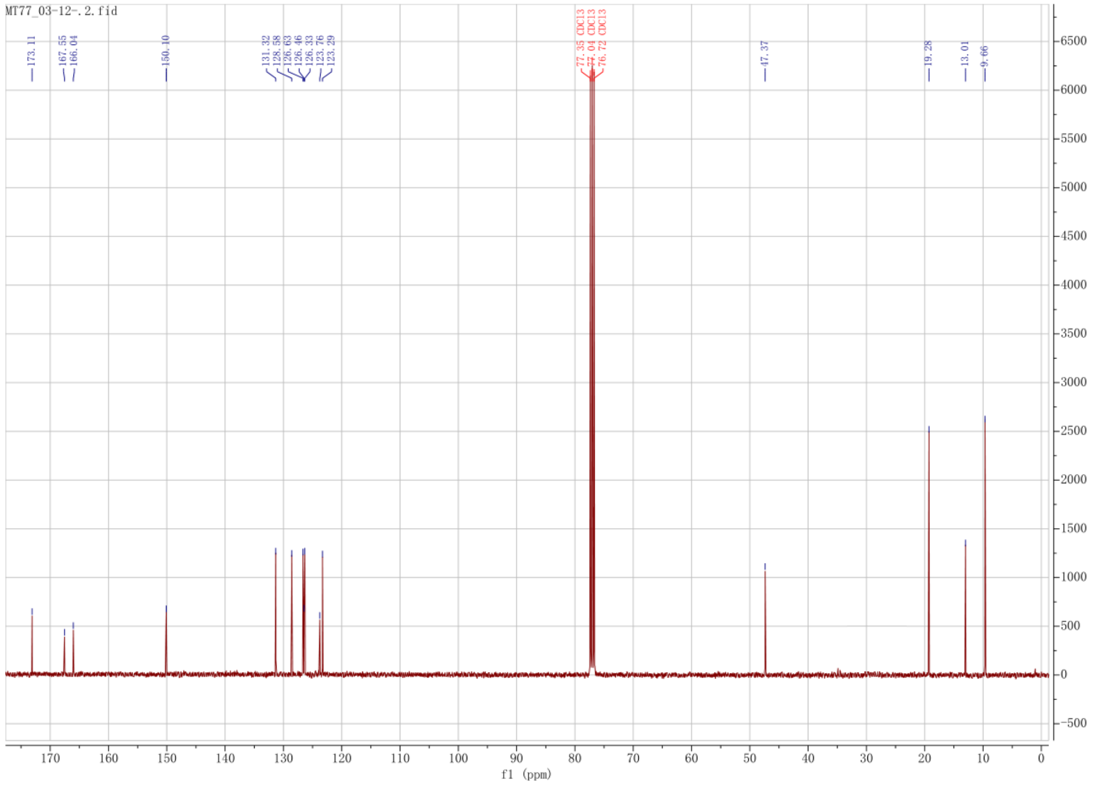

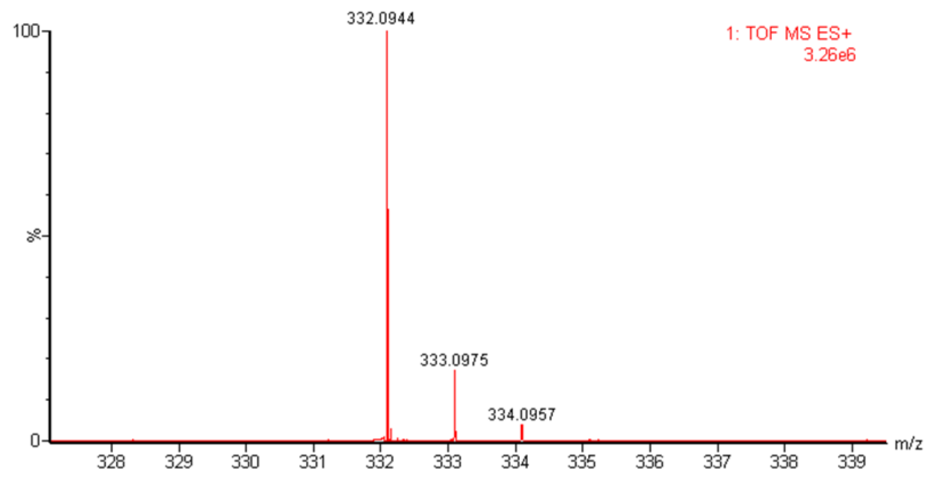

^{1}H NMR, ^{13}C NMR and HR-MS Spectra of compound 22

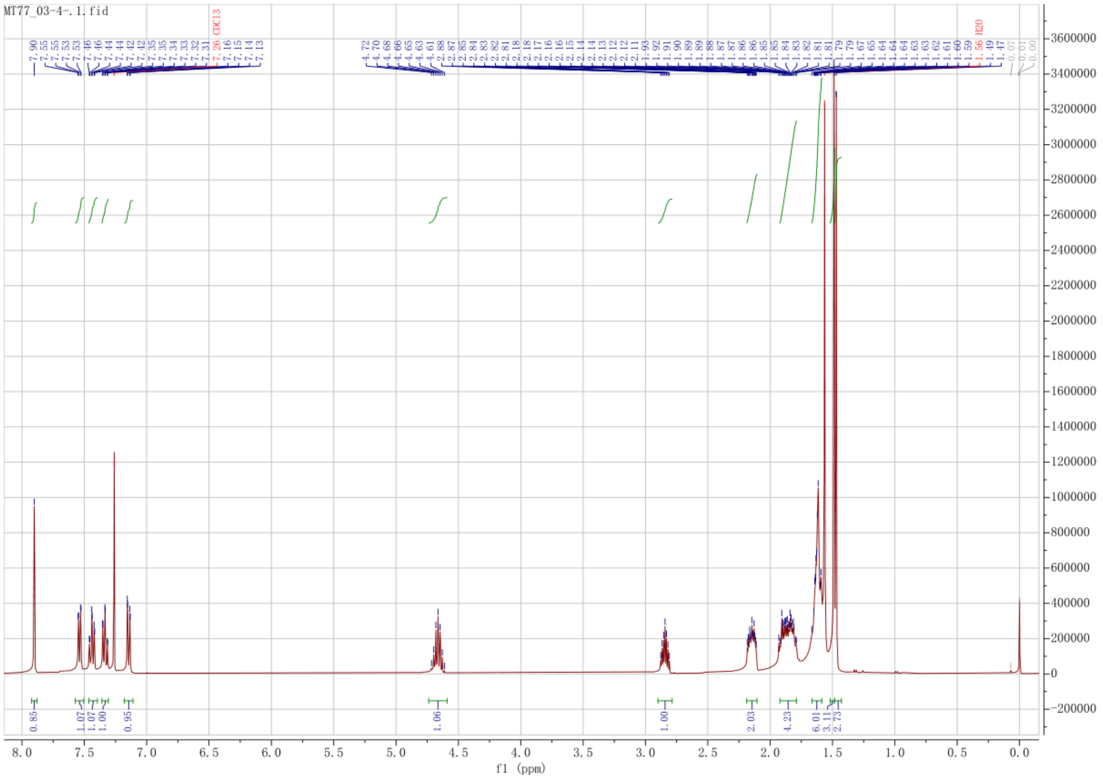

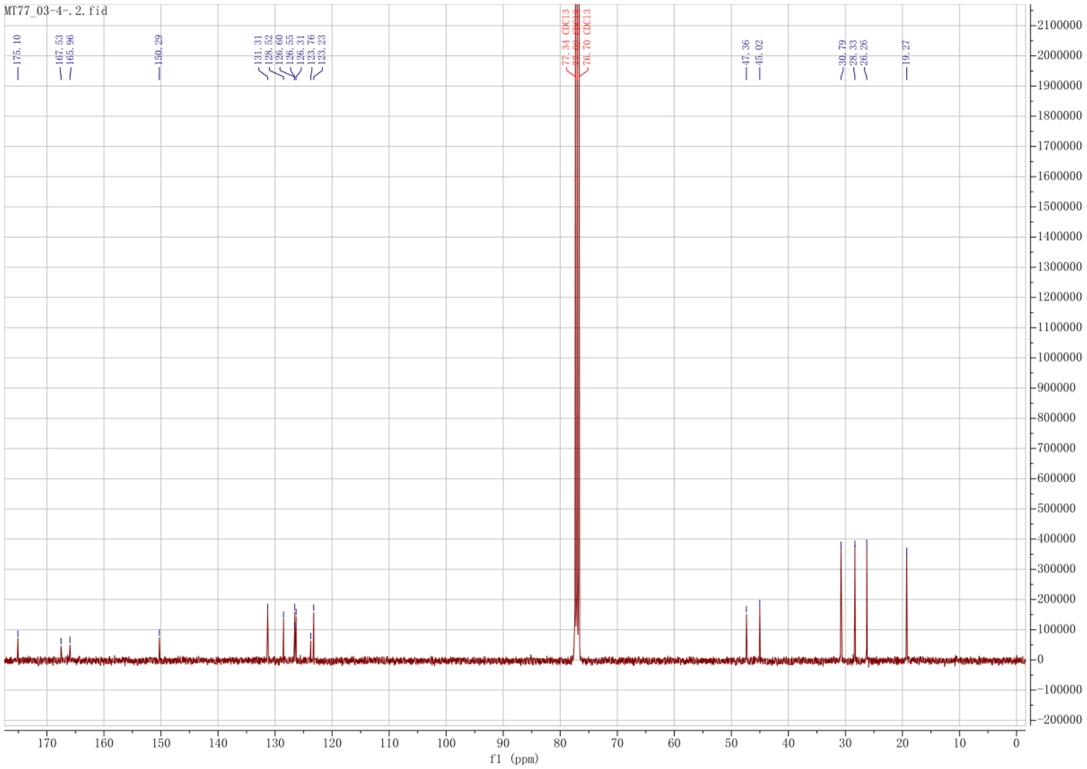

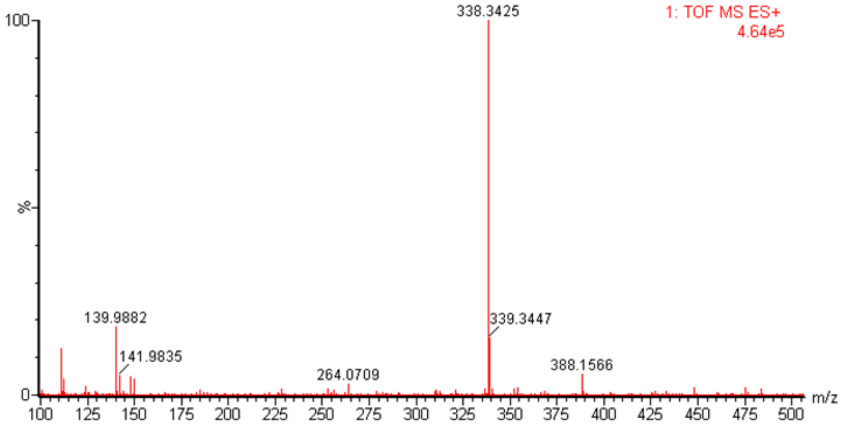

^{1}H NMR, ^{13}C NMR and HR-MS Spectra of compound 23

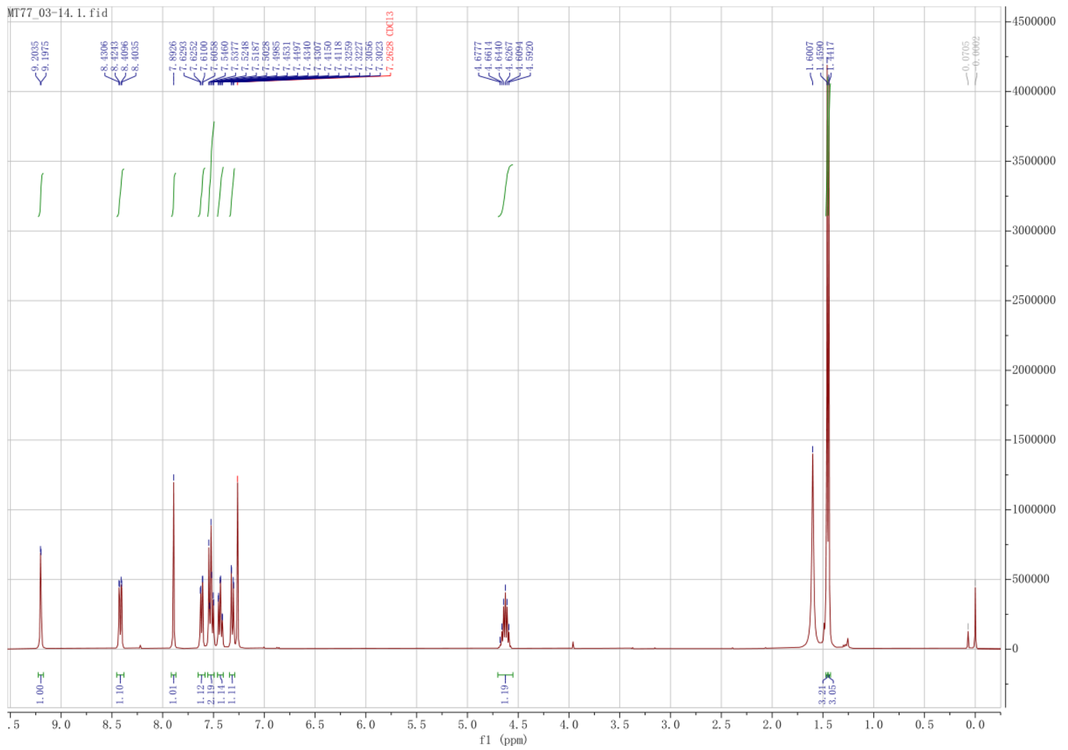
MT77_03-14.1.fid
f1 (ppm)

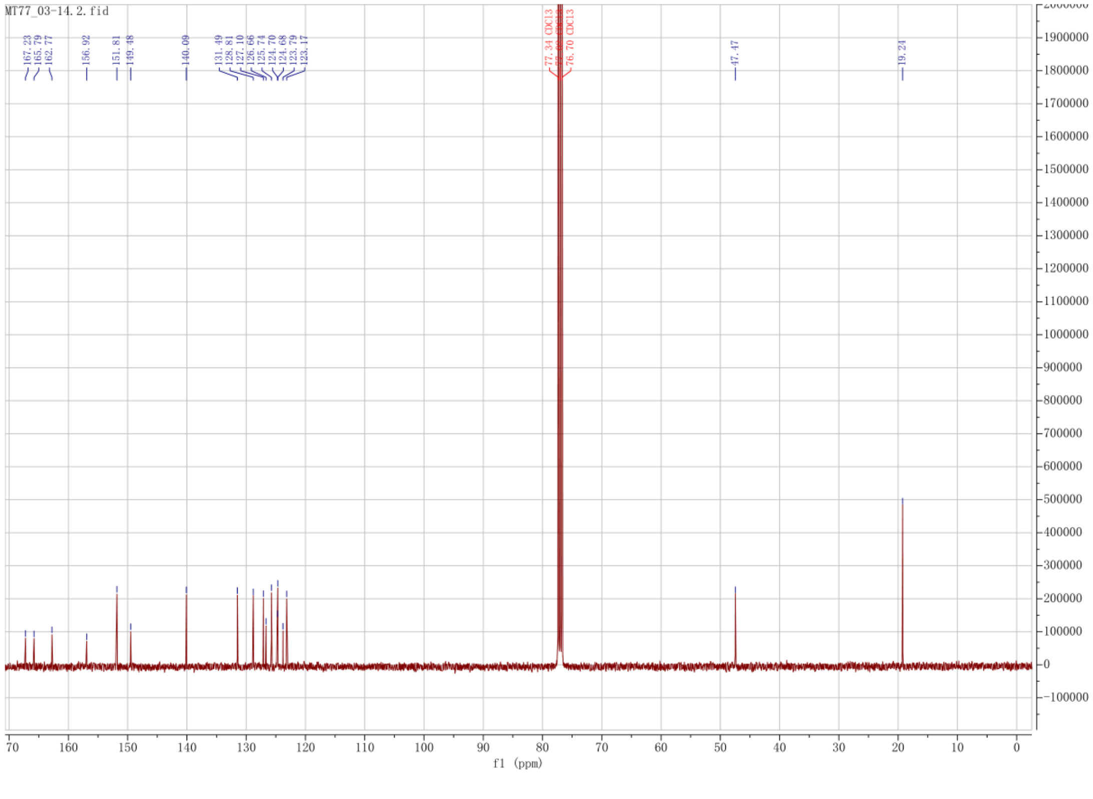
MT77_03-14.2.fid
f1 (ppm)

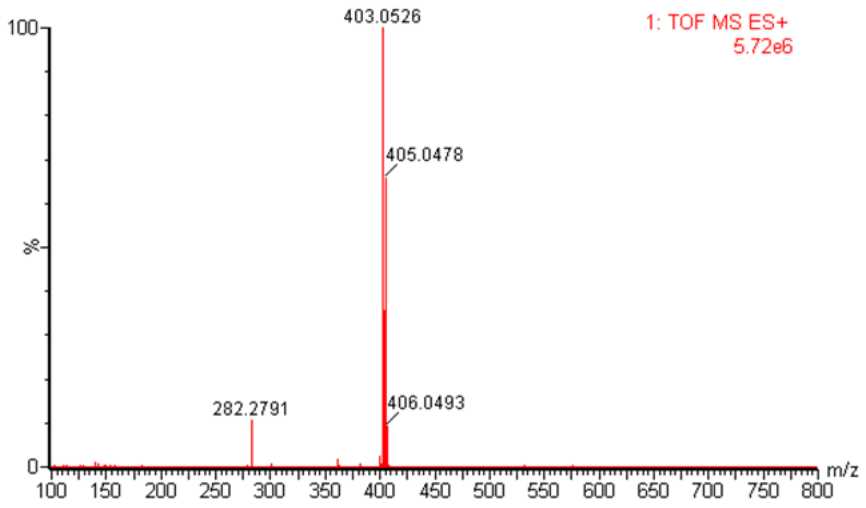

^{1}H NMR, ^{13}C NMR and HR-MS Spectra of compound 24

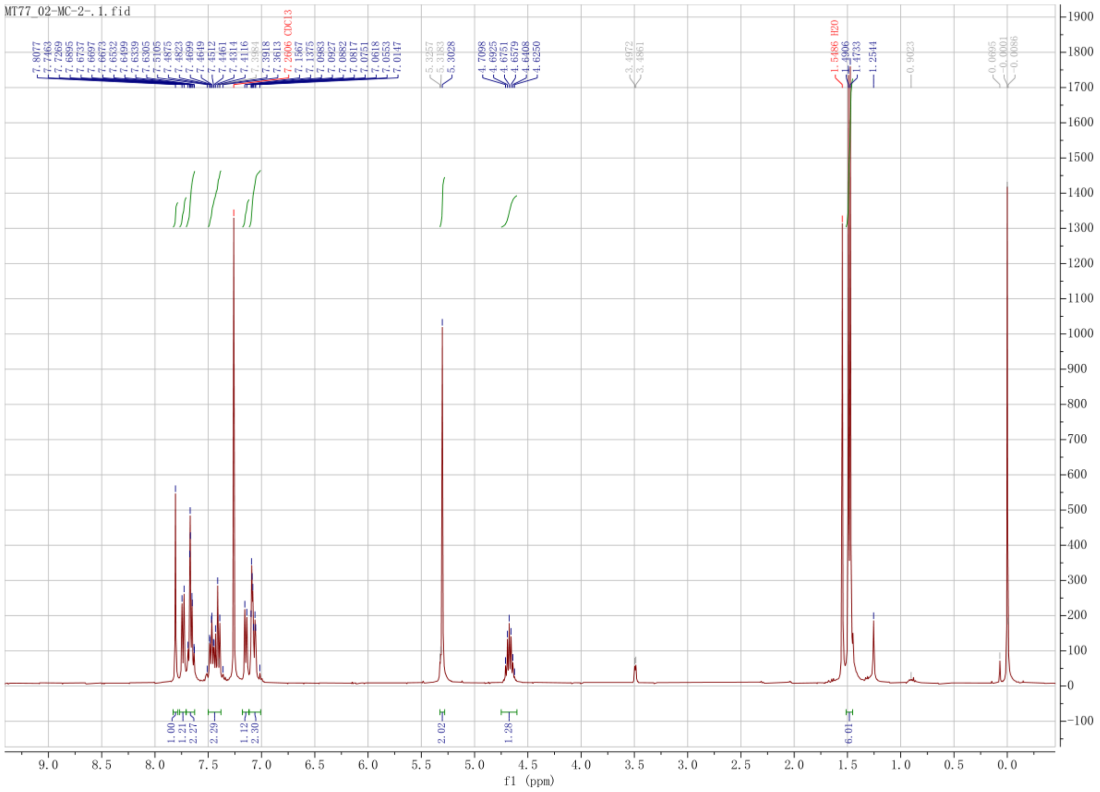

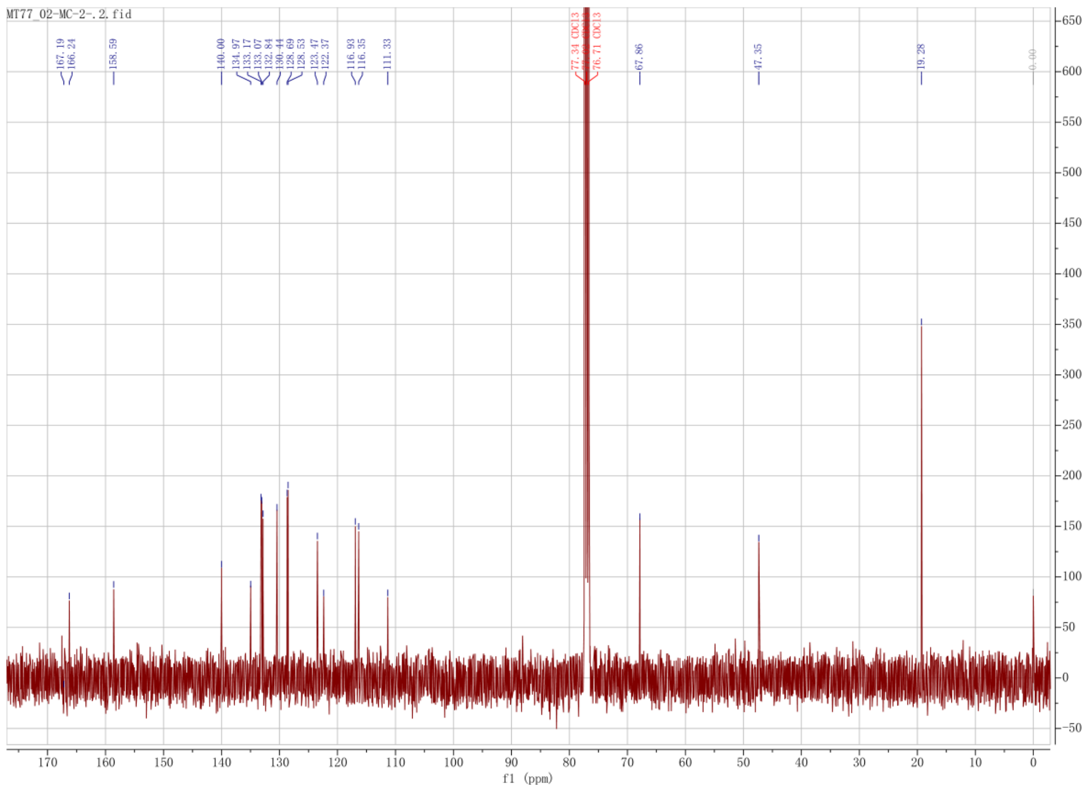

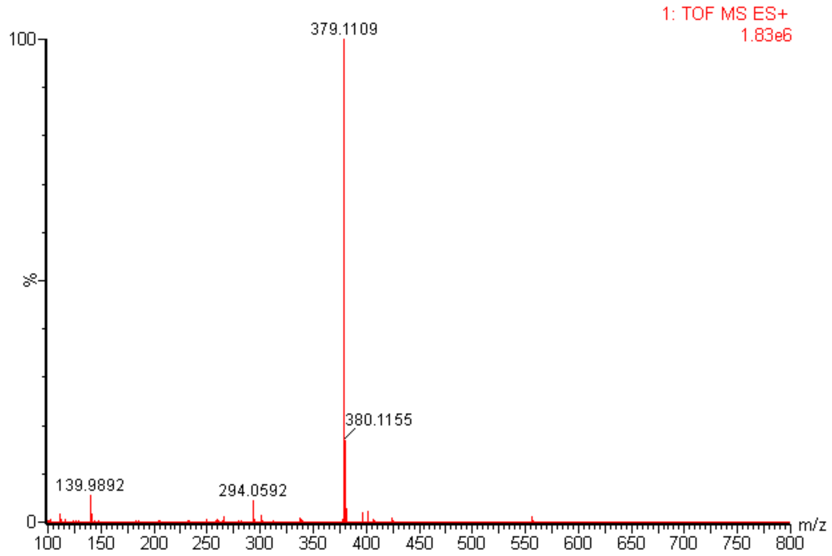

^{1}H NMR, ^{13}C NMR and HR-MS Spectra of compound 25

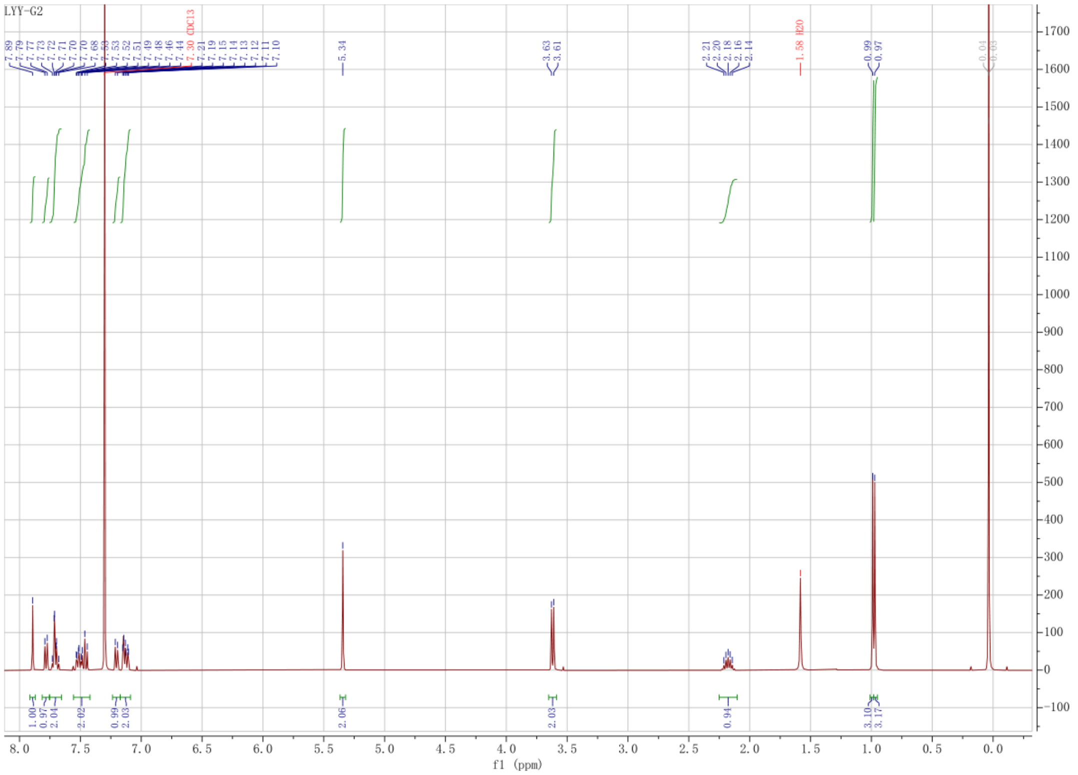

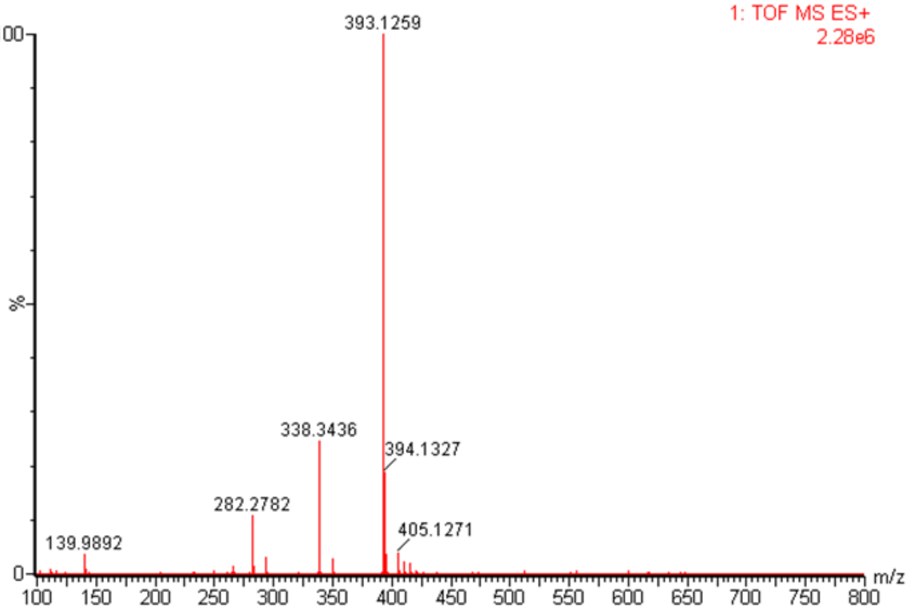

^{1}H NMR and HR-MS Spectra of compound 26

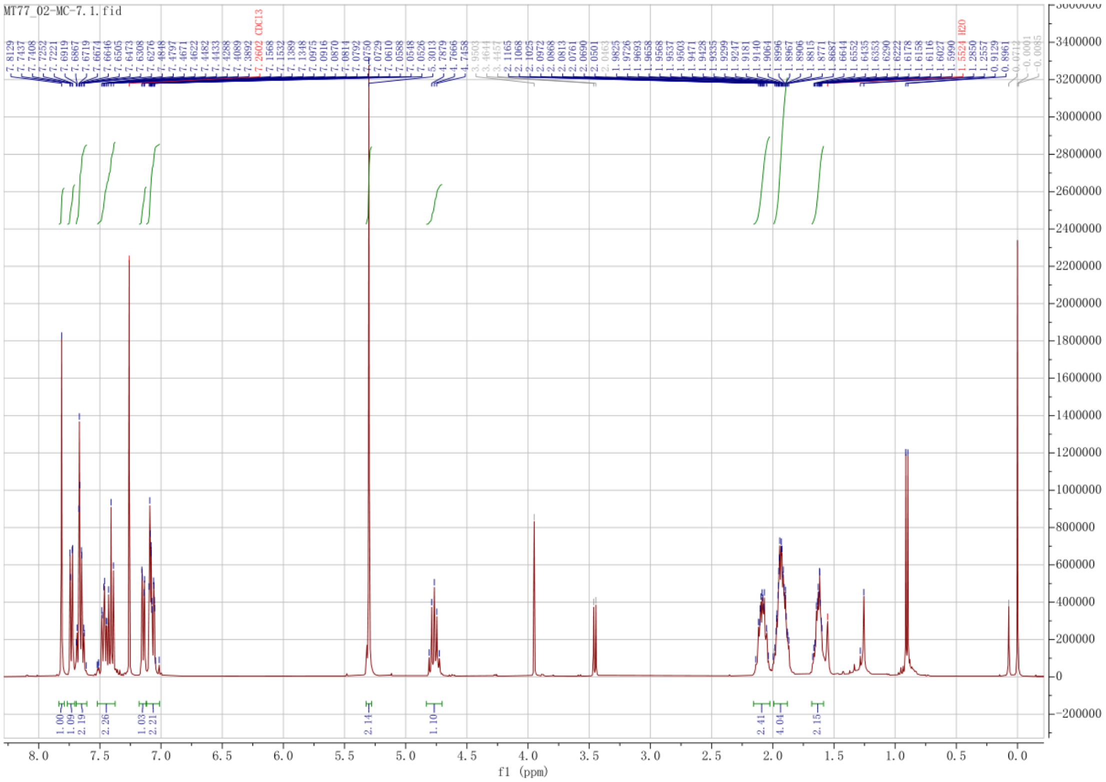
MT77_02-MC-7.1.fid
f1 (ppm)

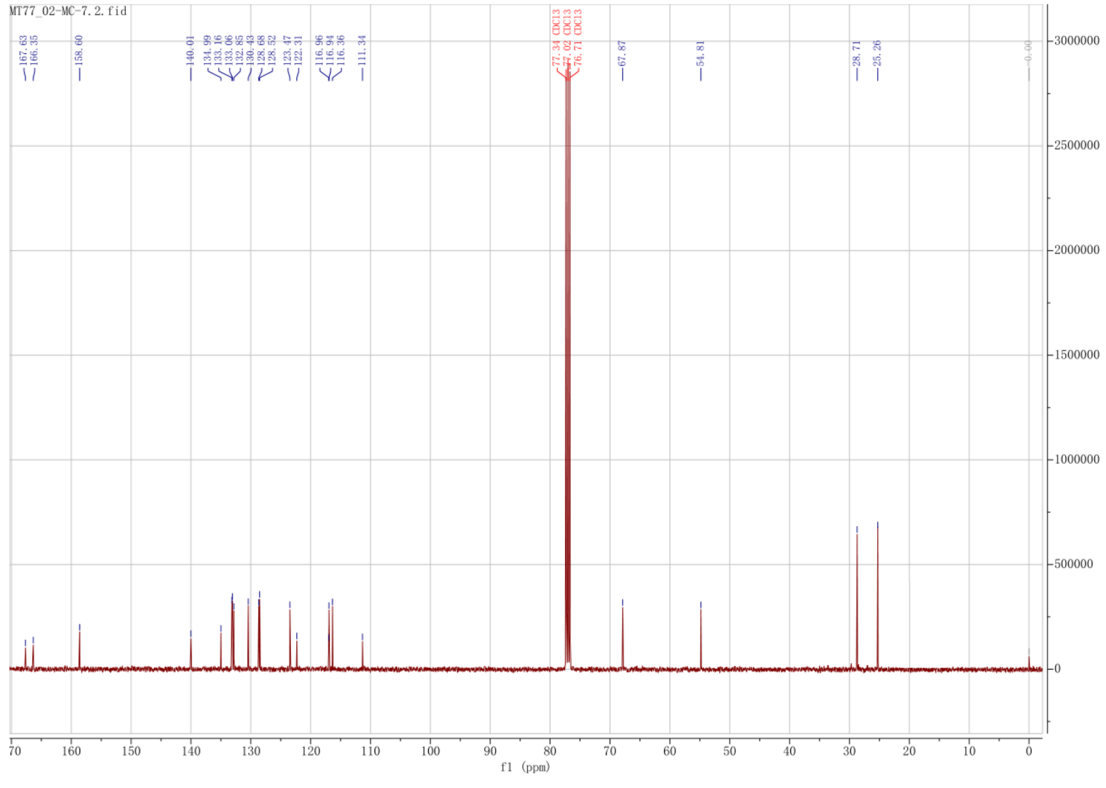
MT77_02-MC-7.2.fid
167.63
166.35
158.60
140.01
134.99
133.16
133.06
132.85
130.43
128.68
128.52
123.47
122.31
116.96
116.94
116.36
111.34
77.34 CDCl3
77.02 CDCl3
76.71 CDCl3
67.87
54.81
28.71
25.26
f1 (ppm)

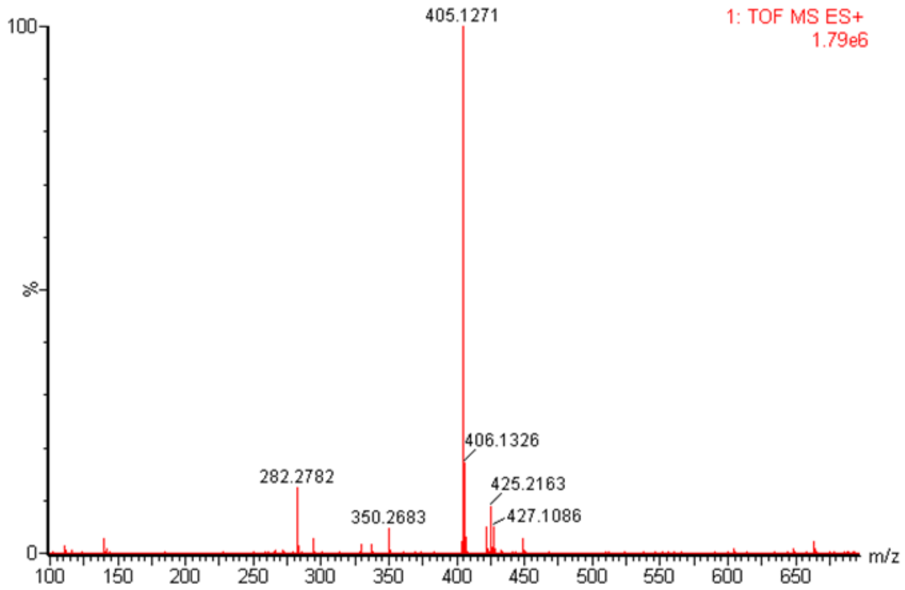

^{1}H NMR, ^{13}C NMR and HR-MS Spectra of compound 27

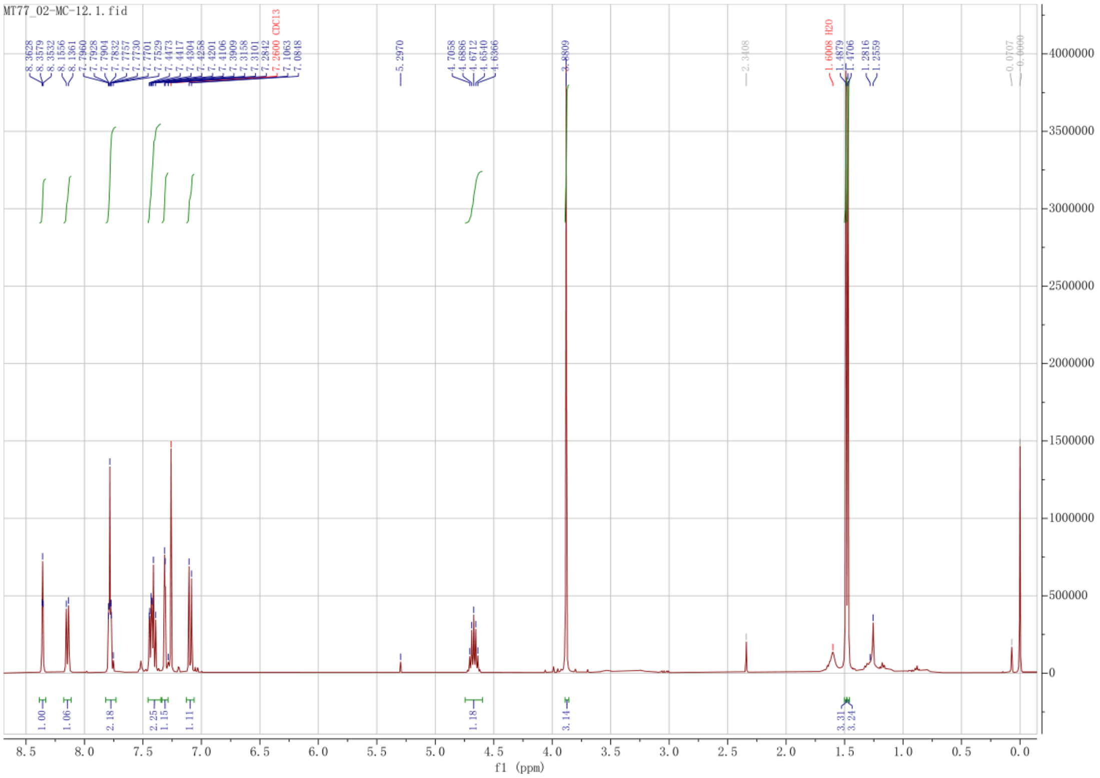

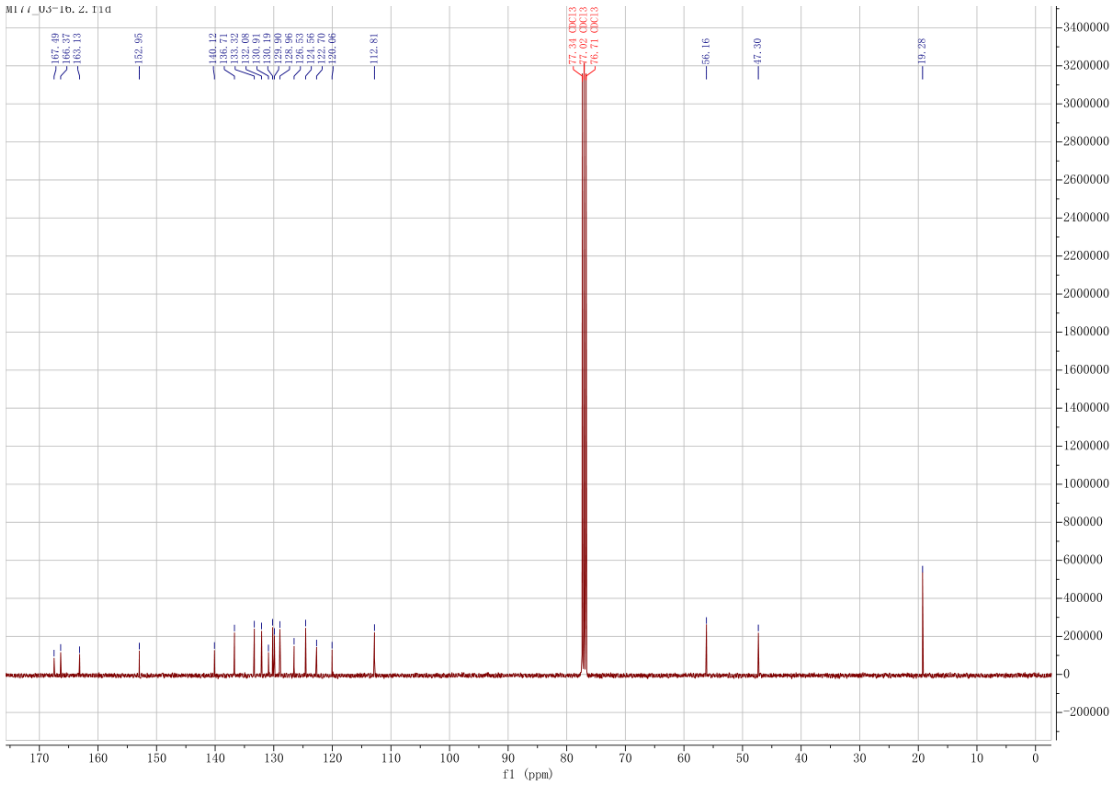

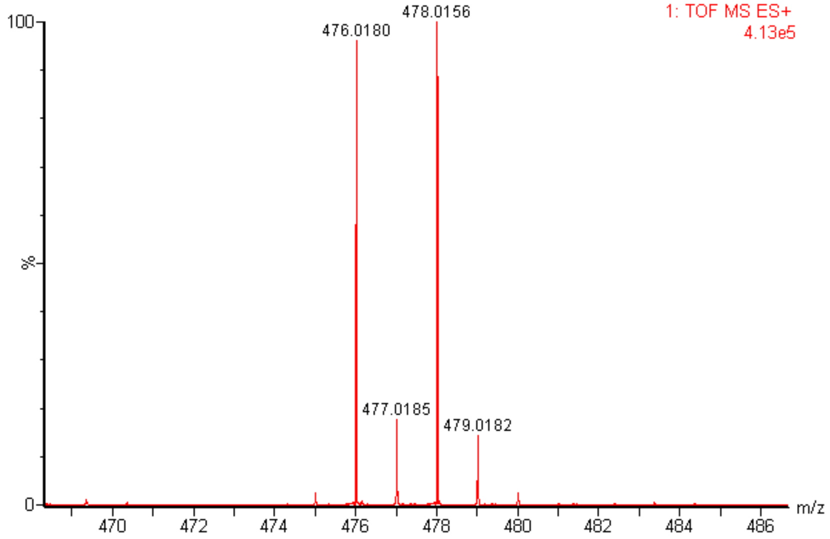

^{1}H NMR, ^{13}C NMR and HR-MS Spectra of compound 28

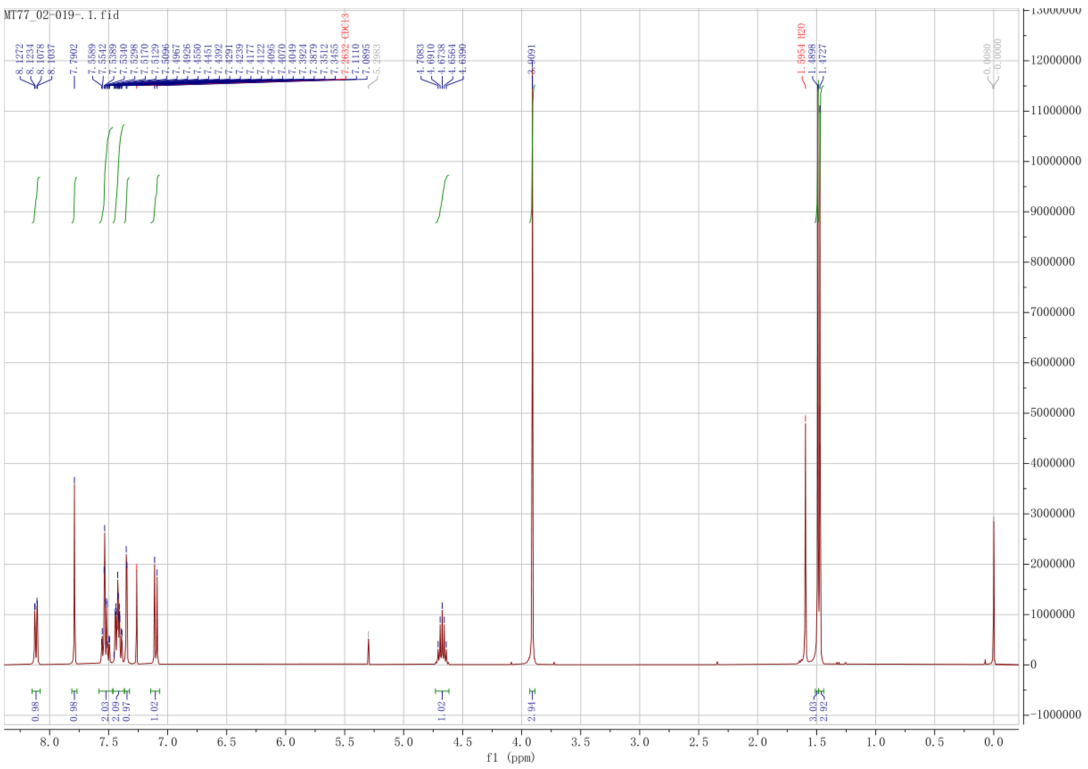

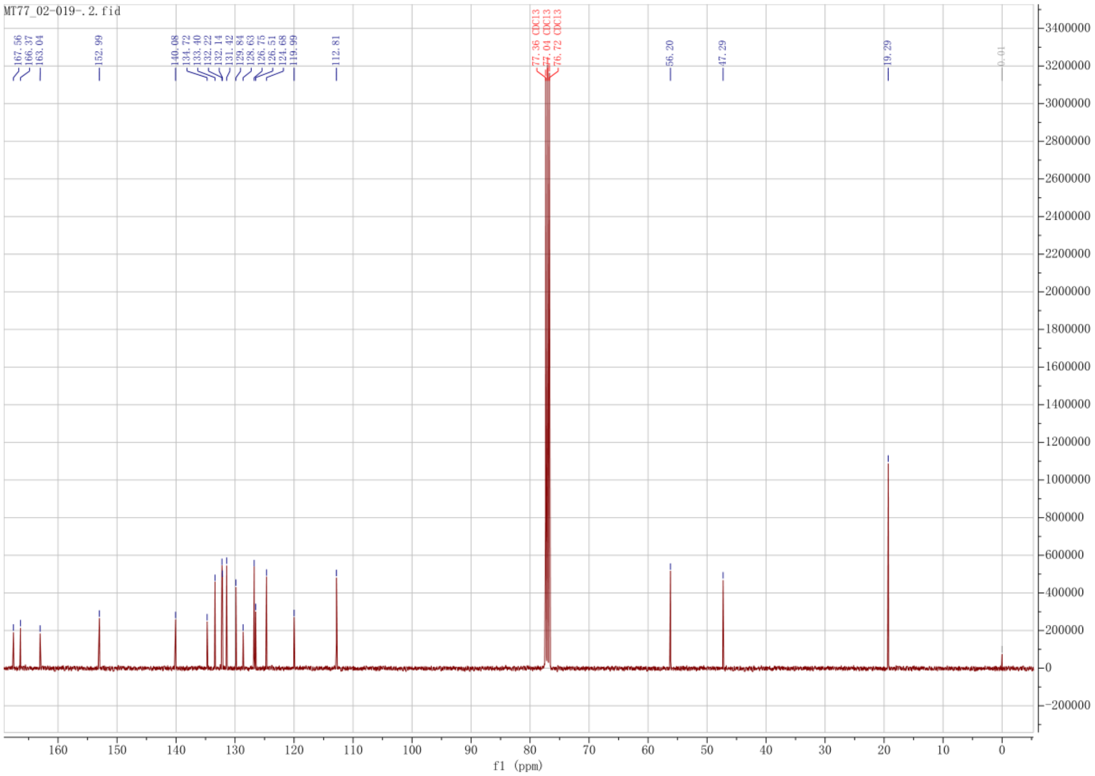

¹H NMR, ¹³C NMR and HR-MS Spectra of compound 29

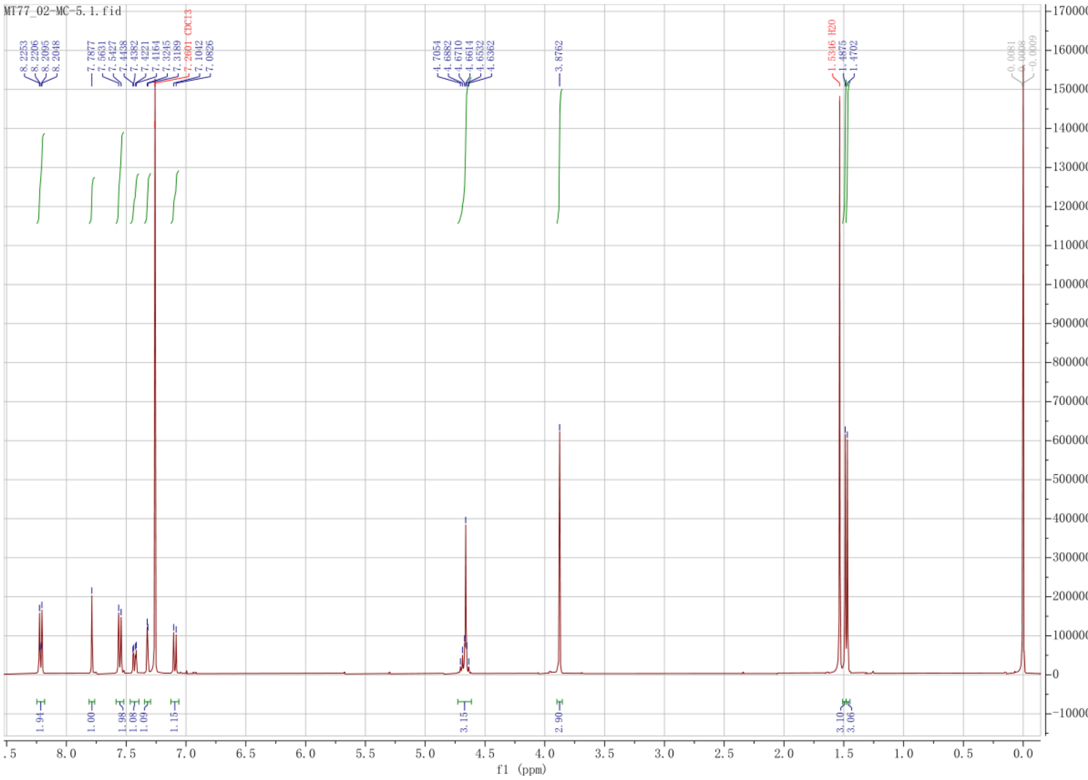

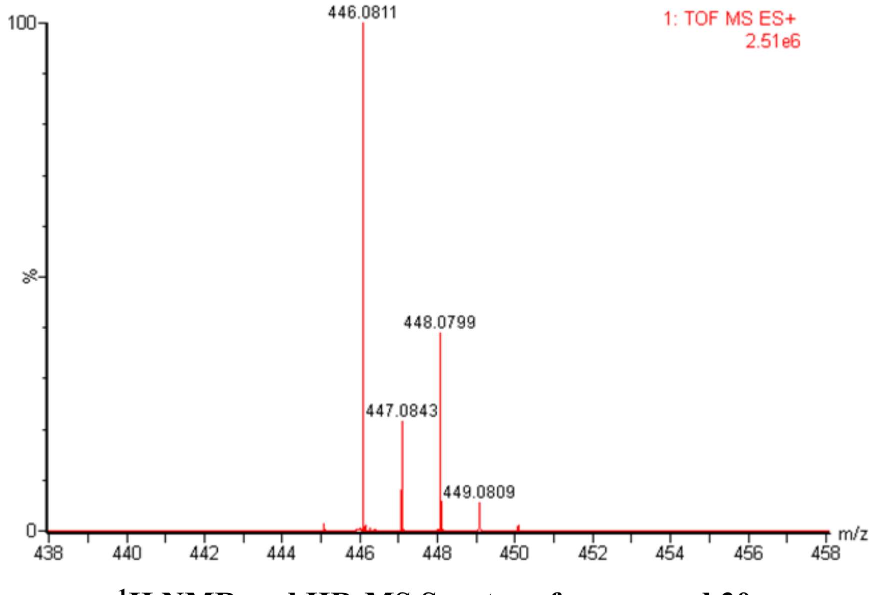

^{1}H NMR and HR-MS Spectra of compound 30

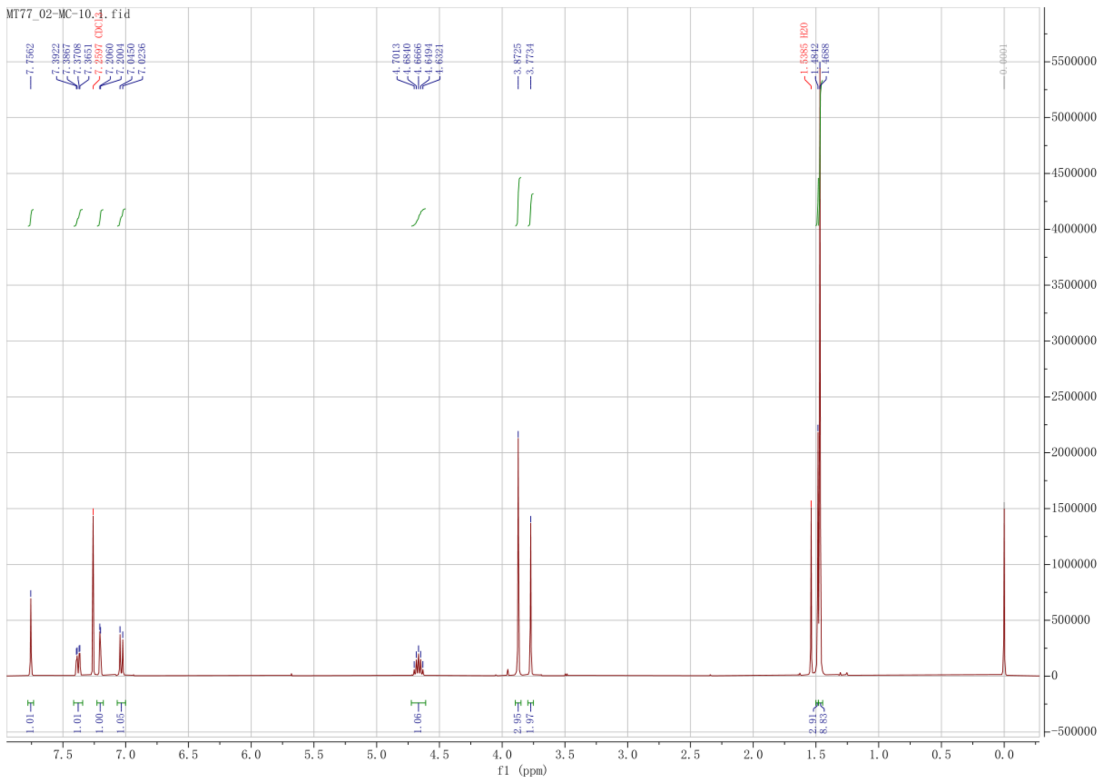
MT77_02-MC-10.1.fid
7.7562
7.3922
7.3867
7.3708
7.3651
7.2597 CDCl3
7.2060
7.2004
7.0450
7.0236
4.7013
4.6840
4.6666
4.6494
4.6321
3.8725
3.7734
1.5385 H2O
1.4842
1.4688
0.0001
1.01
1.01
1.00
1.05
1.06
2.95
1.97
2.91
8.83
f1 (ppm)

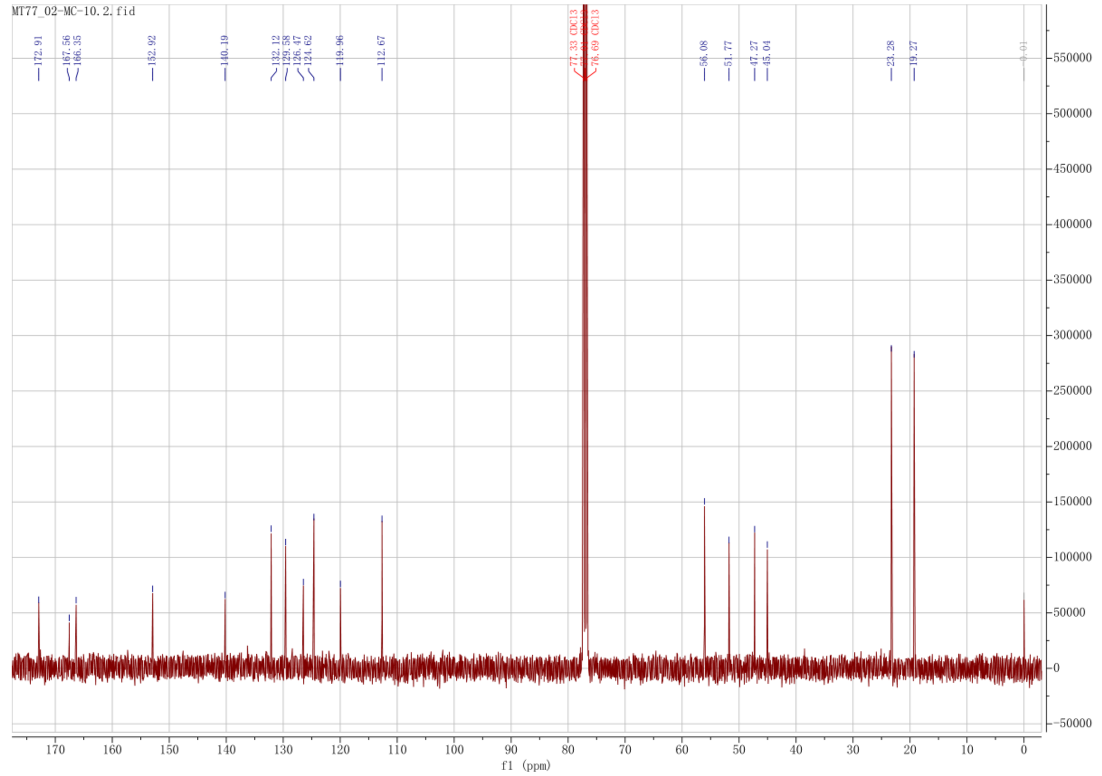
MT77_02-MC-10.2.fid
172.91
167.56
166.35
152.92
140.19
132.12
129.58
126.47
124.62
119.96
112.67
77.33 CDCl3
76.69 CDCl3
56.08
51.77
47.27
45.04
23.28
19.27
f1 (ppm)

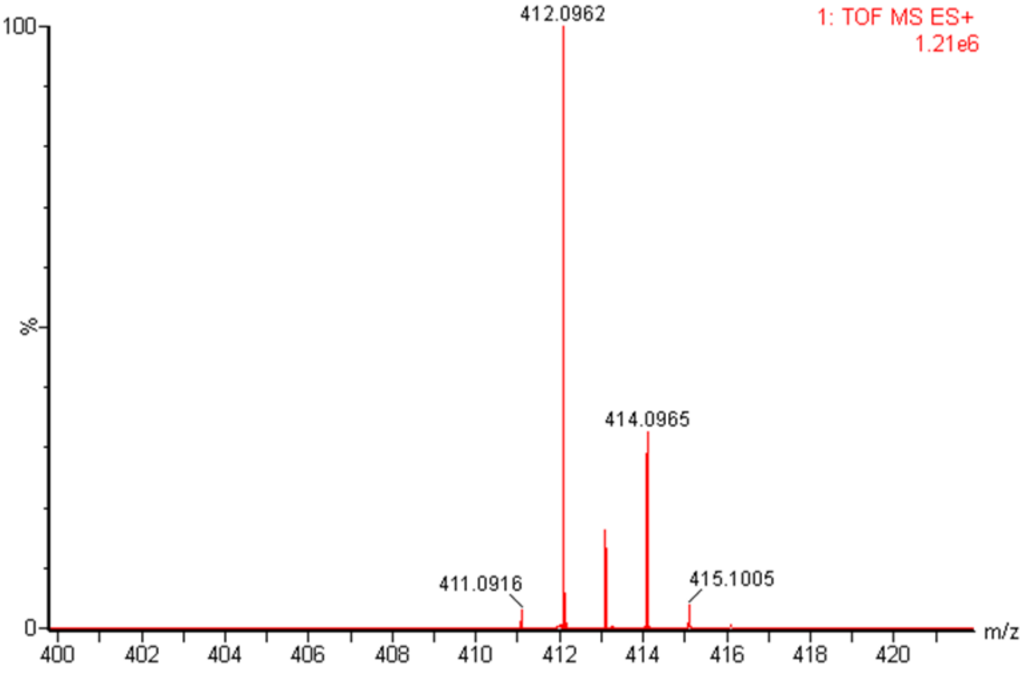

^{1}H NMR, ^{13}C NMR and HR-MS Spectra of compound 31

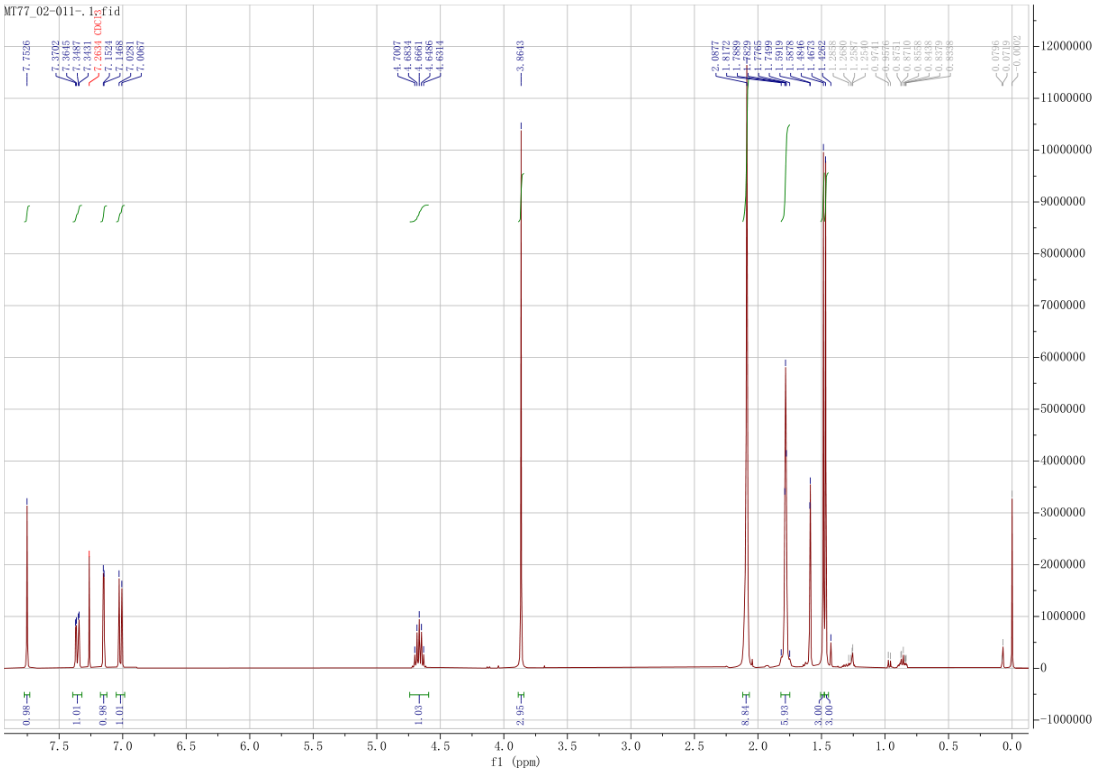

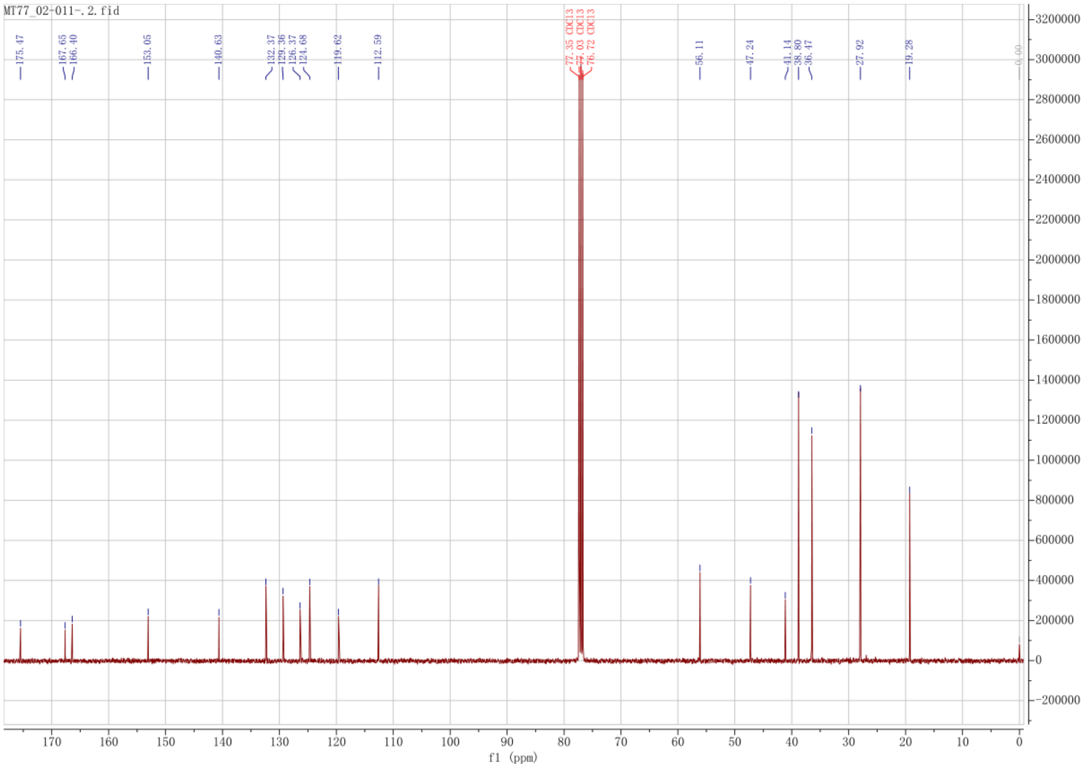

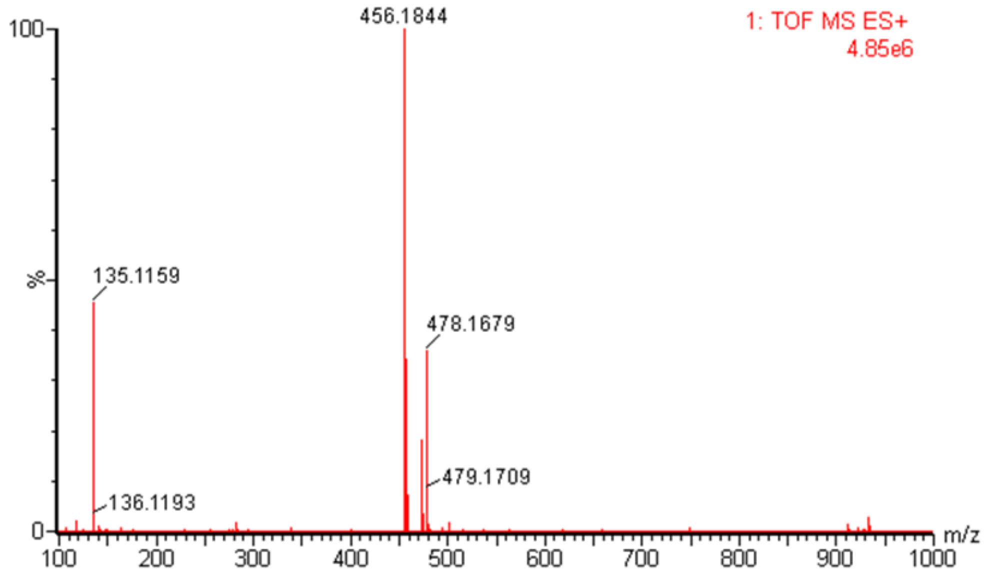

^{1}H NMR, ^{13}C NMR and HR-MS Spectra of compound 32

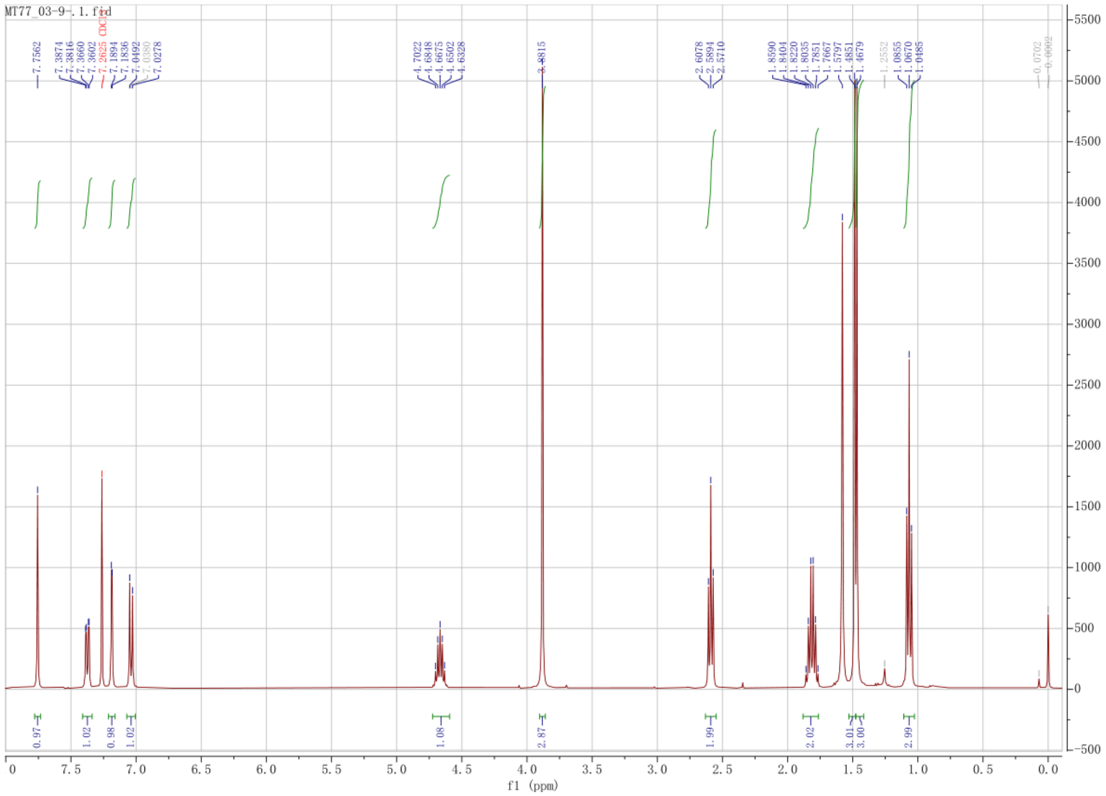
MT77_03-9-.1.fid
f1 (ppm)

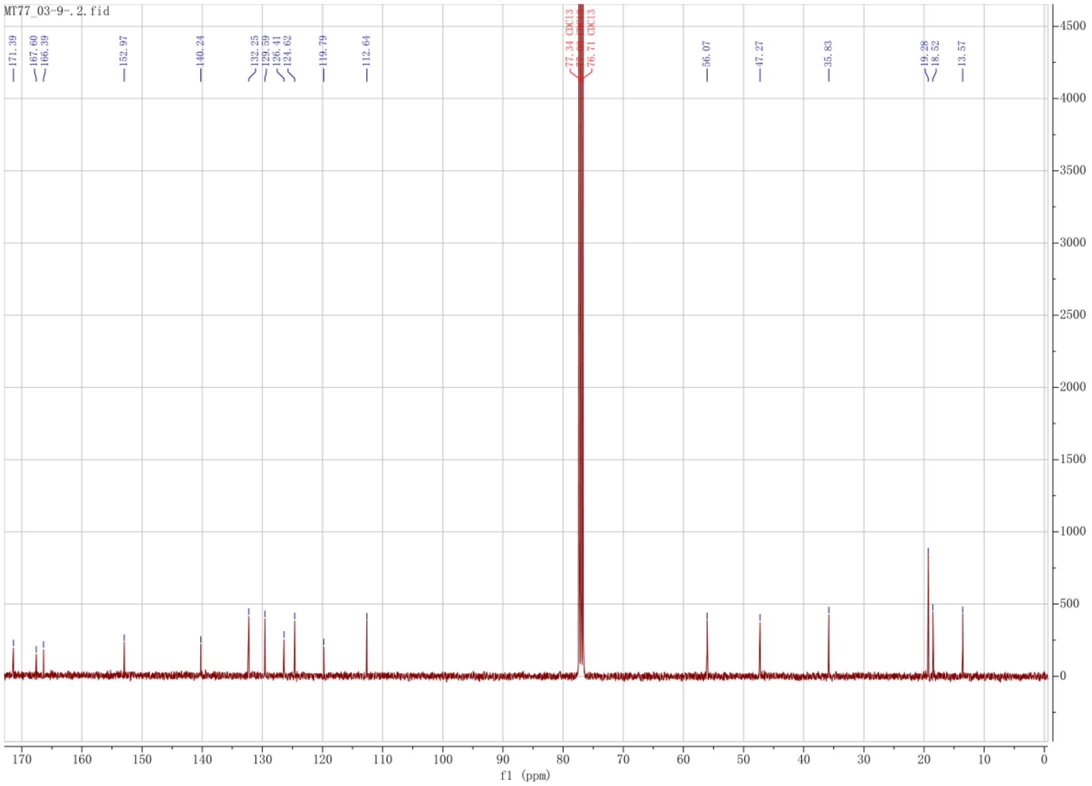
MT77_03-9-.2.fid
f1 (ppm)

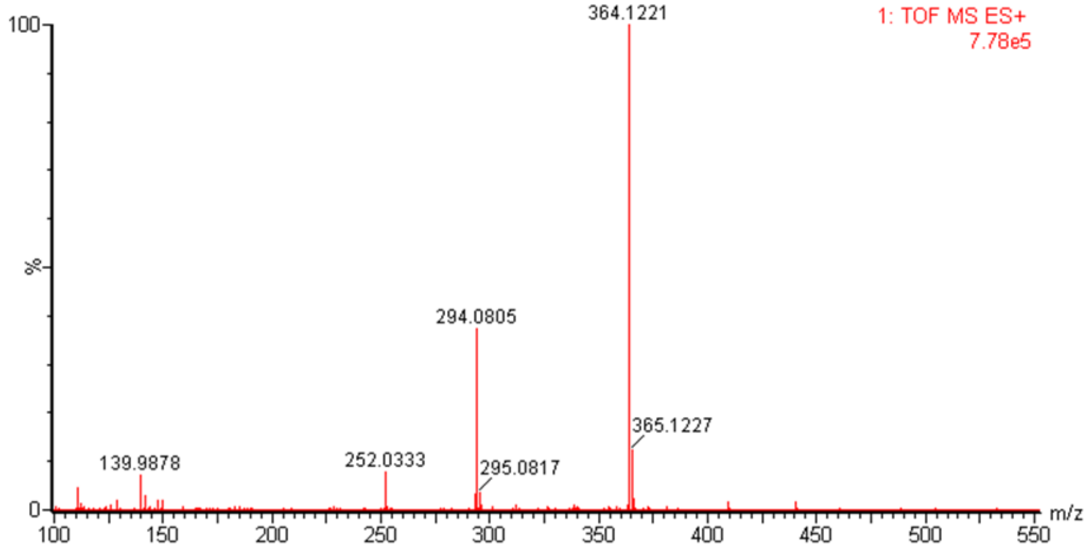

^{1}H NMR, ^{13}C NMR and HR-MS Spectra of compound 33

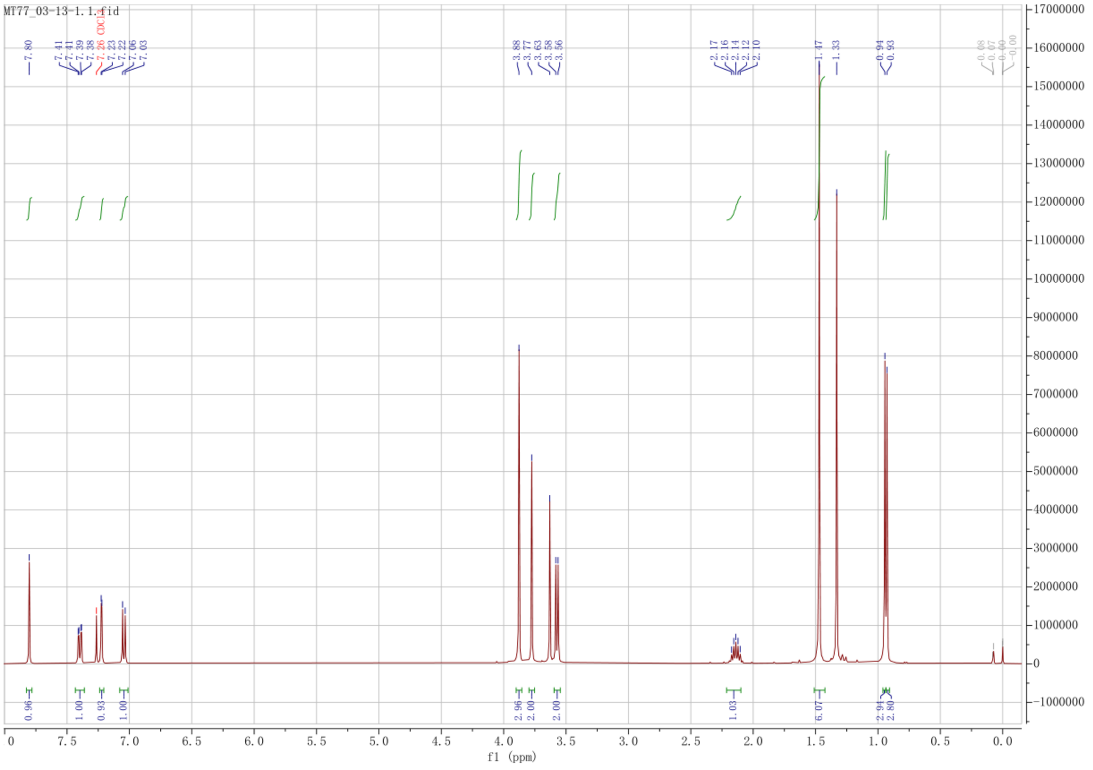

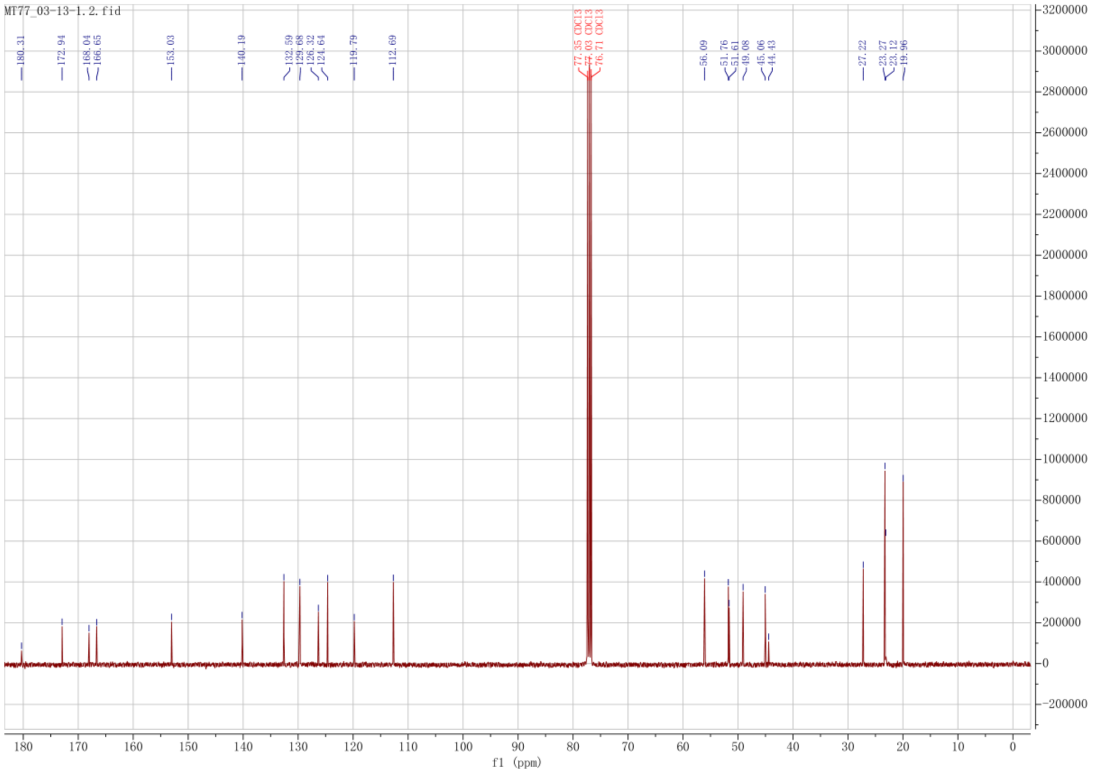

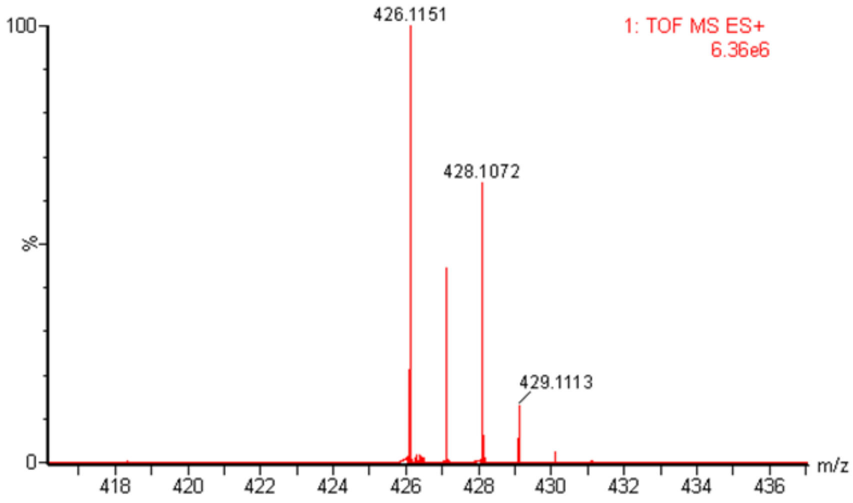

^{1}H NMR, ^{13}C NMR and HR-MS Spectra of compound 34

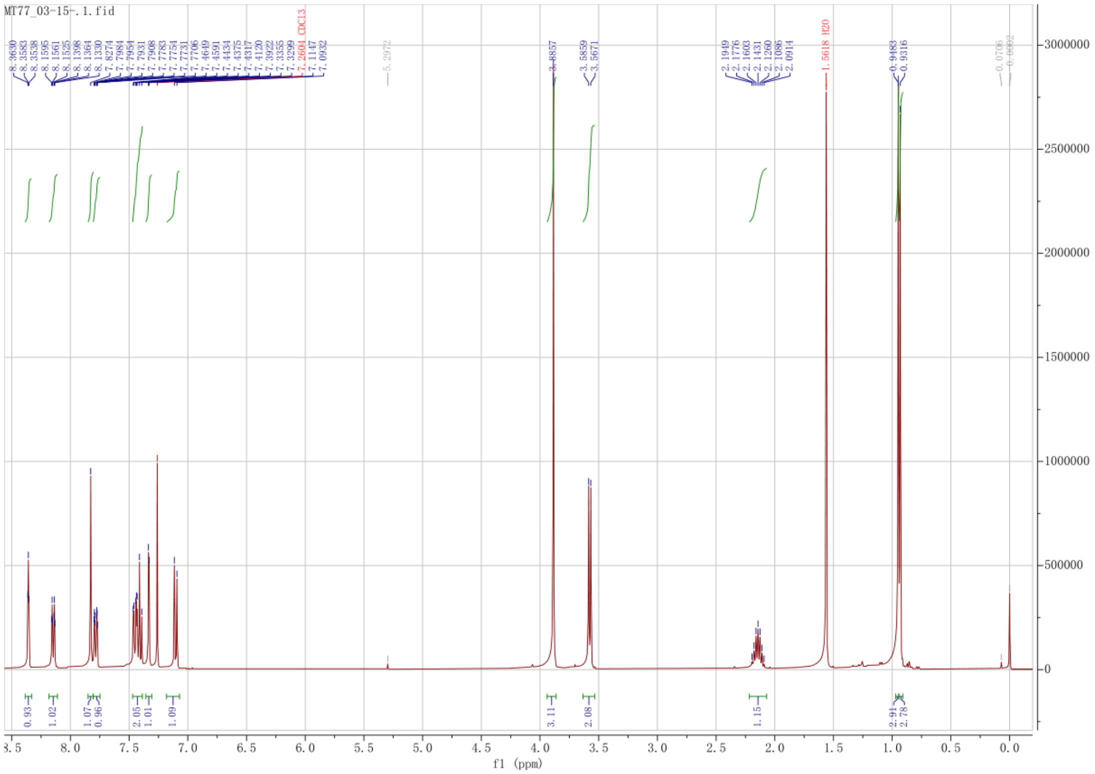

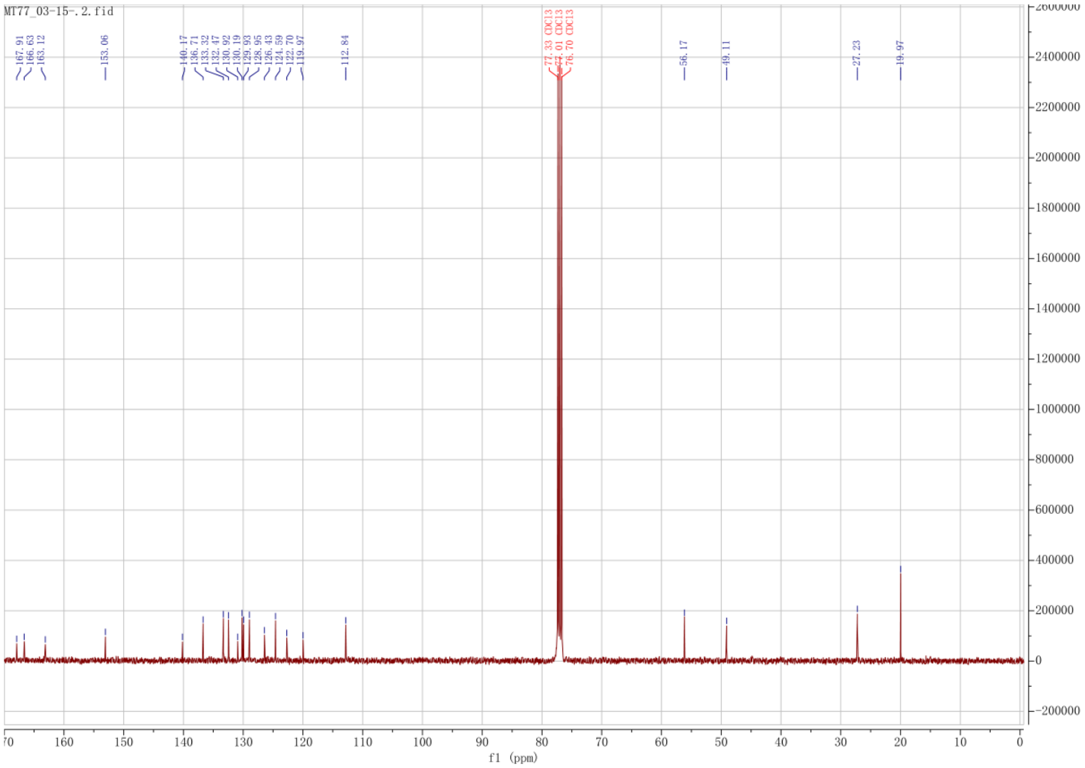

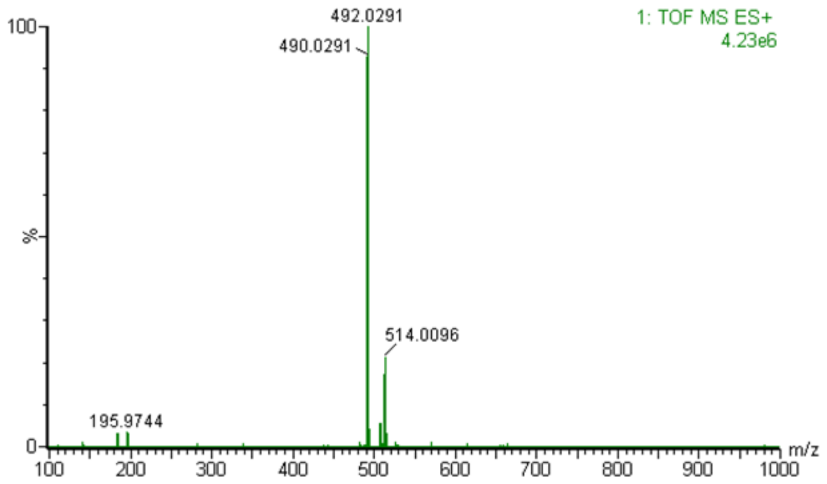

^{1}H NMR, ^{13}C NMR and HR-MS Spectra of compound 35

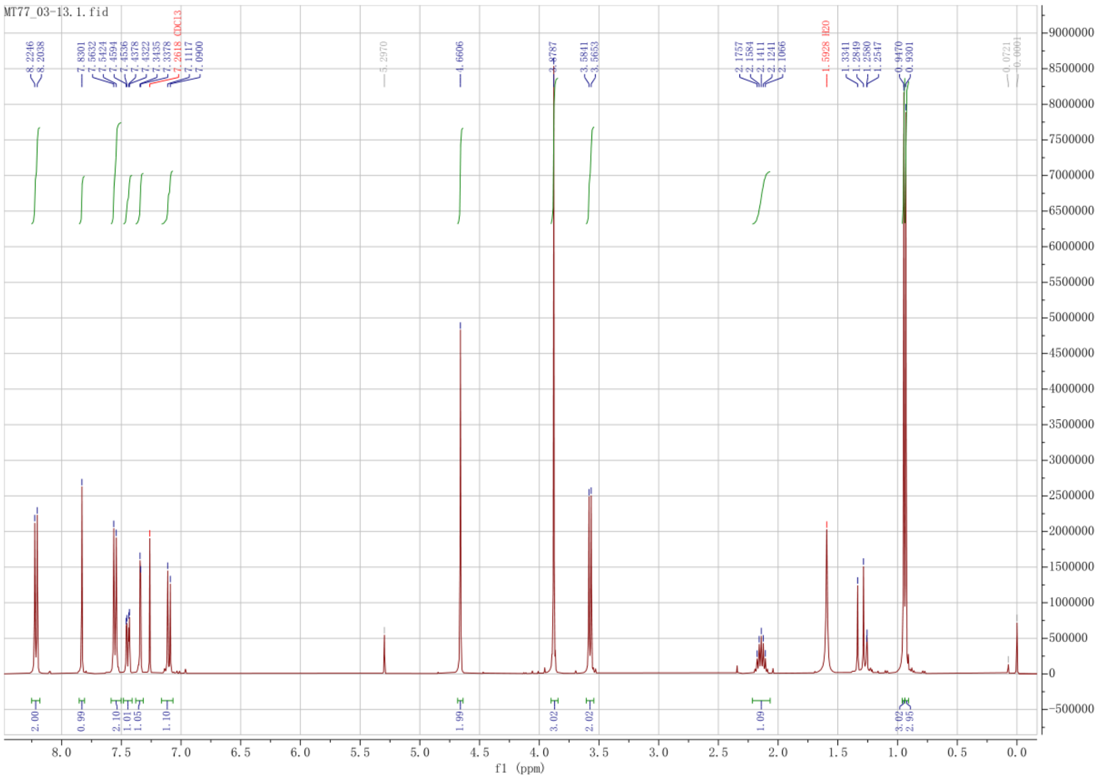

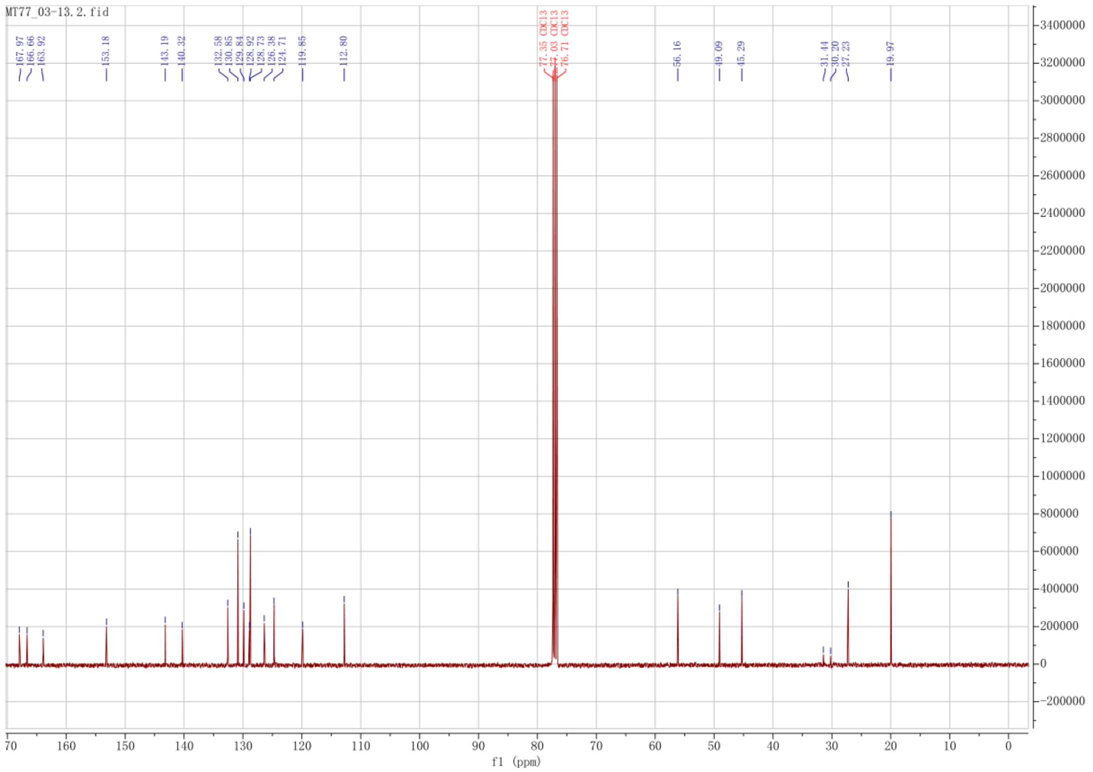

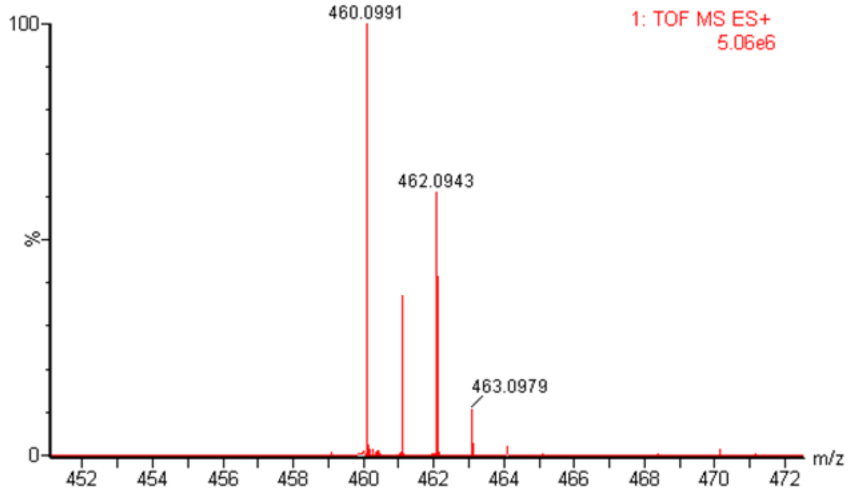

^{1}H NMR, ^{13}C NMR and HR-MS Spectra of compound 36

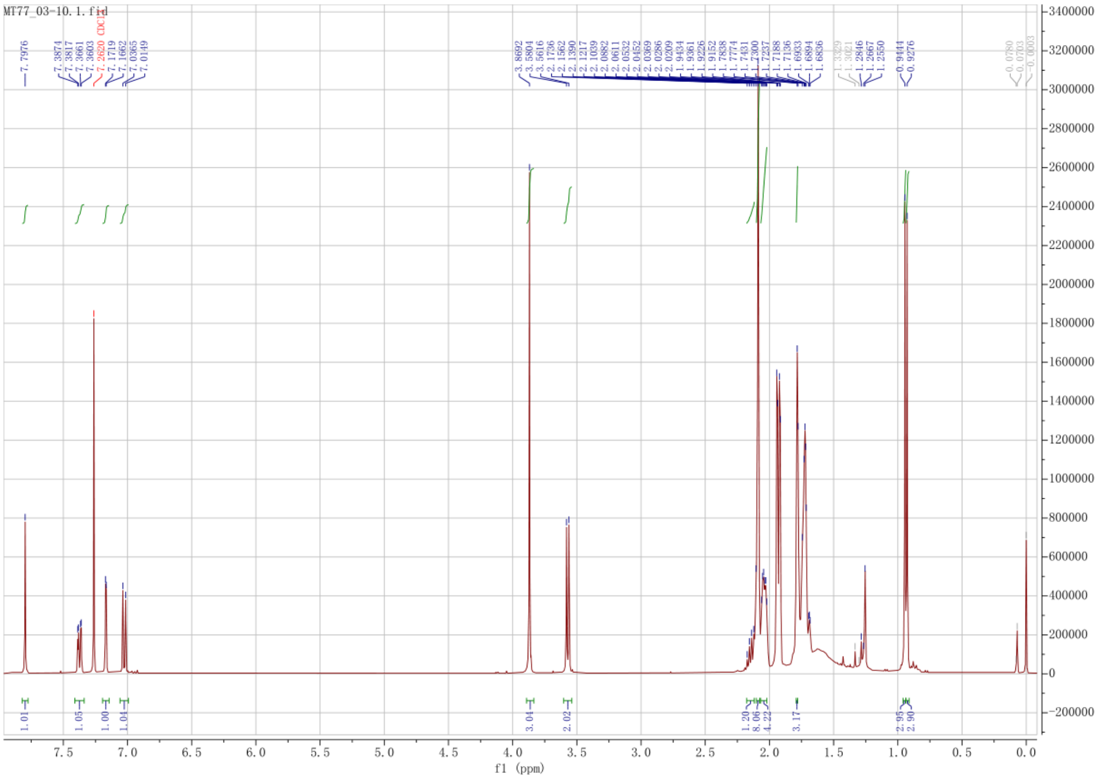

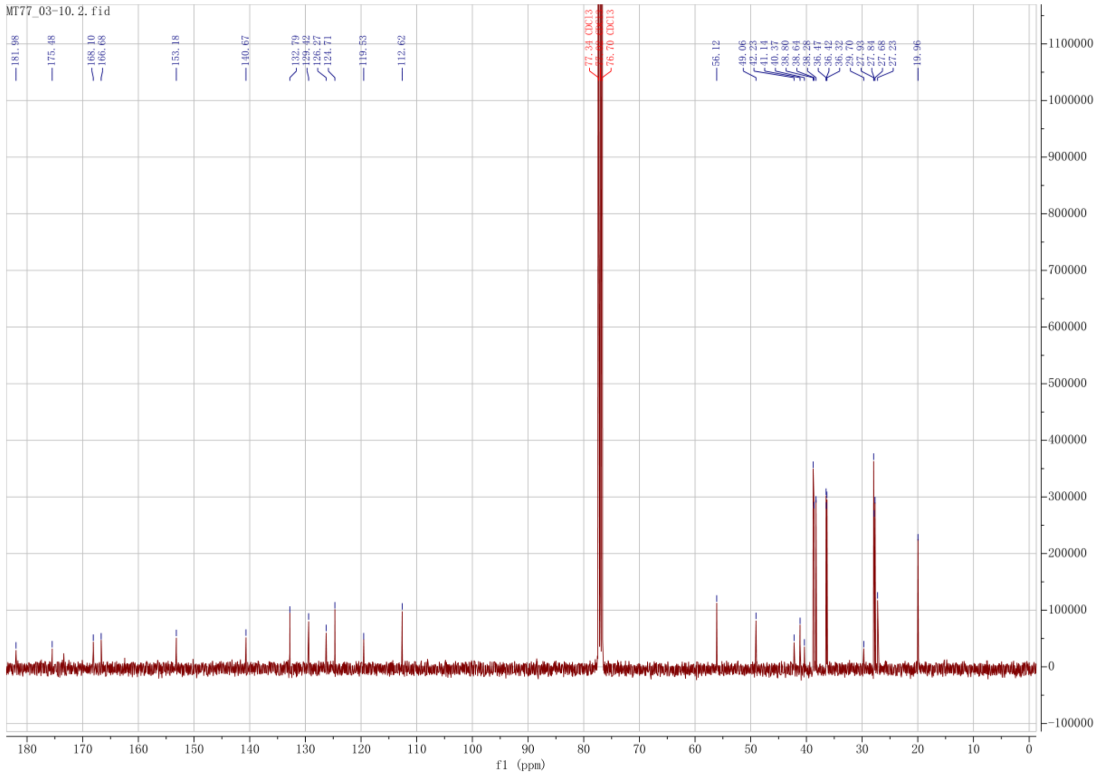

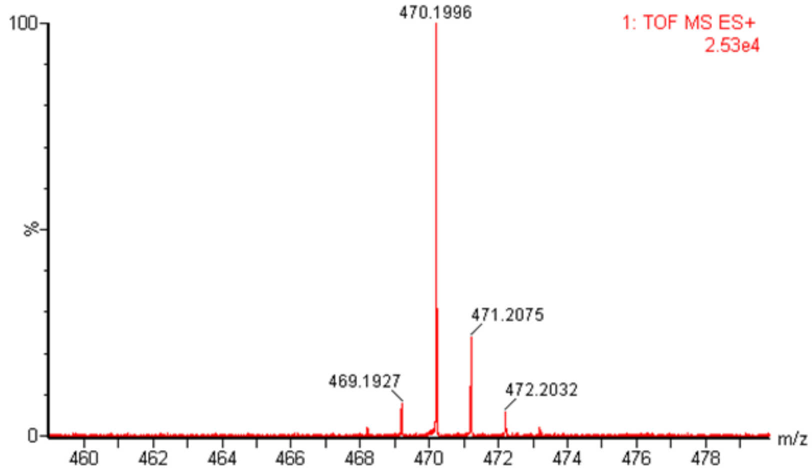

^{1}H NMR, ^{13}C NMR and HR-MS Spectra of compound 37

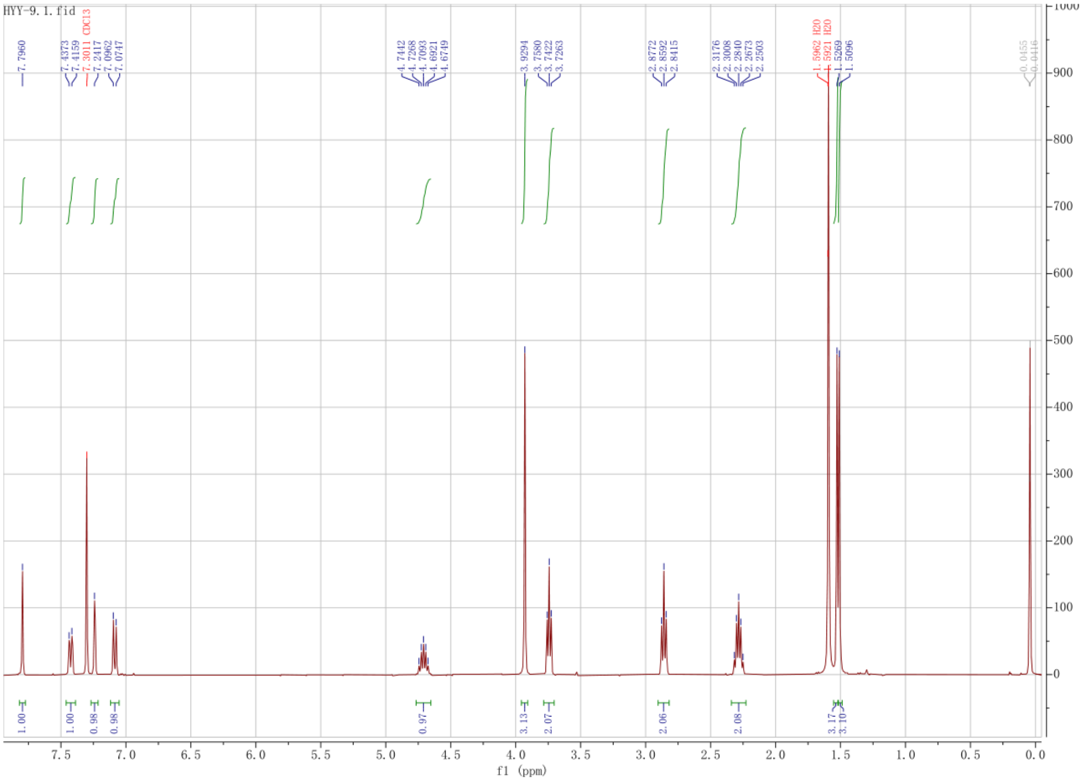

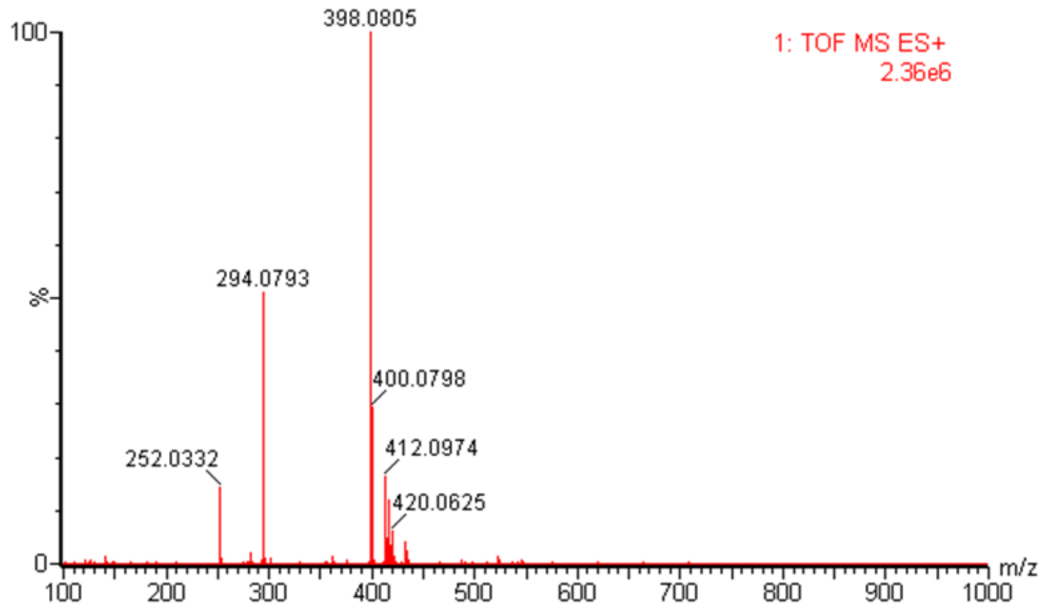

^{1}H NMR, ^{13}C NMR and HR-MS Spectra of compound 38

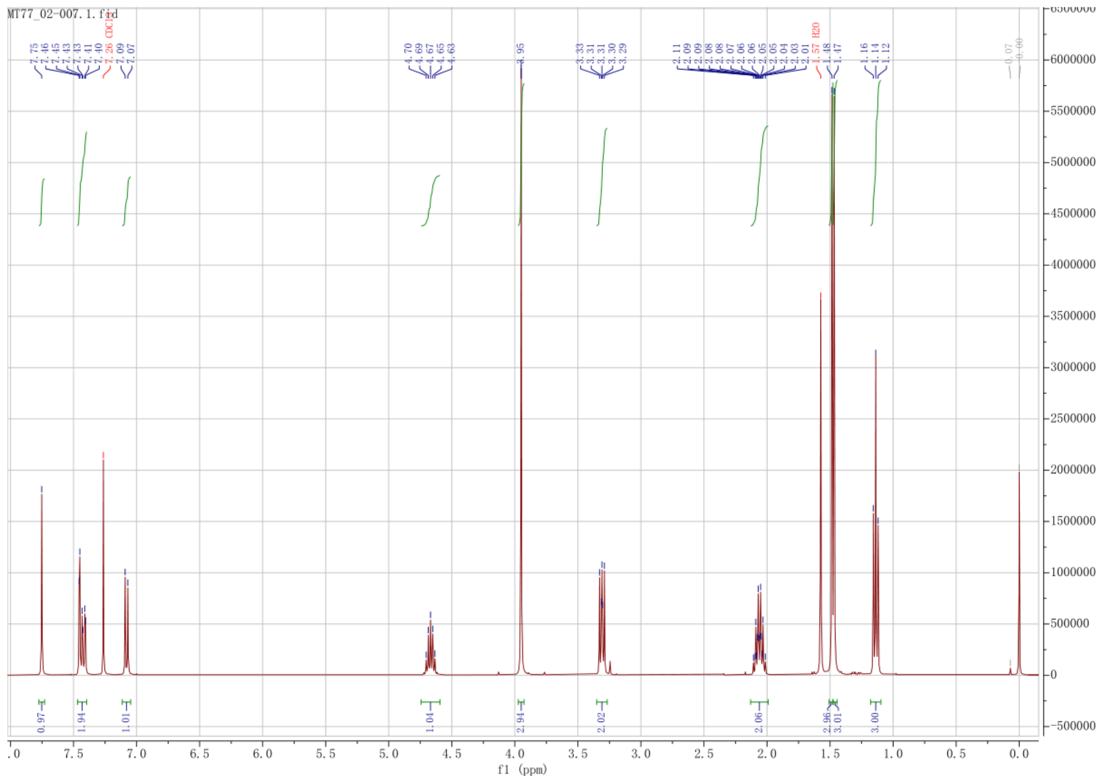

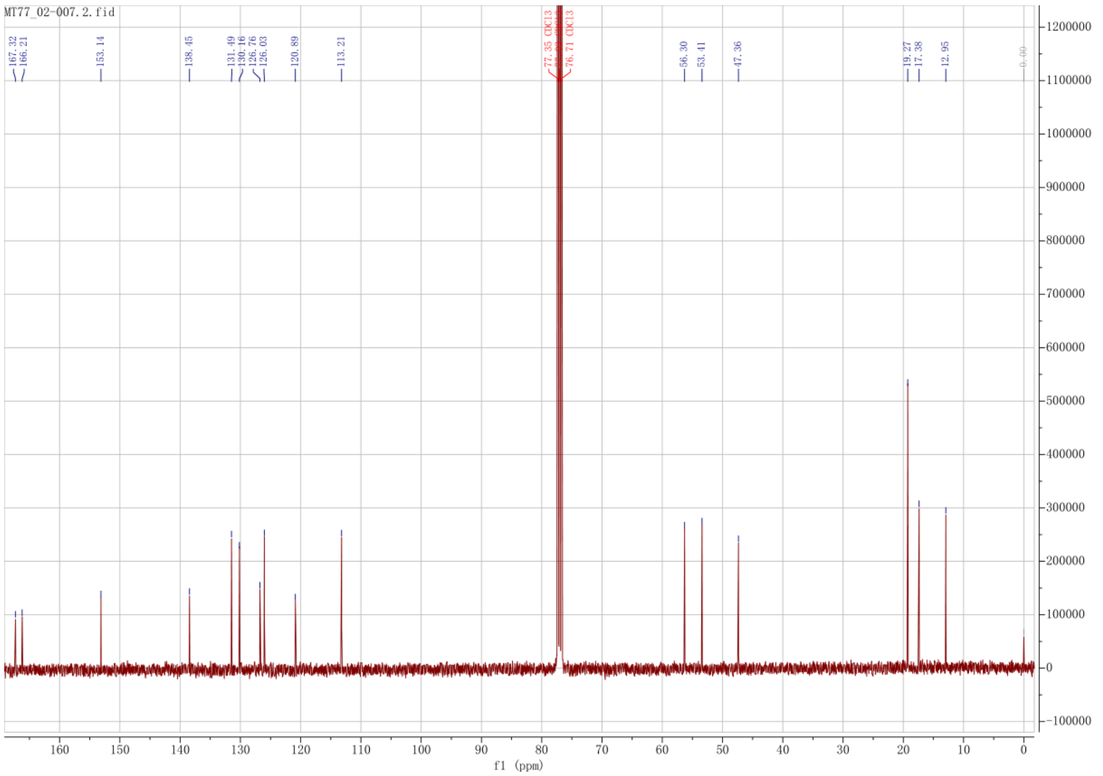

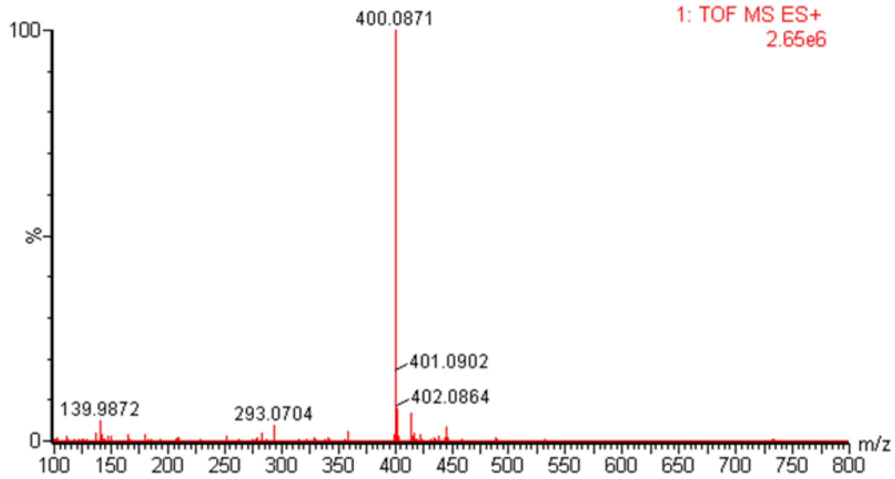

^{1}H NMR, ^{13}C NMR and HR-MS Spectra of compound 39

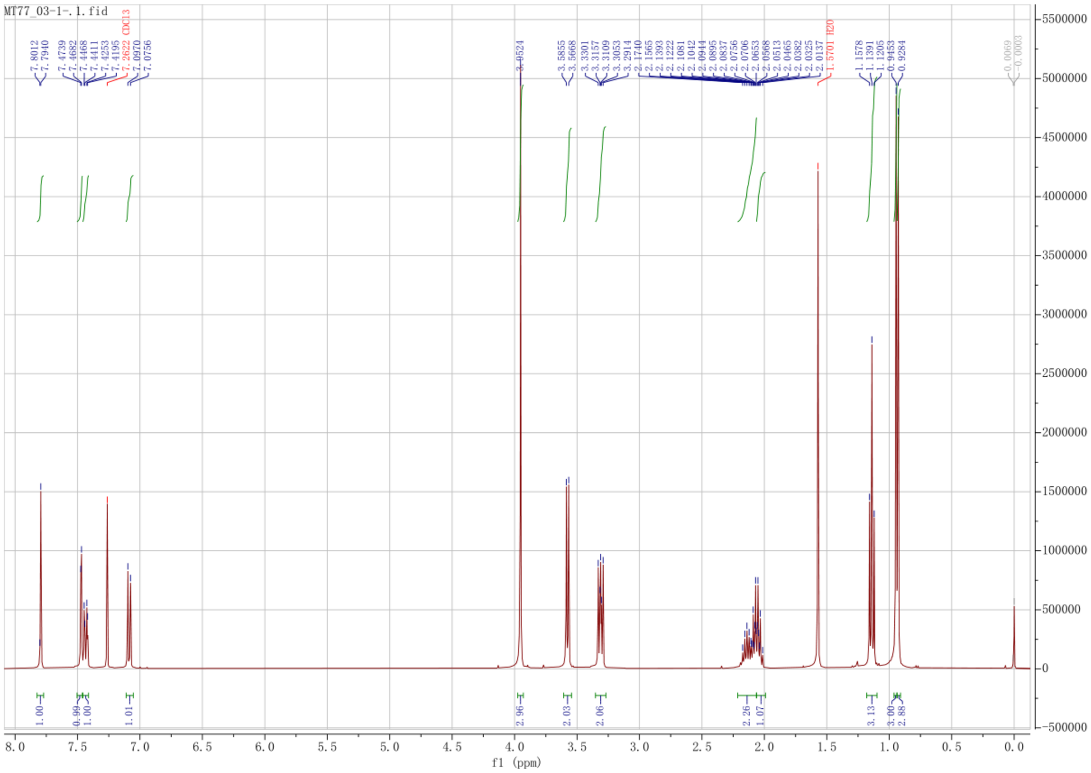
MT77_03-1-.1.fid
f1 (ppm)

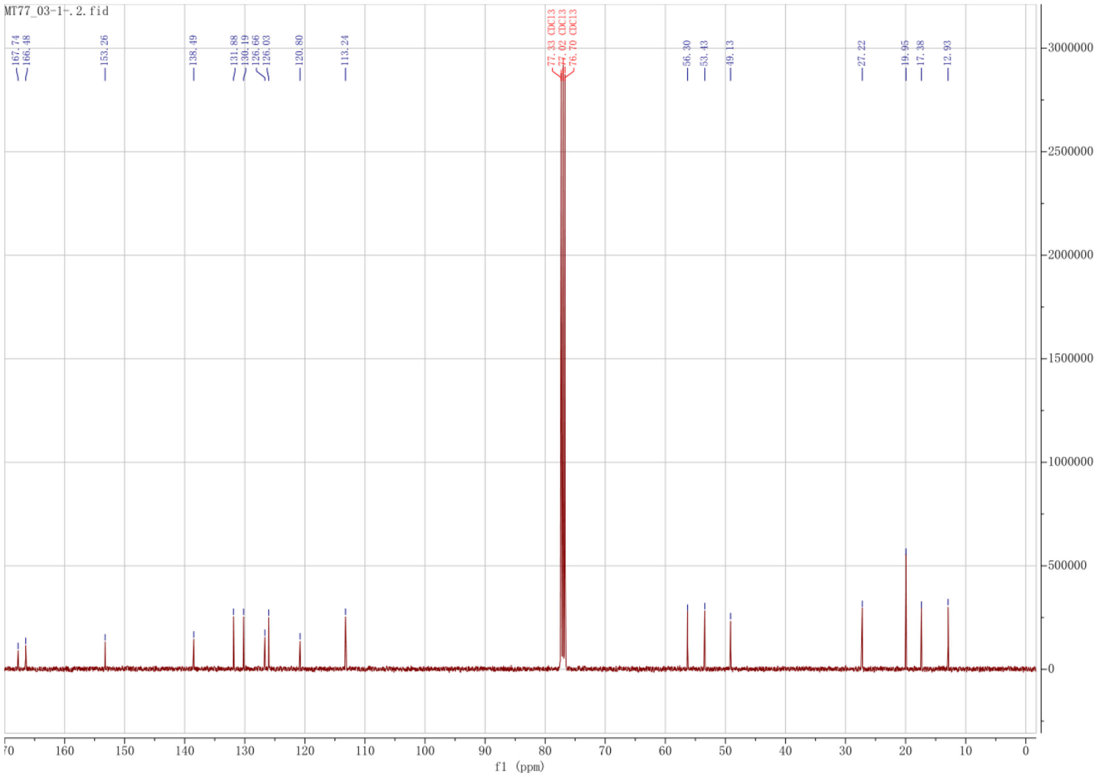
MT77_03-1-.2.fid
f1 (ppm)

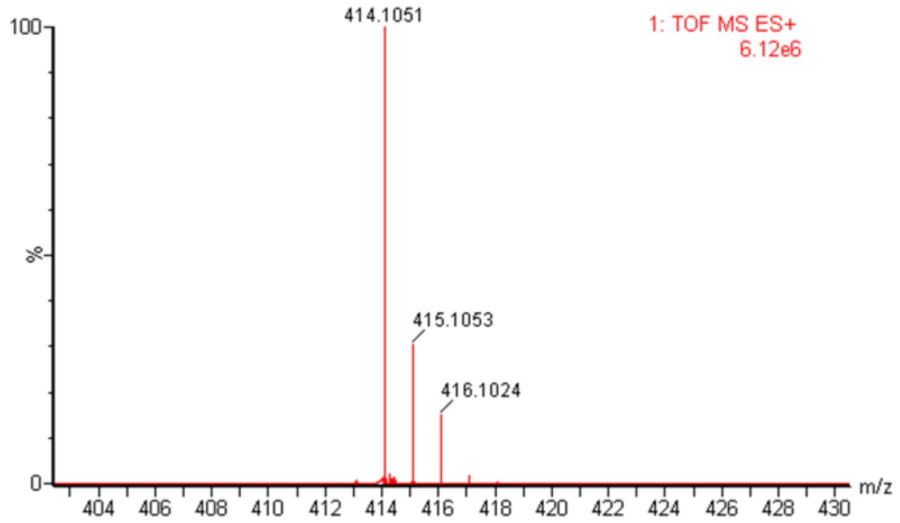

^{1}H NMR, ^{13}C NMR and HR-MS Spectra of compound 40

RMSD values of CB1 active, CB2 active, CB1 inactive and CB2 inactive systems sequentially along the MD simulation time. Black curves represent the backbone atoms of the transmembrane helices of CB1/CB2 (7TM); Green and red curves are the RMSD ligand without (No Fit) and with least-square fittings (LS Fit), respectively.

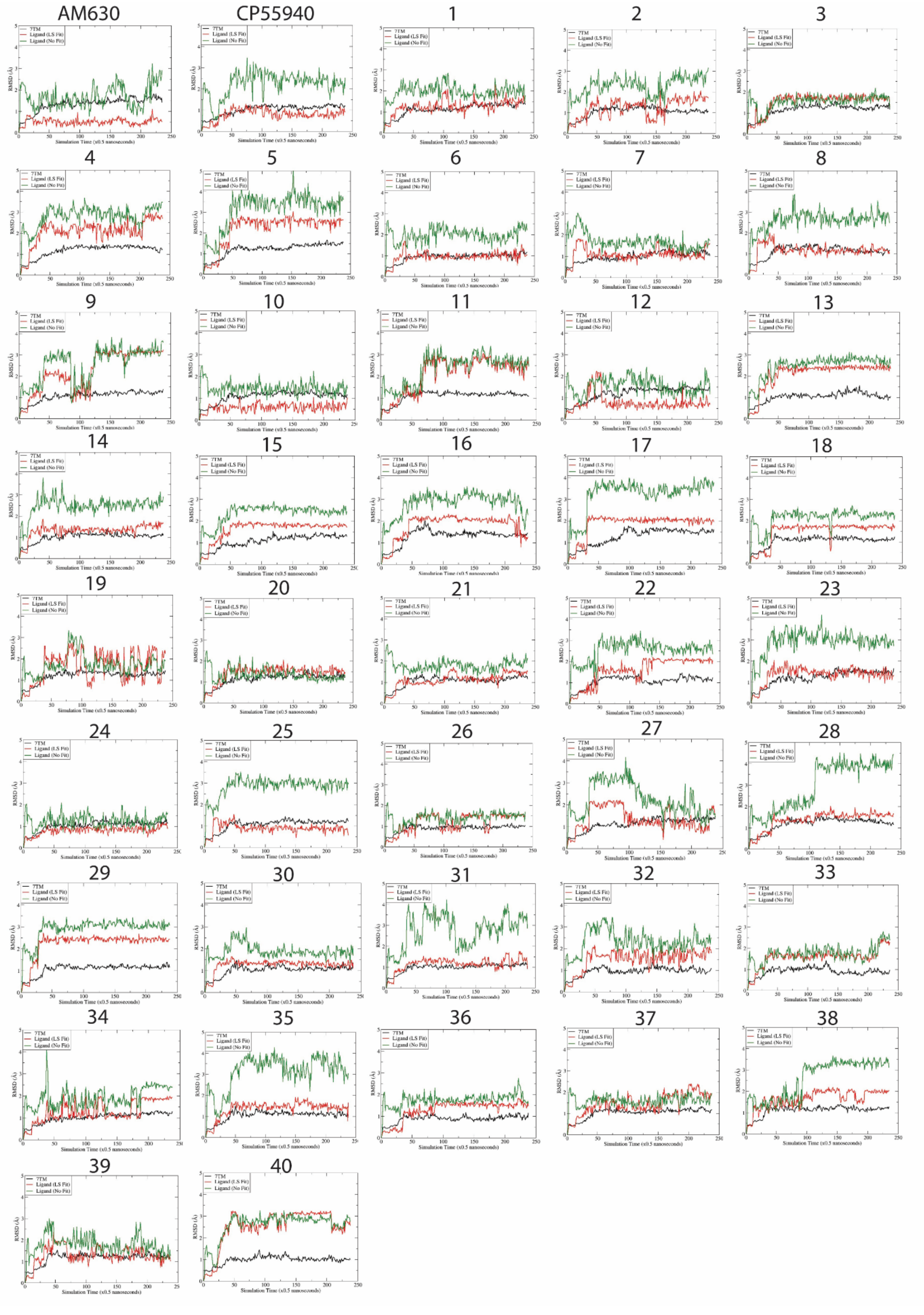

AM630
CP55940
1
2
3
4
5
6
7
8
9
10
11
12
13
14
15
16
17
18
19
20
21
22
23
24
25
26
27
28
29
30
31
32
33
34
35
36
37
38
39
40
7TM
Ligand (LS Fit)
Ligand (No Fit)
RMSD (Å)
Simulation Time (x0.5 nanoseconds)

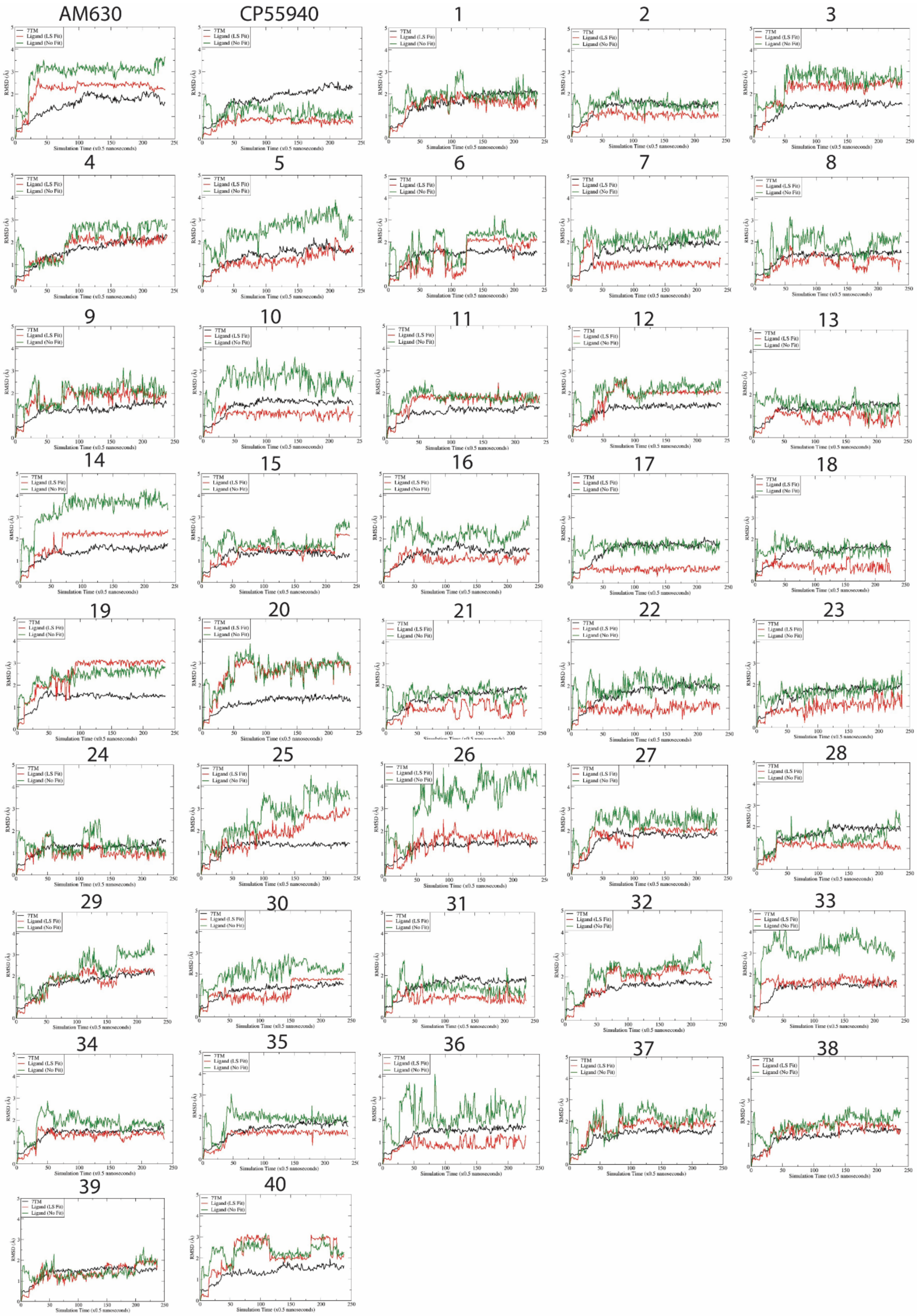
AM630
CP55940
1
2
3
4
5
6
7
8
9
10
11
12
13
14
15
16
17
18
19
20
21
22
23
24
25
26
27
28
29
30
31
32
33
34
35
36
37
38
39
40
7TM
Ligand (LS Fit)
Ligand (No Fit)
RMSD (Å)
Simulation Time (x0.5 nanoseconds)

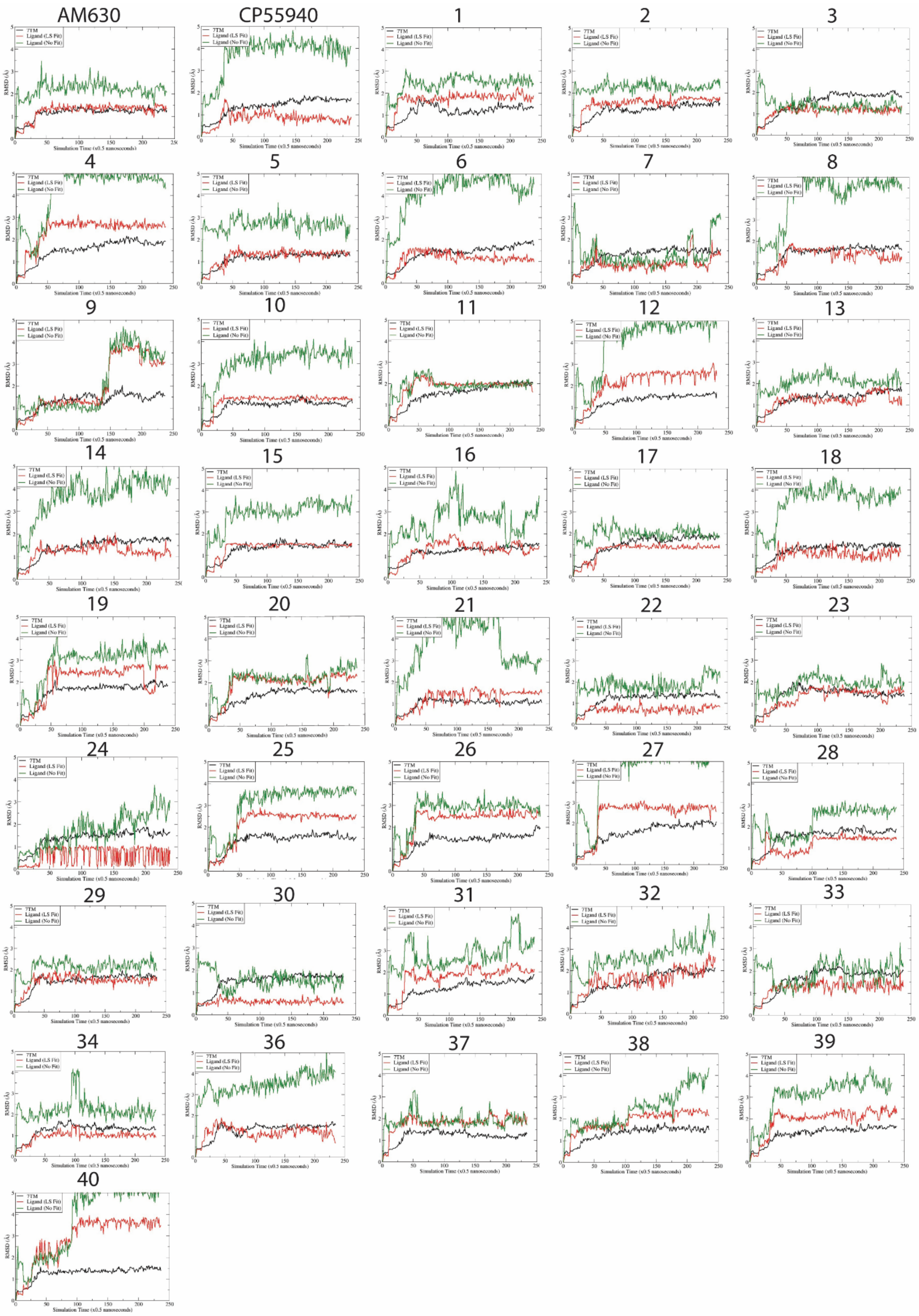
AM630
CP55940
1
2
3
4
5
6
7
8
9
10
11
12
13
14
15
16
17
18
19
20
21
22
23
24
25
26
27
28
29
30
31
32
33
34
36
37
38
39
40
7TM
Ligand (LS Fit)
Ligand (No Fit)
RMSD (Å)
Simulation Time (x0.5 nanoseconds)

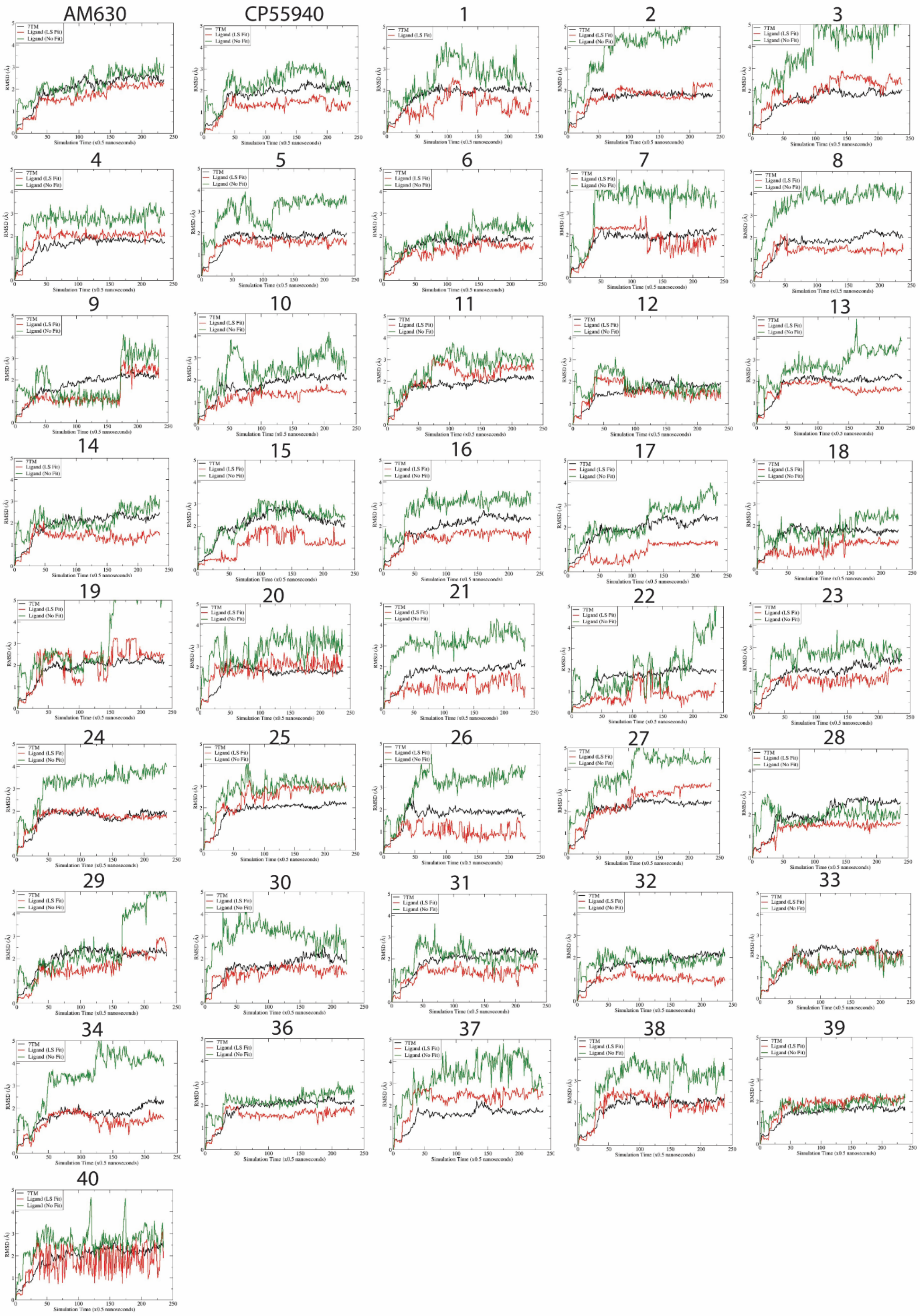
AM630
CP55940
1
2
3
4
5
6
7
8
9
10
11
12
13
14
15
16
17
18
19
20
21
22
23
24
25
26
27
28
29
30
31
32
33
34
36
37
38
39
40
7TM
Ligand (LS Fit)
Ligand (No Fit)
RMSD (Å)
Simulation Time (x0.5 nanoseconds)

Example of input file for MD simulation:

Molecular Dynamics Simulations

```
 &cntrl
   igb = 0, imin = 0, ntx = 5, irest = 1,
   ioutfm = 0, ntxo = 1, ntb = 2,
   maxcyc = 1000, ncyc = 100, drms = 0.001,
   ntpr = 1000, ntwr = 5000, ntwx = 5000, iwrap = 1,
   nstlim = 5000000, dt = 0.002,
   cut = 10.0, dielc = 1.0,
   ntc = 2, ntf = 2,
   ntt = 3, gamma_ln=5.0,
   tempi = 298.15, temp0 = 298.15,
   tautp = 0.5, ig = 71277,
   ntp = 2, taup = 0.5,
   ntr = 0, ibelly = 0,
 &end
```

Example of input file for trajectory analysis:

```
trajin prmcrd
trajin md1.trj.gz 1 10000 50
trajin md2.trj.gz 1 10000 50
trajin md3.trj.gz 1 10000 50
trajin md4.trj.gz 1 10000 50
trajin md5.trj.gz 1 10000 50
trajin md6.trj.gz 1 10000 50
trajin md7.trj.gz 1 10000 50
trajin md8.trj.gz 1 10000 50
trajin md9.trj.gz 1 10000 50
trajin md10.trj.gz 1 10000 50
trajin md11.trj.gz 1 10000 50
trajin md12.trj.gz 1 10000 50
trajin md13.trj.gz 1 10000 50
trajin md14.trj.gz 1 10000 50
trajin md15.trj.gz 1 10000 50
trajin md16.trj.gz 1 10000 50
trajin md17.trj.gz 1 10000 50
trajin md18.trj.gz 1 10000 50
trajin md19.trj.gz 1 10000 50
trajin md20.trj.gz 1 10000 50

center  :1-295

autoimage
strip :WAT
strip :K+
```

```
strip :Na+
strip :Cl-
strip :PA
strip :PC
strip :OL

rms first out rmsd.dat          :1-294@CA,C,N,O
rms first out rmsd_LIG.dat      :295

rms first out rmsd_1_20.dat     :1-20@CA,C,N,O
rms first out rmsd_56_61.dat    :56-61@CA,C,N,O
rms first out rmsd_89_93.dat    :89-93@CA,C,N,O
rms first out rmsd_128_137.dat  :128-137@CA,C,N,O
rms first out rmsd_162_178.dat  :162-178@CA,C,N,O
rms first out rmsd_212_218.dat  :212-218@CA,C,N,O
rms first out rmsd_249_252.dat  :249-252@CA,C,N,O
rms first out rmsd_281_294.dat  :281-294@CA,C,N,O

rms first out rmsd_7TM.dat      :21-55,62-88,94-127,138-161,179-211,219-248,253-
280@CA,C,N,O

average average.pdb pdb
trajout snapshot pdb multi
```
